# Aggregation, Photoluminescence, and Cytotoxicity of Pt(II) and Re(I) Complexes Bearing Multimodal‐Coordinating Luminophores

**DOI:** 10.1002/chem.202404115

**Published:** 2025-06-04

**Authors:** Stefan Buss, Lorenz Borsdorf, Elisabeth C. Muschiol, María Victoria Cappellari, Iván Maisuls, Toni M. A. Weise, Alexander Hepp, Jutta Kösters, Melanie Esselen, Gustavo Fernández, Cristian A. Strassert

**Affiliations:** ^1^ Institut für Anorganische und Analytische Chemie, Universität Münster Corrensstraße 28/30 Münster Germany; ^2^ CiMIC, SoN, CeNTech Universität Münster Heisenbergstraße 11 Münster Germany; ^3^ Organisch‐Chemisches Institut Universität Münster Corrensstraße 36 Münster Germany; ^4^ Institut für Lebensmittelchemie Universität Münster Corrensstraße 45 Münster Germany

**Keywords:** aggregation, photoluminescence, cytotoxicity, Pt⋯Pt interactions, Pt(II) complexes

## Abstract

In this study, a polydentate ligand capable of adopting multiple coordination modes (N*N, C^N*N, and C^N*N^C) was established. Depending on the chelation conditions and the co‐ligand used in the subsequent steps, nine complexes became accessible. The four Pt(II)‐based species with N*N‐coordination motif show tunable emission and cytotoxicity. Two Pt(II) complexes with C^N*N‐type coordination were also obtained, and the exchange of the co‐ligand from chlorido to cyanido led to an enhancement in the photoluminescence quantum yield from <0.02 to 0.55. These compounds exhibit distinct solid‐state characteristics associated with Pt⋯Pt‐interactions. Interestingly, the tetradentate C^N*N^C‐coordination mode was not accessible, most likely due to kinetic reasons and insolubility‐related purification hurdles. However, the use of a N*N‐coordination mode for a Re(I) center yielded non‐cytotoxic ^3^MLCT‐state emitters. The multifaceted coordination modes available with the luminophoric ligand enable the design and realization of diverse metal complexes with tailored cytotoxicity and tendency towards intermolecular coupling, both in solution and in crystalline phases.

## Graphical abstract

A polydentate ligand with multiple coordination patterns (bidentate N*N‐mode for Pt(II) and Re(I) centers, tridentate C^N*N‐motive for Pt(II)‐based complexes) is herein presented. The attained coordination‐chemical versatility in combination with different co‐ligands was employed to achieve sophisticated structural features and tunable photophysical properties, including aggregation and cytotoxicity.

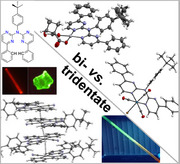

## 1 Introduction

Due to their broad range of possible applications, Pt(II) complexes based on multidentate ligands have been in the focus of different research fields for many years. For Pt(II)‐based triplet emitters, their use ranges from light‐emitting devices^[^ ^1^, ^2^, ^3^, ^4^, ^5^, ^6^ ^]^ and sensors^[^ ^7^, ^8^, ^9^, ^10^ ^]^ over photocatalysis^[^ ^11^, ^12^, ^13^, ^14^, ^15^ ^]^ to bio‐imaging^[^ ^16^, ^17^, ^18^ ^]^ and photodynamic therapy.^[^ ^19^, ^20^, ^21^ ^]^ In addition to the strong spin orbit coupling (SOC) and the resulting fast intersystem crossing (ISC) into the triplet manifold as well as the efficient phosphorescent decay from the triplet state supported by the Pt(II)‐center, the square planar coordination stemming from the *d* ^8^‐configuration enables interactions involving the $d_{z^{2}}$‐orbitals, e.g., by aggregation involving Pt⋯Pt interactions.^[^ ^22^, ^23^, ^24^ ^]^ Multidentate ligands can increase the rigidity and suppress the thermal population of dissociative states, which results in improved photophysical properties.^[^ ^25^ ^]^ Additionally, cyclometallated phenylido‐ligands constitute strong *σ*‐donors, which increase the ligand field splitting and destabilize the antibonding metal‐centered states, which in turn disfavors radiationless deactivation paths.^[^ ^26^ ^]^

**Figure 1:**
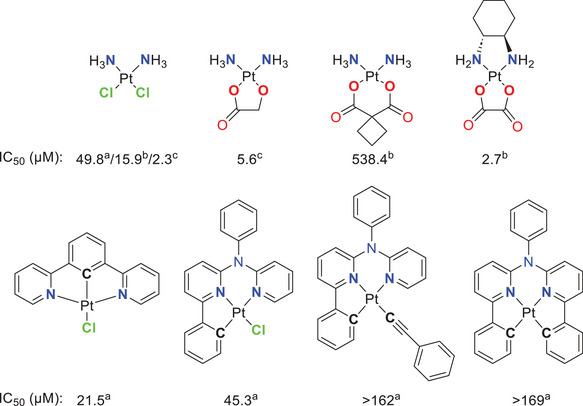
Structural formulae and IC_50_‐values (µm) for cisplatin and Pt(II)‐based triplet emitters presented by Huo and coworkers,^[^ ^35^ ^]^ along with nedaplatin,^[^ ^36^ ^]^ carboplatin and oxaliplatin.^[^ ^37^ ^]^ (a) Against NCI‐H522 lung cancer; (b) against HepG2 liver cancer; (c) averaged over four different cell lines, namely: PC‐9 and PC‐14 human luminary adenocarcinoma as well as MKN‐45 and KATO III human stomach adenocarcinoma.

The aforementioned factors render Pt(II)‐based complexes with cyclometallated ligands as privileged functional probes for various applications.^[^ ^2^, ^14^, ^23^, ^27^, ^28^ ^]^ For example, Pt(II) complexes with bidentate chromophores can exhibit outstanding photophysical properties when the proper co‐ligands are introduced.^[^ ^29^, ^30^, ^31^, ^32^, ^33^ ^]^ Another application for tunable Pt(II) complexes with bidentate ligands involves the development of cytostatic drugs. For instance, nedaplatin, carboplatin and oxaliplatin differ from the well‐known cisplatin by the exchange of two monodentate co‐ligands for bidentate units. In nedaplatin, the chlorido ligands are replaced by glycolic acid (GlyH_2_), which enhances the water solubility. Carboplatin uses cyclobutyl‐1,1‐dicarboxylate (cbda^2−^) as a ligand, altering the complex´s reactivity and resulting in a different toxicity profile. Additionally, oxaliplatin (which incorporates an oxalate ligand) uses (1*R*,2*R*)‐1,2‐diaminocyclohexane as a nitrogen donor (Figure 1).^[^ ^34^ ^]^

In addition to the photophysical properties, the cytotoxicity of Pt(II) complexes based on tridentate ligands is of great relevance in current research. In this regard, Huo and co‐workers compared the cytotoxicity of cisplatin with triplet‐emitting Pt(II) complexes (Figure 1) bearing cyclometallating multidentate ligands. For the mono‐cyclometallated Pt(II) complexes bearing a tridentate chelator and a chlorido co‐ligand, similar or even lower IC_50_ values than cisplatin were observed, whereas the complexes with an additional C─Pt‐bond show low cytotoxicity.^[^ ^35^ ^]^

**Figure 2:**
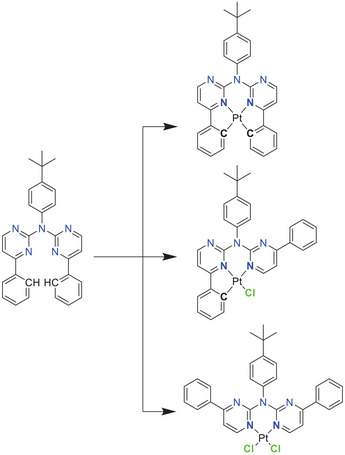
Ligand design and different possible coordination modes.

The coordination mode of a ligand is primarily determined by its design. We herein aimed to develop a versatile ligand (Figure 2) that can coordinate in different modes, depending on the reaction conditions, the chosen metal center and the intended application. Hence, achieving *bis*‐cyclometallation can represent a kinetic coordination barrier.^[^ ^38^, ^39^ ^]^ If combined with Pt(II) centers, we reasoned that the N*N‐bidentate coordination mode may be useful against neoplastic cells, due to the resemblance with respect to cisplatin.^[^ ^40^ ^]^ In this context, the two chlorido ligands can be replaced by a functional bidentate ligand to enhance the chemical properties, such as solubility and aggregation tendency. The N*N‐bidentate coordination mode was further extended beyond the Pt(II) center, as the resulting Re(I) complexes display a useful phosphorescence for bioimaging, even if neutral N^N‐donors are used.^[^ ^41^, ^42^, ^43^, ^44^, ^45^, ^46^, ^47^, ^48^, ^49^, ^50^, ^51^ ^]^ For optical imaging in biomedicine, a low cytotoxicity is essential. Thus, we further evaluated the cytotoxicity of the herein presented complexes. Finally, the tridentate C^N*N coordination mode results in triplet emitters with excellent photophysical properties, where the fourth exchangeable co‐ligand could potentially allow for the incorporation of monodentate units with targeting groups for optical imaging or singlet dioxygen photogeneration towards theranostics.^[^ ^52^, ^53^ ^]^ We observed that the combination of the pyrimidine and the enlarged *π*‐system induces aggregation in the solid state, as well as weak self‐association in dichloromethane (DCM) solutions. In the future, *bis*‐cyclometallation is expected to yield more versatile and robust triplet emitters with an even higher tendency towards self‐assembly via intermolecular stacking upon aggregation.

## 2 Results and Discussion

### 2.1 General Information

General information about experimental procedures including instrumental and synthetic methods, structural characterization of the ligand precursors and the complexes as well as photophysical measurements are provided in the Supporting Information.

#### 2.1.1 Materials

All chemicals were used as purchased from commercially available sources. For the photophysical measurements, spectroscopic grade solvents (Uvasol) were used.

#### 2.1.2 Synthesis

The detailed synthetic procedures and analytical data are provided in the Supporting Information. The new compounds were characterized by ^1^H‐ and ^19^F‐, ^13^C‐, ^31^P‐, ^195^Pt‐, and 2D‐nuclear magnetic resonance spectroscopies (NMR, Figures S1–S161, Supporting Information) as well as by mass spectrometry (EM‐ESI‐MS or MALDI‐MS). The presence of cyanido and carbonyl co‐ligands were confirmed via infra‐red (IR) spectra and given in Figures S62 and S63, Supporting Information.

#### 2.1.3 X‐ray diffractometry

Suitable single crystals for X‐ray diffraction measurements were obtained by slowly evaporating the solvent from a saturated DCM solution or by diffusion of cyclohexane into such a solution, if not noted otherwise. The full set of data is given in Tables S1–S11 and Figures S64–S95, Supporting Information), and have been uploaded to the CCDC database. The molecular structures were graphically processed using the software package Mercury from CCDC.^[^ ^54^ ^]^

Deposition numbers 2 394 603 (for **1**), 2 394 610 (for **LH_2_**), 2 394 604 (for [**PtLH_2_Cl_2_**]), 2 394 611 (for [**PtLH_2_cbda**]), 2 394 607 (for [**PtLHCl**]), 2 394 606 (for [**PtLHCN**](yellow)), 2 394 609 (for [**PtLHCN**](orange)), 2 394 605 (for [**PtLH_2_(CO)_3_Br**]) and 2 394 608 (for [**PtLH_2_(CO)_3_Cl**]) contain the supplementary crystallographic data for this paper. These data are provided free of charge by the joint Cambridge Crystallographic Data Centre and Fachinformationszentrum Karlsruhe (http://www.ccdc.cam.ac.uk/structures).

#### 2.1.4 Steady‐state and time‐resolved photoluminescence spectroscopy

The photophysical properties are summarized in Table 1 and Figures 11, 12, 13, 14 (the complete set of data is detailed in Tables S12–S14, with the spectra and photoluminescence decay plots shown in Figures S96–S155, Supporting Information).

**Table 1:** Selected photophysical data for the thiazole‐based coordination compounds and the reference complexes. The complete set of data provided in Table S12, Supporting Information.

Complex	λ_abs_ /nm [*ε*/10^3^ m ^−1^ cm^−1^]	Medium [*T*/K]	*λ*_Em_^[^ ^a^ ^]^/nm	$\tau_{\mathrm{av}}$ ^[^ ^b^ ^]^/µs	Φ_L_
**[PtCl(L_Ref1_)]^[^** ^61^ ^]^	265 (30.8), 277 (28.0), 313 (12.0), 348 (12.1), 368 (7.8)	DCM, Ar (298)	494	0.018	<0.02
		Glassy matrix (77)	484	31	0.98
**[PtCN(L_Ref1_)]** ^[^ ^61^ **^]^**	255 (28.2), 265 (30.5), 275 (28.7), 300 (19.0), 339 (12.2), 355 (13.6), 395 (1.5)	DCM, Ar (298)	489	33.5	0.46
		Glassy matrix (77)	486	42	0.98
**[PtLH_2_Cl_2_]**	287 (49.1), 325 (20.2), 376sh (4.4)	DCM, Ar (298)	n.d.	n.d.	n.d.
		Glassy matrix (77)	n.d.	n.d.	n.d.
**[PtLH_2_Gly]**	287 (43.5), 328 (17.0), 379sh (5.2)	DCM, Ar (298)	501	2.45	<0.02
		Glassy matrix (77)	452	121.8	n.d.
**[PtLH_2_cbda]**	287 (57.9), 319sh (30.5), 355sh (11.1)	DCM, Ar (298)	n.d.	n.d.	n.d.
		Glassy matrix (77)	450	120.0	n.d.
**[PtLH_2_Tsgly]**	286 (50.8), 336 (16.8), 377 (8.2)	DCM, Ar (298)	n.d.	n.d.	n.d.
		Glassy matrix (77)	468	86.3	n.d.
**[PtLHCl]**	263 (33.4), 305 (28.2), 345 (15.0), 387 (6.3)	DCM, Ar (298)	509	0.023	<0.02
		Glassy matrix (77)	484	8.04	n.d.
**[PtLHCN]**	257 (35.8), 288 (39.6), 340 (24.0), 399 (2.7), 422sh, (1.6)	DCM, Ar (298)	494	10.34	0.55 ± 0.03
		Glassy matrix (77)	477	19.26	n.d.
**[ReLH_2_(CO)_3_Br]**	285 (44.7), 349 (13.8)	DCM, Ar (298)	600	0.137	<0.02
		Glassy matrix (77)	520	28.28	n.d.
**[ReLH_2_(CO)_3_Cl]**	283 (48.9), 348 (15.5)	DCM, Ar (298)	596	0.141	<0.02
		Glassy matrix (77)	522	26.33	n.d.
**[ReLH_2_(CO)_3_CN]**	284 (45.6), 341 (15.5)	DCM, Ar (298)	584	0.721	<0.02
		Glassy matrix (77)	512	49.5	n.d.

^[a]^ The highest energy band is indicated;

^[b]^ *λ*_exc_ = 376 nm, expressed as amplitude‐weighted averaged lifetimes according to the suggestions from the work of Engelborghs and Sillen^[^ ^74^ ^]^ (the single exponential components, relative amplitudes and propagated uncertainties are listed in Table S12, Supporting Information).

#### 2.1.5 Aggregation studies

The variable temperature (VT)‐^1^H‐NMR‐spectra were measured as described for the structural characterization (see *Synthesis* part). The spectra, fittings and extracted values are provided in Figures S156–S175 and Tables S15–S18, Supporting Information.

#### 2.1.6 Cytotoxicity tests

The cytotoxicity of the synthesized complexes towards the HepG2 liver carcinoma cell line was assessed using the resazurin assay.^[^ ^55^ ^]^ Cells were grown in Dulbecco's modified eagle's medium (DMEM) supplemented with 10% (*v*/*v*) of fetal calf serum (FCS), 100 units/mL penicillin and 0.1 mg/mL streptomycin at 37 °C and 5% CO_2_ in a humidified atmosphere. Detailed information can be found in the Supporting Information. The data is reported in Figure 16 as well as in Table S19, Supporting Information.

### 2.2 Synthesis

The required intermediates for the tetradentate ligand precursors (i.e., the proto‐ligands), were usually synthesized using suitable 2,6‐dibromopyridine derivatives,^[^ ^27^, ^56^, ^57^ ^]^ which can result in side products with a double substitution at the pyridine ring. The herein used commercially available 2‐chloro‐4‐phenyl‐pyrimidine bypasses this problem. This results in a one‐step synthesis for the preparation of the ligand precursor **LH_2_** by a employing a Buchwald–Hartwig cross‐coupling with 4‐*^t^* butylaniline. Using 2.0 equivalents of the chlorinated species leads to the isolation of the disubstituted product **LH_2_** in 49% yield, but also the monosubstituted secondary amine product **1** (see SI for more information) in 42% yield. Utilization of **1** in a subsequent Buchwald‐Hartwig reaction yields **LH_2_** as the only product in 90%. With this polydentate ligand precursor we explored different coordination modes (bi‐, tri‐, and tetradentate) while binding to Pt(II) centers.

**Figure 3:**
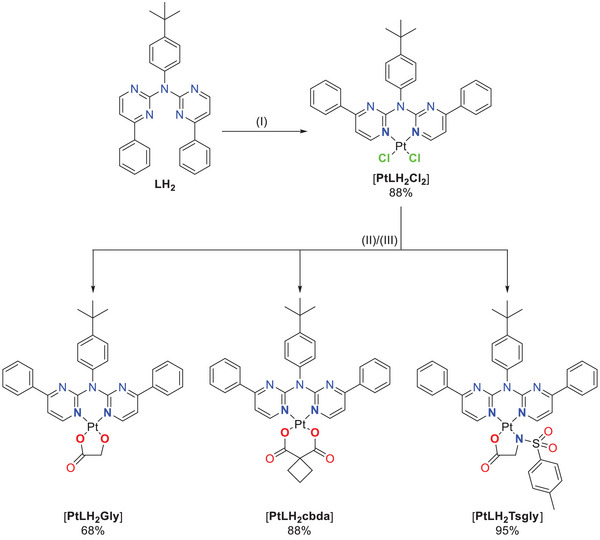
N*N‐type bidentate coordination of Pt(II) with **LH_2_** and exchange of the chlorido ligands by **L_2_H_2_** (GlyH_2_; cbdaH_2_; TsglyH_2_) to yield [**PtLH_2_L_2_**]. (I) Bidentate coordination: [**PtCl_2_DMSO_2_**], DCM, room temperature, 120 h, yielding 88%. Ligand exchange: (II) AgTFA, MeOH, room temperature, 1 h; (III) **L_2_Na_2_** (**L_2_H_2_**, NaOMe, MeOH, room temperature, 1 h), MeOH, reflux, 4 h (yielding 68% for **L_2_H_2_** = GlyH_2_, 88% for cbdaH_2_, 95% for TsglyH_2_).

The bidentate coordination via the two nitrogen atoms of this ligand (yielding [**PtLH_2_Cl_2_**]) was achieved by stirring the free ligand with the DMSO‐Pt(II) complex [**PtCl_2_DMSO_2_**] in DCM for multiple days. Inspired by cytostatic agents (nedaplatin and carboplatin), an exchange of the chlorido ligands was performed. First, glycolic acid (GlyH_2_, as used in nedaplatin) and cyclobutyl‐1,1‐dicarboxylic acid (cbdaH_2_, as used in carboplatin) as oxygen donors were introduced to yield [**PtLH_2_Gly**] or [**PtLH_2_cbda**] respectively. To complete the series, an amino acid derivative was chosen as the bidentate ligand. The simplest amino acid glycine has the tendency to coordinate in an mono‐anionic fashion;^[^ ^58^, ^59^ ^]^ thus, we used the *N*‐*p*‐toluenesulfonylated glycine‐derivative (TsglyH_2_), which acts as a dianionic bidentate ligand^[^ ^60^ ^]^ and resulted in the overall neutral complex [**PtLH_2_Tsgly**]. All three ligands were introduced by refluxing the mixture of a solution of the [**PtLH_2_Cl_2_**] (treated with silver(I) trifluoroacetate) with a solution of the co‐ligand precursor (treated with NaOMe), both in methanol (Figure 3). The attempted exchange to the cyanido co‐ligand only resulted in the isolation of the ligand precursor **LH_2_**.^[^ ^52^, ^61^ ^]^

**Figure 4:**
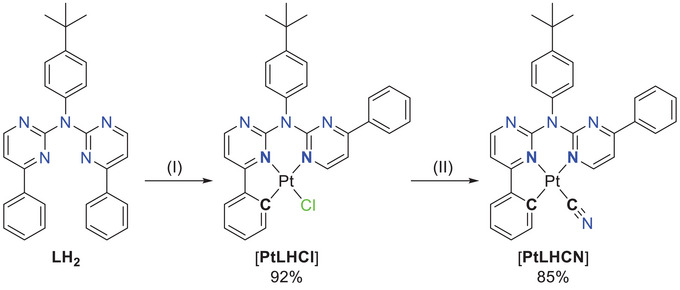
Tridentate C^N*N coordination of Pt(II) by mono‐cyclometallation with **LH_2_** and ligand exchange of the chlorido unit by cyanido to afford [**PtLHCN**]. (I) Mono‐cyclometallation: K_2_[PtCl_4_], ^N^Bu_4_NCl, glacial acetic acid, 250 °C, 1 h, max. 850 W, yield: 92%. (II) Ligand exchange: AgCN, MeCN, reflux, 16 h, yield: 85%.

The usual cyclometallation conditions (acetic acid, H_2_O/MeCN)^[^ ^52^, ^56^, ^57^, ^61^ ^]^ result in a complex product mixture with [**PtLH_2_Cl_2_**] and [**PtLHCl**] only in traces. A microwave‐assisted cyclometallation in acetic acid was used for the synthesis of [**PtLHCl**], which was obtained as a red solid. For complexes with C^N*N‐type ligands, the chlorido co‐ligand leads to poor photophysical performances. Thus, it was exchanged by the cyanido unit using AgCN in MeCN (Figure 4).^[^ ^61^ ^]^

Rourke and co‐workers showed that the bis‐cyclometallation for C^N^C‐type ligands results from a two‐step process, where the second cyclometallation can be achieved by precipitation of the product with H_2_O from a hot DMSO solution of the mono‐cyclometallated complex.^[^ ^38^ ^]^ Attempts towards *bis*‐cyclometallation treating [**PtLHCl**] under such conditions did not lead to the desired product.

**Figure 5:**
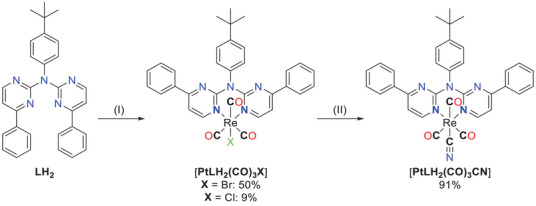
Bidentate N*N coordination of Re(I) with **LH_2_** and ligand exchange of the chlorido unit by cyanido to afford [**ReLH_2_(CO)_3_CN**]. (I) Bidentate coordination: [**Re(CO)_5_Br**], MeOH, reflux, 16 h. YIELDS: 50% with X = Br; 9% with Cl. (II) Ligand exchange: AgCN, MeCN, reflux, 16 h, yielding 91%.

For Pt(II) complexes, a strong *σ*‐donor (such as phenylido) is necessary to create emitters with advantageous photophysical properties. Conversely, coordination of Re(I) yields emitters that are potentially interesting for bioimaging, even with neutral N^N‐donors.^[^ ^41^, ^42^, ^43^, ^44^, ^45^, ^46^, ^47^, ^48^, ^49^, ^50^, ^51^ ^]^ Therefore, we extended our investigation to explore the bidentate N*N‐coordination mode of **LH_2_** (Figure 5) with Re(I). Refluxing [**Re(CO)_5_Br**] with **LH_2_** in MeOH yielded two emissive products, which were isolated via column chromatography. These products include the anticipated [**ReLH_2_(CO)_3_Br**] complex, along with the chlorido species [**ReLH_2_(CO)_3_Cl**]. However, an exchange of the bromine atom by chlorine from the DCM solvent seems unlikely, as both products are stable in DCM‐*d*_2_ for the NMR experiments. To enhance the ligand field splitting, we subsequently substituted the bromido unit by a cyanido ligand, as previously achieved with the Pt(II) complex. The carbonyl ligands were also detected via ATR‐FT‐IR studies (Figure S63, Supporting Information). The characteristic CO stretching bands are found in the range of 2100 to 1800 cm^−1^. Here, the bands can be attributed to stretching of the CO‐ligands (totally symmetric in‐phase (A´1), to totally symmetric out‐of‐phase (A´2) or to asymmetric (A´´, only the equatorial ligands), according to previous reports).^[^ ^45^, ^62^ ^]^

### 2.3 X‐ray diffractometry on single crystals

We confirmed the obtention of **1**, **LH_2_**, [**PtLH_2_Cl_2_**], [**PtLH_2_cbda**], [**PtLHCl**], [**PtLHCN**], [**ReLH_2_(CO)_3_Br**], and [**ReLH_2_(CO)_3_Cl**] by using X‐ray diffractometric analysis on single crystals and obtained the molecular structures, as shown in Figures 6, 7, 8, 9, 10 (further details are found in Figures S64–S95 and Tables S1–S11, Supporting Information). The ligand **LH_2_** and its precursor **1** crystalize in the monoclinic space group *P*2_1_/*n*, whereas [**PtLHCN**](yellow and orange) crystalizes in the space group *P*2_1_/*c*. All the other obtained crystal structures display a triclinic structure ($P\bar{1}$).

**Figure 6:**
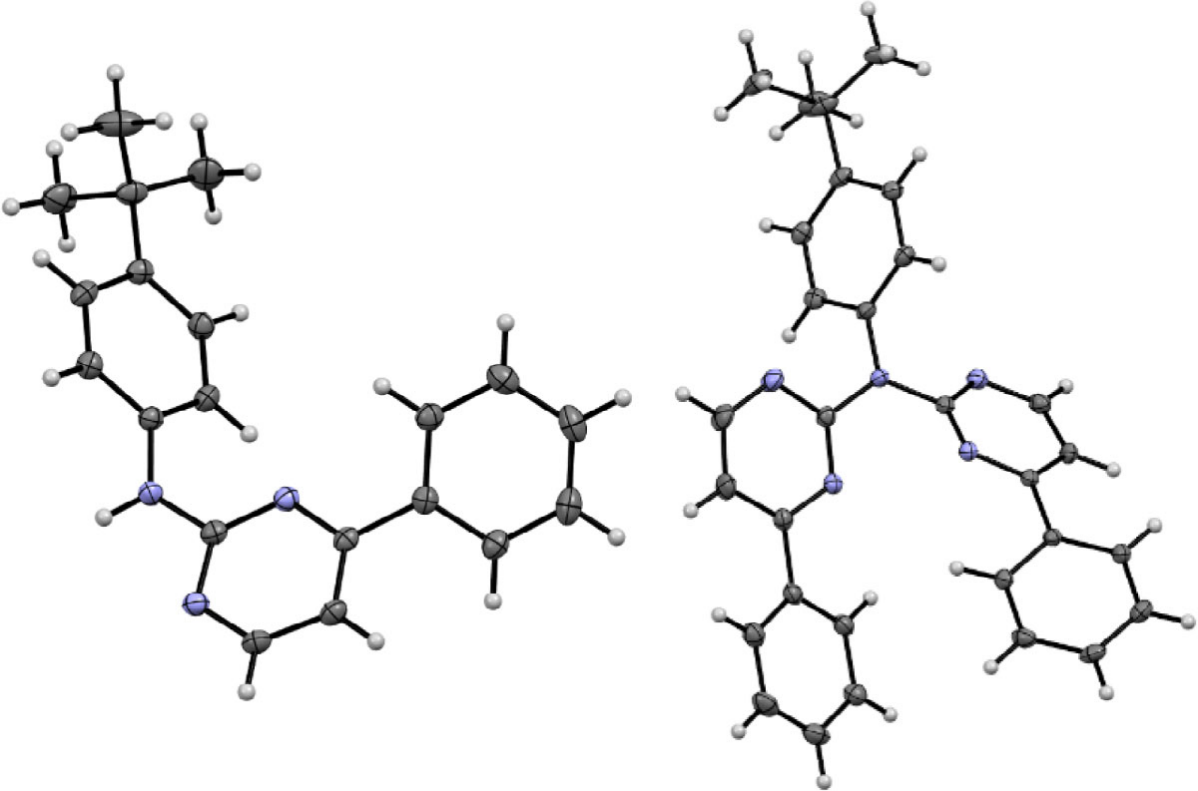
Molecular structure in the crystalline phase of **1** (left), and **LH_2_** (right). Displacement ellipsoids are shown at 50% probability.

**Figure 7:**
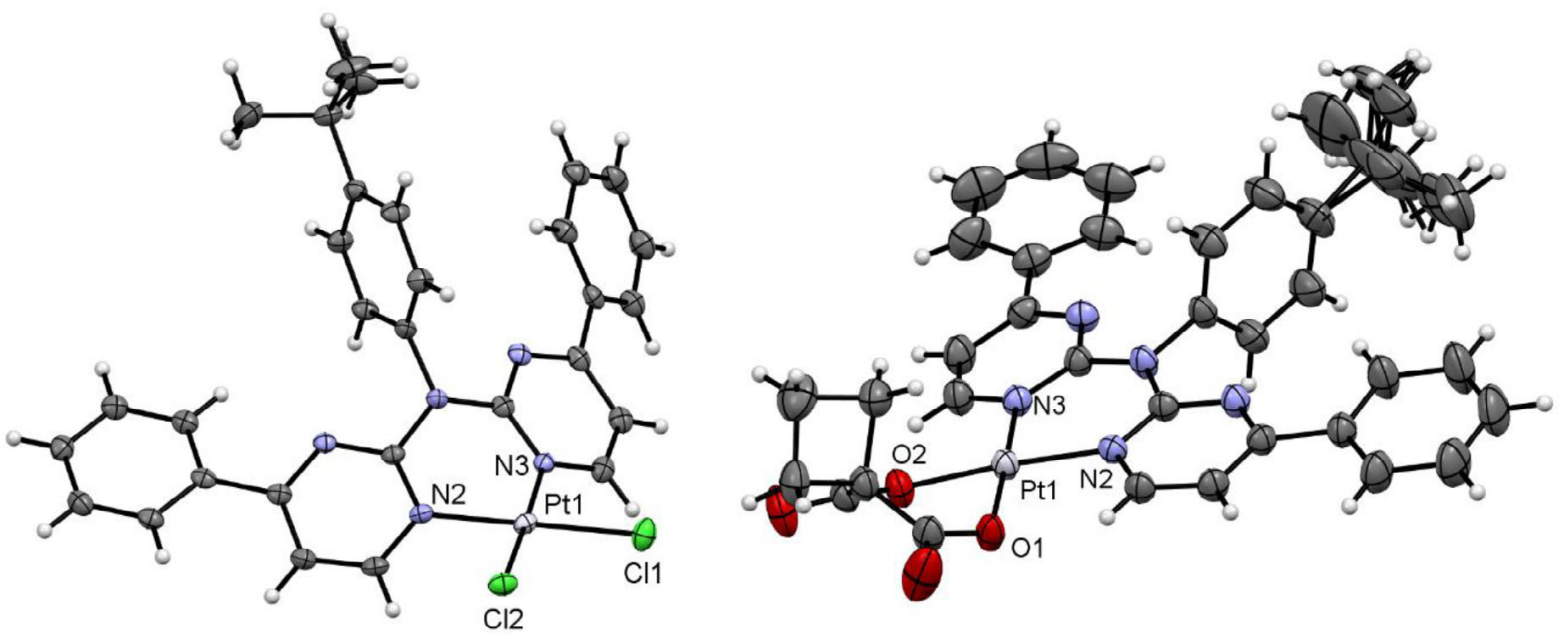
Molecular structure in the crystalline phase of [**PtLH_2_Cl_2_**] (left), and [**PtLH_2_cbda**] (right). Displacement ellipsoids are shown at 50% probability.

**Figure 8:**
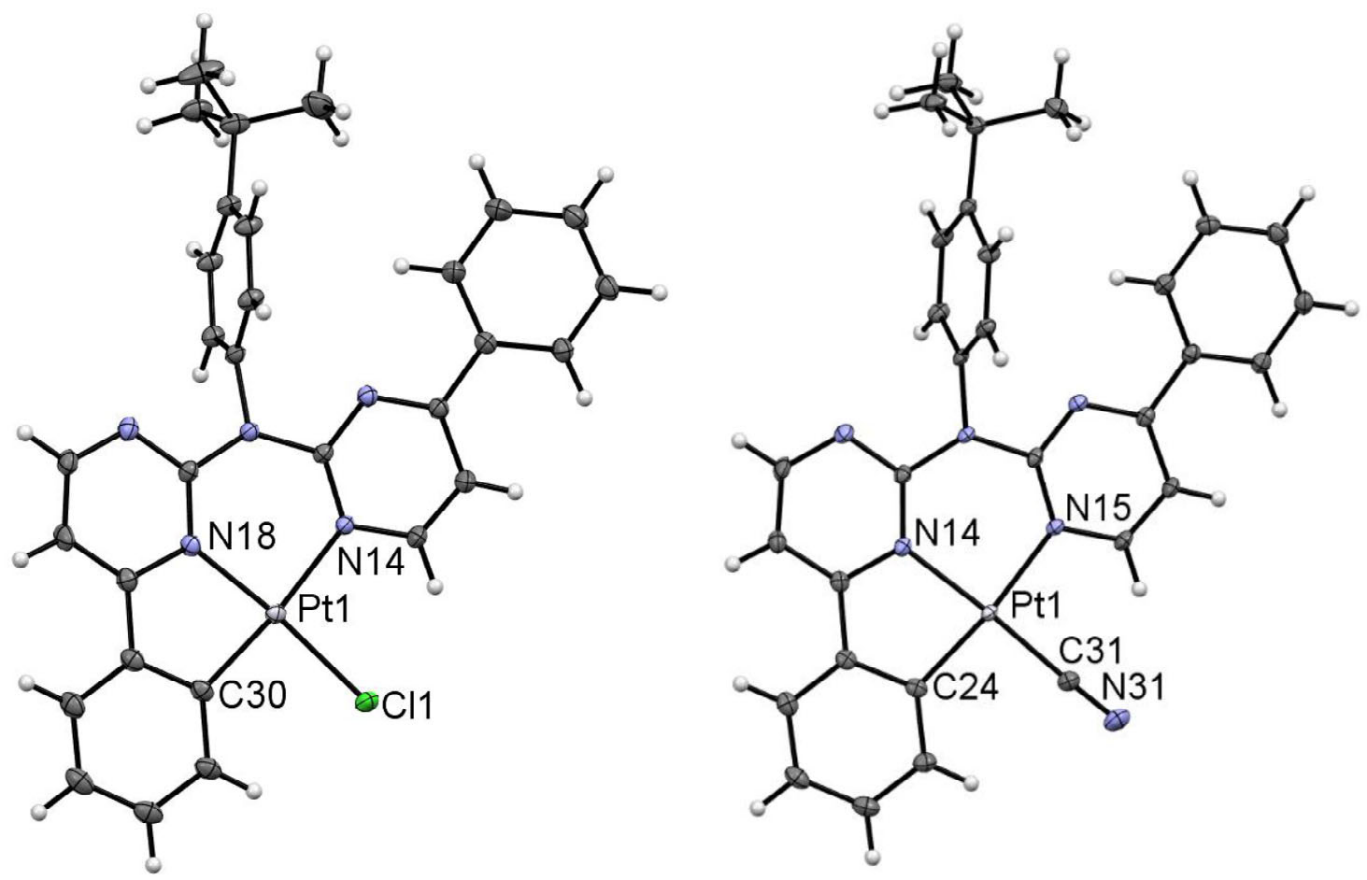
Molecular structure in the crystalline phase of [**PtLHCl**] (left, molecule A), and [**PtLHCN**] (right). Displacement ellipsoids are shown at 50% probability.

**Figure 9:**
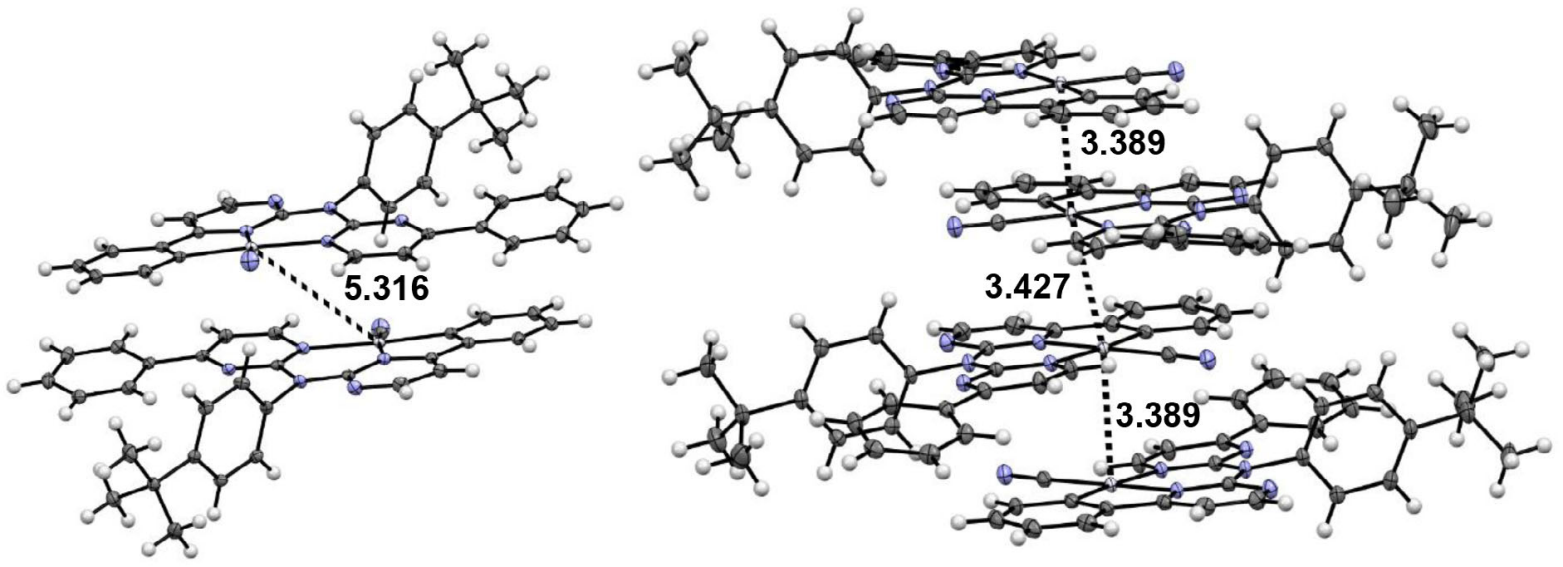
Depiction of the dimer formation in the single crystal of [**PtLHCN**] (left, yellow form; right, orange form) with the shortest Pt⋯Pt distances given in Å.

**Figure 10:**
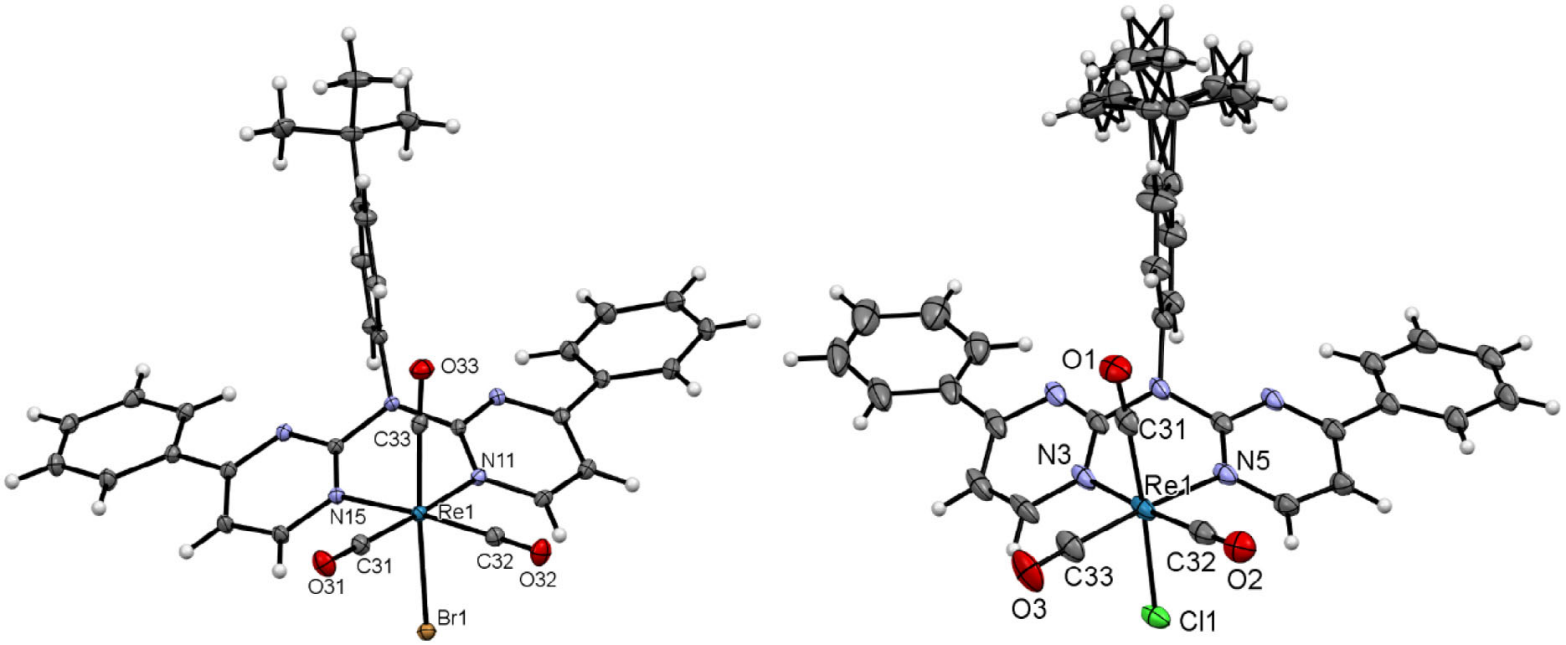
Molecular structure in the crystalline phase of [**ReLH_2_(CO)_3_Br**] (left), and [**ReLH_2_(CO)_3_Cl**] (right). Displacement ellipsoids are shown at 50% probability.

The molecular structure of **1** reveals that none of the aromatic rings are co‐planar to each other (Figure 6 left and Figure S64, Supporting Information). Two of these molecules form a dimer via a hydrogen bond, utilizing the amine proton and a pyrimidine‐based nitrogen atom. These dimers form chains through *π⋯π*/H interactions and the remaining 3D‐structure is mostly built up by H(*^t^* Bu/Ph)‐H/*π*(Ph) interactions (Figure S65, Supporting Information).

For **LH_2_**, the molecular structure in the single crystal (Figure S66, Supporting Information) displays two conformers of the molecule (**A** and **B**) and co‐crystalizes with one CH_2_Cl_2_ molecule (Figure S67, Supporting Information). For conformer **A** (Figure 6, right) both pyrimidine‐phenyl moieties point in the same direction, whereas for **B** these moieties are oriented in opposite directions. Additionally, **A** forms stacked dimers, but the rest of the 3D‐structure is built up solely via H‐*π*/N/Cl interactions (Figure S68, Supporting Information).

The single crystals from [**PtLH_2_Cl_2_**] (Figure 7, left) were obtained by slowly cooling a hot DMSO solution of the complex, resulting in an incorporation of the solvent in the crystal structure (Figure S69, Supporting Information). The molecular structure shows that the coordination plane around the Pt(II) center is completed with two chlorido ligands. To relieve steric strain, the aromatic plane of the **LH_2_** ligand is bent out of the coordination plane and the *^t^* Bu‐phenyl unit is perpendicular to the rest of the ligand. This structure is reminiscent of the literature‐known complex [Pt(2,2′‐dipyridylamine)Cl_2_] where the bond lengths and angles closely resemble the reported values.^[^ ^63^ ^]^ *π⋯π* interactions of the pyrimidine‐phenyl unit leads to dimers, which are visible in the elementary cell (Figures S69–S71, Supporting Information). Additional interactions of the aromatic system of these dimers (H‐*π*‐, *π⋯π*‐ and solvent interactions) lead to a layered structure. These 2D layers are connected through Cl⋯H⋯, H⋯H⋯ and H⋯O/S⋯interactions to form the 3D structure.

The molecular structure of [**PtCl_2_cbda**] (Figure 7 and Figure S72, Supporting Information) shows the coordination of the cbda^2−^ unit via two oxygen donors. The *^t^* Bu moiety is displayed over two positions. Compared with the chlorido precursor, the cbda co‐ligand is bent out of the coordination plane, but both phenylpyridine moieties from the **LH_2_**‐ligand are co‐planar to the rest of the coordination plane, differing from its literature‐known counterpart [Pt(2,2′‐dipyridylamine)cbda]^[^ ^63^ ^]^ and [**PtCl_2_LH_2_**]. The *^t^* Bu‐phenyl group is again orthogonal to the rest of the aromatic system to minimize steric repulsion. The crystals show incorporation of the solvent (two DCM units for each complex, one of them distorted over multiple positions). The molecules form dimers involving interactions of the two coordination planes, resulting in a Pt⋯Pt distance of 3.35 Å (Figure S73, Supporting Information). Because of the bent cyclobutyl unit, the Pt⋯Pt interactions are limited to these dimers. 2D layers are formed by H‐H/O interactions for the cbda ligands (Figure S74, Supporting Information), as well as *π⋯π* interactions between the phenyl moieties and the pyrimidine units (Figure S75, Supporting Information). The interaction of two of these layers are mostly built up from H⋯H and H⋯Cl interactions occurring from the *^t^* Bu group or the incorporated DCM solvent, thus leading to the observed 3D structure (Figure S76, Supporting Information).

[**PtLHCl**] forms a deep‐red solid or yellow crystals. X‐ray diffraction on these yellow crystals reveals the incorporation of two DCM molecules and the mono‐cyclometalation of one phenyl substituent (Figure 8, left). Additionally, because of different rotational orientation of the *^t^* Bu substituents, two different molecules (**A** and **B**) are displayed in the asymmetric unit (Figures S77 and S78, Supporting Information). Bond angles and lengths are in the expected range^[^ ^52^, ^61^, ^64^ ^]^ and comparable to the other herein reported complexes. As the cyclometallation results in a five‐membered metallocycle, this angle (i.e., 82.58(6)° for molecule **A**) deviates the most from the optimal 90° value. The complexes form the usual head‐to‐tail dimers (**AA** and **BB**) though *π‐π* interactions of the coordination planes, but Pt⋯Pt contacts are not observed (with interatomic distances above 3.7 Å, Figure S79, Supporting Information). These dimers interact between each other (**ABAB**) through Cl⋯H interactions of the chlorido co‐ligand and the non‐cyclometallated phenylpyridine unit (Figure S79, Supporting Information). The dimers are capped in the z‐direction over H⋯*π*/H/Cl interactions between the coordination plane and the DCM solvent and the *^t^* Bu‐phenyl group from a neighboring dimer (Figure S80, Supporting Information). The remaining 3D structure is built up mostly from interactions between the DCM molecule and H‐H interactions with the *^t^* Bu‐phenyl group (Figure S81, Supporting Information).

For [**PtLHCN**], we also observe different solid phases (a reddish solid and also two different crystalline phases). Yellow crystals were obtained by slowly diffusing *n*‐hexane into a EtOAc solution of the complex, whereas a solution of the complex in DCM was used for the orange‐reddish crystals. Both molecular structures in the single crystal demonstrate the exchange of the chlorido unit by the cyanido co‐ligand (Figure 8, right). They both display similar bond lengths and angles.

The yellow phase ([**PtLHCN**](yellow)) shows no incorporated solvent molecules. The complexes form characteristic head‐to‐tail dimers, in this case involving the whole aromatic system of the coordination plane (Figure 9), with a Pt⋯Pt‐distance above 5 Å. Utilizing further *π⋯π* interactions in addition to H‐*π* contacts between the *^t^* Bu‐phenyl units and the coordination plane, a 2D layer is formed (Figure S83, Supporting Information). To form the 3D structure, the layers interact through H─N bonds involving the cyanido co‐ligands, as well as H⋯H interactions (Figures S83 and S85, Supporting Information). Changing from one layer to another, the orientation of the coordination plane rotates by about 90°.

For the orange phase ([**PtLHCN**](orange)), two molecules in the asymmetric unit are found and the structure is solved in a *P* symmetry. The structure was solved as *C*2/*c* with one molecule in the asymmetric unit; due to superlattice reflections, this results in relatively large displacement ellipsoids for the *^t^* Bu moiety. Again, the distinction between these molecules involves a slightly different position of the *^t^* Bu group. This orange crystalline phase incorporates one solvent molecule (DCM) in its structure. Head‐to‐tail dimers are formed via *π⋯π* and Pt⋯Pt interactions, leading to a Pt⋯Pt‐distance of 3.369 Å. The Pt⋯Pt distance to the next dimer is 3.427 Å and with slightly different orientation of the coordination planes facing each other. This leads to the formation of 1D chains with Pt⋯Pt contacts (Figure S88, Supporting Information) that are further stabilized by H⋯H/Cl/*π* interactions of the non‐cyclometallated phenyl substituents, the DCM solvent and the *^t^* Bu‐phenyl unit (Figures S88 and S89, Supporting Information).

Interestingly, for [**PtLHCl**], the incorporated DCM molecule seems to prevent Pt⋯Pt interactions, whereas inclusion of the solvent leads to formation of such interactions for [**PtLHCN**] (Figure 4).

Single crystals of [**ReLH_2_(CO)_3_Br**] (Figure 10, left) were obtained by slowly evaporating a CHCl_3_ solution of the complex, resulting in the co‐crystallization of CHCl_3_. The molecular structure confirms the distorted pseudo‐octahedral coordination with three facial carbonyl ligands, the bidentate‐bound chromophore (where the aromatic plane is bent out of the coordination plane, like for their 2,2′‐dipyridylamine‐counterparts^[^ ^65^, ^66^, ^67^, ^68^, ^69^, ^70^ ^]^) and the bromido ligand. The largest deviation from the optimal 90° value corresponds to the N─Re─N angle (81.66(4)°) involving the bidentate unit. H─O Interactions between the carbonyl ligand and an adjacent chromophoric chelator leads to the formation of 1D chains (Figure S91, Supporting Information). Due to the center of inversion, a counter chain is formed that interacts via Br‐H interactions with another chain (Figure S92, Supporting Information). The H⋯Br interactions and the additional *π⋯π*, H─O bridges (as well as interactions including the CHCl_3_ molecule) also contribute to stabilize the 3D structure (Figures S93 and S94, Supporting Information).

The molecular structure of [**ReLH_2_(CO)_3_Cl**] (Figure 10, right) proves the coordination of a chlorido ligand. The crystal structure also shows co‐crystallized solvent molecules, in this case DCM and cyclohexane. Since these molecules occupy the same position in the crystal structure (half of a DCM and half of a cyclohexane unit are incorporated per coordination‐chemical entity). Additionally, the *^t^* Bu group is displayed over two positions. Even with the change of the incorporated solvent entities, the rest of the structural features (bond lengths and angles, pseudo‐octahedral coordination and 3D structure) remain similar to the structure of complex [**ReLH_2_(CO)_3_Br**].

### 2.4 Photophysics

The photophysical properties of the complexes are summarized in Table 1, the UV–vis absorption spectra at 298 K and the photoluminescence spectra (298 and 77 K) of selected compounds are shown in Figure 11 (for the complete set of spectra, time‐resolved photoluminescence decay plots as well as for the uncertainties and the (multi‐)exponential lifetime fits see the Table S12 and Figures S96–S125, Supporting Information).

**Figure 11:**
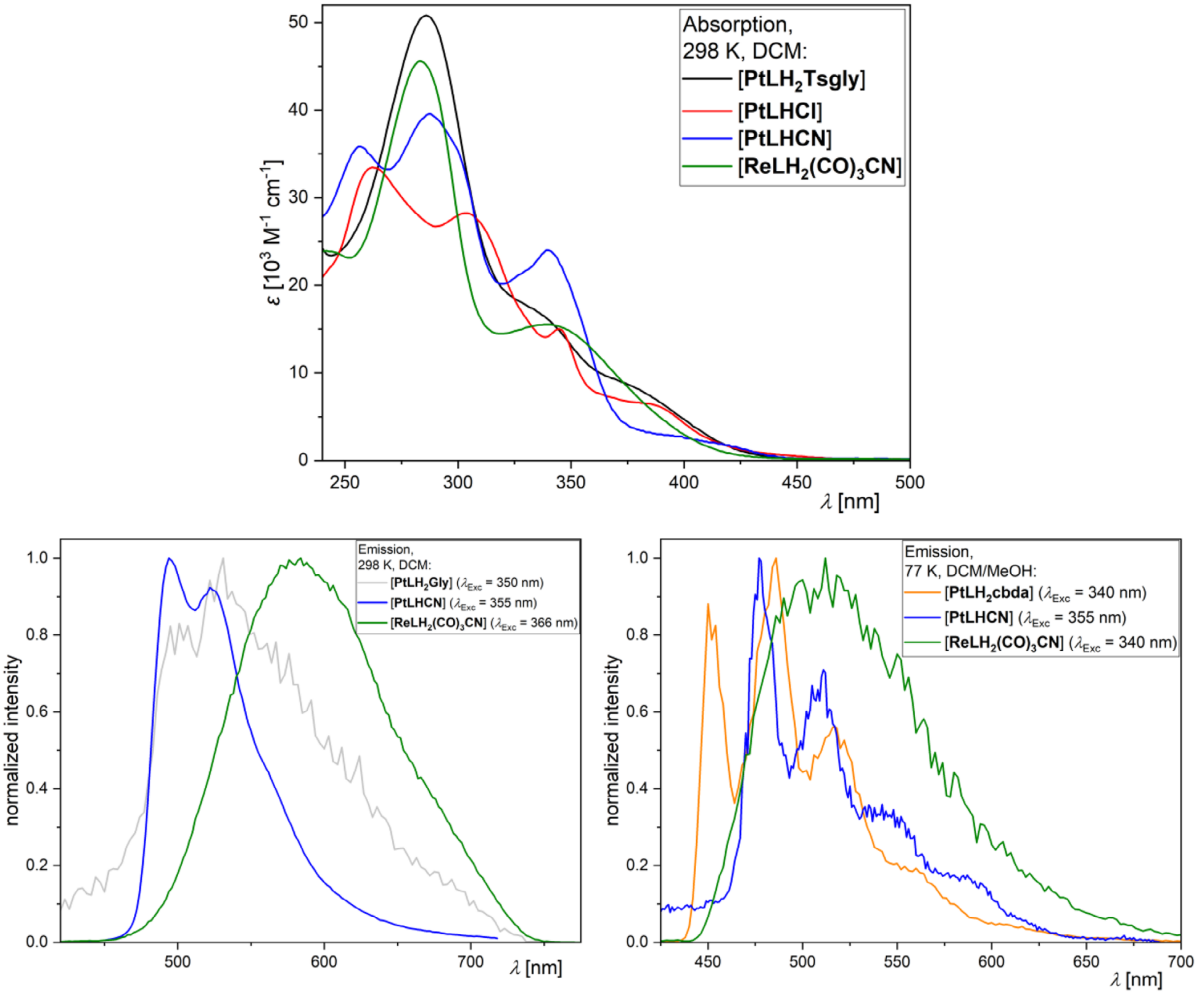
UV–vis absorption spectra in liquid DCM at 298 K (top), steady‐state photoluminescence emission spectra (bottom) in liquid DCM at 298 K (left) and in a frozen glassy matrix of DCM/MeOH (V:V = 1:1) at 77 K (right) of [**PtLH_2_Tsgly**] (black), [**PtLH_2_Gly**] (gray), [**PtLH_2_cbda**] (orange), [**PtLHCl**] (red), [**PtLHCN**] (blue) and [**ReLH_2_(CO)_3_CN**] (green). Validity range of the absorption spectra: *c* = 5 × 10^−5^–2 × 10^−6^ m in liquid DCM at 298 K. All solutions for the emission spectra were optically diluted (*A* < 0.1) and normalized to the highest intensity.

The assignment of the bands in the UV–vis absorption spectra was done by comparison with previously reported complexes.^[^ ^27^, ^45^, ^46^, ^52^, ^56^, ^61^, ^62^, ^64^, ^71^, ^72^ ^]^ The high‐energy bands with strong molar absorption coefficients below 325 nm correspond to transitions into states that can be described as ^1^π⋯π* configurations (i.e., with ligand‐centered character, ^1^LC). The low‐energy bands around 350 nm and above can generally be assigned to transitions into states with mixed ligand‐centered/metal‐to‐ligand charge‐transfer character (i.e., ^1^LC/^1^ILCT/^1^MLCT). For clarity, the discussion of the complexes is structured into three groups: N*N‐coordinated Pt(II) complexes ([**PtLH_2_L_2_**]), C^N*N‐chelated Pt(II) compounds ([**PtLHX**]) and N*N‐coordinated Re(I) species ([**ReLH_2_(CO)_3_X**]).

N*N‐Pt(II): The absorption profiles of all four [**PtLH_2_L_2_**] complexes is nearly identical, dominated by LC states. A prominent ^1^LC‐related band is observed at *λ*_abs_ = 287 nm, along with another LC‐attributed feature at around 330 nm. At 375 nm, a CT‐related band becomes increasingly prominent in the order cbda < Cl_2_ ≈ Gly < Tsgly, suggesting the strongest CT character for the latter (Figure S96, Supporting Information). The missing C^−^‐donor results in a weaker LFS and a very weak perturbation of the excited states by the metal while leading to an emission that is dominated by the chromophoric ligand. The combination of the N*N‐coordinated main ligand with two chlorido units (which act as *σ*‐ and *π*‐donors) leads to the weakest LFS in the series and renders the excited state of [**PtLH_2_Cl_2_**] quenched by thermally‐accessible yet non‐radiative decay pathways (while leaving the complex non‐emissive even at 77 K). [**PtLH_2_Gly**] is the only complex of the four to show weak yet unstructured emission at RT (Figure 11). At 77 K in a frozen glassy matrix, the three complexes are emissive and show a very long excited state lifetime (>86 µs). Overall, [**PtLH_2_Gly**] and [**PtLH_2_cbda**] show nearly the same photophysical properties, both exhibiting a high‐energy emission band at 450 nm, maxima at ∼485 nm, as well as lifetimes of ∼120 µs and strong yet slightly different vibrational progressions. [**PtLH_2_Tsgly**], having the highest amount of CT‐character, shows the broadest emission spectrum, which is slightly red‐shifted (468 and 496 nm) in combination with the shortest excited state lifetime (86.3 µs).

C^N*N‐Pt(II): The UV–vis absorption spectra (Figure 11) show bands resulting from the now non‐symmetric aromatic system upon cyclometallation, as well as pronounced CT‐related bands for [**PtLHCl**] and [**PtLHCN**] at 387 and 422 nm, respectively. Both complexes show green emission with weak vibrational progression (already at 298 K in liquid DCM solutions). The emission of [**PtLHCl**] peaks at 509 nm and appears red‐shifted compared to the thiazole‐based reference complex [**PtCl(L_ref_**)] (*λ*_Em_ = 494 nm). The shift in emission wavelength could stem from a destabilization of the LUMO (localized presumably at the pyrimidine unit^[^ ^27^, ^56^, ^64^ ^]^) upon switching from the pyridine to a pyrimidine ring. Similar to the reference compound,^[^ ^61^ ^]^ the lifetime is relatively short (in the ns range) due to the fast non‐radiative deactivation of the excited state. This renders the luminescence insensitive to dissolved ^3^O_2_.^[^ ^52^, ^61^, ^71^ ^]^ Upon cooling to 77 K in a frozen glassy matrix, the emission maximum blue‐shifts to 484 nm and exhibits an enhanced vibrational progression, typically associated with the loss of solvent stabilization and the inherent loss of metal participation in the excited state. The lifetime increases to $\tau_{\mathrm{av}}$ = 8.04 µs. The ligand exchange to yield [**PtLHCN**] effectively enhances the LFS, leading to strong oxygen‐dependent emission. In argon‐purged DCM solutions, the lifetime increases to 10.34 µs with a quantum yield of Φ_L_ = 0.55. These values are comparable to the phenyl‐acetylido complex reported by Huo and co‐workers^[^ ^56^ ^]^ and surpass those of our previously reported thiazole‐based complexes.^[^ ^52^, ^61^ ^]^ The lifetime is also much shorter compared to the thiazole‐based complexes, indicating increased metal perturbation of the excited states and resulting in higher deactivation rate constants.^[^ ^52^, ^61^, ^71^, ^72^ ^]^ Compared to the chlorido complex, the emission is blue‐shifted in both liquid solution and at 77 K. Additionally, the lifetime at 77 K is more than twice as long (19.26 µs), suggesting that the *π*‐backbonding of the cyanido ligand not only enhances the LFS but it also reduces the metal perturbation of the excited states, leading to a stronger LC character of the excited state.

N*N‐Re(I): The UV–vis absorption spectra of the [**ReLH_2_(CO)_3_X**] complexes show two bands: one corresponding to transitions into excited states with LC character around 285 nm, and another one that is likely related to a LLCT state with sizeable MLCT character admixed at ∼345 nm. All complexes are emissive at RT with weak unstructured MLCT state‐related emission bands between 584 – 600 nm and short lifetimes. At 77 K in frozen glassy matrices, the emission undergoes a blue‐shift in combination with prolonged lifetime.^[^ ^62^, ^73^ ^]^ The latter may occur due to the reduction of the MLCT character of the emissive triplet state upon the loss of solvent stabilization. Upon ligand exchange to a cyanido unit, an overall increase in the lifetime and a blue‐shift of the emission maxima are observed, suggesting, a decrease of metal participation in the excited state. No pronounced vibronic progression is observed, though. The longest lifetime of* τ*_av_ = 49.5 µs for [**ReLH_2_(CO)_3_CN**] may result from higher LC character with a lower *k*_R_ along with a higher LFS and lower *k*_NR_ (if compared with the analogous Cl/Br complexes). However, due to instrumental limitations and insufficient solubility, it was not possible to determine the photoluminescence quantum yields at 77 K, thus impairing further analysis.

### 2.5 Aggregation studies

#### 2.5.1 Solid‐state measurements

**Figure 12:**
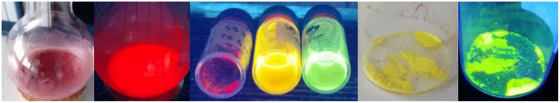
Images of the red (round flask, left) and yellow (powders, right) solids of [**PtLHCl**] under ambient condition and UV irradiation (*λ*_Exc_ = 366 nm). Pure red solid of [**PtLHCN**] obtained from DCM (center‐left), suspended in EtOAc (yellow suspension, center), and in an EtOAc/MeOH‐mixture (center‐right) for 10 min observed under UV irradiation (*λ*_Exc_ = 366 nm).

After observing different morphologies in the solids of the [**PtLHX**] family (Figure 12), we explored the relationship between phases and their photophysical properties. For both complexes (**X** = Cl or CN), orange‐reddish and yellow solids were obtained; for all the yellowish phases as well as for the orange‐reddish form of [**PtLHCN**], molecular structures were attainable by X‐ray diffractometry. The phases obtained depend on the solvent, evaporation process, and time. Rapid evaporation of DCM at higher temperatures results in reddish forms, whereas a slow evaporation of the same DCM solution can yield a yellow phase (Figure 12). If the orange‐reddish form of [**PtLHCN**] is suspended in EtOAc, it gradually transforms into a yellow solid, with the addition of MeOH accelerating the transformation (Figure 12).

**Figure 13:**
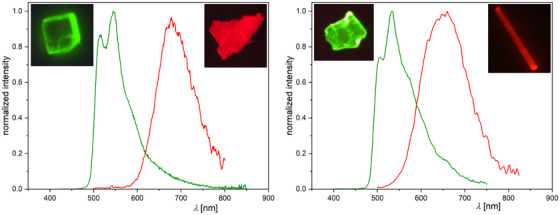
Photoluminescence spectra of the complexes [**PtLHCl**] (left) and [**PtLHCN**] (right) emitting as monomeric (green) or aggregated (red) species in crystalline phases at 298 K (normalized to the highest intensity; measured using a photoluminescence spectrometer coupled to the confocal microscope). Luminescence micrographs of the crystals are shown as insets (*λ*_exc_ = 375 ± 20 nm).

For all four (micro‐)crystalline phases, time‐resolved photoluminescence micro(spectro)scopy measurements were performed using a confocal microscope coupled to a photoluminescence spectrometer via an optical fiber. The emission spectra are shown in Figure 13; the complete set of spectra, time‐resolved photoluminescence decay plots, and lifetime maps measured by photoluminescence lifetime imaging micro(spectro)scopy (PLIM), along with uncertainties and (multi‐)exponential lifetime fits, can be found in Table S13 and Figures S126–S133, Supporting Information.

Both complexes, namely [**PtLHCl**] and [**PtLHCN**], exhibit very similar behavior in their crystalline forms (Figure 13). The yellow phases show a greenish emission, peaking at *λ*_Em_ = 544 nm and *λ*_Em_ = 534 nm, respectively. A vibronic progression is visible, but compared to the spectra in solution, the second band appears more pronounced. Such behavior has been attributed to the possible restriction of vibrational modes in the crystalline environment, as reported in our earlier work on solid‐state emission.^[^ ^61^ ^]^ As in solution or glassy matrix, the lifetime of the cyanido complex ($\tau_{\mathrm{av}}$ = 3.51 µs) is significantly longer than that of [**PtLHCl**] ($\tau_{\mathrm{av}}$ = 0.972 µs), most likely due to the higher LC character of the excited state. In the orange‐reddish phases, a shift of emission becomes apparent, with broad maxima at *λ*_Em_ = 678 nm (**X** = Cl) and *λ*_Em_ = 660 nm (**X** = CN) that lack vibrational progression. Additionally, the $\tau_{\mathrm{av}}$ values decrease to 184 ns and 154 ns, respectively. This emission arises from a ^3^MMLCT state of the aggregated forms, which is in perfect agreement with the 1D‐Pt⋯Pt interactions visible in the crystal structure (Figure 9). These findings suggest that the differences between these phases result from dissimilar intermolecular interactions of the complexes in the solid state.

#### 2.5.2 Concentration‐dependent emission studies in liquid solutions

**Figure 14:**
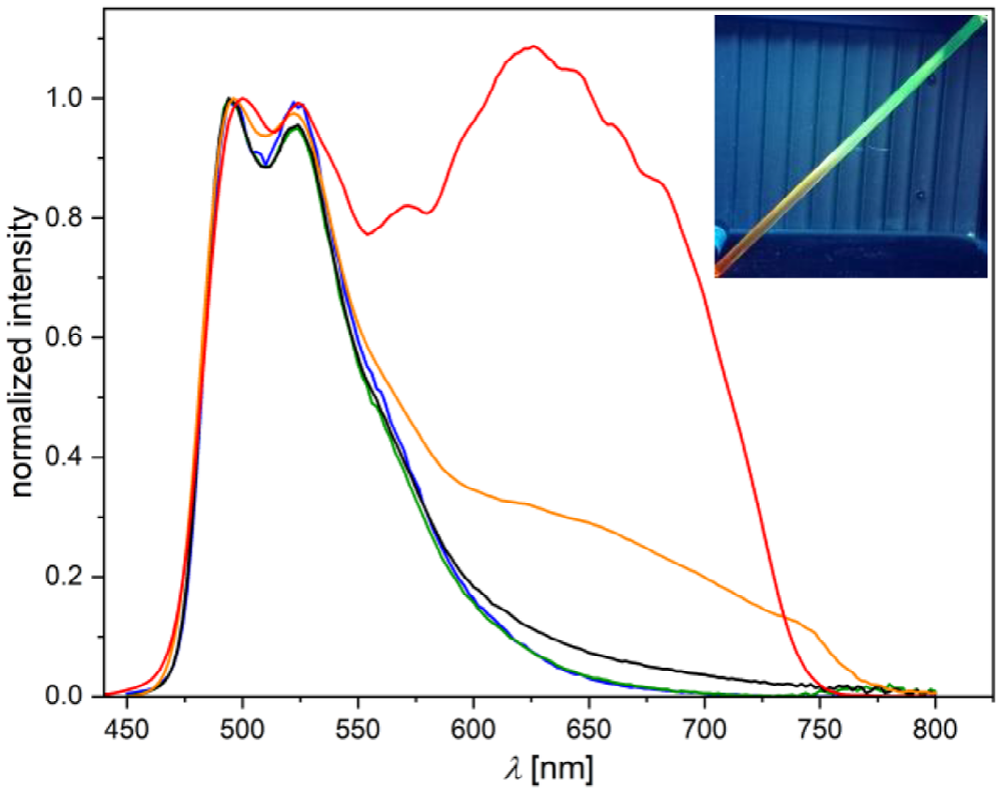
Concentration‐dependent photoluminescence emission spectra (*λ*_Exc_ = 350 nm) in liquid Ar‐purged DCM at 298 K [**PtLHCN**] within the concentration range of *c* = 1·10^−7^ (blue), 1·10^−6^ (green), 1·10^−5^ (black), 1·10^−4^ (orange), 1·10^−3^ (red). Normalized at the highest energy maximum. Inset: Solid [**PtLHCN**] layered with DCM in an NMR tube yielding a concentration gradient at RT under UV irradiation (*λ*_Exc_ = 366 nm).

We investigated the concentration dependent emission of [**PtLHCN**] (*c* = 10^−7^–10^−3^ m) in DCM at 298 K using steady‐state and time‐resolved photoluminescence spectroscopy. At *c* = 10^−5^ m, the rise of a new emission band at 650 nm is observed, which intensifies at higher concentrations (Figure 14). These unstructured emission bands are typical for Pt‐based excimers with MMLCT character.^[^ ^24^, ^27^, ^57^, ^75^ ^]^ Unlike our previously reported complex with ligand‐directed aggregation,^[^ ^27^ ^]^ here the monomeric emission remains clearly visible, even at *c* = 10^−3^ m. An increasing red shift is also observed for the excitation maxima (Figure S134, Supporting Information), due to the appearance of MMLCT‐state related transitions (along with an increasing inner filter effect in the UV at higher concentrations).

When monitoring the monomeric emission at 494 nm, a steady decrease in the $\tau_{\mathrm{av}}$ is noted for the Ar‐purged solutions. The excimeric photoluminescence at 600 nm exactly mirrors the monomeric lifetimes (including the monoexponential decay at lower concentrations, with an additional rise‐time appearing above *c* = 10^−5^ m). This suggests that a kinetic model for excimer formation can be applied, where $\tau_{\mathrm{av}}$ is linearly dependent on the concentration *c*, as the excited state lifetime of the excimer is significantly shorter than the one corresponding to the monomer (Equation 1):

(1) $$\;\frac{1}{\tau_{\mathrm{av}}}=k_{\mathrm{Ex}}\cdot c+\frac{1}{\tau_{0}}$$

where *k*_Ex_ is the excimer formation rate constant, and $\tau_{0}$ is the exited state lifetime of the monomer at infinite dilution. In this model, the mirroring of the decays results from the shorter excimer versus the monomer lifetime, with the risetime of around 300 ns corresponding to the decay of the excimer (as it was previously explained and evaluated in our earlier report).^[^ ^27^ ^]^ This is also discussed in the established literature (see, e.g., “Molecular Fluorescence” by Bernard Valeur).^[^ ^76^ ^]^ Utilizing this model, we can extrapolate $\tau_{0}$ = 14.34 µs (the excited state lifetime at an infinite dilution, which matches well with the lifetime at a concentration of 10^−7^ m, i.e., $\tau_{\mathrm{av}}$ = 14.38 µs), as well as an excimer formation rate constant (*k*_Ex_ = 5.6·10^9^ m ^−1^s^−1^). Higher concentrations are excluded from the fit (Figures S135 and S136, Supporting Information) due to potential ground state aggregation, which affects the linearity of the 1/τ versus *c* plot.

#### 2.5.3 Concentration‐ and temperature‐dependent NMR spectroscopic studies in solution

The investigation of the aggregation through photophysical measurements was limited to [**PtLHCN**] due to the short lifetime and low Φ_L_ of the chlorido complex [**PtLHCl**]. Since solubility and degree of aggregation can play a crucial role in practical applications, we further studied the self‐association behavior of Pt(II) complexes [**PtLHCl**] and [**PtLHCN**]. Common methods for investigating self‐assembly in solution include temperature‐ or solvent‐dependent UV–vis absorption spectroscopy.^[^ ^77^, ^78^ ^]^ However, within suitable concentration ranges for these experiments, [**PtLHCl**] and [**PtLHCN**] did not show significant self‐assembly in the chosen solvents before precipitation occurred. Following previously reported experimental protocols, we employed concentration‐ and temperature‐dependent ^1^H‐NMR spectroscopy as a quantitative tool for this study.^[^ ^75^, ^79^ ^]^

**Figure 15:**
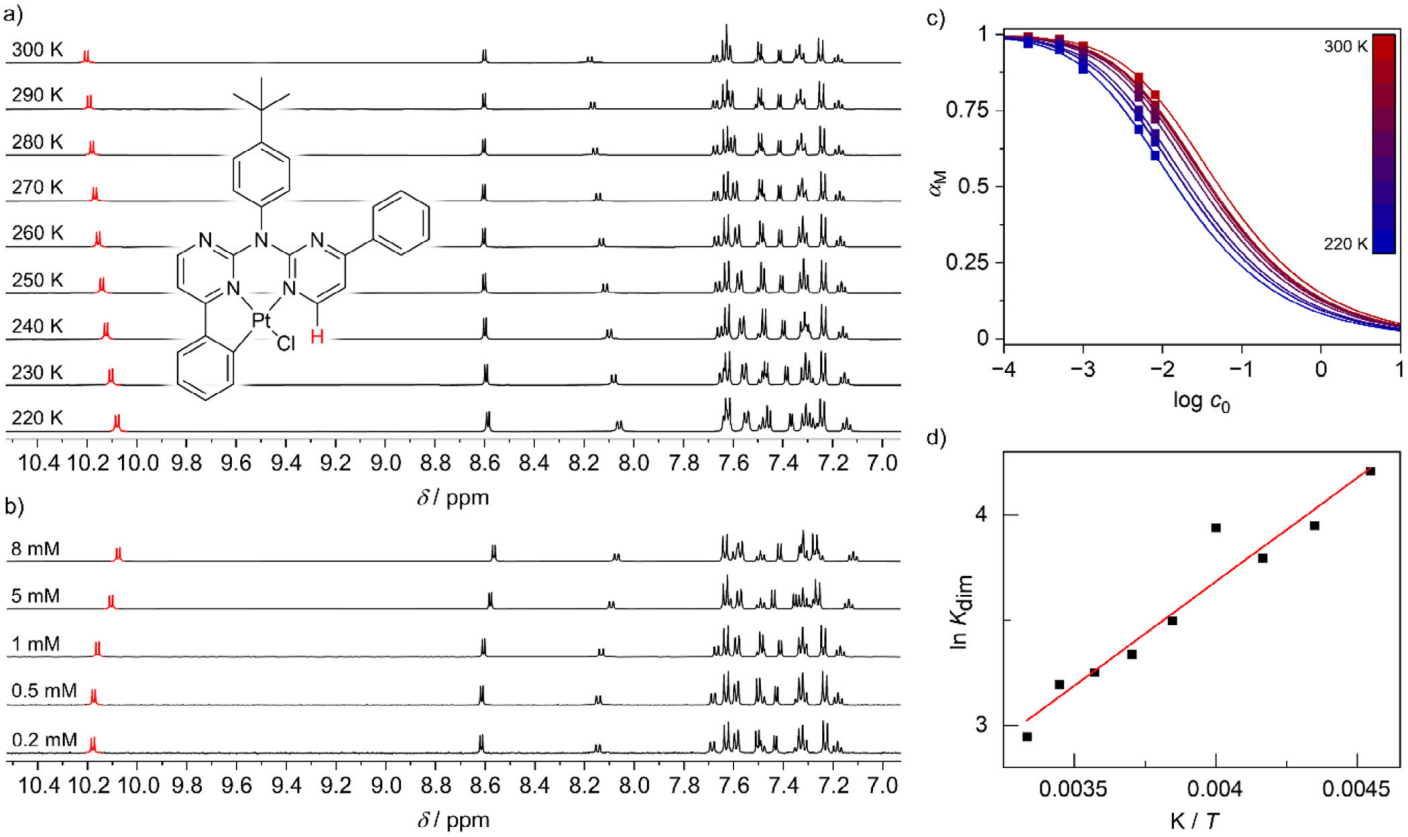
a) VT‐^1^H‐NMR experiment (400 MHz, CD_2_Cl_2_) of a 1 mm solution of [**PtCl(LH)**]. (b) ^1^H‐NMR spectra (400 MHz, CD_2_Cl_2_) of solutions of [**PtCl(LH)**] at different concentrations *c*_0_. (c) *α*_M_ (*c*_0_) plots of [**PtCl(LH)**] at 220 to 300 K (squares) and non‐linear fit based on Equation (2) (curves). (d) ln *K*_dim_ (1/*T*) plot of [**PtCl(LH)**] and linear fit following van't Hoff's relation (3).

For this purpose, solutions of both compounds [**PtLHCl**] and [**PtLHCN**] in DCM‐*d*_2_ were prepared in a concentration range between 0.2 and 15 mm (0.2–15·10^−3^ m). However, in the samples with concentrations of 10 and 15 mm, the complexes precipitated rapidly. Therefore, temperature‐dependent ^1^H‐NMR spectra of the remaining solutions were recorded (with concentrations between 0.2 and 8 mm, see Figures 15a and S156–S164, Supporting Information). The H‐atom in *α*‐position to one of the N─Pt‐bonds (highlighted red in Figure 15a) shows a significant high‐field shift with decreasing temperature in all conducted experiments. Consequently, this resonance signal was used to quantify the self‐association process. However, as the change in the chemical shift is not accompanied by spectral broadening, the formation of larger aggregates can be excluded in the observed temperature and concentration ranges. A similar behavior is observed with increasing concentration when comparing spectra at constant temperature (highlighted in red within Figure 15b). This shift is likely due to shielding of these H‐atoms by the aromatic units of adjacent molecules, caused by pronounced aromatic interactions. The lack of spectral broadening in the observed temperature and concentration range indicates initial stages of the aggregation process, most likely a dimerization event. As already described in a previously published report,^[^ ^75^ ^]^ the self‐association equilibrium is much faster than the nuclear spin decay in ^1^H‐NMR. Therefore, each composition of monomers and dimers shows only a single resonance signal, with the chemical shift correlated to the dimerization constant *K*_dim_. Würthner and co‐workers previously reported a model correlating optical absorbance with the dimerization constant of merocyanine dyes.^[^ ^80^ ^]^ Adapting the model to our NMR studies leads to the following relation (Equation 2):

(2) $$\delta \left(c_{0}\right)=\frac{\sqrt{8\;K_{\dim}c_{0}+1}-1}{4\;K_{\dim}c_{0}}\;\left(\delta_{M}-\delta_{D}\right)+\delta_{D}$$

where *δ*_M_ represents the chemical shift of the monomeric species, and *δ*_D_ represents the chemical shift of the dimer, providing a reference for the measured *δ* values as a function of the total concentration *c*_0_. Nonlinear regression analysis of each set of *δ*(*c*_0_) values based on Equation (2) gives *K*_dim_, *δ*_M_, and *δ*_D_ (Figures S165–S173 and Tables S15 and S16, Supporting Information). Interestingly, [**PtLHCN**] generally shows lower dimerization constants compared to [**PtLHCl**], beyond the respective uncertainties, indicating a weaker self‐association for [**PtLHCN**]. However, all *K*_dim_ values lie between ∼4–67 m ^−1^, suggesting that the general self‐association tendency of both compounds is comparable to other reported Pt(II) complexes.^[^ ^75^, ^81^, ^82^ ^]^ Further, the fraction of monomer *α*_M_ can be calculated from the chemical shifts *δ*, *δ*_M_, and *δ*_D_ (Equation 3):^[^ ^80^ ^]^

(3) $$\;\alpha_{M}=\frac{\delta -\delta_{D}}{\delta_{M}-\delta_{D}}$$

**Figure 16:**
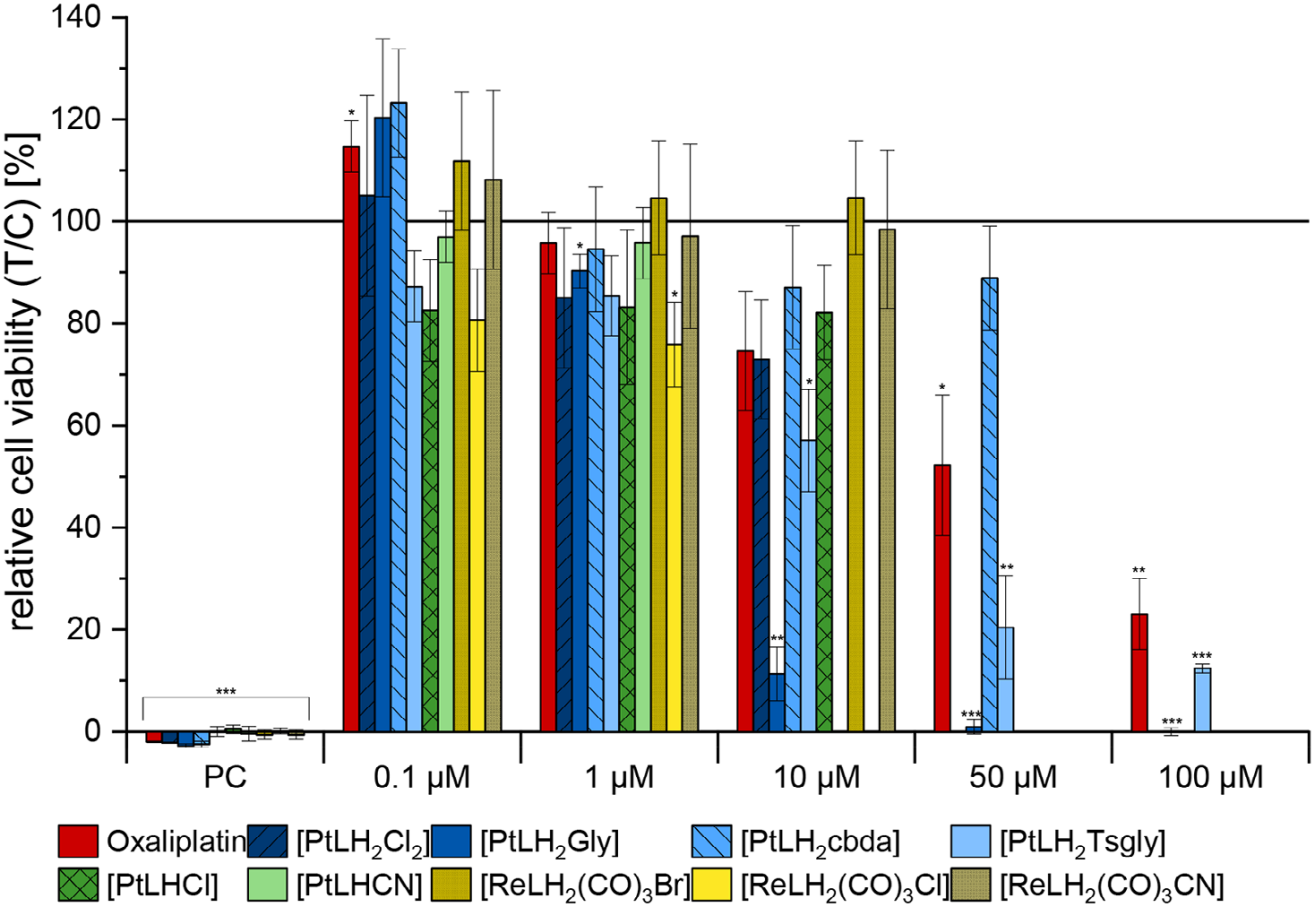
Relative viability of HepG2 cells after 24 h of incubation with the tested compounds. 1% DMSO and 0.01% saponin were used as a negative and positive control, respectively. Student's one‐sample *t*‐test relative to 100% was used for statistical evaluation: *P = 95.0%, **P = 99.0%, ***P = 99.9%.

Plotting the *α*_M_ values calculated from measured data points (Figure 15c: squares) and fits following Equation (2) (Figure 15c: lines) versus log *c*_0_ shows a sigmoidal progression upon dimer formation. While the 0.2 mm solutions of both complexes exhibit predominantly the monomeric state (*α*_M_ ∼ 0.97–1.00), *α*_M_ does not fall below 0.60 and 0.64 for the 8 mm solutions of [**PtLHCl**] and [**PtLHCN**], respectively (Tables S17 and S18, Supporting Information). Notably, [**PtLHCN**] bears higher monomer fractions at high temperatures and high concentrations (Figure S174, Supporting Information).

The provided *K*_dim_ values further allow the calculation of thermodynamic parameters for the dimerization using van't Hoff's relation (Equation 4):^[^ ^79^ ^]^

(4) $$\ln K_{\dim}=-\frac{\Delta H}{R\cdot T}+\frac{\Delta S}{R}$$

Plotting ln *K*_dim_ versus 1/*T* yields a linear relationship, with the slope and intercept proportional to Δ*H* and Δ*S*, respectively (Figure 15d and Figure S175, Supporting Information). Linear fitting yields Δ*H* values of −8.2 ± 0.8 kJ mol^−1^ for [**PtLHCl**] and −15.6 ± 0.9 kJ mol^−1^ for [**PtLHCN**]. Additionally, relatively low Δ*S* values (−2 ± 3 J mol^−1^ K^−1^ for [**PtLHCl**] and −37 ± 4 J mol^−1^ K^−1^ for [**PtLHCN**]) were obtained, the latter of which is consistent with values reported for structurally related Pt(II) complexes.^75^ This is likely due to the restricted rotation of the terminal phenyl and *p*‐*tert*‐butyl‐phenyl groups in [**PtLHCN**] upon aggregation. The high uncertainties in the calculated values for [**PtLHCl**] can be explained by the strong tendency to precipitate before a high fraction of monomer could undergo dimerization. On the other hand, [**PtLHCN**] exhibits a stronger drop in monomer fraction upon cooling of higher concentrated samples, induced by a lower dimerization enthalpy. This can be interpreted as a better stabilization of dimers of [**PtLHCN**] compared to [**PtLHCl**], minimizing the dipole moment and delaying the formation of larger aggregated structures. The latter ultimately leads to precipitation, as the lack of solubilizing moieties (such as linear alkyl chains) prevents dissolution. In summary, [**PtLHCl**] exhibits a stronger tendency to precipitate, whereas [**PtLHCN**] tends to form well stabilized dimers and precipitates later upon cooling of concentrated solutions.

### 2.6 Cytotoxicity

Due to structural similarities with anticancer drugs, the Pt(II) and Re(I) complexes were evaluated with regards to their cytotoxicity against HepG2 cells.^[^ ^37^, ^83^, ^84^ ^]^ As the liver plays an important role in drug metabolism, the well‐established human liver carcinoma cell line was chosen as a suitable *in vitro* model.^[^ ^83^ ^]^ Cytotoxicity was assessed using the resazurin assay after 24 h of incubation with the Pt(II) and Re(I) complexes.^[^ ^55^ ^]^ Oxaliplatin, a well‐known cytostatic drug, was included for comparison. Where possible, inhibitory concentrations at half‐maximum levels (IC_50_) were calculated through sigmoidal fitting.

Varying results were observed for the different complexes. Notably, only those complexes with a bidentate auxiliary ligand were soluble at concentrations of ≥50 µm under cell culture conditions. Among these, only the complexes with the (modified) glycolate co‐ligand remained soluble at 100 µm. All Re(I) complexes, the Pt(II) complexes with tridentate ligands, and [**PtLH_2_Cl_2_**] precipitated at 10 or 50 µm. Thus, for the complexes with low solubility, the respective concentrations were excluded from the cytotoxicity evaluation. It is important to note that substances at concentrations up to 10 µm did not reduce cell viability by more than 50%. [**PtLH_2_cbda**] was soluble up to 50 µm but did not significantly inhibit cell viability either.

However, incubation with the two complexes featuring glycolate ligands resulted in a significant reduction in HepG2 cell viability. IC_50_ values 20 ± 10 µm, 12 ± 1 µm, and 60 ± 4 µm were obtained for [**PtLH_2_Gly**], [**PtLH_2_Tsgly**] and oxaliplatin, respectively (Table 2). Pascale and co‐workers found IC_50_ values for oxaliplatin against HepG2 cells of 2.7 µm ^[^ ^37^ ^]^ whereas Müller, Strassert and co‐workers found a value of 9.6 µm;^[^ ^83^ ^]^ this suggests different sensitivities of the cytotoxicity assays used. Interestingly, the effect against HepG2 cells of the two compounds tested herein (i.e., those with the (modified) glycolate co‐ligand) is even higher than that of oxaliplatin. Hong and co‐workers found comparable IC_50_ values for Pt(II) complexes with glycolate co‐ligands (e.g., 5.6 µm for nedaplatin)^[^ ^36^ ^]^ in comparison to cisplatin, and the successful use of nedaplatin in cancer treatment also suggests the value of this auxiliary ligand for cytotoxic activation. However, in contrast to the two cytotoxic agents discussed in this work, the rest of the tested complexes did not show any toxic effects.

**Table 2:** IC_50_ values for the complexes that lead to a significant reduction in HepG2 cell viability.

Complex	IC_50_ ± Std. dev. [µm]
**[PtLH_2_Gly]**	20 ± 10
**[PtLH_2_Tsgly]**	12 ± 1
**Oxaliplatin**	60 ± 4

## 3 Conclusion

In this study, we successfully synthesized a ligand precursor with three different coordination modes. Bi‐ and tridentate coordination to Pt(II) and bidentate coordination to Re(I) were achieved. Since the bidentate coordination motif for Pt(II) resembles typical Pt(II)‐based chemotherapeutic agents, the chlorido co‐ligands were exchanged for bidentate units. None of these complexes displayed intense emission at RT; even at 77 K, the emission was dominated by LC states.

Through microwave‐assisted cyclometallation, the tridentate coordination mode on Pt(II) was achieved, and the chlorido co‐ligand was replaced by a cyanido unit to increase the LFS. Both complexes exhibit green emission in solution, peaking around *λ*_Em_ = 500 nm and stemming from ^3^MP‐LC excited states, with [**PtLHCN**] outperforming all other complexes with a phosphorescence quantum yield of 0.55. This coordination mode results in intricate solid‐state behavior, where intermolecular interactions can be adjusted, leading to yellow phases with green monomeric emission or to red phases with emission from aggregated species. In addition, the aggregation in solution was studied, showing a stronger tendency of precipitation for [**PtLHCl**], and more favorable dimerization of [**PtLHCN**], resulting in delayed precipitation for the cyanido‐based complex.

The bidentate coordination mode also facilitates the easy complexation of Re(I), resulting in the formation of the pseudo‐octahedral compounds [**ReLH_2_(CO)_3_Br**] and [**ReLH_2_(CO)_3_Cl**]. Similar to the tridentate mode, the halogenido co‐ligand was exchanged by cyanido unit. All three complexes show typical reddish emission from a ^3^MLCT excited state, but only moderate cytotoxicity.

Considering these results and the phosphorescent properties of the tested complexes, optical bioimaging opens up as another field of application, as platinum complexes have been observed to accumulate in different cell compartments *in vitro*.^[^ ^85^, ^86^ ^]^ Hence, [**PtLH_2_cbda**], being the non‐cytotoxic complex with the best solubility, would be the most promising candidate; however, its low brightness might pose a limitation. Further tests to examine the accumulation of the complex in the cell can be foreseen to elucidate these possibilities. In general, the low solubility of many of the compounds could be the limiting factor. This could be overcome by modification of the substitution pattern (e.g., by introduction of hydrophilic moieties). It is noteworthy that to achieve reasonable solubility along with a competitive IC_50_ values for chemotherapeutic purposes, an unsymmetric bidentate co‐ligand is essential. In terms of cytotoxicity, two of the complexes reported herein even outperformed the commercially available oxaliplatin.

In conclusion, this ligand architecture with multiple coordination modes offers various potential applications depending on the coordination mode. Prospectively, the bidentate mode in combination with Pt(II) yield potentially cytostatic agents, whereas the Re(I) center leads to low cytotoxicity and could become an interesting platform for optical bioimaging, provided that the low photoluminescence quantum yields can be increased. The tunable emission and performance of the Pt(II) complexes with the tridentate coordination motive could inspire the development of triplet emitters for optoelectronic applications or optical sensors.

## Author Contributions

Stefan Buss and Toni M. A. Weise carried out the synthesis. Stefan Buss performed the spectroscopic characterization. Alexander Hepp did the NMR‐spectroscopic measurements. Alexander Hepp and Stefan Buss did the NMR‐spectroscopic analysis. Jutta Kösters performed the X‐ray diffractometric analysis. Lorenz Borsdorf analyzed the temperature and concentration dependent NMR‐measurements; María Victoria Cappellari performed the photophysical measurements in solution and glassy matrix; Iván Maisuls performed the photophysical measurements in solid state; Elisabeth C. Muschiol performed the cytotoxicity studies; Gustavo Fernández, Melanie Esselen, and Cristian A. Strassert supervised the work; Stefan Buss, Elisabeth C. Muschiol, and Lorenz Borsdorf wrote the draft manuscript; Iván Maisuls, María Victoria Cappellari, Melanie Esselen, Gustavo Fernández, and Cristian A. Strassert revised it.

## Conflict of Interests

The authors declare no conflicts of interest.

## Supporting information



Supporting Information

## Data Availability

CCDC 2394603 – 2394611 contain the supplementary crystallographic data for this paper. These data can be obtained free of charge via www.ccdc.cam.ac.uk/data_request/cif, or by emailing data_request@ccdc.cam.ac.uk, or by contacting The Cambridge Crystallographic Data Center, 12 Union Road, Cambridge CB2 1EZ, UK; fax: +44‐1223‐226033.
