# Supplementary material for: Aggregation, Photoluminescence, and Cytotoxicity of Pt(II) and Re(I) Complexes Bearing Multimodal‐Coordinating Luminophores
Source: Chemistry. 2025 Jun 4;31(35):e202404115. doi: 10.1002/chem.202404115 (PMC12188160; doi:10.1002/chem.202404115)
Supplement: Supplementary file 1 — Supporting Information [file CHEM-31-e202404115-s001.pdf]

Aggregation, Photoluminescence, and Cytotoxicity of Pt(II) and Re(I) Complexes Bearing Multimodal-Coordinating Luminophores

- Supporting Information -

Stefan Buss,[a,b] Lorenz Borsdorf,[c] Elisabeth C. Muschiol,[d] María Victoria Cappellari,[a,b] Iván Maisuls,[a,b] Toni M. A. Weise,[a,b], Alexander Hepp,[a] Jutta Kösters,[a] Melanie Esselen,[d] Gustavo Fernández[c] and Cristian A. Strassert[a,b]*

[a] Institut für Anorganische und Analytische Chemie - Universität Münster, Corrensstraße 28/30, 48149 Münster, Germany.

[b] CeNTech, CiMIC, SoN, Universität Münster, Heisenbergstraße 11, 48149 Münster, Germany.

[c] Organisch-Chemisches Institut, Universität Münster, Corrensstraße 36, 48149 Münster, Germany.

[d] Institut für Lebensmittelchemie, Universität Münster, Münster 48149, Germany.

<u>Contents:</u>

I. **Synthetic procedures and characterization**

I.1 *Synthesis*

I.2 *NMR spectra*

I.3 *FTIR spectra*

II. **X-ray diffractometry on single crystals**

III. **Photophysical characterization**

IV. **Aggregation study**

V. **Cytotoxicity test**

I. Synthetic procedures and characterization

I.1. Synthesis

Commercially available reagents were used without further purification. Silica gel 60 (0.063 – 0.200 mm) for column chromatography was purchased from Merck (mentioned as silica) was used for column chromatography if not otherwise stated. [$PtCl_2(DMSO)_2$] was prepared from $K_2[PtCl_4]$ using a literature-known procedure.[1,2]

Exact mass (EM) determination by mass spectrometry (MS) was carried out at the Organisch-Chemisches Institut (Univ. Münster) by Denise Defayay, using a LTQ Orbitrap LTQ XL (Thermo-Fisher Scientific, Bremen) with nanospray-injection (ESI) or Autoflex Speed MALDI-TOF with matrix assisted Laser desorption ionization (MALDI).

NMR spectra were obtained on an AV(Neo)500, AV(Neo)400, AV(III)400 or an AV(I)400 from Bruker. All measurements were obtained at room temperature if not otherwise mentioned. The ^{1}H-NMR and ^{13}C-NMR chemical shifts (δ) of the signals are given in parts per million and referenced to residual protons in the deuterated solvent: methylene-chloride-d_2 (DCM-d_2, 5.32 ppm / 54.0 ppm). The signal multiplicities are abbreviated as follows: s, singlet; d, doublet; t, triplet; q, quartet; m, multiplet.

Preparation of 1 and LH_2

Method a starting from the aniline derivative: The mixture of 2-chloro-4-phenylpyrimidine (2.00 g, 10.49 mmol, 2.0 eq.), 4-^{t}butylaniline (0.84 mL, 5.27 mmol, 1.0 eq.), NaO^{t}Bu (1.26 g, 13.11 mmol, 2.5 eq.), DPPF (122 mg, 0.22 mmol, 0.04 eq.), [$Pd_2(dba)_3$] (97 mg, 0.11 mmol, 0.02 eq.) and KI (30 mg, 0.18 mmol, 0.03 eq.) was suspended in toluene (30 mL) and purged with argon for 10 min before refluxed for 72 h. After reaching room temperature, H_2O (100 mL) was added to the mixture and the aqueous phase was extracted with EtOAc (4x50 mL). The combined organic phases were washed with H_2O (100 mL) and brine (100 mL) and dried over Na_2SO_4 before the solvent was removed under reduced pressure. The residue was purified *via* column chromatography over silica with cyclohexane:DCM = 1:4 as an eluent till the monosubstituted product **1** was obtained as a white crystalline solid. Yield: 678 mg; 2.23 mmol; 42%. Afterwards, the eluent was changed to cyclohexane:EtOAc = 3:2 to yield the disubstituted product **LH_2** as an off-white solid. Yield: 1.17 mg; 2.57 mmol; 49%.

Method b starting from 1: The mixture of 2-chloro-4-phenylpyrimidine (69 mg, 0.36 mmol, 1.0 eq.), **1** (108 mg, 0.36 mmol, 1.0 eq.), NaO^{t}Bu (89 mg, 0.93 mmol, 2.6 eq.), DPPF (8 g, 0.014 mmol, 0.04 eq.) and [$Pd_2(dba)_3$] (7 g, 0.008 mmol, 0.02 eq.) was suspended in toluene (30 mL) and purged with argon for 10 min before refluxed for 16 h. After reaching room temperature, H_2O (100 mL) was added to the mixture and the aqueous phase was extracted with EtOAc (4x50 mL). The combined organic phases were washed with H_2O (100 mL) and brine (100 mL) and dried over Na_2SO_4 before the solvent was removed under reduced pressure. The residue was purified *via* column chromatography over silica (cyclohexane:EtOAc = 3:2) to yield the disubstituted product **LH_2** as an off-white solid. Yield: 148 mg; 0.32 mmol; 90%.

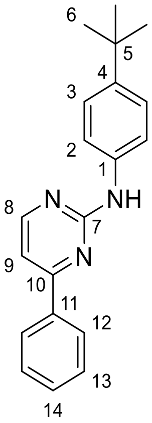

Analytics of 1:

^{1}H-NMR (400 MHz, CD_2Cl_2): δ (ppm) = 8.49 (d, $^3J_{HH}$ = 5.2 Hz, 1H, H_8), 8.19 – 8.05 (m, 2H, H_{12}), 7.77 (s, 1H, NH), 7.70 – 7.64 (m, 2H, H_2), 7.55 – 7.49 (m, 3H, H_{13+14}), 7.46 – 7.37 (m, 2H, H_3), 7.19 (d, $^3J_{HH}$ = 5.3 Hz, 1H, H_9), 1.37 (s, 9H, H_6).

^{13}C{^{1}H}-NMR (101 MHz, CD_2Cl_2): δ (ppm) = 165.2 (C_{10}), 161.0 (C_7), 159.0 (C_8), 145.7 (C_4), 137.7 (C_{11}), 137.6 (C_1), 131.1 (C_{14}), 129.2 (C_{13}), 127.5 (C_{12}), 126.0 (C_3), 119.6 (C_2), 108.5 (C_9), 34.6 (C_5), 31.6 (C_6).

MS-ESI-EM (MeOH, M = $C_{20}H_{21}N_3$), *m/z*: found 304.18037 for $[M+H]^+$, calcd. 304.18082 for $[M+H]^+$; found 326.16248 for $[M+Na]^+$, calcd. 326.16277 for $[M+Na]^+$.

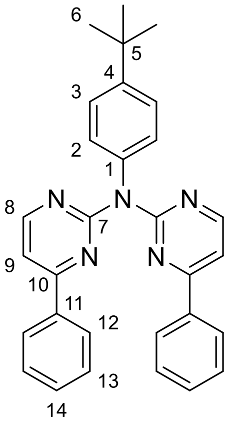

Analytics of LH_2:

^{1}H-NMR (400 MHz, CD_2Cl_2): δ (ppm) = 8.70 (d, $^3J_{HH}$ = 5.2 Hz, 2H, H_8), 8.22 – 7.97 (m, 4H, H_{12}), 7.66 – 7.61 (m, 2H, H_3), 7.59 – 7.52 (m, 2H, H_{14}), 7.52 – 7.44 (m, 8H, H_{2+9+13}), 1.54 (s, 9H, H_6).

^{13}C{^{1}H}-NMR (101 MHz, CD_2Cl_2): δ (ppm) = 165.1 (C_{10}), 163.5 (C_7), 159.1 (C_8), 149.2 (C_4), 141.4 (C_1), 137.1 (C_{11}), 131.2 (C_{14}), 129.1 (C_{13}), 127.5 (C_{12}), 127.5 (C_2), 126.4 (C_3), 111.9 (C_9), 34.9 (C_5), 31.6 (C_6).

MS-ESI-EM (MeOH, M = $C_{30}H_{27}N_5$), *m/z*: found 458.23374 for $[M+H]^+$, calcd. 458.23392 for $[M+H]^+$; found 480.21550 for $[M+Na]^+$, calcd. 480.21587 for $[M+Na]^+$.

Preparation of [$PtLH_2Cl_2$]

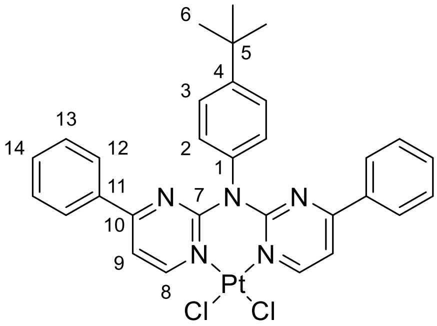

[$PtCl_2(DMSO)_2$] (139 mg, 0.33 mmol, 1.0 eq.) and **LH_2** (150 mg, 0.33 mmol, 1.0 eq.) were suspended in DCM (40 mL) and stirred for 120 h at room temperature. The mixture was purified *via* column chromatography over silica (DCM) to yield the product [**$PtLH_2Cl_2$**] as a yellow solid. Yield: 209 mg; 0.29 mmol; 88%.

^{1}H-NMR (400 MHz, CD_2Cl_2): δ (ppm) = 9.45 (d, $^3J_{HH}$ = 6.4 Hz, 2H, H_8), 7.85 – 7.78 (m, 4H, H_{12}), 7.75 – 7.66 (m, 2H, H_3), 7.61 – 7.49 (m, 6H, H_{2+9+14}), 7.46 – 7.37 (m, 4H, H_{13}), 1.50 (s, 9H, H_6).

^{13}C{^{1}H}-NMR (101 MHz, CD_2Cl_2): δ (ppm) = 165.3 (C_{10}), 160.4 (C_8), 155.5 (C_7), 152.4 (C_4), 138.0 (C_1), 134.4 (C_{11}), 133.3 (C_{14}), 130.2 (C_2), 129.6 (C_{13}), 128.2 (C_{12}), 126.4 (C_3), 112.8 (C_9), 35.3 (C_5), 31.6 (C_6).

^{195}Pt{^{1}H}-NMR (86 MHz, CD_2Cl_2): δ (ppm) = -2078.

MS-ESI-EM (MeOH, M = $C_{30}H_{27}N_5PtCl_2$), *m/z*: found 746.11770 for $[M+Na]^+$, calcd. 746.11735 for $[M+Na]^+$.

General method for the chloride ligand exchange of [$PtLH_2Cl_2$]:

[**$PtLH_2Cl_2$**] (1.0 eq.) and silver(I) trifluoroacetate (2.2 eq.) were stirred in MeOH (25 mL) for 1 h at room temperature. Additionally, the bidentate ligand precursors (1.1 eq.) were stirred with NaOMe (2.2 eq.) in MeOH (25 mL) for 1 h at room temperature, before the solution was added to the flask containing the Pt(II) complex. The mixture was purged with argon for 10 min and the refluxed for 4 h. The solvent was removed under reduced pressure and the residue was purified *via* column chromatography over silica (DCM + MeOH 0-2%) to yield product.

Preparation of [$PtLH_2Gly$]

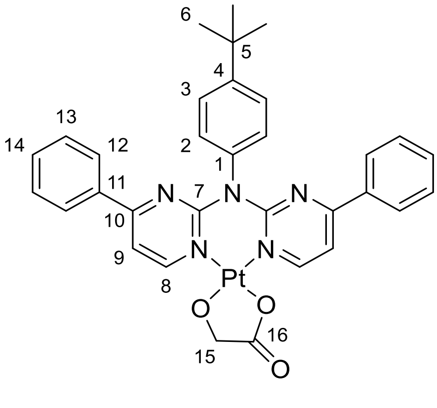

[**$PtLH_2Cl_2$**] (65 mg, 0.090 mmol), silver(I) trifluoroacetate (44 mg, 0.198 mmol), glycolic acid (7.5 mg, 0.100 mmol) and NaOMe (10.7 mg, 0.198 mmol) were refluxed for 4 h. After purification *via* column chromatography, the product was obtained a a yellow-orange solid. Yield: 44 mg; 0.061 mmol; 68%.

^{1}H-NMR (400 MHz, CD_2Cl_2): δ (ppm) = 9.32 (d, $^3J_{HH}$ = 6.4 Hz, 1H, H_8), 9.31 (d, $^3J_{HH}$ = 6.4 Hz, 1H, $H_{8'}$), 7.71 – 7.64 (m, 6H, $H_{3+3'+12+12'}$), 7.55 – 7.49 (m, 2H, $H_{14+14'}$), 7.42 – 7.26 (m, 8H, $H_{2+9+9'+13+13'}$), 4.34 (s, 2H, H_{15}), 1.48 (s, 9H, H_6).

^{13}C{^{1}H}-NMR (101 MHz, CD_2Cl_2): δ (ppm) = 190.9 (C_{16}), 163.6 (C_{10}), 163.0 ($C_{10'}$), 158.4 ($C_{8'}$), 156.0 (C_8), 153.5 (C_7), 153.3 ($C_{7'}$), 151.8 (C_4), 139.5 (C_1), 134.9 ($C_{11'}$), 134.7 (C_{11}), 132.9 (C_{14}), 132.8 ($C_{14'}$), 129.5 ($C_{13/13'}$), 129.5 ($C_{13/13'}$), 129.1 (C_2), 127.8 (C_{12}), 127.7 ($C_{12'}$), 126.8 (C_3), 111.9 ($C_{9'}$), 111.4 (C_9), 71.7 (C_{15}), 35.2 (C_5), 31.6 (C_6).

^{195}Pt{^{1}H}-NMR (86 MHz, CD_2Cl_2): δ (ppm) = -1541.

MS-ESI-EM (MeOH, M = $C_{32}H_{29}N_5O_3Pt$), *m/z*: found 727.19956 for $[M+H]^+$, calcd. 727.19934 for $[M+H]^+$; found 749.18160 for $[M+Na]^+$, calcd. 749.18129 for $[M+Na]^+$.

Preparation of [PtLH$_2$cbda]

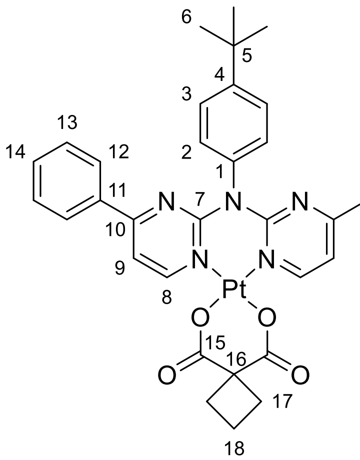

[**PtLH$_2$Cl$_2$**] (64 mg, 0.090 mmol), silver(I) trifluoroacetate (43 mg, 0.19 mmol), 1,1-cyclobutanedicarboxylic acid (14 mg, 0.100 mmol) and NaOMe (11 mg, 0.19 mmol) were refluxed for 4 h. After purification *via* column chromatography, the product was obtained a yellow solid. Yield: 63 mg; 0.079 mmol; 88%.

^{1}H-NMR (400 MHz, CD$_2$Cl$_2$): δ (ppm) = 9.23 (d, $^3J_{HH}$ = 6.5 Hz, 2H, H$_8$), 7.78 – 7.72 (m, 4H, H$_{12}$), 7.72 – 7.67 (m, 2H, H$_3$), 7.59 – 7.50 (m, 4H, H$_{9+14}$), 7.46 – 7.33 (m, 6H, H$_{2+13}$), 2.94 – 2.80 (m, 4H, H$_{17}$), 2.00 – 1.81 (m, 2H, H$_{18}$), 1.49 (s, 9H, H$_6$).

^{13}C{^{1}H}-NMR (101 MHz, CD$_2$Cl$_2$): δ (ppm) = 177.9 (C$_{15}$), 164.8 (C$_{10}$), 156.9 (C$_8$), 154.6 (C$_7$), 152.3 (C$_4$), 138.9 (C$_1$), 134.4 (C$_{11}$), 133.3 (C$_{14}$), 129.6 (C$_{13}$), 129.3 (C$_2$), 128.1 (C$_{12}$), 126.8 (C$_3$), 112.1 (C$_9$), 56.2 (C$_{16}$), 35.3 (C$_5$), 31.6 (C$_6$), 31.2 (C$_{17}$), 16.0 (C$_{18}$).

^{195}Pt{^{1}H}-NMR (86 MHz, CD$_2$Cl$_2$): δ (ppm) = -1666.

MS-ESI-EM (MeOH, M = C$_{36}$H$_{33}$N$_5$O$_4$Pt), *m/z*: found 795.22561 for [M+H]$^+$, calcd. 795.22559 for [M+H]$^+$; found 817.20742 for [M+Na]$^+$, calcd. 817.20727 for [M+Na]$^+$.

Preparation of [PtLH$_2$Tsgly]

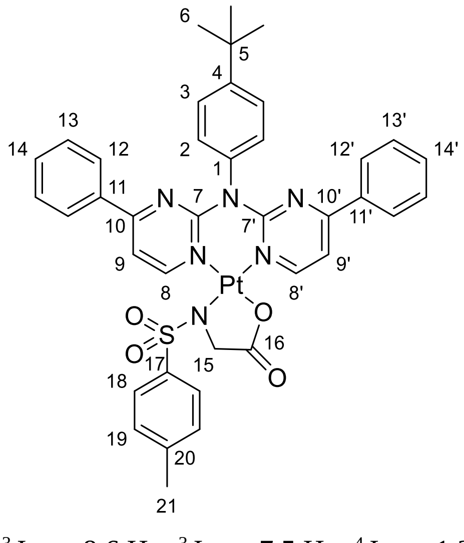

[**PtLH$_2$Cl$_2$**] (55.7 mg, 0.077 mmol), silver(I) trifluoroacetate (38.4 mg, 0.174 mmol), *N*-(*p*-toluenesulfonyl)glycine (18.5 mg, 0.081 mmol) and NaOMe (10.7 mg, 0.198 mmol) were refluxed for 4 h. After purification *via* column chromatography the product was obtained a yellow solid. Yield: 64.5 mg; 0.073 mmol; 95%.

^{1}H-NMR (500 MHz, CD$_2$Cl$_2$): δ (ppm) = 9.48 (d, $^3J_{HH}$ = 6.4 Hz, 1H, H$_8$), 8.89 (d, $^3J_{HH}$ = 6.4 Hz, 1H, H$_{8'}$), 8.06 (d, $^3J_{HH}$ = 8.3 Hz, 2H, H$_{18}$), 7.80 (dd, $^3J_{HH}$ = 8.5 Hz, $^4J_{HH}$ = 1.2 Hz, 2H, H$_{12}$), 7.79 – 7.75 (m, 2H, H$_{12'}$), 7.75 – 7.71 (m, 2H, H$_3$), 7.55 (ddt, $^3J_{HH}$ = 7.4 Hz, $^3J_{HH}$ = 5.8 Hz, $^4J_{HH}$ = 1.3 Hz, 2H, H$_{14+14'}$), 7.53 – 7.51 (m, 1H, H$_9$), 7.51 – 7.48 (m, 2H, H$_2$), 7.47 (d, $^3J_{HH}$ = 6.4 Hz, 1H, H$_{9'}$), 7.40 (ddd, $^3J_{HH}$ = 8.6 Hz, $^3J_{HH}$ = 7.5 Hz, $^4J_{HH}$ = 1.3 Hz, 4H, H$_{13+13'}$), 7.32 – 7.26 (m, 2H, H$_{19}$), 4.10 (s, 2H, H$_{15}$), 2.38 (s, 3H, H$_{21}$), 1.51 (s, 9H, H$_6$).

^{13}C{^{1}H}-NMR (126 MHz, CD$_2$Cl$_2$): δ (ppm) = 184.2 (C$_{16}$), 165.1 (C$_{10'}$), 164.6 (C$_{10}$), 162.9 (C$_8$), 157.9 (C$_{8'}$), 154.7 (C$_{7'}$), 154.6 (C$_7$), 152.4 (C$_4$), 142.1 (C$_{20}$), 140.6 (C$_{17}$), 138.4 (C$_1$), 134.6 (C$_{11'}$), 134.5 (C$_{11}$), 133.2 (C$_{14'}$), 133.1 (C$_{14}$), 129.9 (C$_2$), 129.7 (C$_{13}$), 129.6 (C$_{19}$), 129.5 (C$_{13'}$), 128.0 (C$_{12+12'}$), 128.0 (C$_{18}$), 126.6 (C$_3$), 112.4 (C$_{9'}$), 111.4 (C$_9$), 54.9 (C$_{15}$), 35.2 (C$_5$), 31.6 (C$_6$), 21.6 (C$_{21}$).

^{195}Pt{^{1}H}-NMR (107 MHz, CD$_2$Cl$_2$): δ (ppm) = -1987.

No prediction of the orientation of the ligands (*cis*/*trans* of 8/8') can be made, no signal interactions between 8/8' and 18 where observed.

MS-ESI-EM (MeOH/CHCl$_3$, M = C$_{39}$H$_{36}$N$_6$O$_4$SPt), *m/z*: found 880.22552 for [M+H]$^+$, calcd. 880.22420 for [M+H]$^+$; found 902.20691 for [M+Na]$^+$, calcd. 902.20615 for [M+Na]$^+$; found

1782.42568 for $[2M+Na]^+$, calcd. 1782.42387 for $[2M+Na]^+$; found 1342.31632 for $[3M+2Na]^{2+}$, calcd. 1342.31492 for $[3M+2Na]^{2+}$;

Preparation of [PtLHCl]

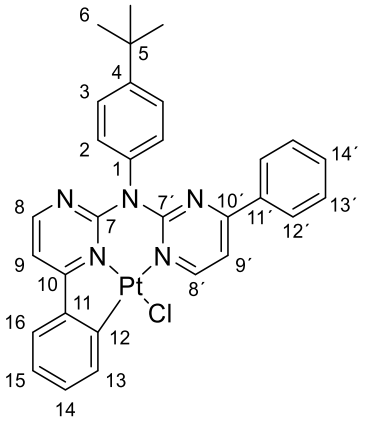

LH_2 (115 mg, 0.251 mmol, 1.0 eq) and $K_2[PtCl_4]$ (104 mg, 0.251 mmol, 1.0 eq) were, together with few crystals of $^nBu_4NCl$ (cat.), suspended in glacial acetic acid (20 mL). The mixture was purged with argon for 10 min before heated in a microwave. The mixture was heated for 30 min up to 250°C and kept by this temperature for 30 min using 850 kW (CAUTION: not every microwave can operate these conditions, 3 out 4 reactions worked out perfectly fine for us, for the 4th reaction the pressure release did not work and the reaction vessel bursted!). The solvent was removed using reduced pressure and the residue was purified *via* column chromatography over silica (DCM) to yield the product as red solid. Yield: 158 mg; 0.230 mmol; 92%.

^{1}H-NMR (500 MHz, CD_2Cl_2): δ (ppm) = 10.13 (d, $^3J_{HH}$ = 6.3 Hz, 1H, $H_{8'}$), 8.57 (d, $^3J_{HH}$ = 5.1 Hz, 1H, H_8), 8.12 (dd, $^3J_{HH}$ = 7.9 Hz, $^4J_{HH}$ = 1.3 Hz, 1H, H_{13}), 7.67 – 7.60 (m, 2H, H_3), 7.62 – 7.57 (m, 3H, $H_{12'+16}$), 7.50 (tt, $^3J_{HH}$ = 7.1 Hz, $^4J_{HH}$ = 1.3 Hz, 1H, $H_{14'}$), 7.42 (d, $^3J_{HH}$ = 6.3 Hz, 1H, $H_{9'}$), 7.36 – 7.30 (m, 3H, $H_{9+13'}$), 7.30 – 7.25 (m, 3H, H_{2+14}), 7.13 (td, $^3J_{HH}$ = 7.5 Hz, $^4J_{HH}$ = 1.3 Hz, 1H, H_{15}), 1.48 (s, 9H, H_6).

^{13}C{^{1}H}-NMR (126 MHz, CD_2Cl_2): δ (ppm) = 175.3 (C_{10}), 163.3 ($C_{10'}$), 157.9 ($C_{8'}$), 156.9 (C_8), 154.7 ($C_{7'}$), 154.3 (C_7), 151.1 (C_4), 145.8 (C_{12}), 143.1 (C_{11}), 141.7 (C_1), 136.0 (C_{13}), 135.3 ($C_{11'}$), 132.6 ($C_{14'}$), 132.0 (C_{14}), 129.4 ($C_{13'}$), 129.2 (C_2), 127.8 ($C_{12'}$), 126.8 (C_3), 125.3 (C_{16}), 124.5 (C_{15}), 110.8 ($C_{9'}$), 109.0 (C_9), 35.2 (C_5), 31.7 (C_6).

^{195}Pt{^{1}H}-NMR (107 MHz, CD_2Cl_2): δ (ppm) = -3249.

MS-ESI-EM (MeOH/$CHCl_3$, M = $C_{30}H_{26}N_5PtCl$), *m/z*: found 709.14269 for $[M+Na]^+$, calcd. 709.14190 for $[M+Na]^+$.

Preparation of [PtLHCN]

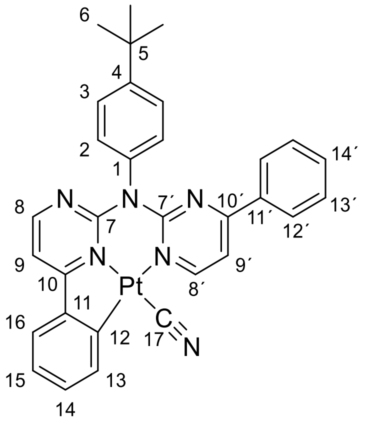

[**PtLHCl**] (52.2 mg, 0.076 mmol, 1.0 eq) and AgCN (18.2 mg, 0.137 mmol, 1.8 eq.) were suspended in MeCN (20 mL). The mixture was purged for 10 min and refluxed for 20 h. The solvent was removed using reduced pressure and the residue was purified *via* column chromatography over silica (DCM/MeOH 2 %) to yield the product as red solid. Yield: 44 mg; 0.065 mmol; 85%.

^{1}H-NMR (400 MHz, CD_2Cl_2): δ (ppm) = 9.71 (d, $^3J_{HH}$ = 6.2 Hz, 1H, $H_{8'}$), 8.61 (d, $^3J_{HH}$ = 5.0 Hz, 1H, H_8), 8.05 (dd, $^3J_{HH}$ = 7.7 Hz, $^4J_{HH}$ = 1.4 Hz, 1H, H_{13}), 7.70 – 7.63 (m, 2H, H_3), 7.62 – 7.55 (m, 3H, $H_{12'+16}$), 7.54 – 7.47 (m, 1H, $H_{14'}$), 7.37 – 7.29 (m, 6H, $H_{2+9+9'+13'}$), 7.15 (td, $^3J_{HH}$ = 7.5 Hz, $^3J_{HH}$ = 1.4 Hz, 1H, H_{14}), 7.06 (td, $^3J_{HH}$ = 7.5 Hz, $^4J_{HH}$ = 1.3 Hz, 1H, H_{15}), 1.49 (s, 9H, H_6).

^{13}C{^{1}H}-NMR (101 MHz, CD_2Cl_2): δ (ppm) = 174.0 (C_{10}), 163.7 ($C_{10'}$), 163.0 ($C_{8'}$), 158.9 (C_8), 155.0 ($C_{7'}$), 154.9 (C_7), 151.2 (C_4), 144.3 (C_{12}), 142.6 (C_{11}), 141.7 (C_1), 139.7 (C_{13}), 139.2 (C_{17}), 135.0 ($C_{11'}$),

133.4 (C_{14}), 132.9 ($C_{14'}$), 129.4 ($C_{13'}$), 129.0 (C_2), 128.0 ($C_{12'}$), 127.0 (C_3), 125.8 (C_{16}), 124.7 (C_{15}), 111.5 ($C_{9'}$), 109.1 (C_9), 35.2 (C_5), 31.7 (C_6).

^{195}Pt{^{1}H}-NMR (86 MHz, CD_2Cl_2): δ (ppm) = -3677.

MS-ESI-EM (MeOH/$CHCl_3$, M = $C_{31}H_{26}N_6Pt$) , *m/z*: found 700.17686 for $[M+Na]^+$, calcd. 700.17612 for $[M+Na]^+$; found 1377.36403 for $[2M+Na]^+$, calcd. 1377.36228 for $[2M+Na]^+$.

IR (ATR): $\tilde{\upsilon}$ (cm^{-1}) = 2120 (C≡N).

Preparation of [ReLH$_2$(CO)$_3$Br] and [ReLH$_2$(CO)$_3$Cl]

[$Re(CO)_5Br$] (91.4 mg, 0.225 mmol, 1.0 eq.) and **LH$_2$** (103 mg, 0.225 mmol, 1.0 eq.) were dissolved in MeOH (15 mL). The mixture was purged with argon for 10 min and afterwards refluxed for 16 h. The solvent was removed under reduced pressure and the residue was purified *via* column chromatography over silica (DCM) to yield the [**ReLH$_2$(CO)$_3$Br**] product as the first fraction as a pale-yellow solid. Yield: 90 mg; 0.111 mmol; 50%. The second fraction contains the chloride complex [**ReLH$_2$(CO)$_3$Cl**] also as a pale-yellow solid. Yield: 15 mg; 0.020 mmol; 9%.

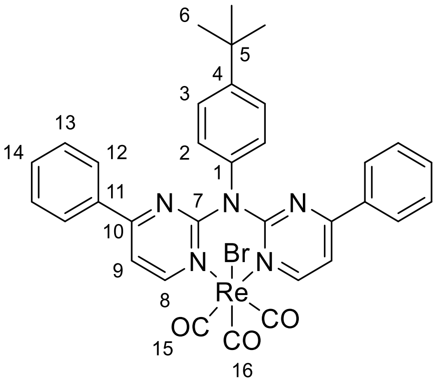

Analytics of [ReLH$_2$(CO)$_3$Br]:

^{1}H-NMR (500 MHz, CD_2Cl_2): δ (ppm) = 9.13 (d, $^3J_{HH}$ = 6.2 Hz, 2H, H_8), 7.88 – 7.80 (m, 4H, H_{12}), 7.71 – 7.66 (m, 2H, H_3), 7.66 – 7.61 (m, 2H, H_2), 7.58 (d, $^3J_{HH}$ = 6.2 Hz, 2H, H_9), 7.56 – 7.50 (m, 2H, H_{14}), 7.43 (m, 4H, H_{13}), 1.48 (s, 9H, H_6).

^{13}C{^{1}H}-NMR (126 MHz, CD_2Cl_2): δ (ppm) = 195.7 (C_{15}), 191.7 (C_{16}), 165.8 (C_{10}), 163.8 (C_8), 159.5 (C_7), 152.2 (C_4), 138.6 (C_1), 134.8 (C_{11}), 133.1 (C_{14}), 130.7 (C_2), 129.5 (C_{13}), 128.1 (C_{12}), 126.0 (C_3), 112.7 (C_9), 35.2 (C_5), 31.6 (C_6).

MS-ESI-EM (MeOH, M = $C_{33}H_{27}N_5O_3ReBr$), *m/z*: found 808.09248 for $[M+H]^+$, calcd. 808.09111 for $[M+H]^+$; found 830.07288 for $[M+Na]^+$, calcd. 830.07306 for $[M+Na]^+$; found 728.16676 for $[M-Cl]^+$, calcd. 728.16673 for $[M-Cl]^+$.

IR (ATR): $\tilde{\upsilon}$ (cm^{-1}) = 2017 (C≡O), 1924 (C≡O), 1891 (C≡O).

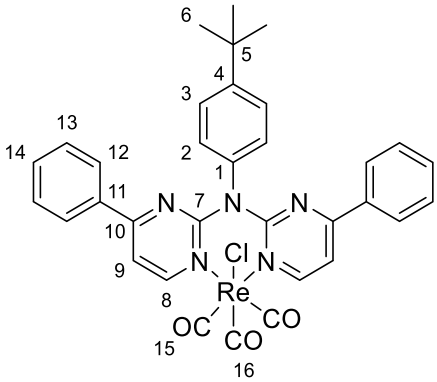

Analytics of [ReLH$_2$(CO)$_3$Cl]:

^{1}H-NMR (400 MHz, CD_2Cl_2) δ (ppm) = 9.03 (d, $^3J_{HH}$ = 6.1 Hz, 2H, H_8), 7.92 – 7.76 (m, 4H, H_{12}), 7.71 – 7.61 (m, 4H, H_{2+3}), 7.59 (d, $^3J_{HH}$ = 6.2 Hz, 2H, H_9), 7.56 – 7.50 (m, 2H, H_{14}), 7.47 – 7.38 (m, 4H, H_{13}), 1.47 (s, 9H, H_6).

^{13}C{^{1}H}-NMR (101 MHz, CD_2Cl_2) δ (ppm) = 196.0 (C_{15}), 192.2 (C_{16}), 165.9 (C_{10}), 163.1 (C_8), 159.4 (C_7), 152.2 (C_4), 138.5 (C_1), 134.9 (C_{11}), 133.1 (C_{14}), 130.7 (C_2), 129.5 (C_{13}), 128.2 (C_{12}), 126.0 (C_3), 112.7 (C_9), 35.2 (C_5), 31.6 (C_6).

MS-ESI-EM (MeOH, M = $C_{33}H_{27}N_5O_3ReCl$), *m/z*: found 786.12483 for $[M+Na]^+$, calcd. 786.12521 for $[M+Na]^+$; found 728.16693 for $[M-Cl]^+$, calcd. 728.16673 for $[M-Cl]^+$.

IR (ATR): $\tilde{\upsilon}$ (cm^{-1}) = 2017 (C≡O), 1912 (C≡O), 1889 (C≡O).

Preparation of [ReLH2(CO)3CN]

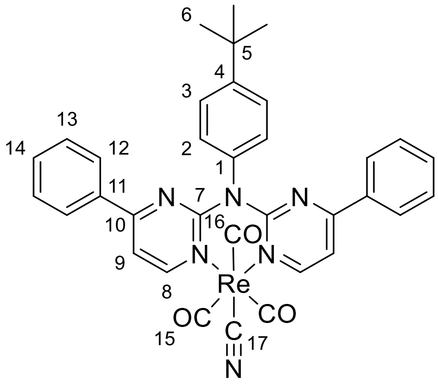

[**ReLH2(CO)3Br**] (36 mg, 0.045 mmol, 1.0 eq) and AgCN (10.7 mg, 0.080 mmol, 1.8 eq.) were suspended in MeCN (15 mL). The mixture was purged for 10 min and refluxed for 16 h. The solvent was removed using reduced pressure and the residue was purified *via* column chromatography over silica (DCM/MeOH 2 %) to yield the product as yellow solid. Yield: 31 mg; 0.041 mmol; 91%.

^{1}H-NMR (400 MHz, CD_2Cl_2): δ (ppm) = 9.06 (d, $^{3}J_{HH}$ = 6.2 Hz, 2H, H_8), 7.86 – 7.78 (m, 4H, H_{12}), 7.70 – 7.65 (m, 2H, H_3), 7.64 – 7.60 (m, 2H, H_3), 7.56 (d, $^{3}J_{HH}$ = 6.2 Hz, 2H, H_9), 7.55 – 7.49 (m, 2H, H_{14}), 7.47 – 7.39 (m, 4H, H_{13}), 1.48 (s, 9H, H_6).

^{13}C{^{1}H}-NMR (101 MHz, CD_2Cl_2): δ (ppm) = 194.4 (C_{15}), 194.2 (C_{16}), 166.1 (C_{10}), 163.7 (C_8), 159.8 (C_7), 152.3 (C_4), 147.2 (C_{17}), 138.6 (C_1), 134.7 (C_{11}), 133.2 (C_{14}), 130.6 (C_2), 129.5 (C_{13}), 128.1 (C_{12}), 126.0 (C_3), 112.8 (C_9), 35.2 (C_5), 31.6 (C_6).

MS-ESI-EM (MeOH/DCM, M = $C_{34}H_{27}N_6O_3Re$), *m/z*: found 777.15978 for $[M+Na]^+$, calcd. 777.15958 for $[M+Na]^+$.

IR (ATR): $\tilde{\upsilon}$ (cm^{-1}) = 2123 (C≡N), 2017 (C≡O), 1910 (C≡O).

I.2 *NMR spectra*

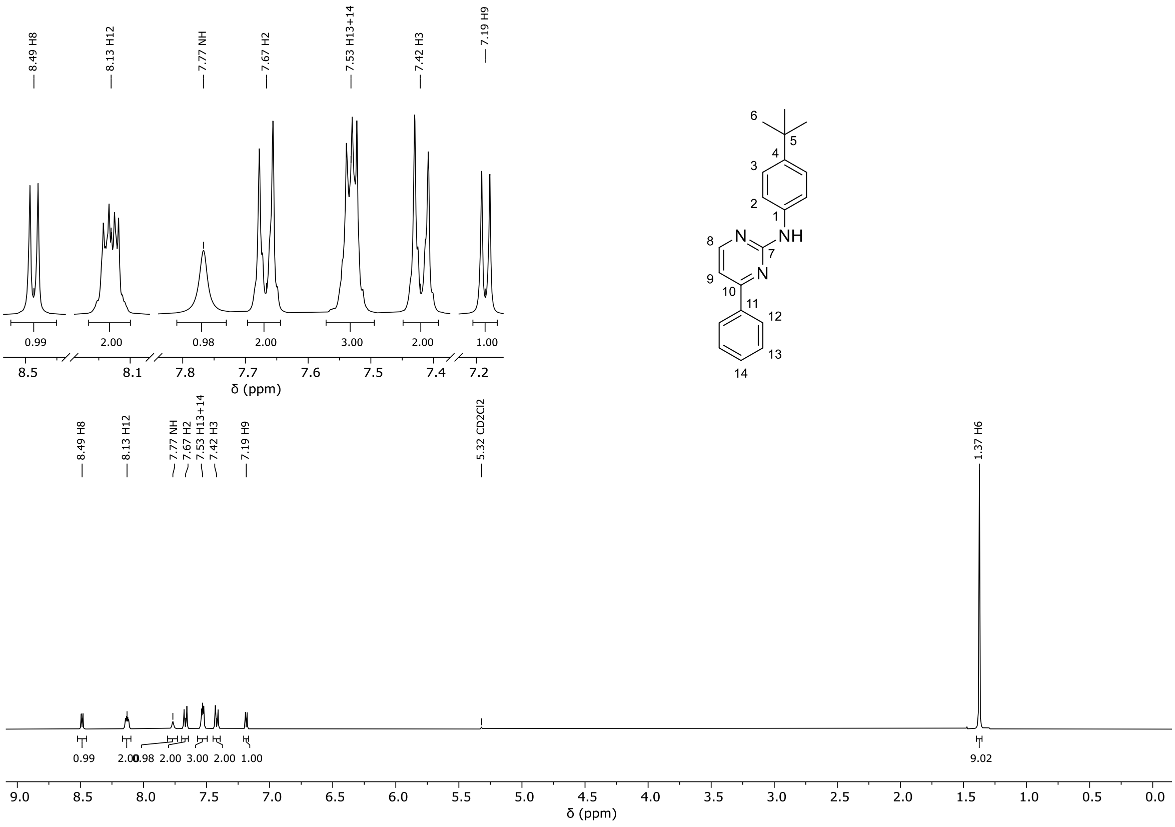

Figure S1: ^{1}H-NMR spectrum (400 MHz, CD_2Cl_2) of **1**.

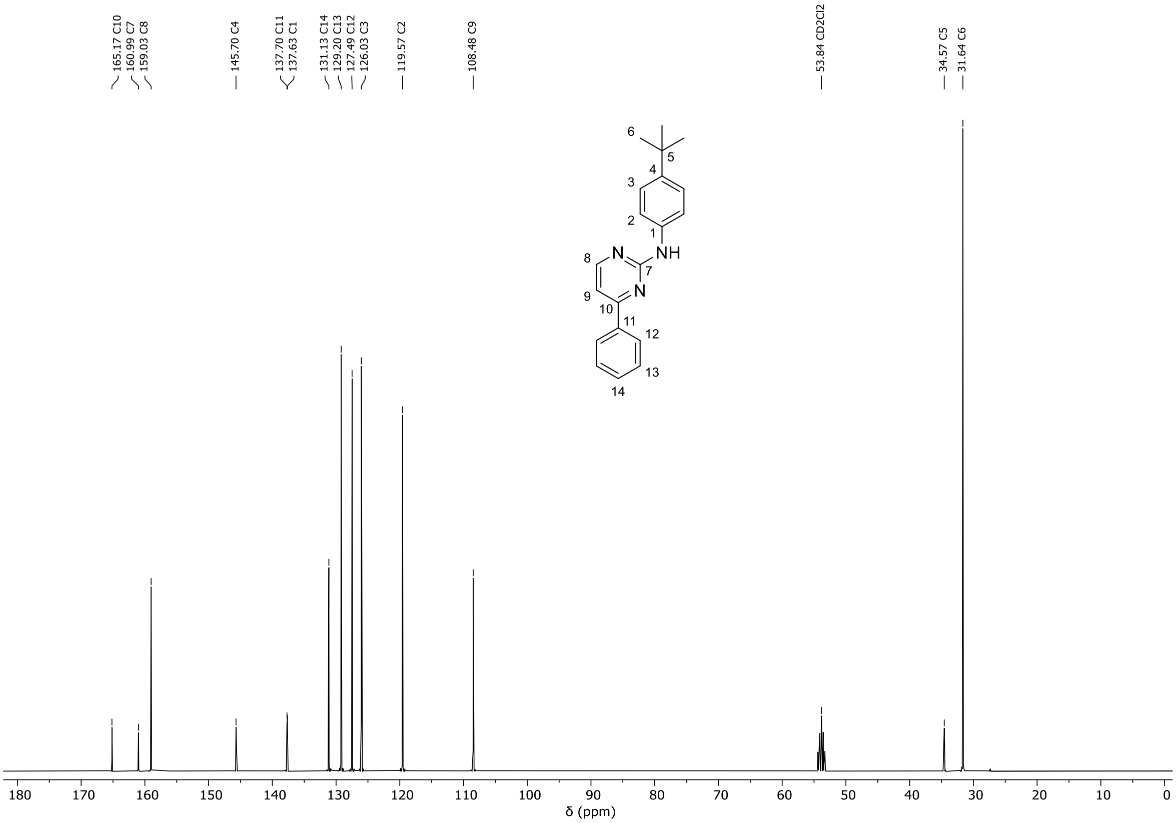

Figure S2: ^{13}C{^{1}H}-NMR spectrum (101 MHz, CD_2Cl_2) of **1**.

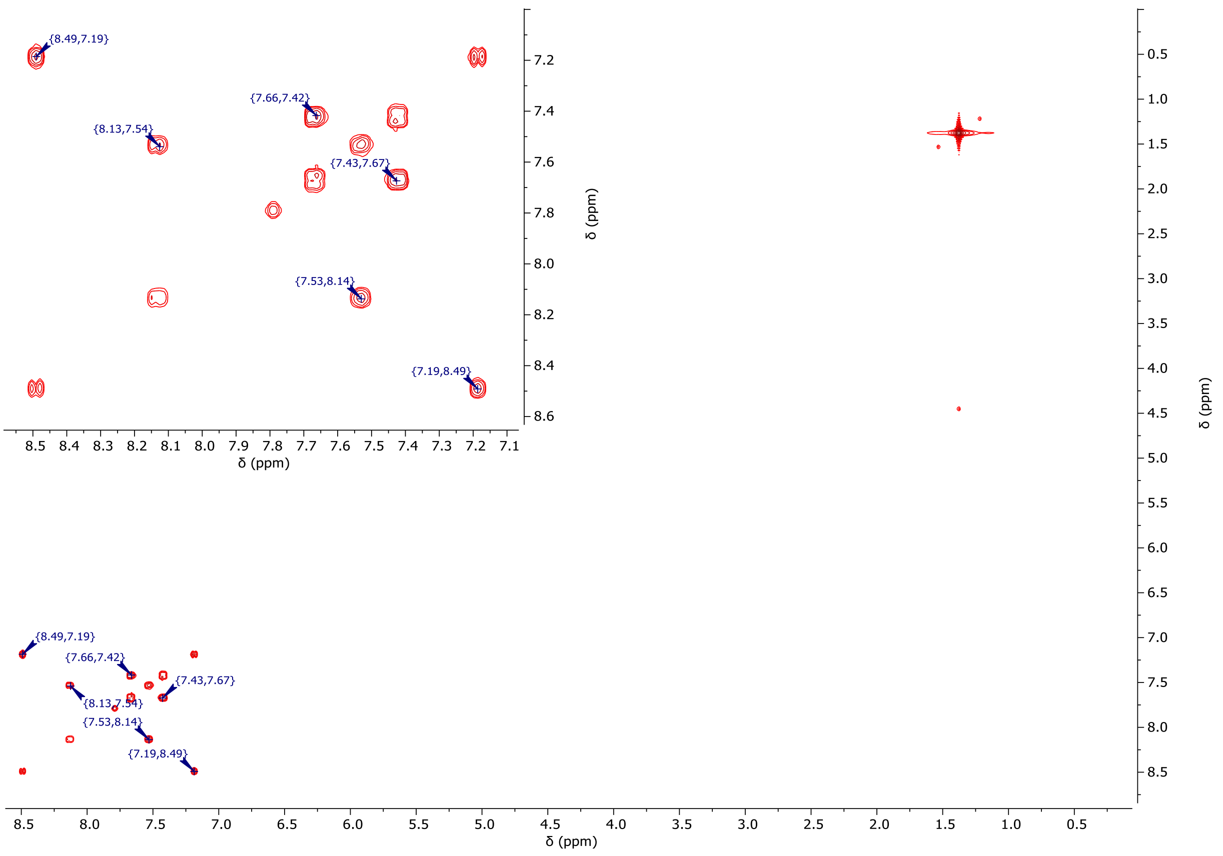

Figure S3: ¹H/¹H-COSY-NMR spectrum (400 MHz/400 MHz, CD_2Cl_2) of **1**.

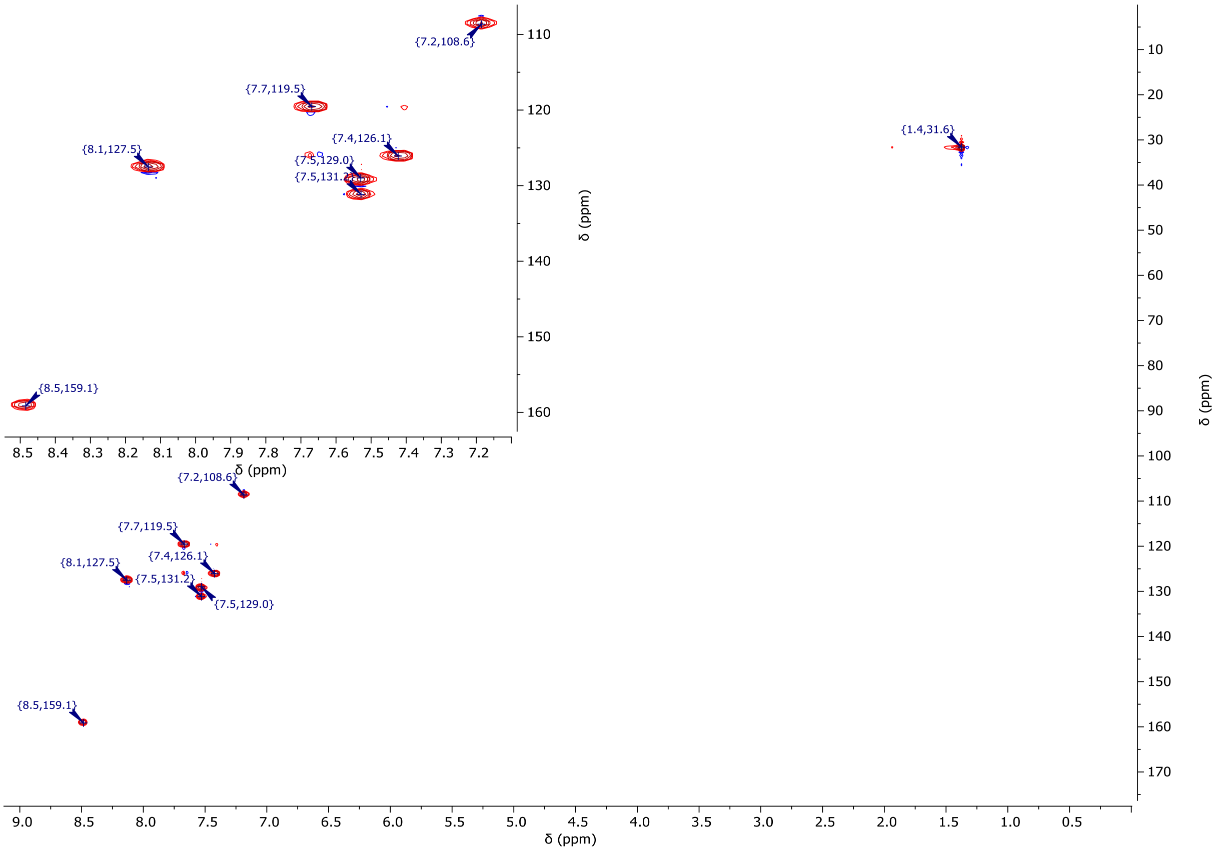

Figure S4: ¹H/¹³C-gHSQC-NMR spectrum (400 MHz/101 MHz, CD_2Cl_2) of **1**.

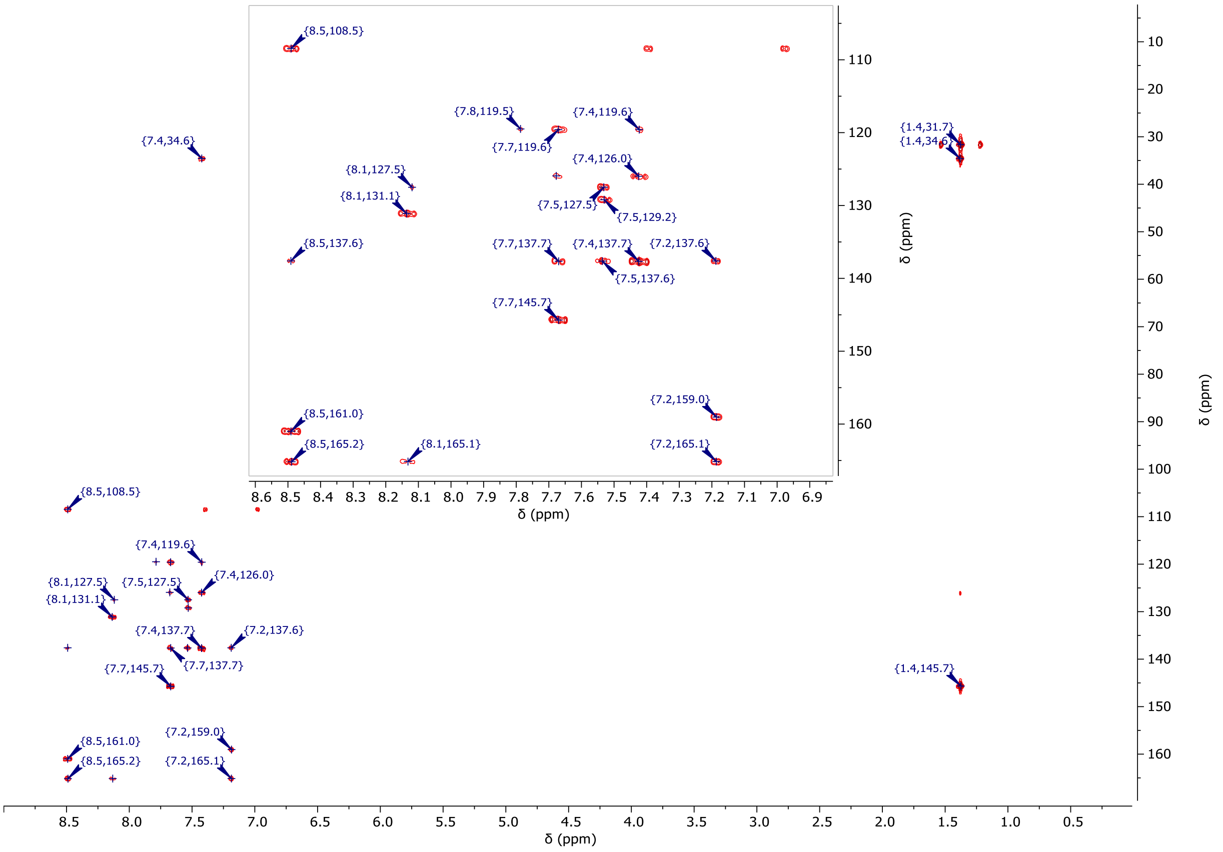

Figure S5: $^1H/^{13}C$-gHMBC-NMR spectrum (400 MHz/101 MHz, CD_2Cl_2) of **1**.

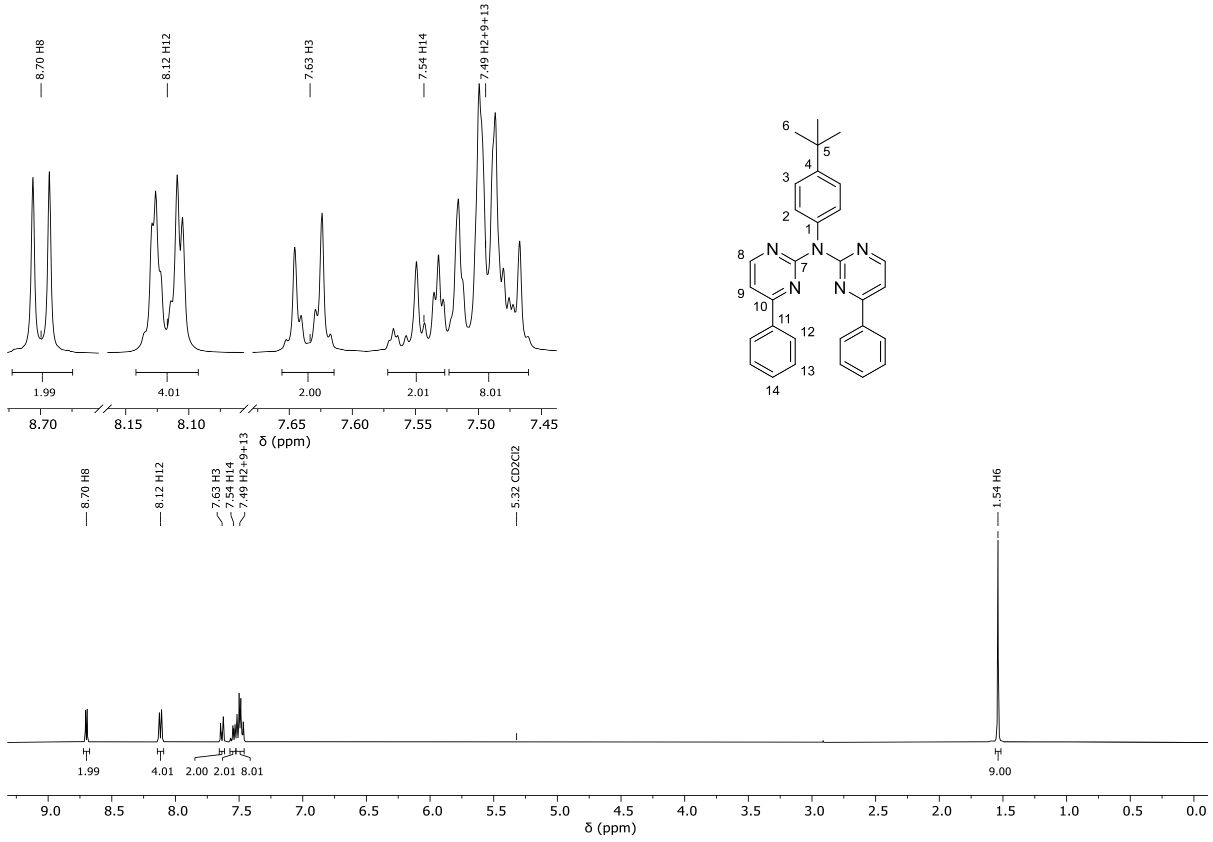

Figure S6: ^1H-NMR spectrum (400 MHz, CD_2Cl_2) of **LH₂**.

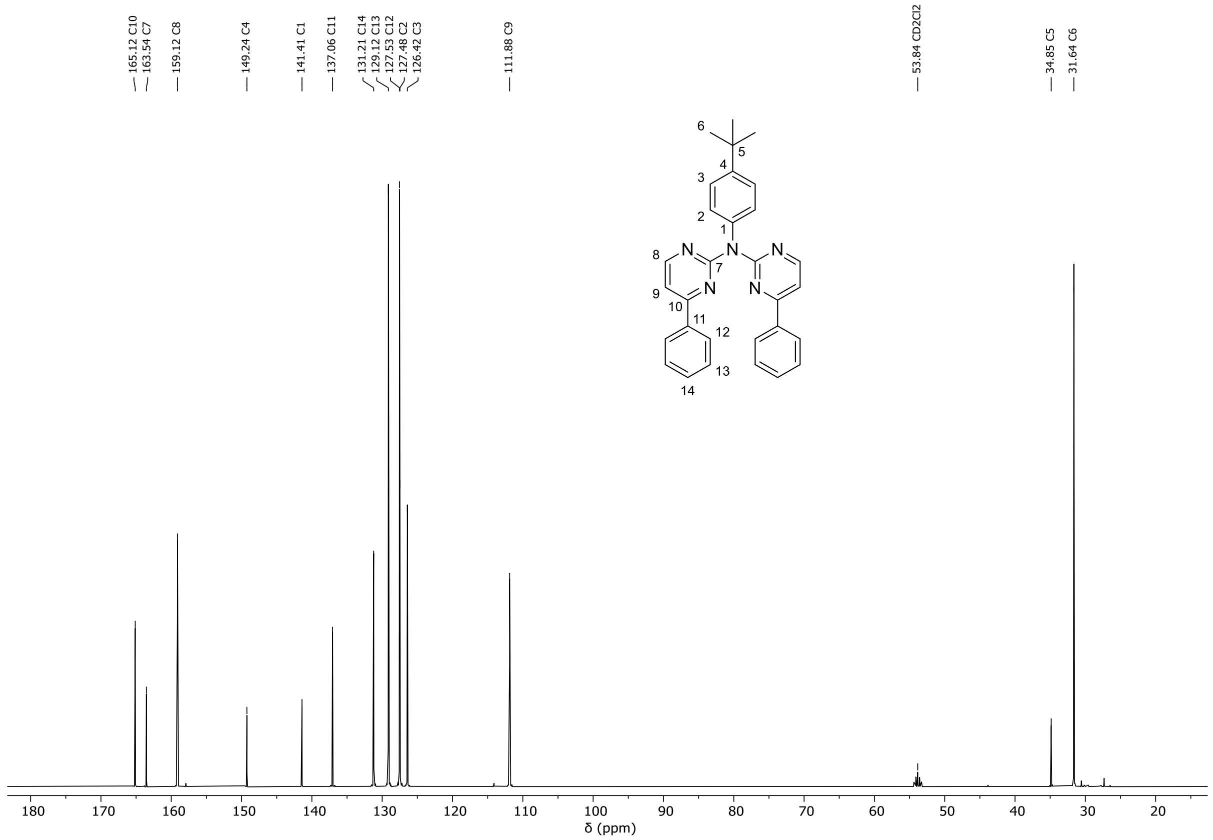

Figure S7: $^{13}C\{^{1}H\}$-NMR spectrum (101 MHz, CD_2Cl_2) of **LH2**.

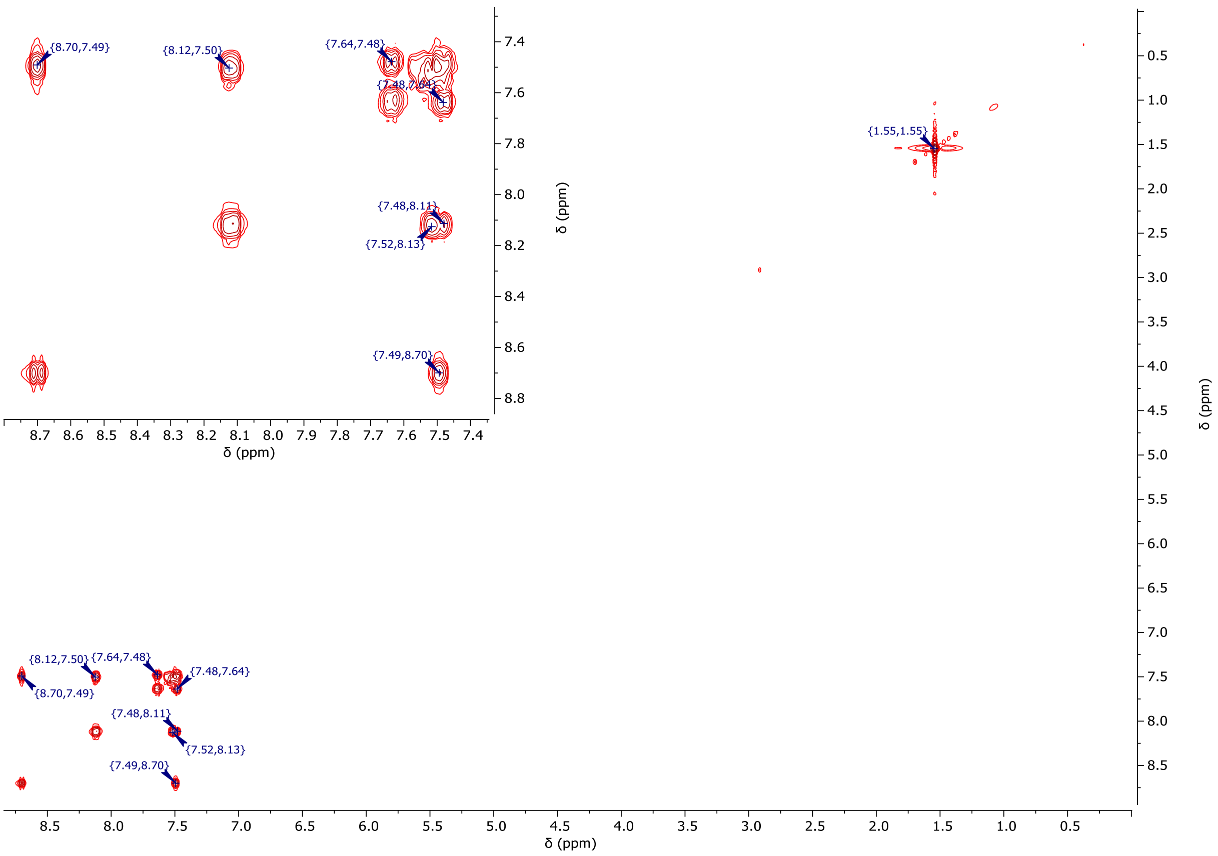

Figure S8: $^{1}H/^{1}H$-COSY-NMR spectrum (400 MHz/400 MHz, CD_2Cl_2) of **LH2**.

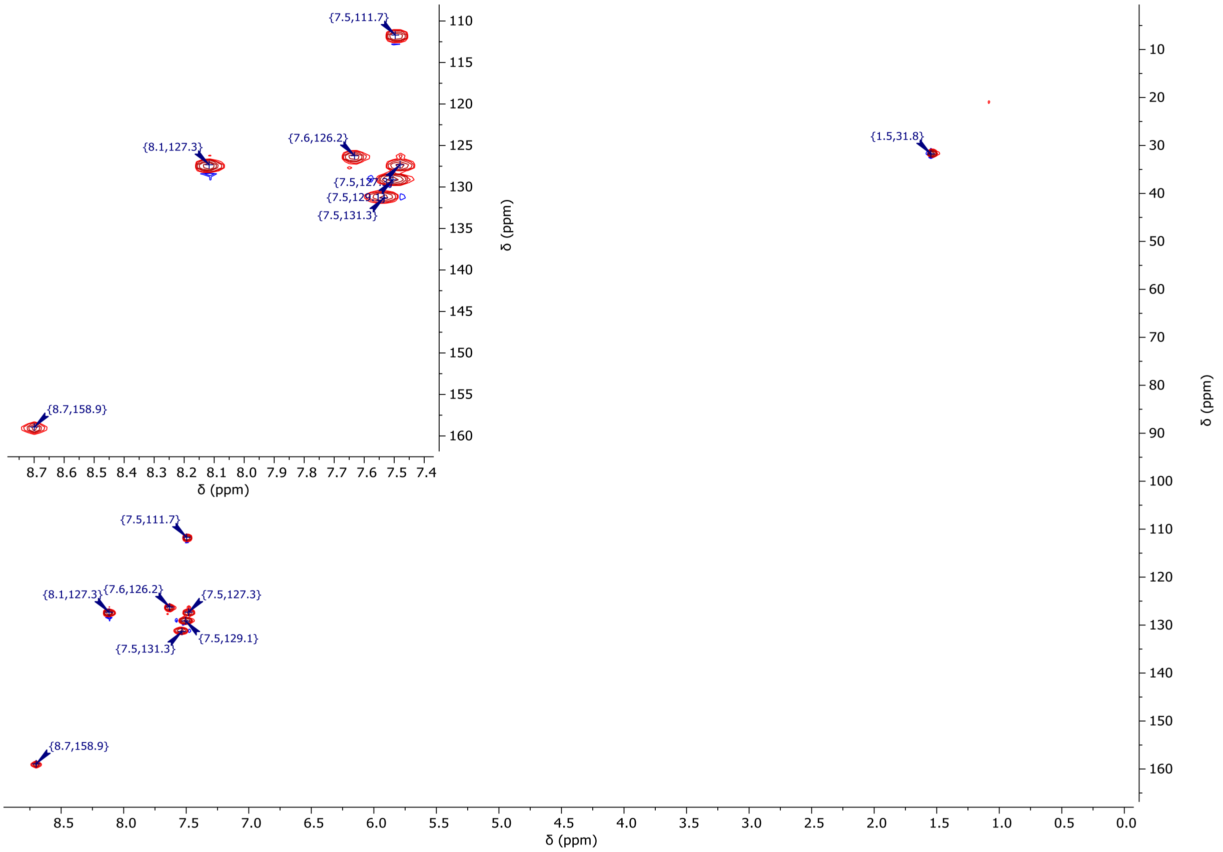

Figure S9: $^1H/^{13}C$-gHSQC-NMR spectrum (400 MHz/101 MHz, CD_2Cl_2) of **LH_2**.

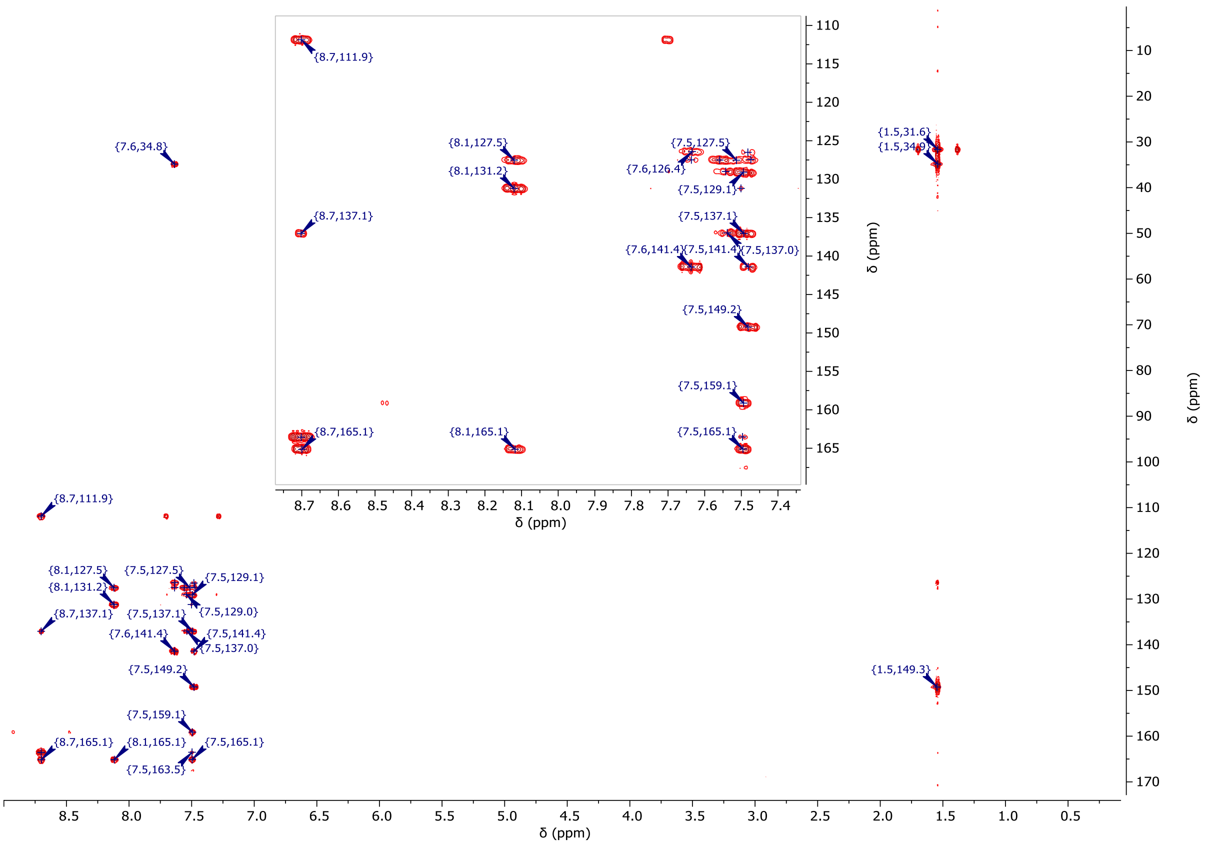

Figure S10: $^1H/^{13}C$-gHMBC-NMR spectrum (400 MHz/101 MHz, CD_2Cl_2) of **LH_2**.

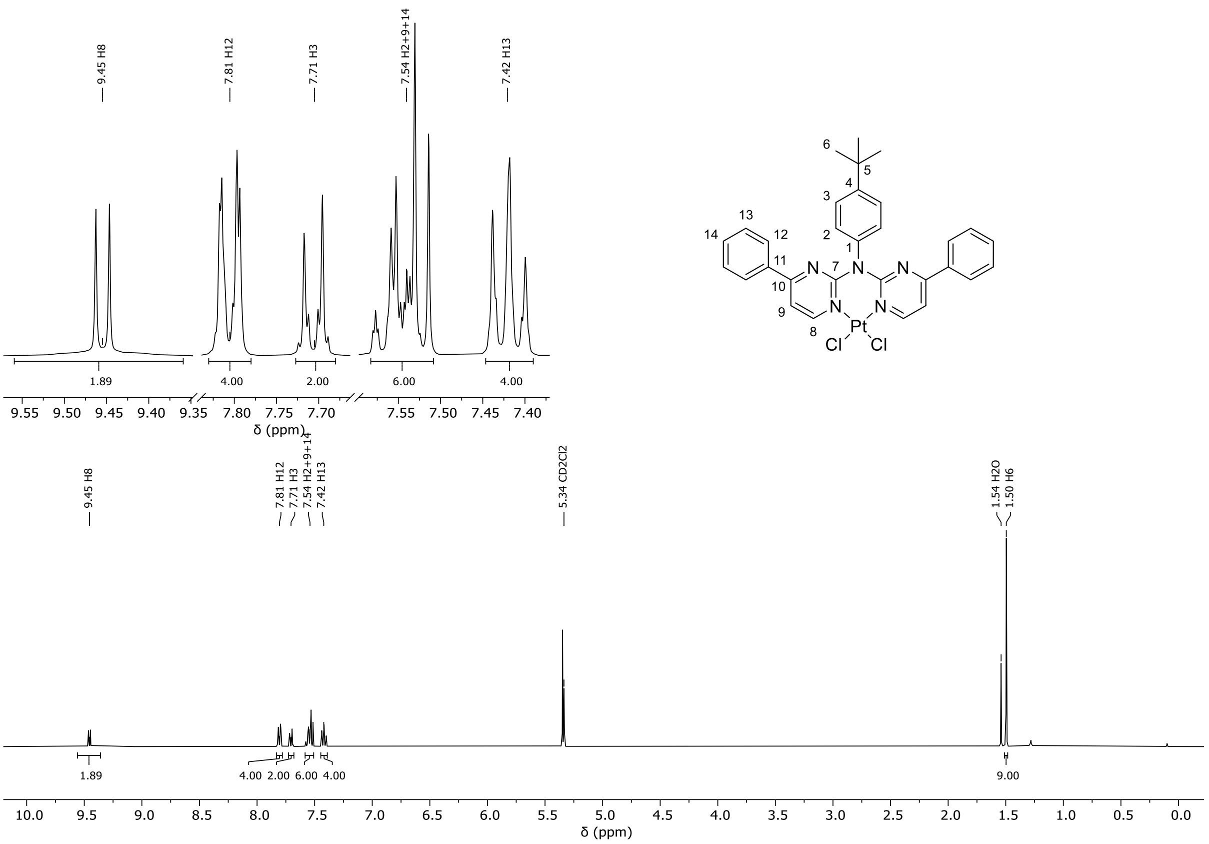

Figure S11: ^{1}H-NMR spectrum (400 MHz, CD_2Cl_2) of [**$PtLH_2Cl_2$**].

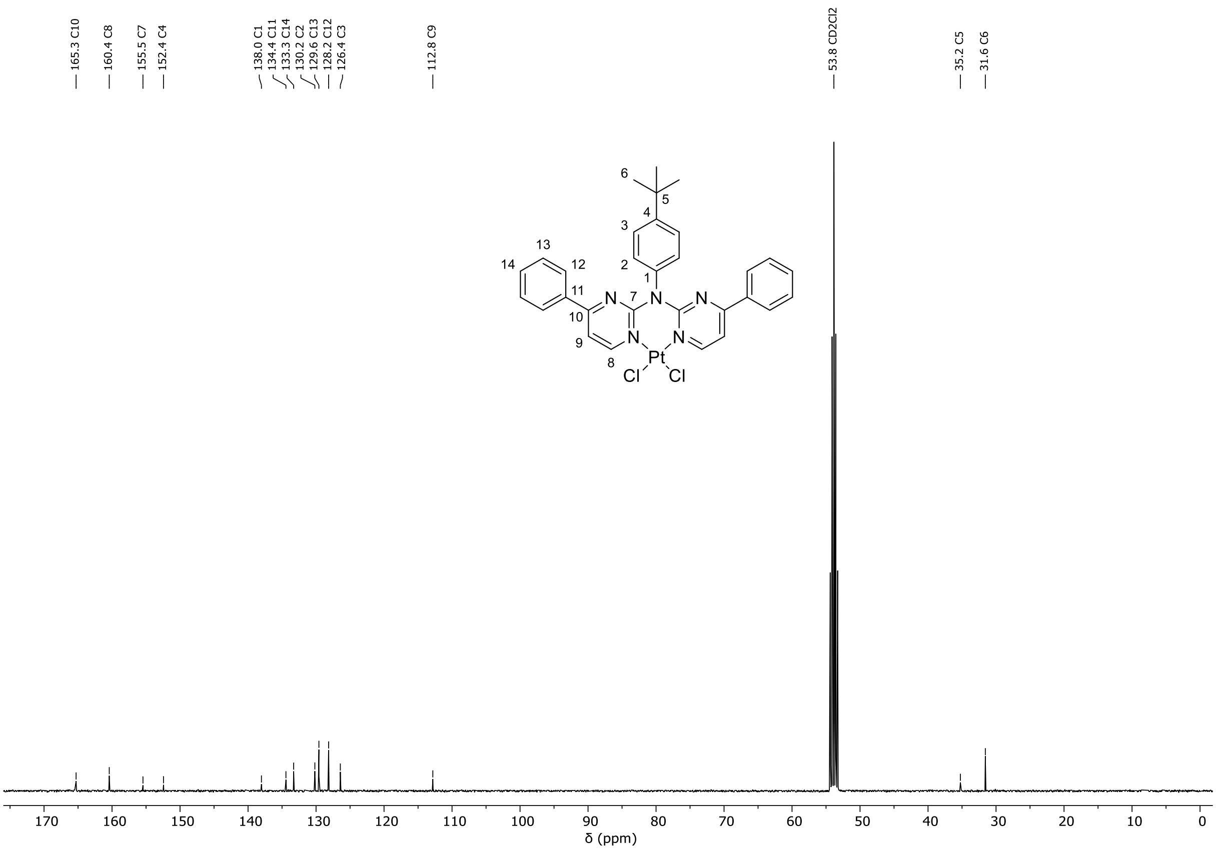

Figure S12: $^{13}C\{^{1}H\}$-NMR spectrum (101 MHz, CD_2Cl_2) of [**$PtLH_2Cl_2$**].

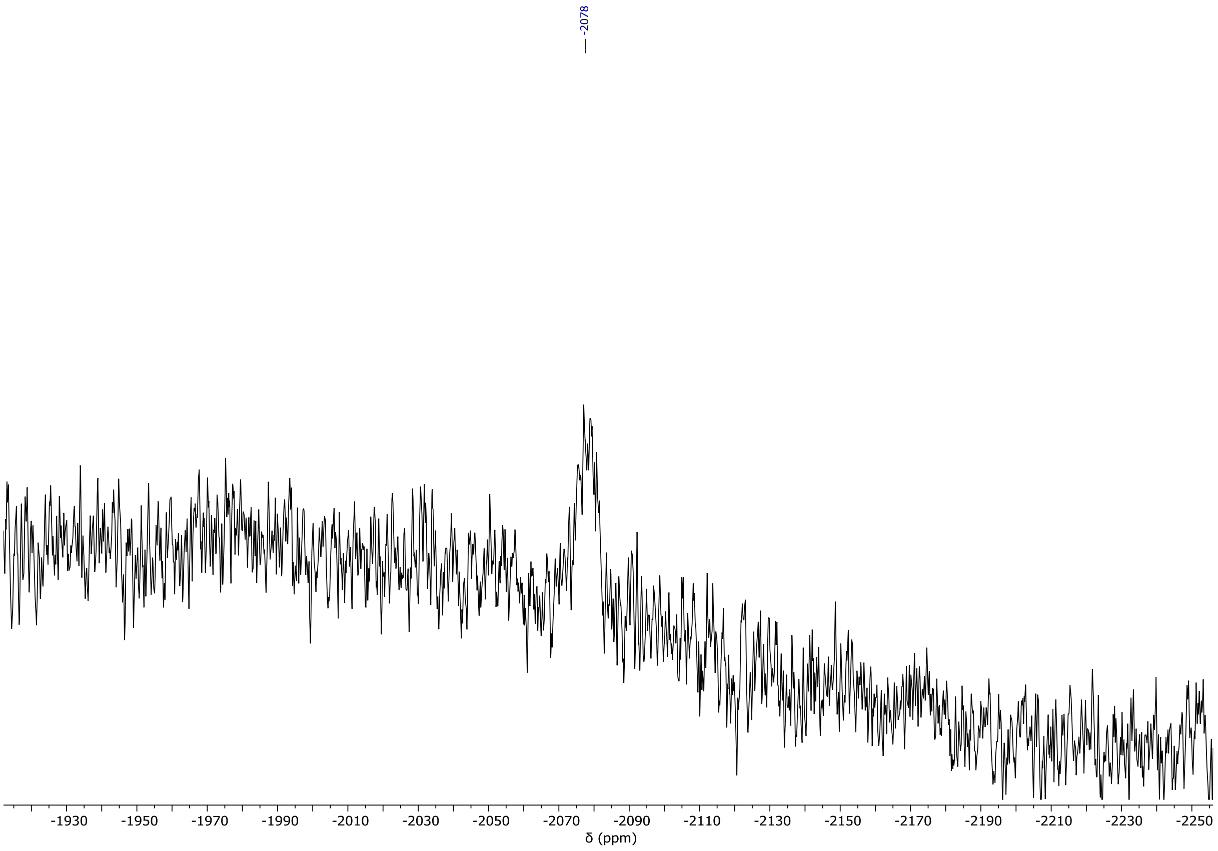

Figure S13: $^{195}Pt\{^{1}H\}$-NMR spectrum (86 MHz, CD_2Cl_2) of [**$PtLH_2Cl_2$**].

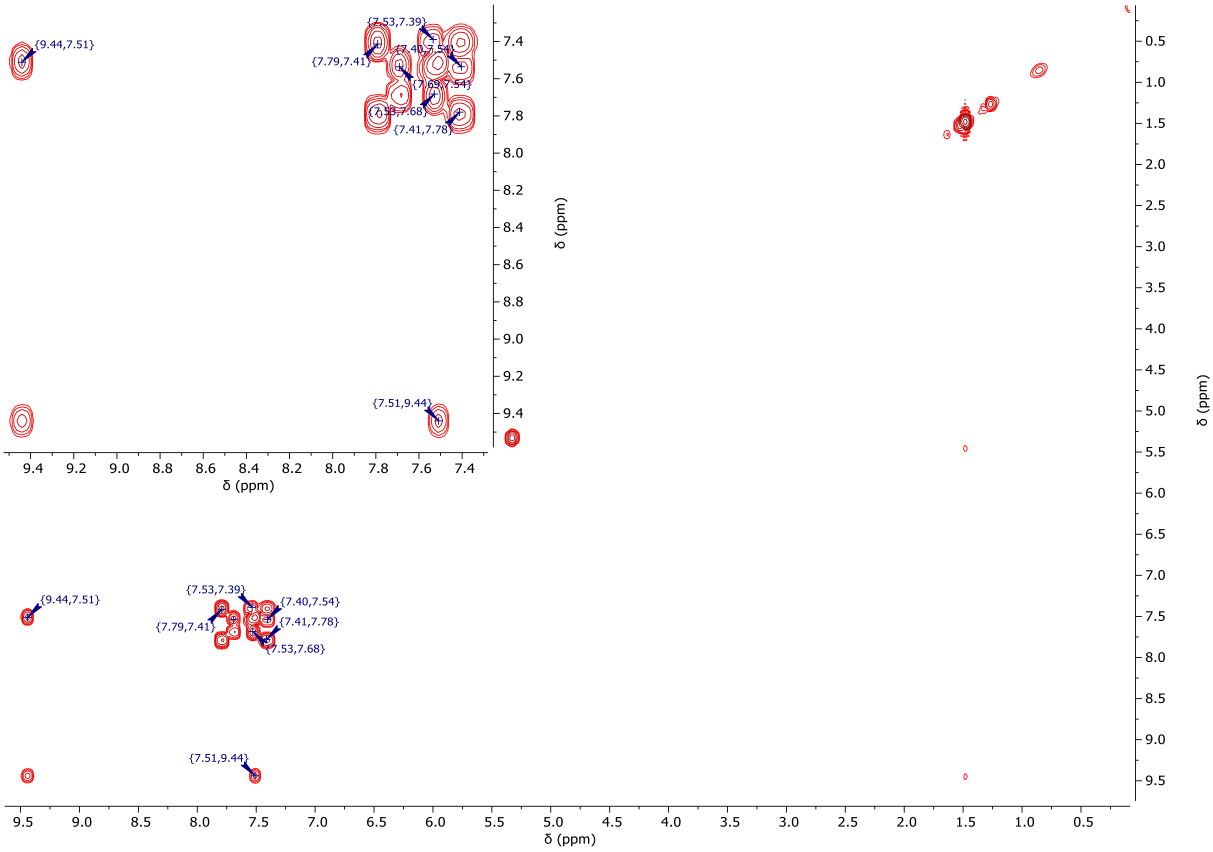

Figure S14: $^{1}H/^{1}H$-COSY-NMR spectrum (400 MHz/400 MHz, CD_2Cl_2) of [**$PtLH_2Cl_2$**].

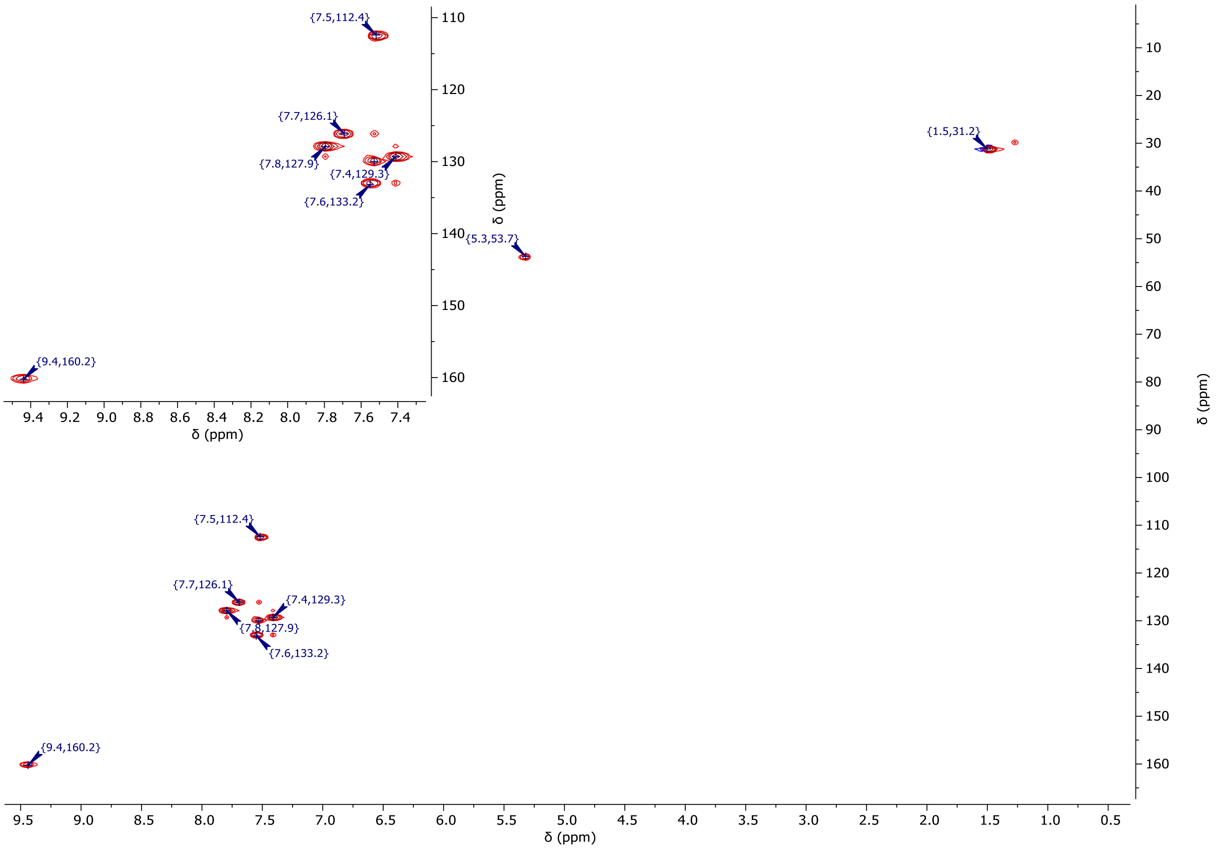

Figure S15: $^{1}H/^{13}C$-gHSQC-NMR spectrum (400 MHz/101 MHz, CD_2Cl_2) of [**$PtLH_2Cl_2$**].

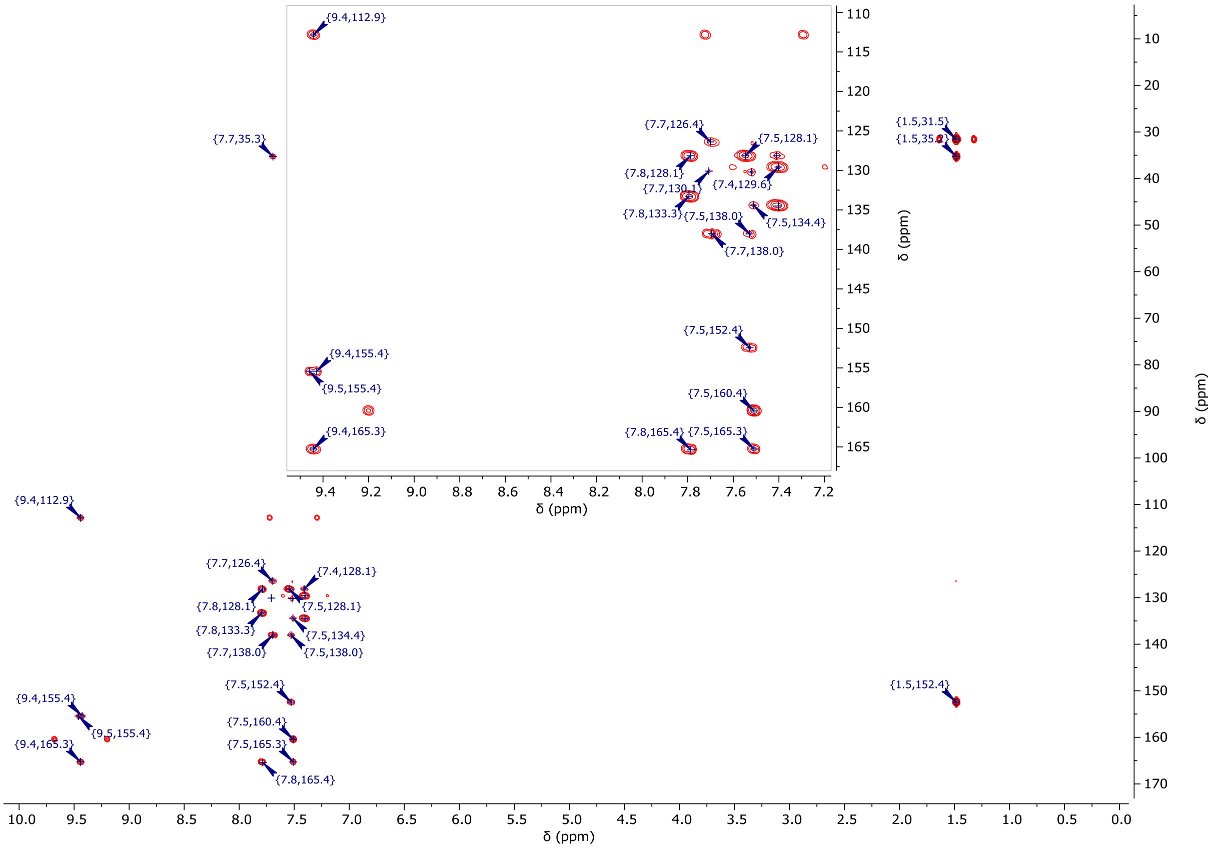

Figure S16: $^{1}H/^{13}C$-gHMBC-NMR spectrum (400 MHz/101 MHz, CD_2Cl_2) of [**$PtLH_2Cl_2$**].

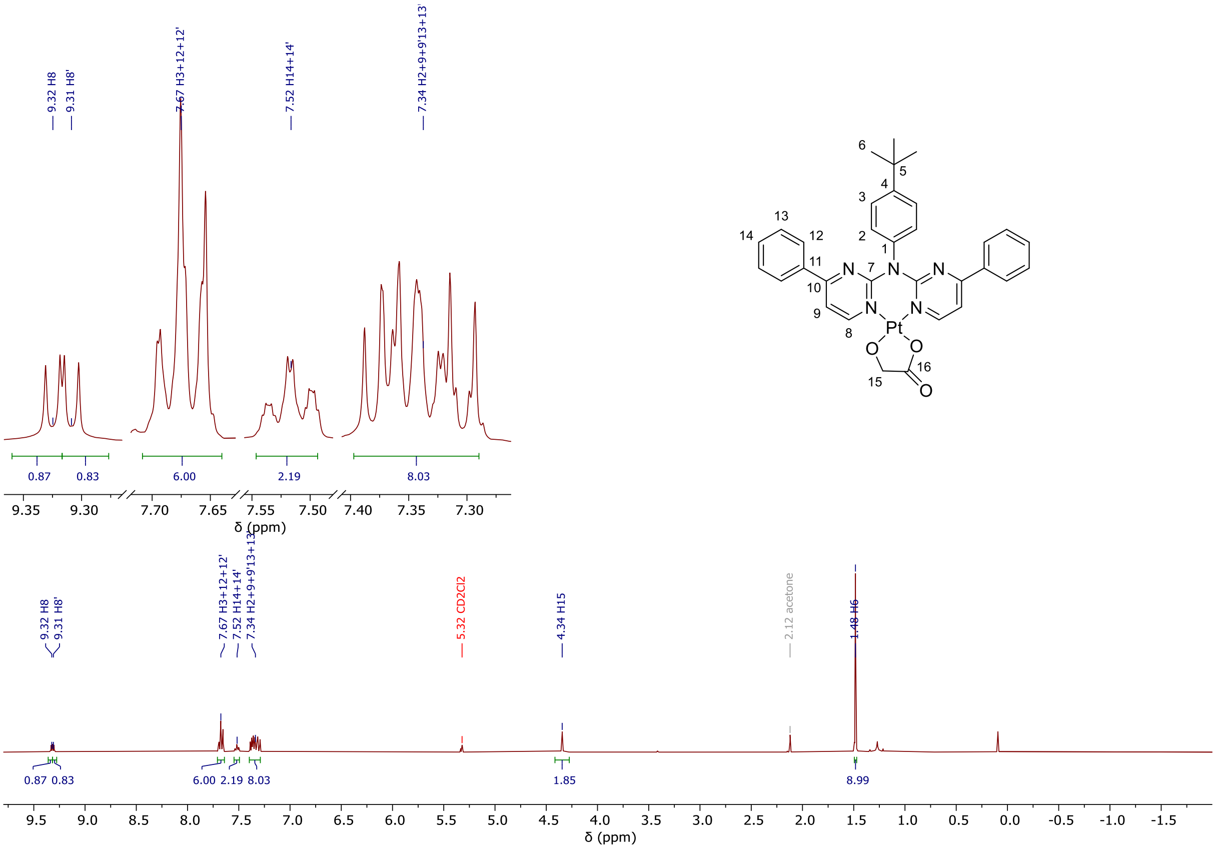

Figure S17: ^{1}H-NMR spectrum (400 MHz, CD_2Cl_2) of [**PtLH$_2$Gly**].

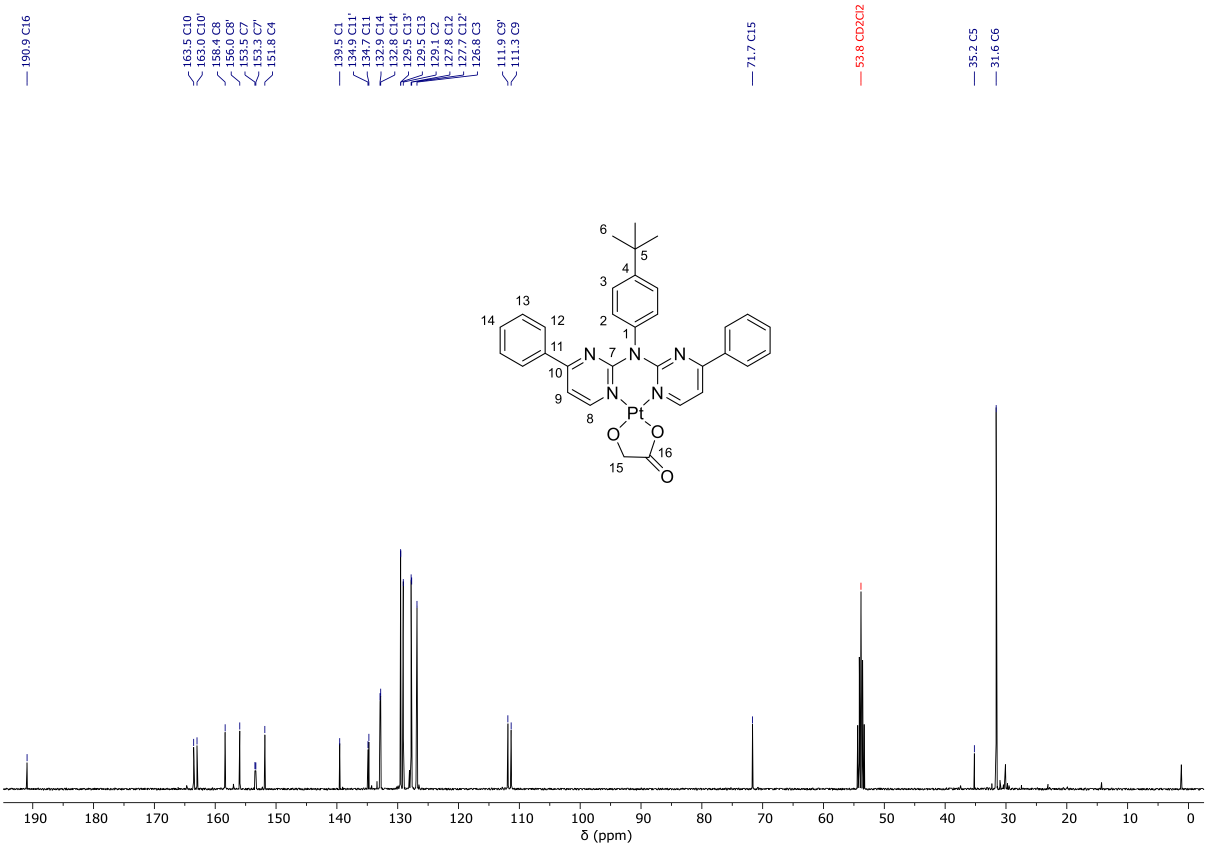

Figure S18: ^{13}C{^{1}H}-NMR spectrum (101 MHz, CD_2Cl_2) of [**PtLH$_2$Gly**].

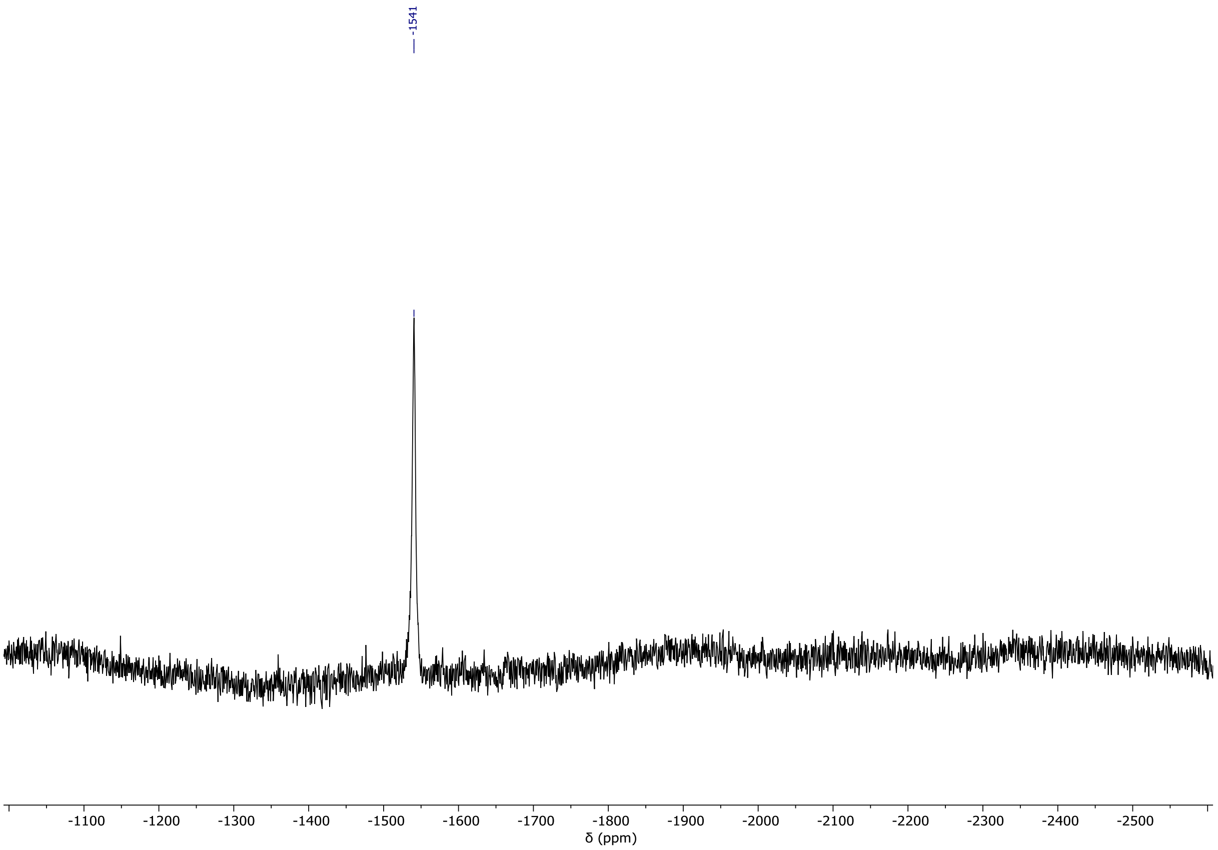

Figure S19: ^{195}Pt{^{1}H}-NMR spectrum (86 MHz, CD_2Cl_2) of [**PtLH$_2$Gly**].

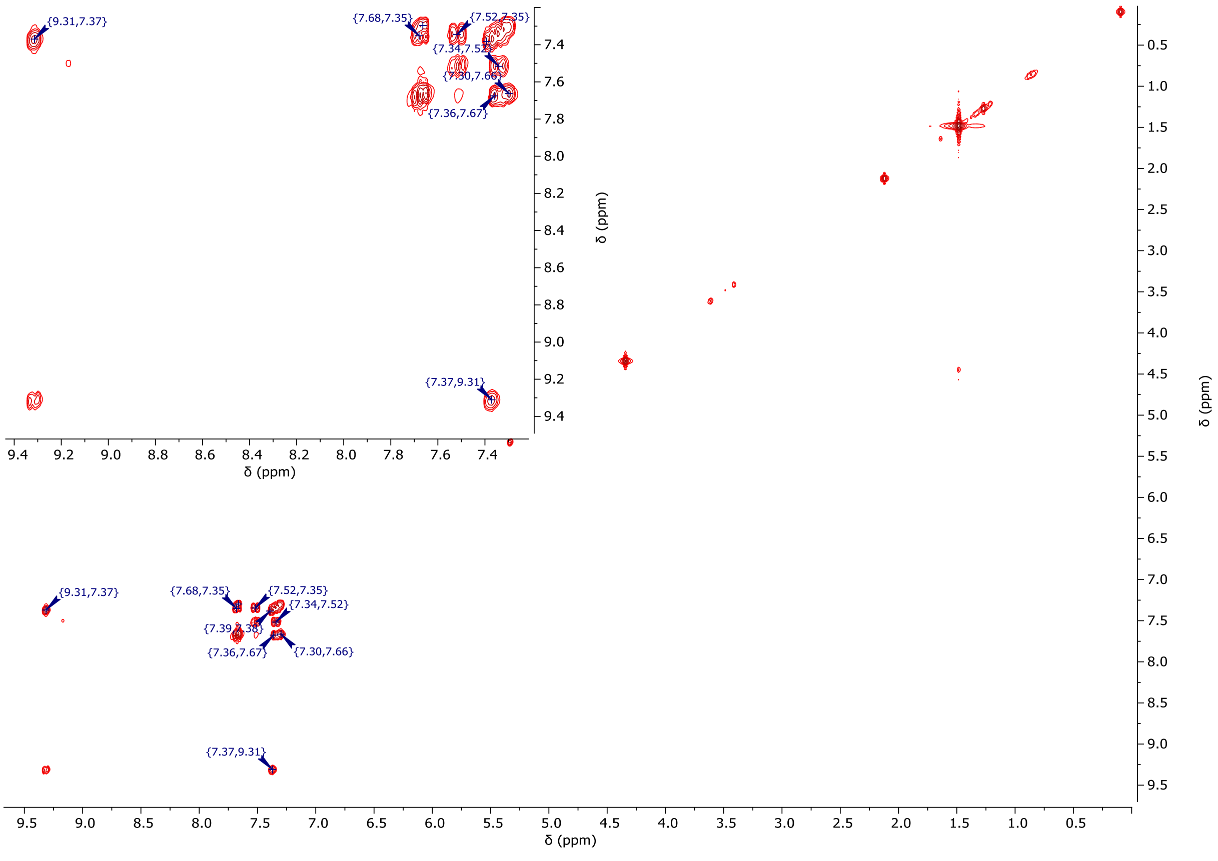

Figure S20: ^{1}H/^{1}H-COSY-NMR spectrum (400 MHz/400 MHz, CD_2Cl_2) of [**PtLH$_2$Gly**].

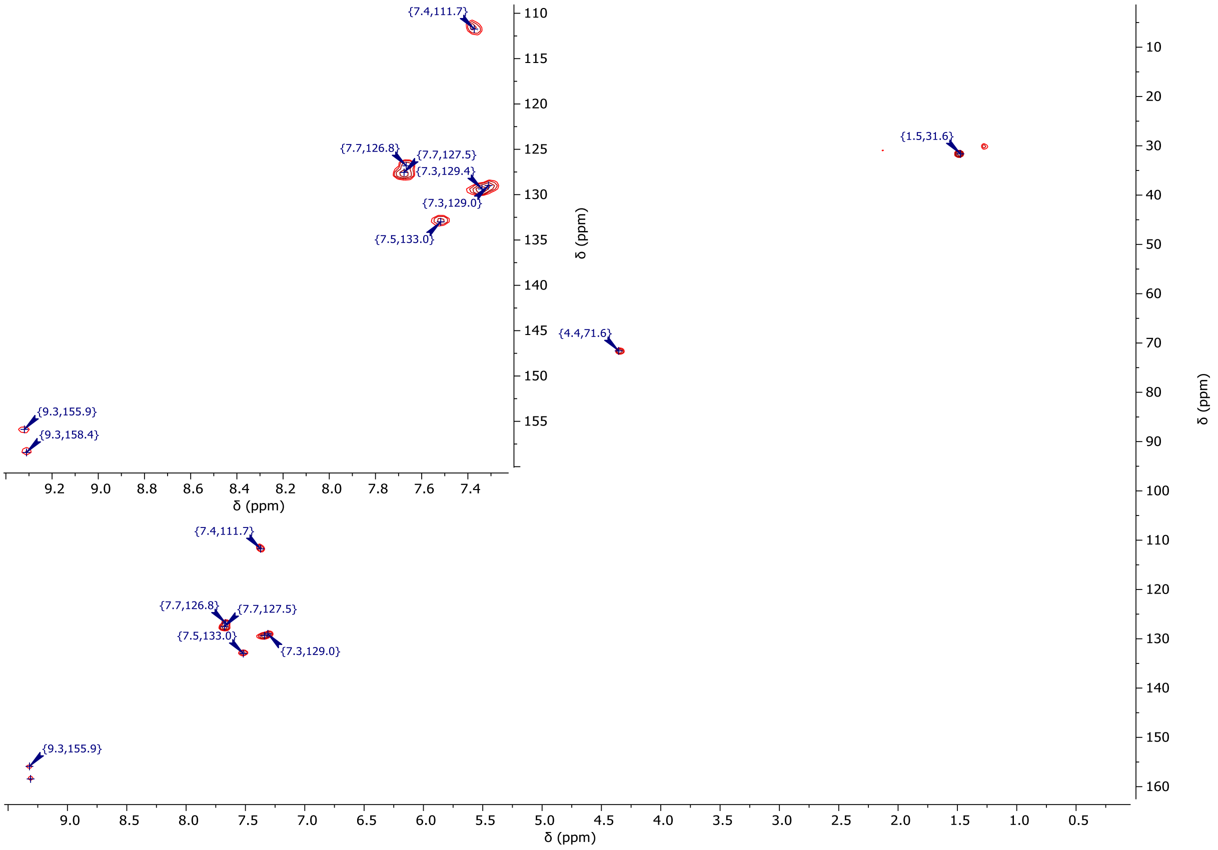

Figure S21: ^{1}H/^{13}C-gHSQC-NMR spectrum (400 MHz/101 MHz, CD_2Cl_2) of [**PtLH$_2$Gly**].

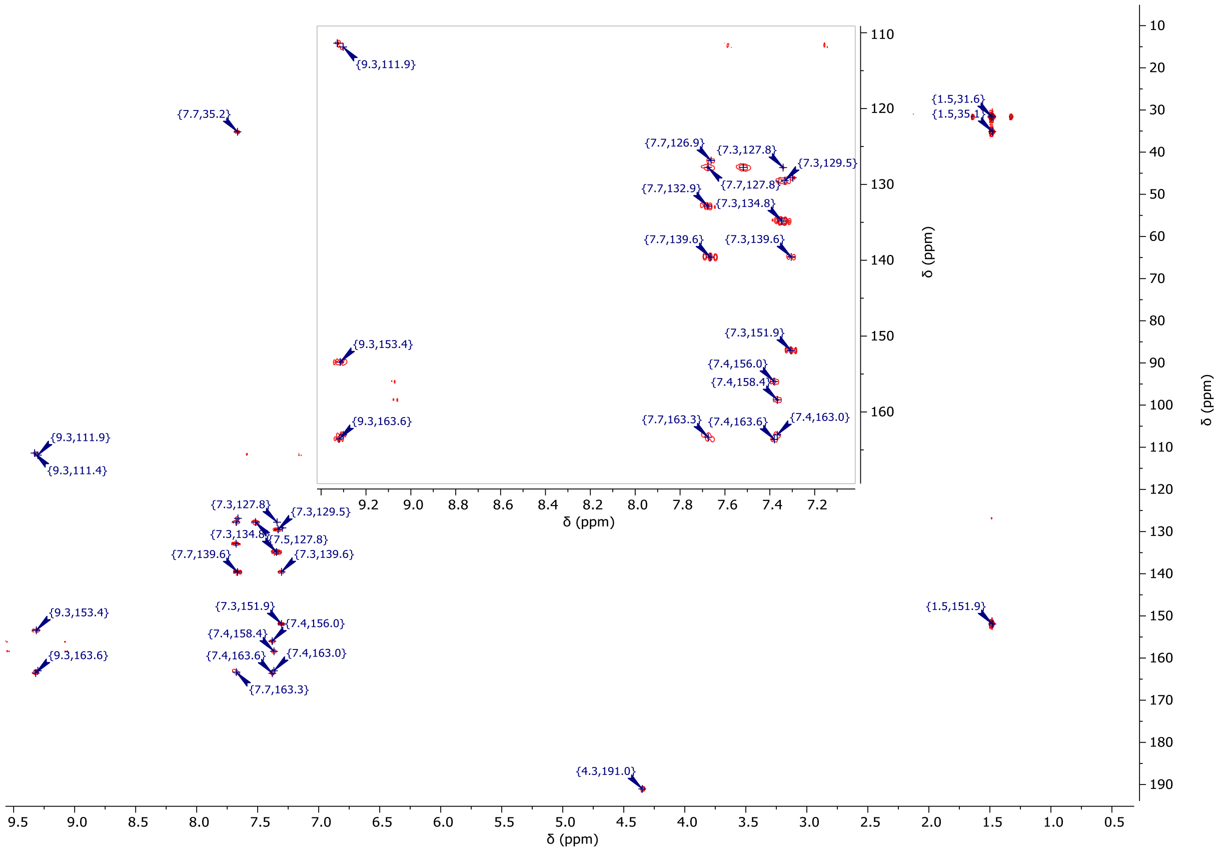

Figure S22: ^{1}H/^{13}C-gHMBC-NMR spectrum (400 MHz/101 MHz, CD_2Cl_2) of [**PtLH$_2$Gly**].

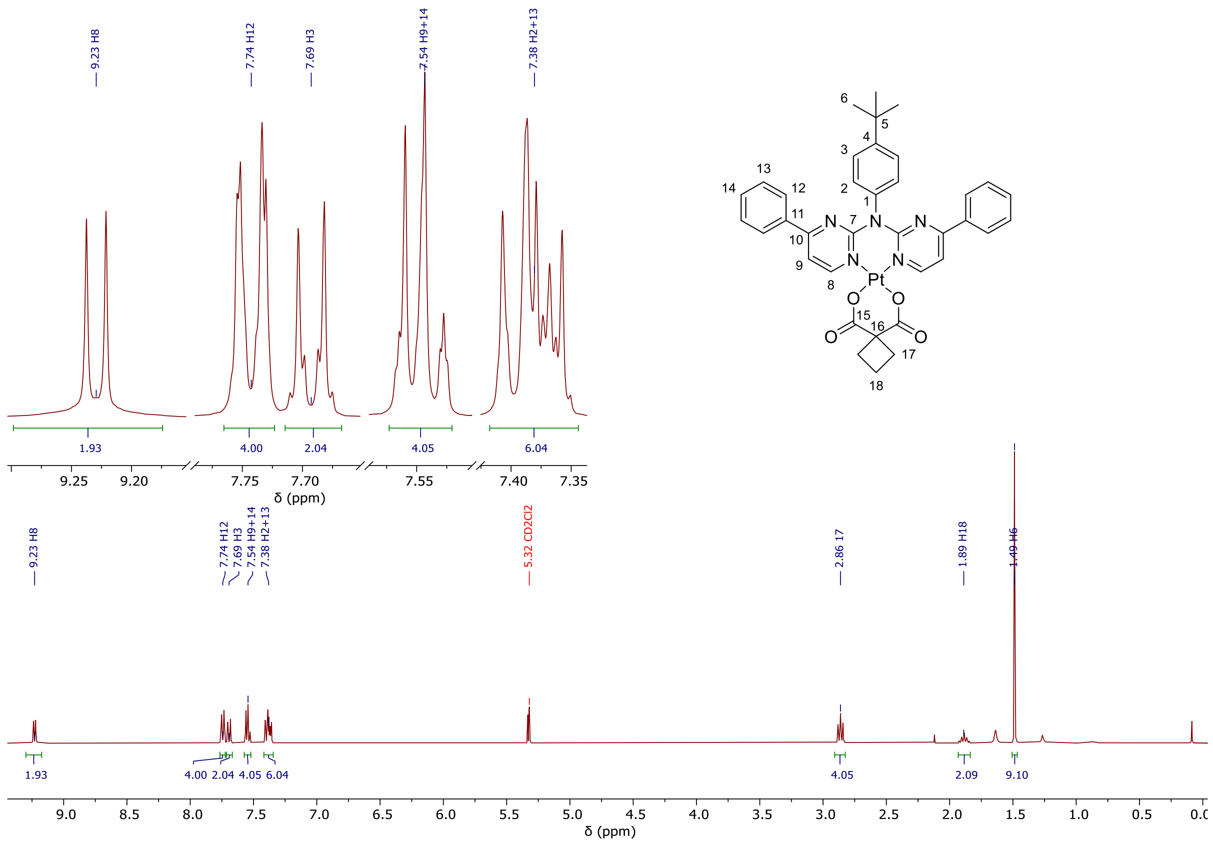

Figure S23: ^{1}H-NMR spectrum (400 MHz, CD_2Cl_2) of [**PtLH₂cbda**].

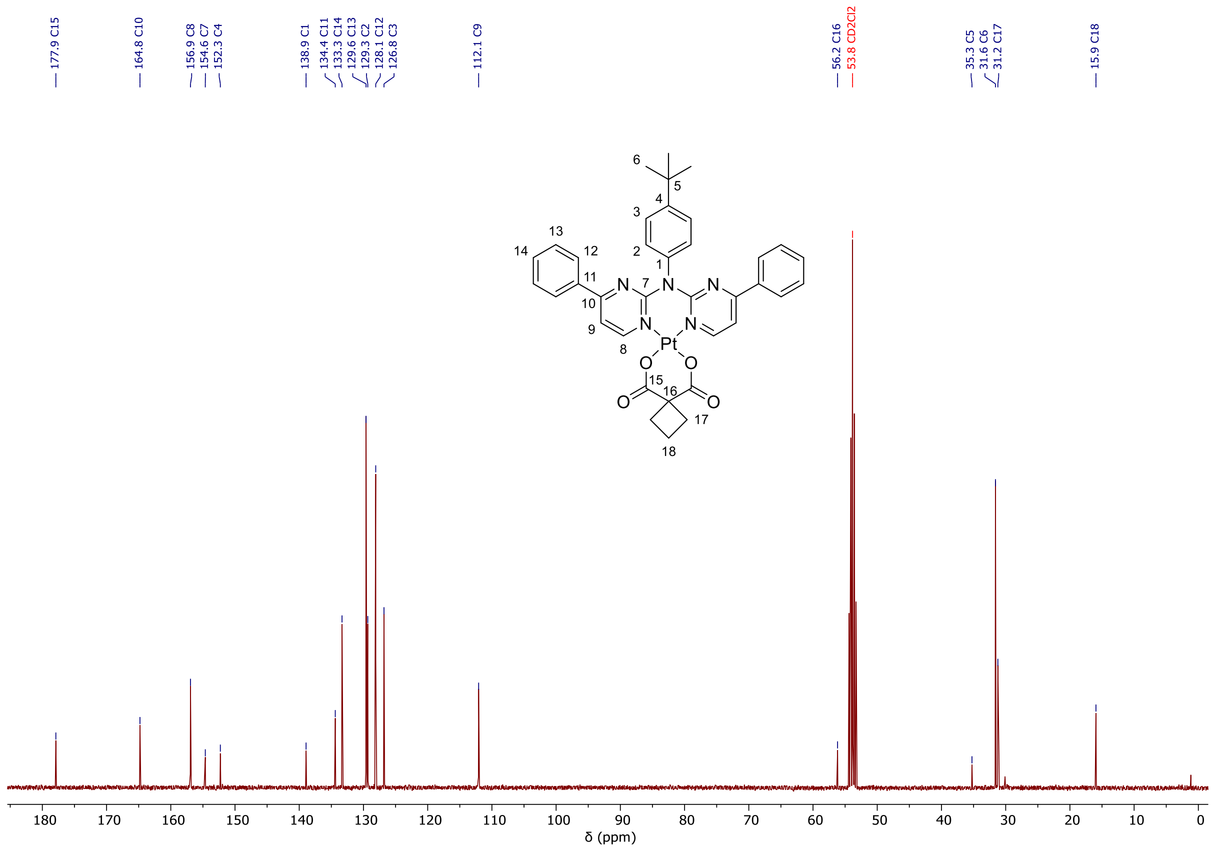

Figure S24: ^{13}C{^{1}H}-NMR spectrum (101 MHz, CD_2Cl_2) of [**PtLH$_2$cbda**].

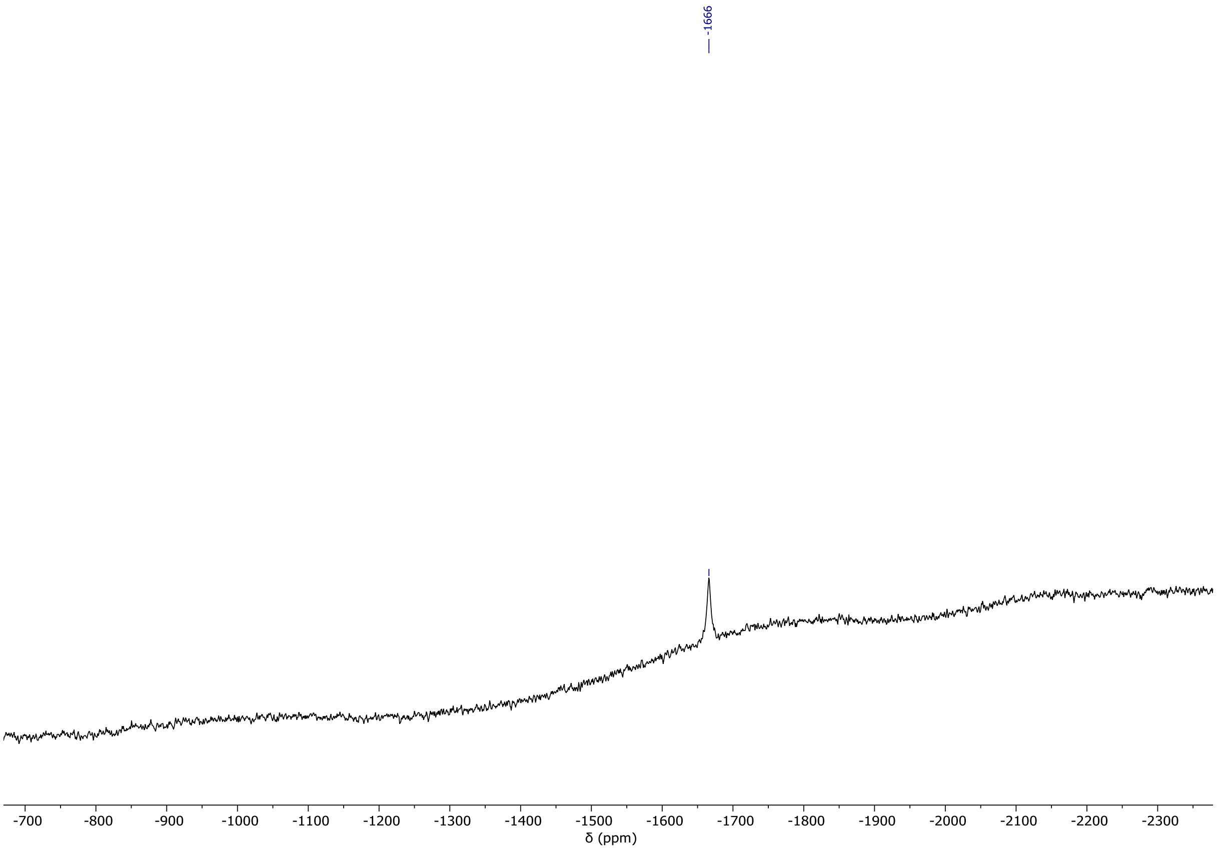

Figure S25: ^{195}Pt{^{1}H}-NMR spectrum (86 MHz, CD_2Cl_2) of [**PtLH$_2$cbda**].

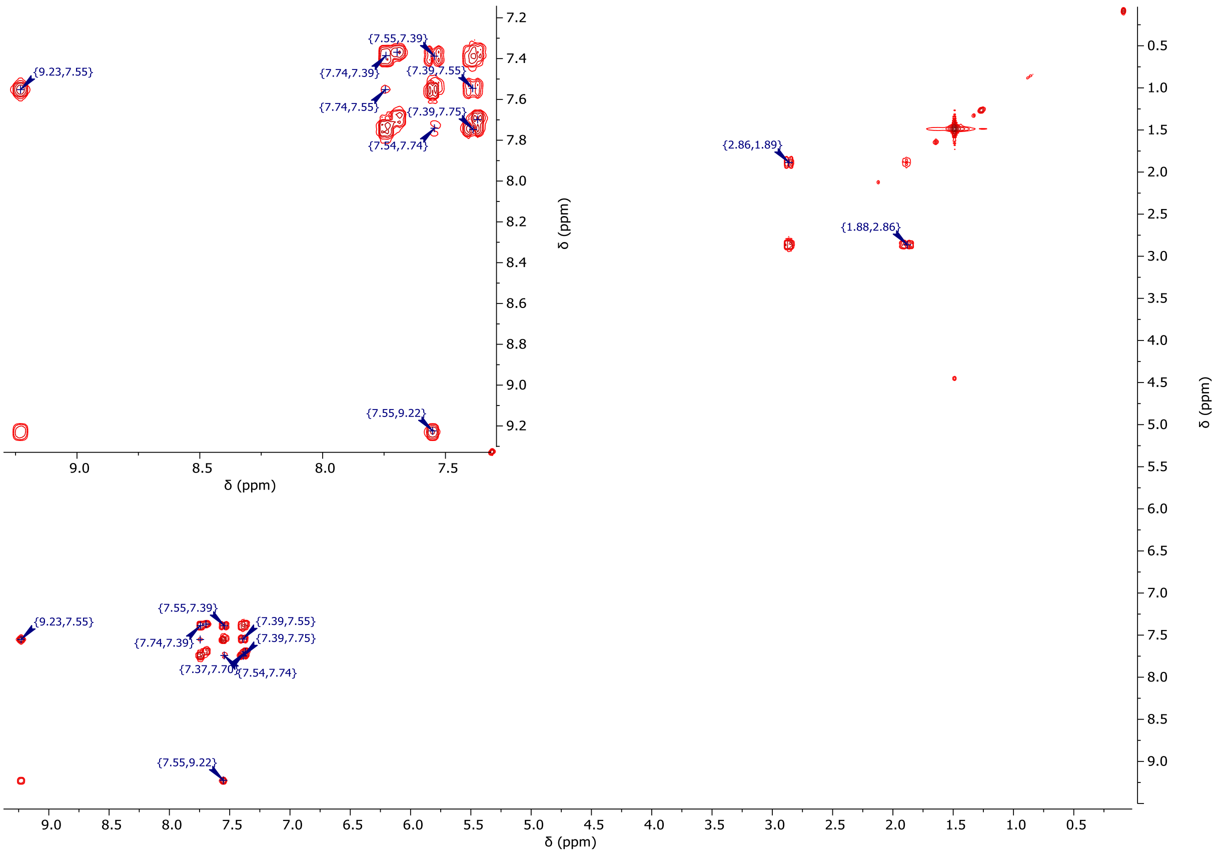

Figure S26: ^{1}H/^{1}H-COSY-NMR spectrum (400 MHz/400 MHz, CD_2Cl_2) of [**PtLH$_2$cbda**].

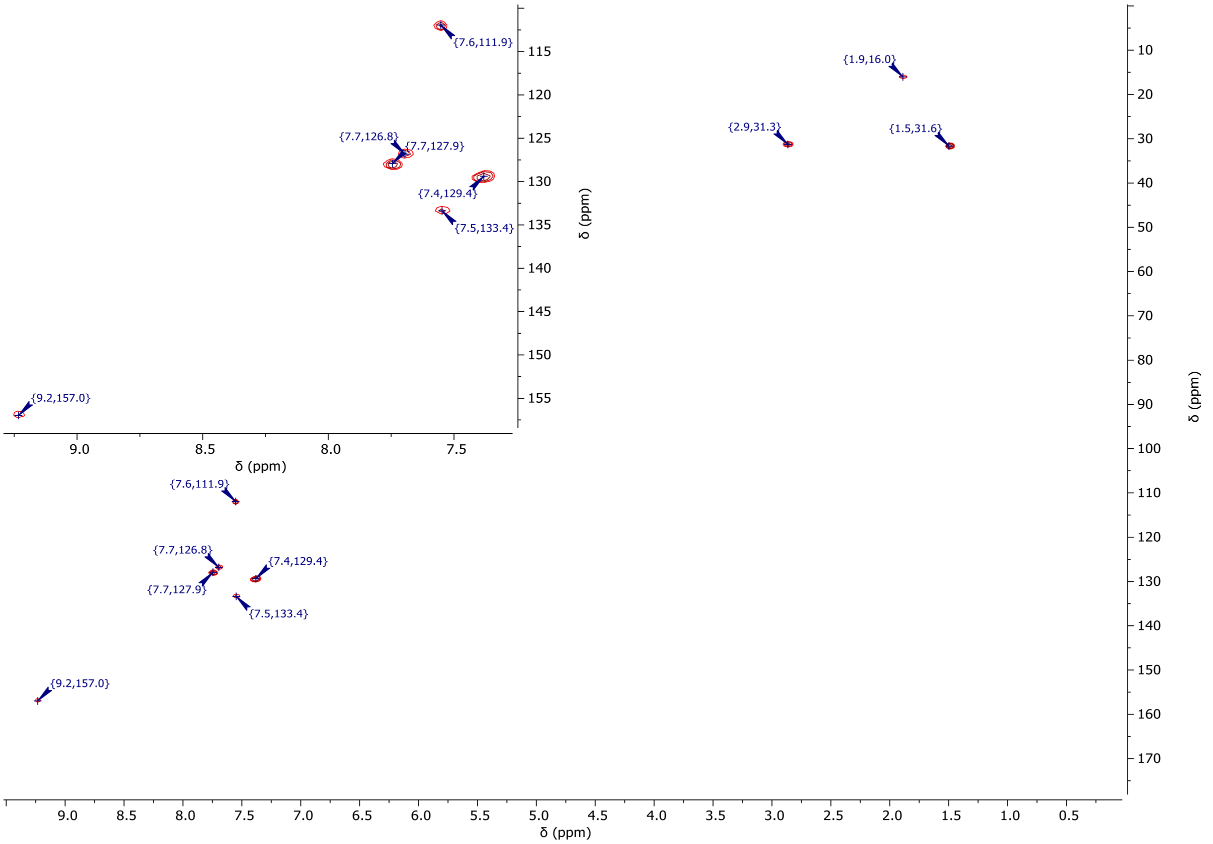

Figure S27: $^{1}H/^{13}C$-gHSQC-NMR spectrum (400 MHz/101 MHz, CD_2Cl_2) of [**PtLH$_2$cbda**].

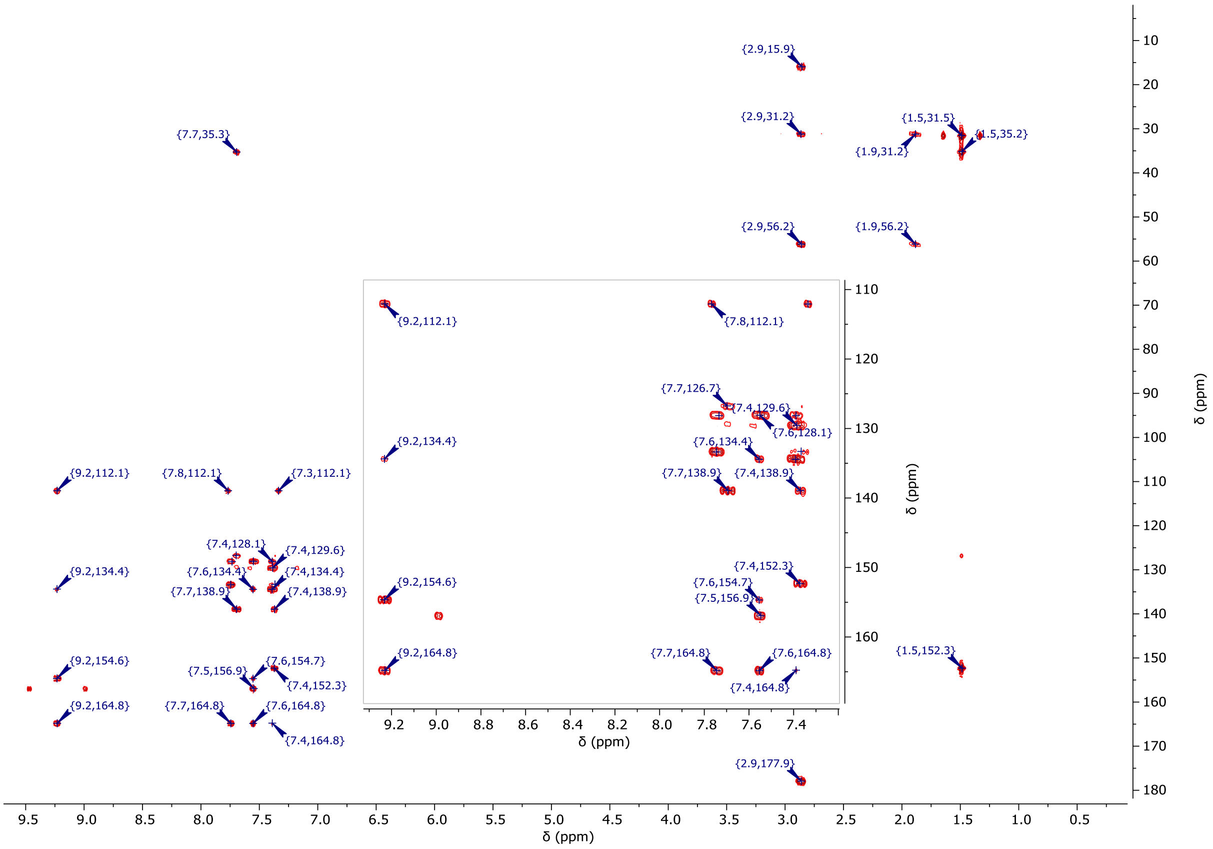

Figure S28: $^{1}H/^{13}C$-gHMBC-NMR spectrum (400 MHz/101 MHz, CD_2Cl_2) of [**PtLH$_2$cbda**].

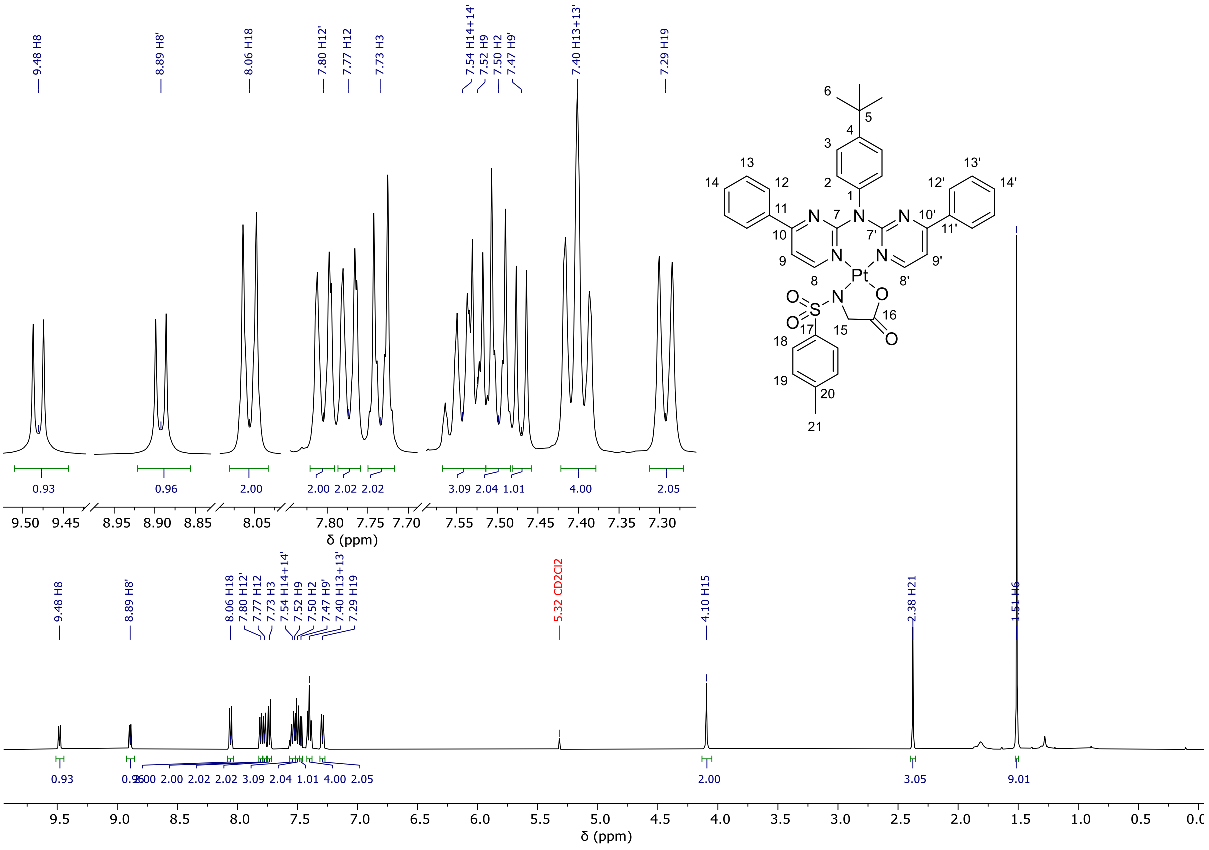

Figure S29: ^{1}H-NMR spectrum (500 MHz, CD_2Cl_2) of [**PtLH$_2$Tsgly**].

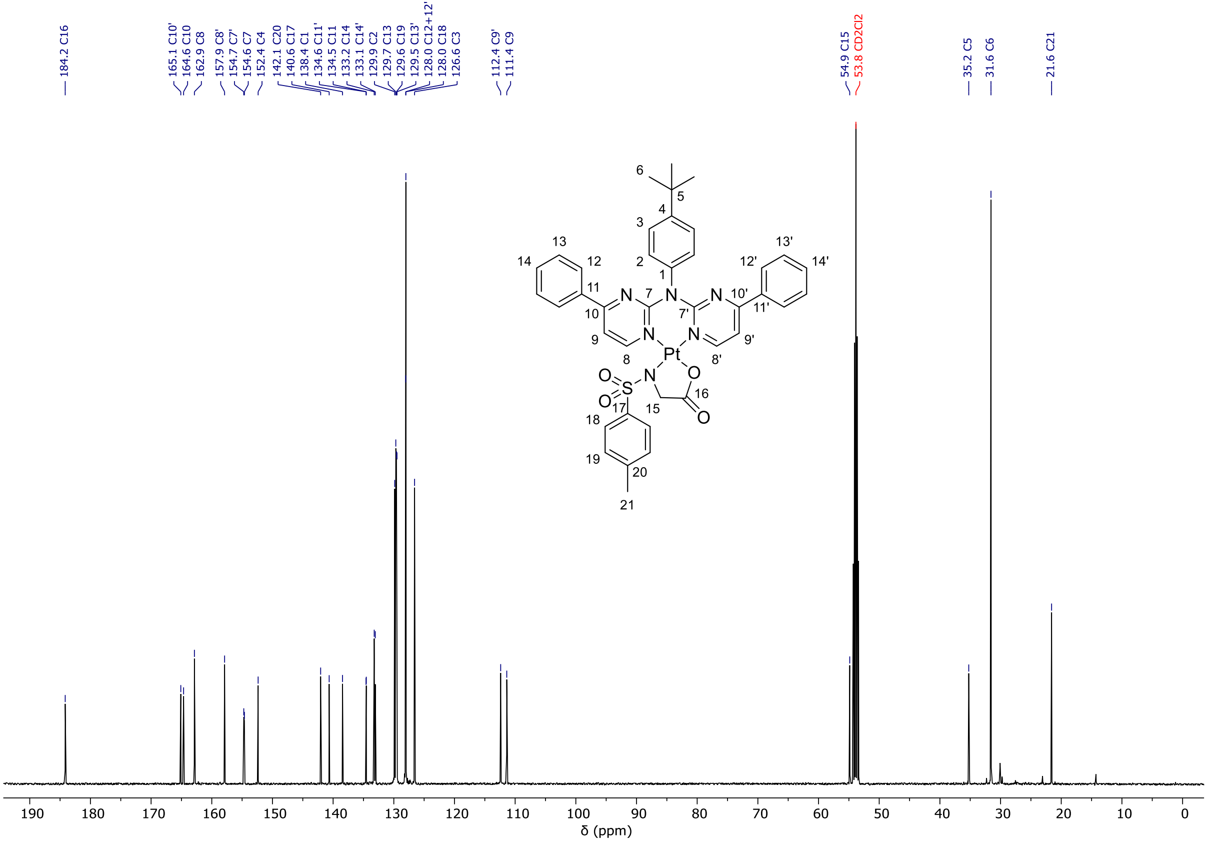

Figure S30: ^{13}C{^{1}H}-NMR spectrum (126 MHz, CD_2Cl_2) of [**PtLH$_2$Tsgly**].

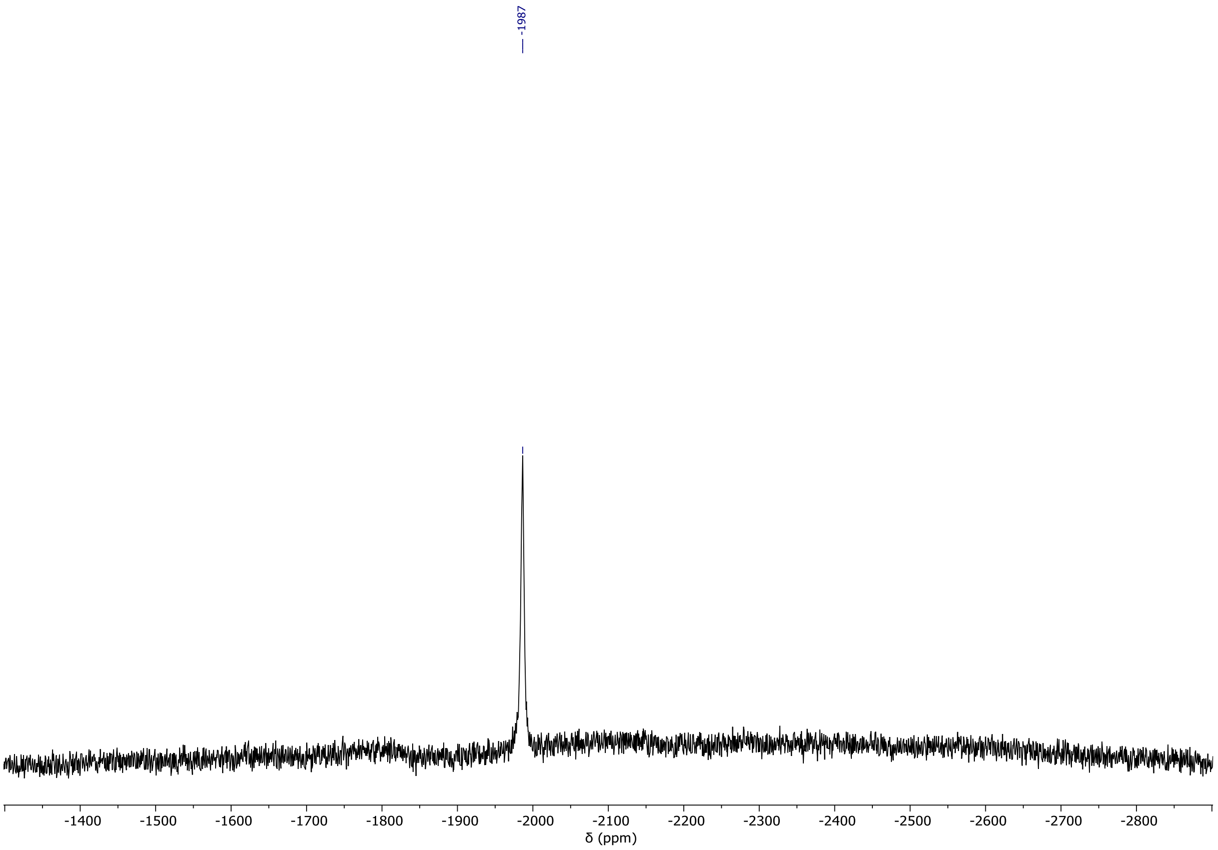

Figure S31: ^{195}Pt{^{1}H}-NMR spectrum (107 MHz, CD_2Cl_2) of [**PtLH$_2$Tsgly**].

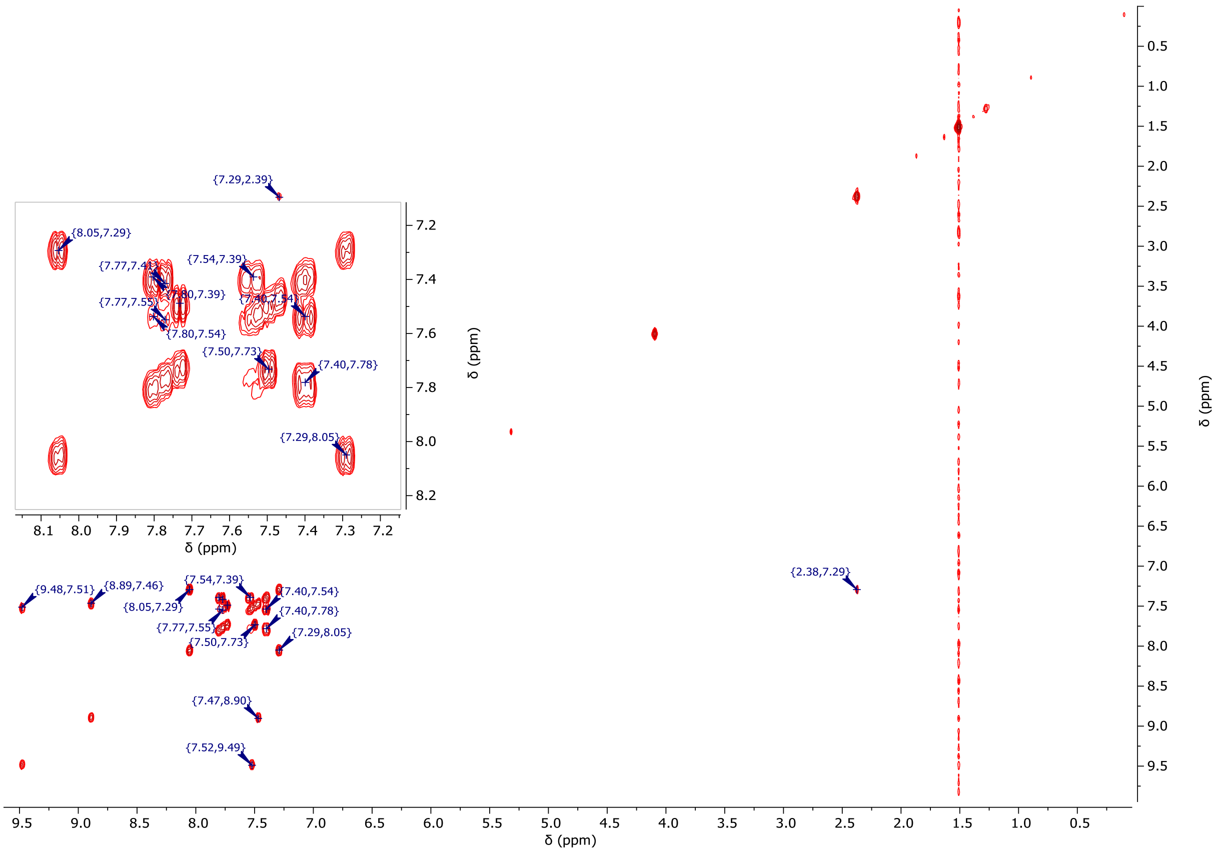

Figure S32: ^{1}H/^{1}H-COSY-NMR spectrum (500 MHz/500 MHz, CD_2Cl_2) of [**PtLH$_2$Tsgly**].

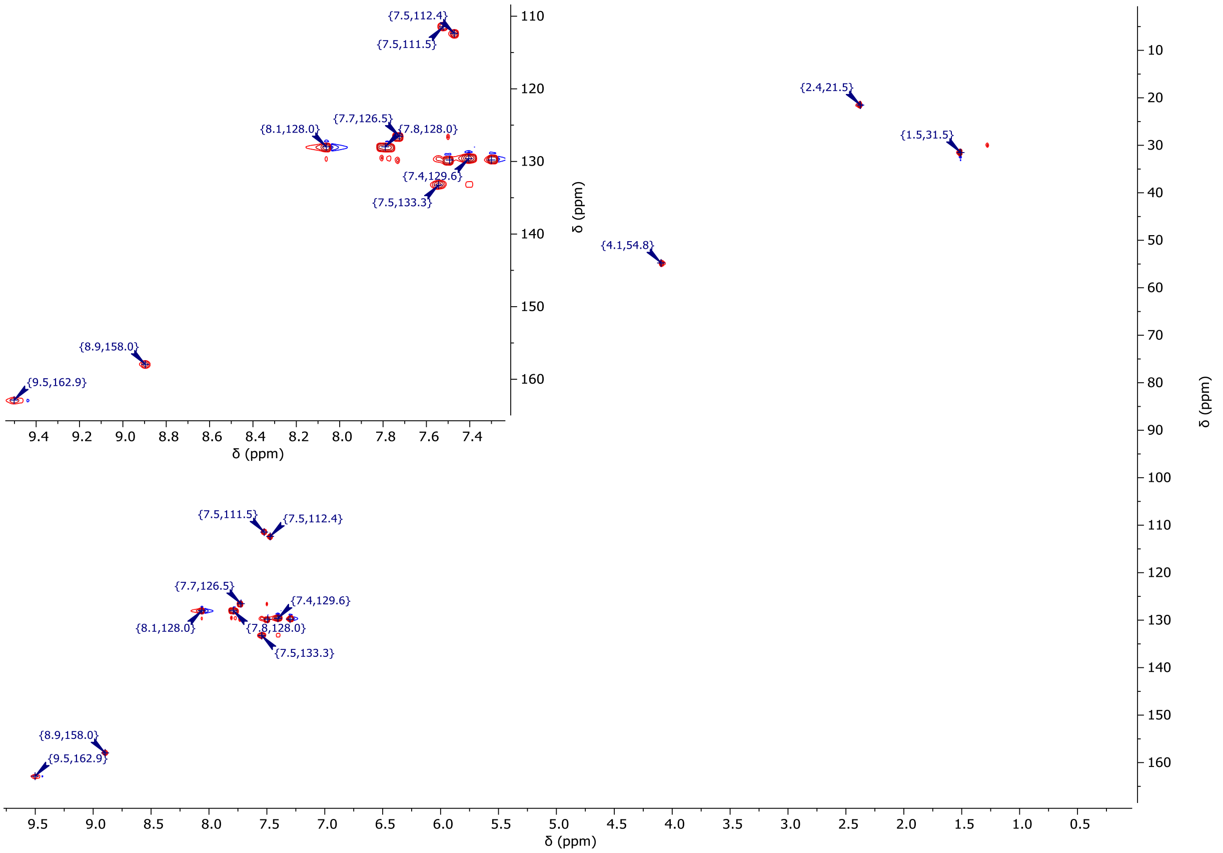

Figure S33: $^{1}H/^{13}C$-gHSQC-NMR spectrum (500 MHz/126 MHz, CD_2Cl_2) of [**PtLH₂Tsgly**].

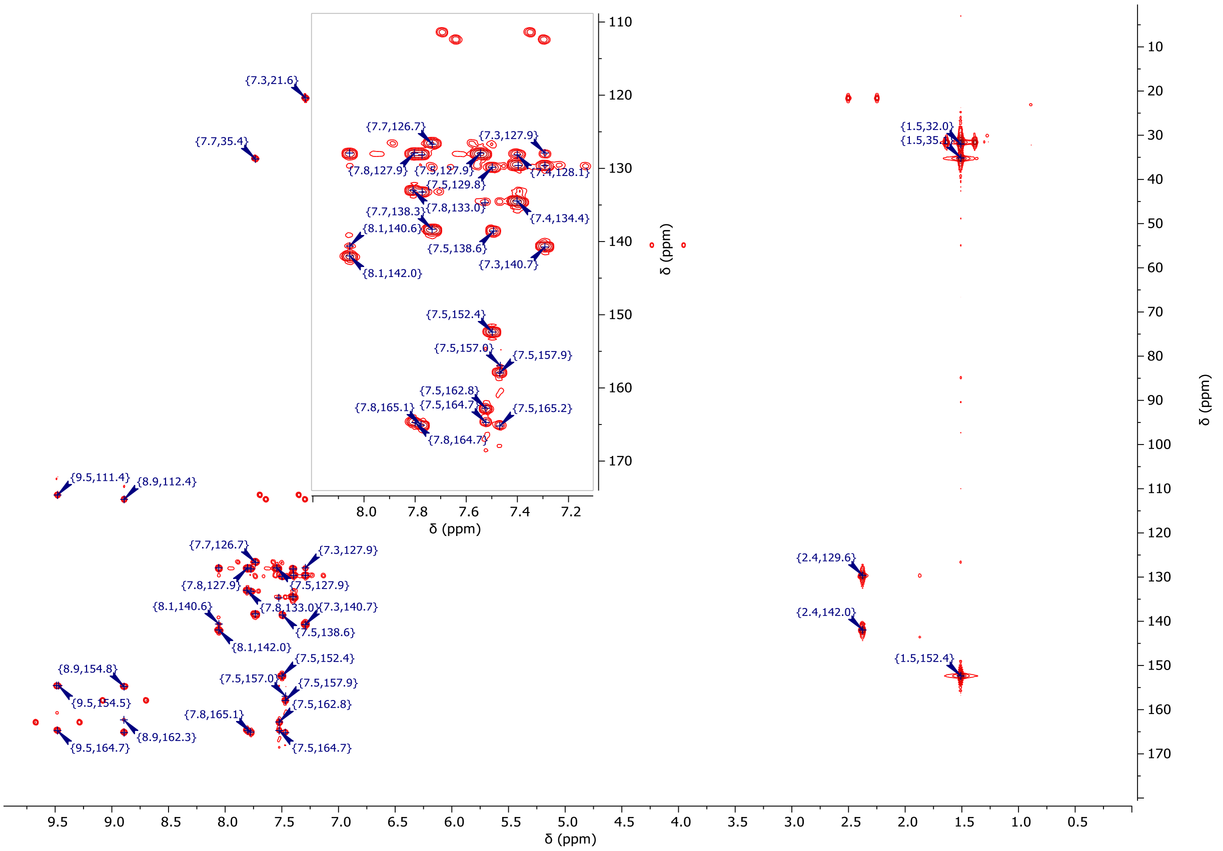

Figure S34: $^{1}H/^{13}C$-gHMBC-NMR spectrum (500 MHz/126 MHz, CD_2Cl_2) of [**PtLH₂Tsgly**].

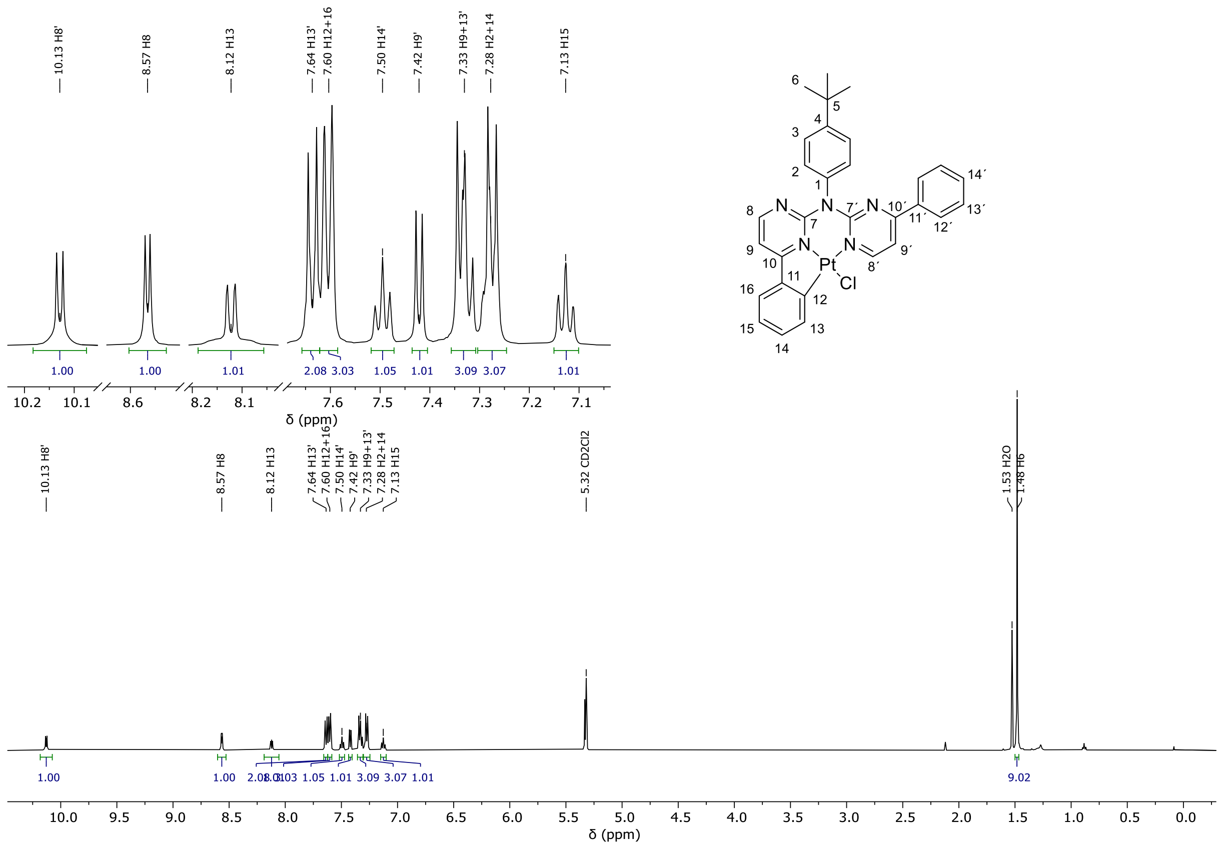

Figure S35: ^{1}H-NMR spectrum (500 MHz, CD_2Cl_2) of [**PtLHCl**].

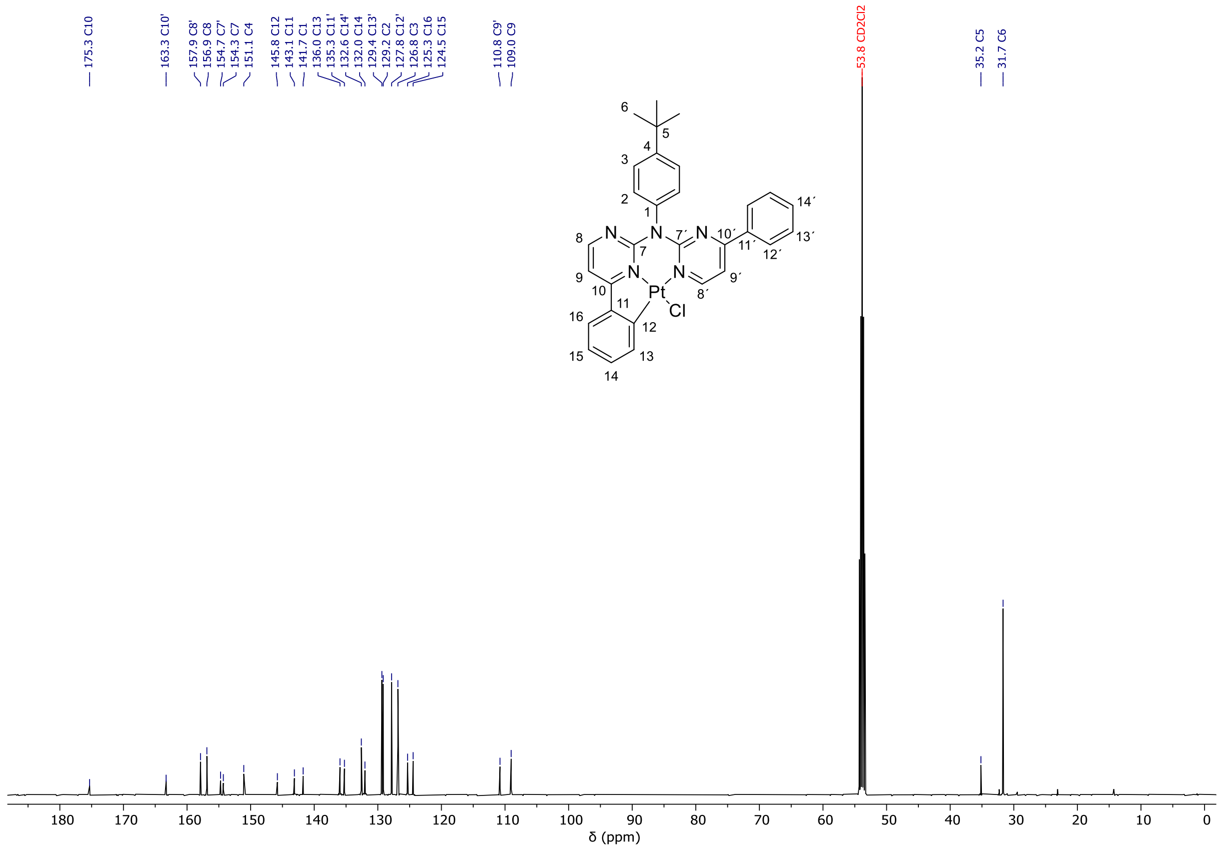

Figure S36: $^{13}C\{^1H\}$-NMR spectrum (126 MHz, CD_2Cl_2) of [**PtLHCl**].

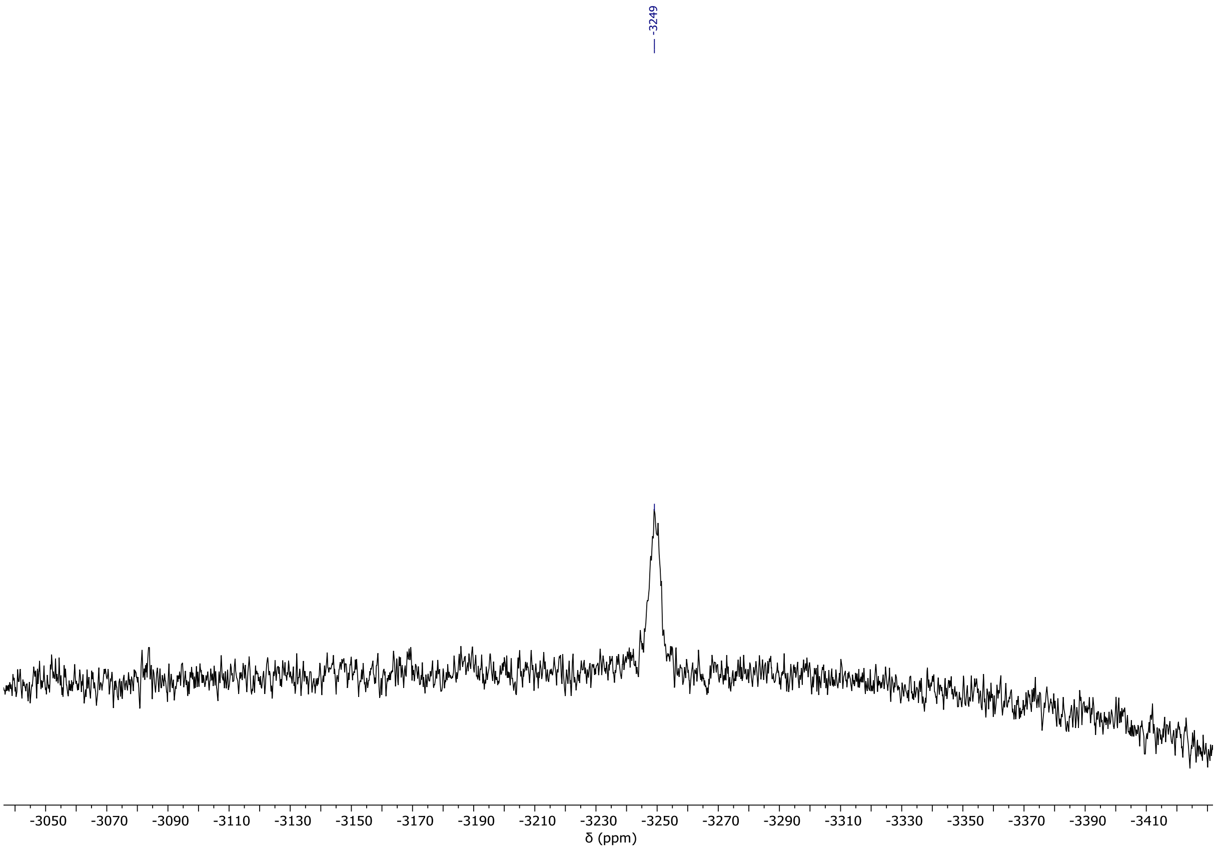

Figure S37: $^{195}Pt\{^{1}H\}$-NMR spectrum (107 MHz, CD_2Cl_2) of [**PtLHCl**].

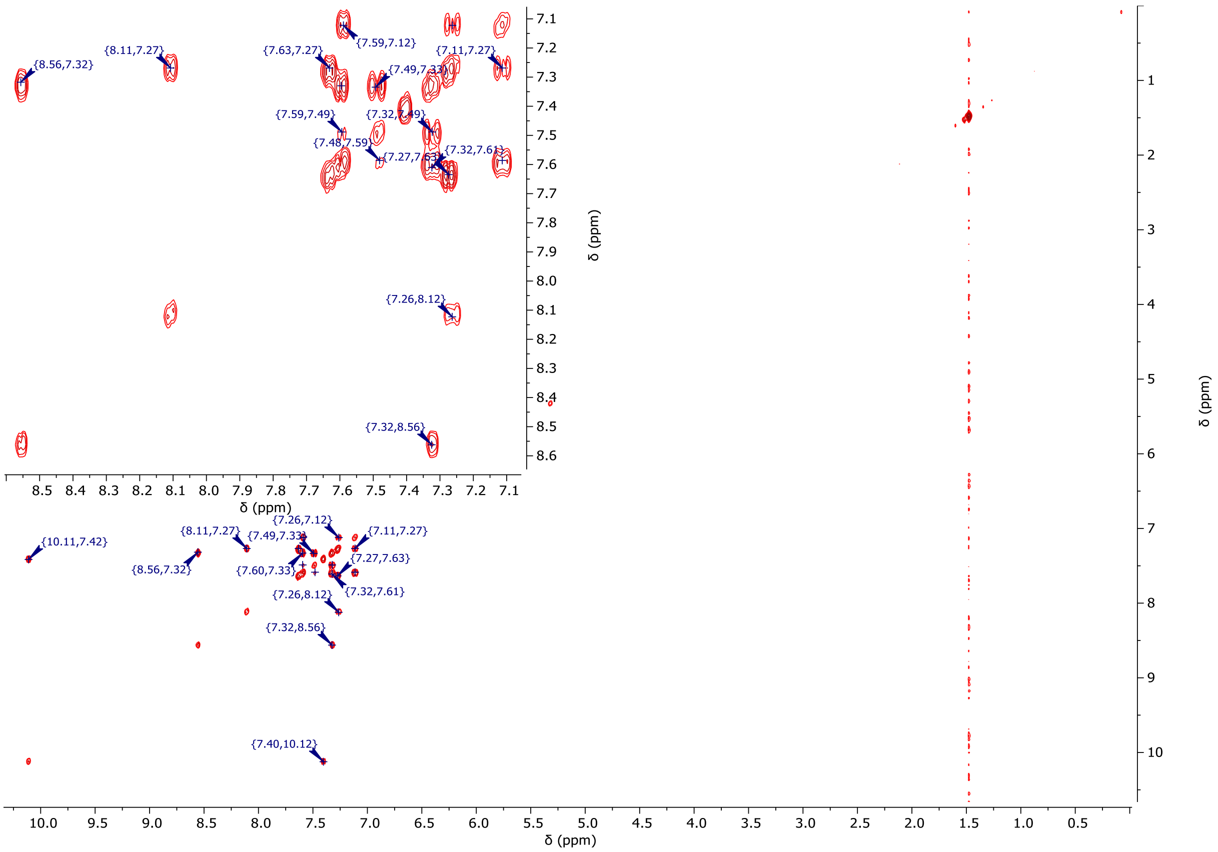

Figure S38: $^{1}H/^{1}H$-COSY-NMR spectrum (500 MHz/500 MHz, CD_2Cl_2) of [**PtLHCl**].

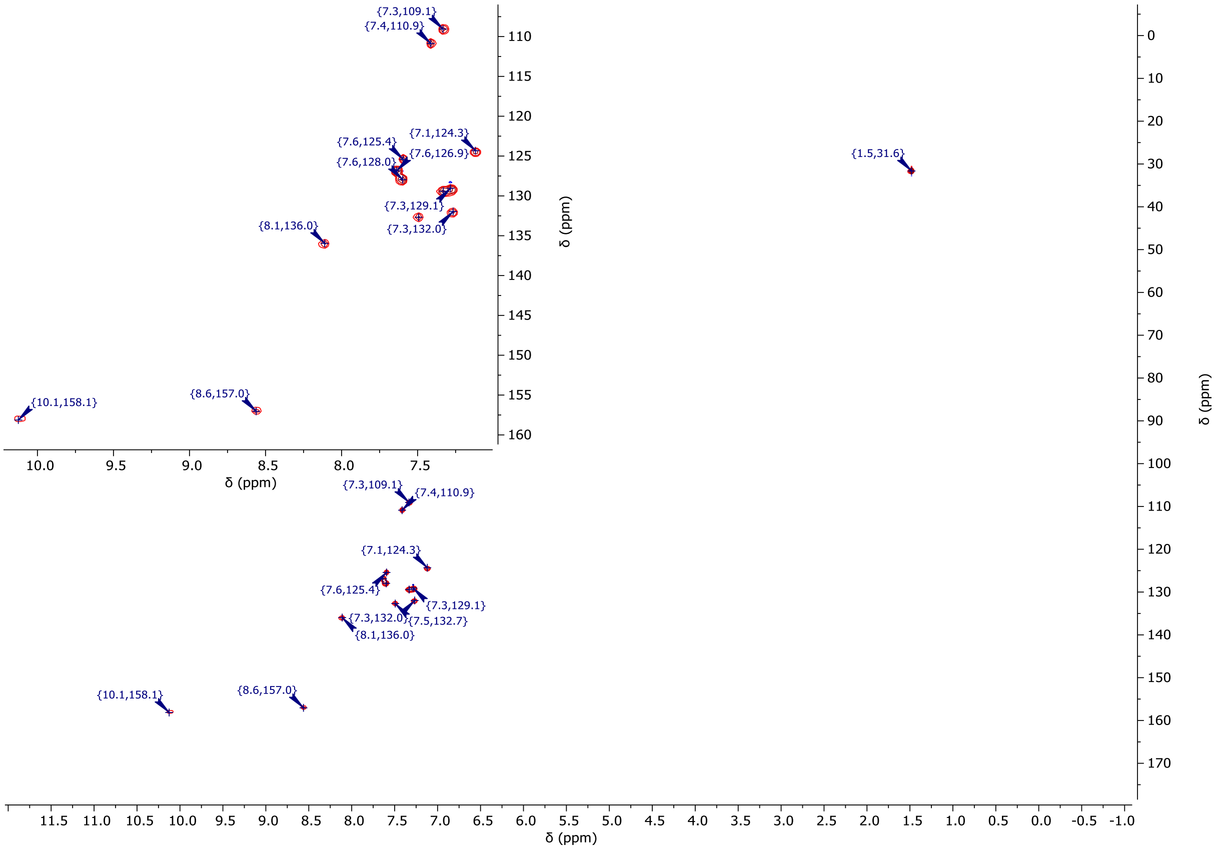

Figure S39: $^{1}H/^{13}C$-gHSQC-NMR spectrum (500 MHz/126 MHz, CD_2Cl_2) of [**PtLHCl**].

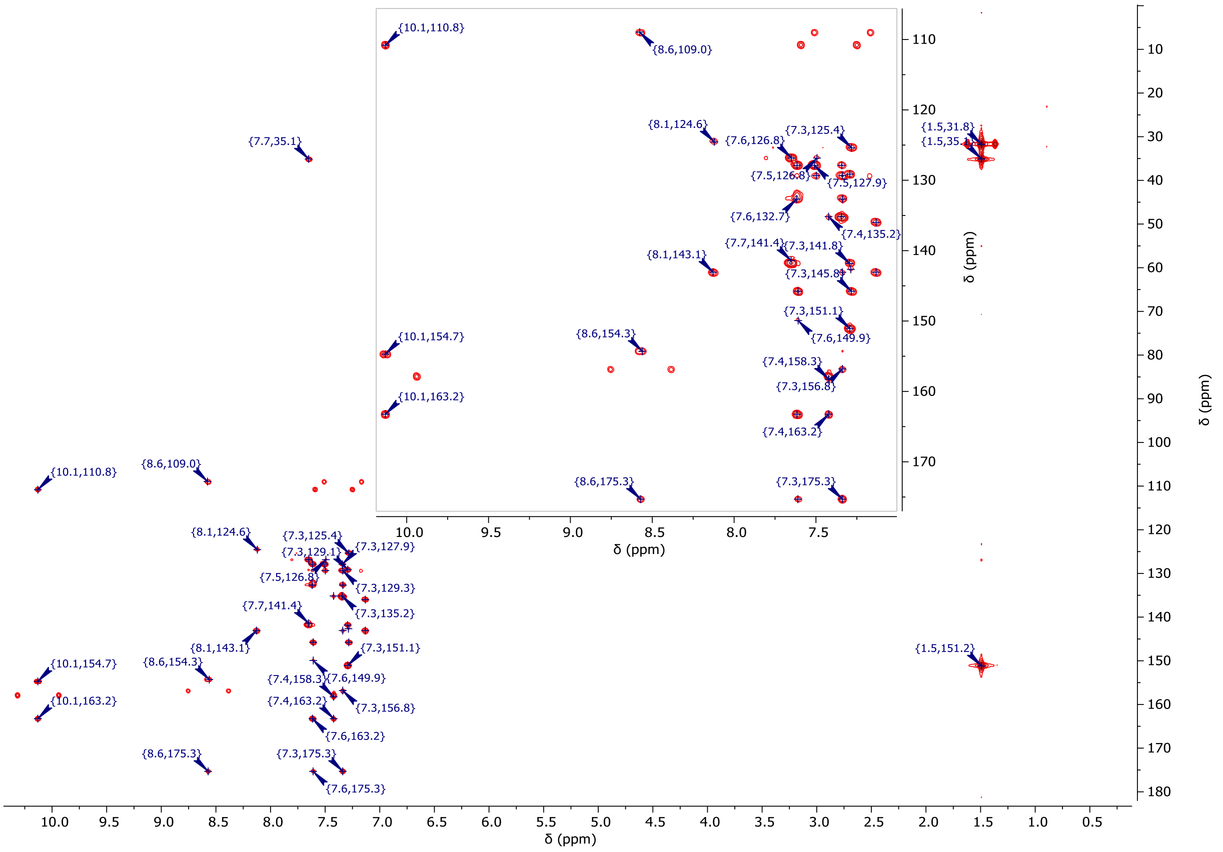

Figure S40: $^{1}H/^{13}C$-gHMBC-NMR spectrum (500 MHz/126 MHz, CD_2Cl_2) of [**PtLHCl**].

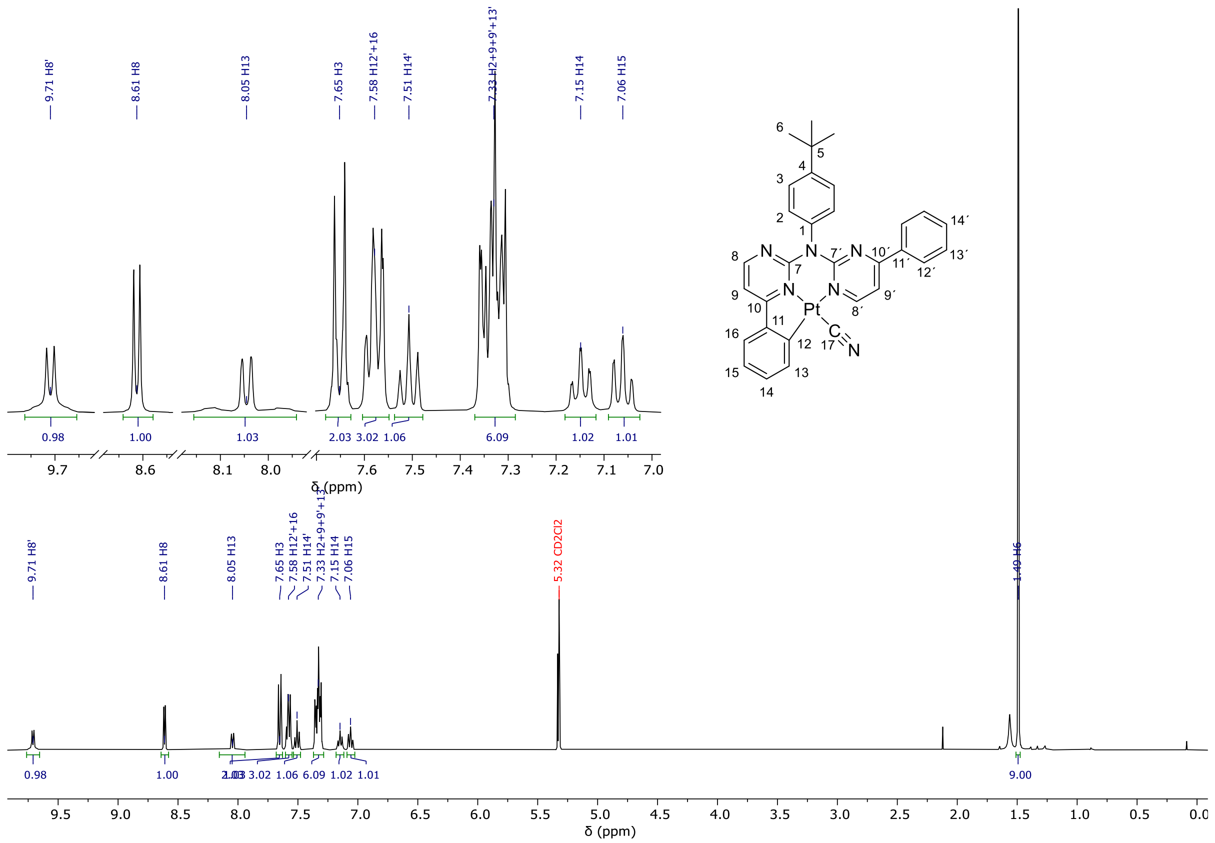

Figure S41: ^{1}H-NMR spectrum (400 MHz, CD_2Cl_2) of [**PtLHCN**].

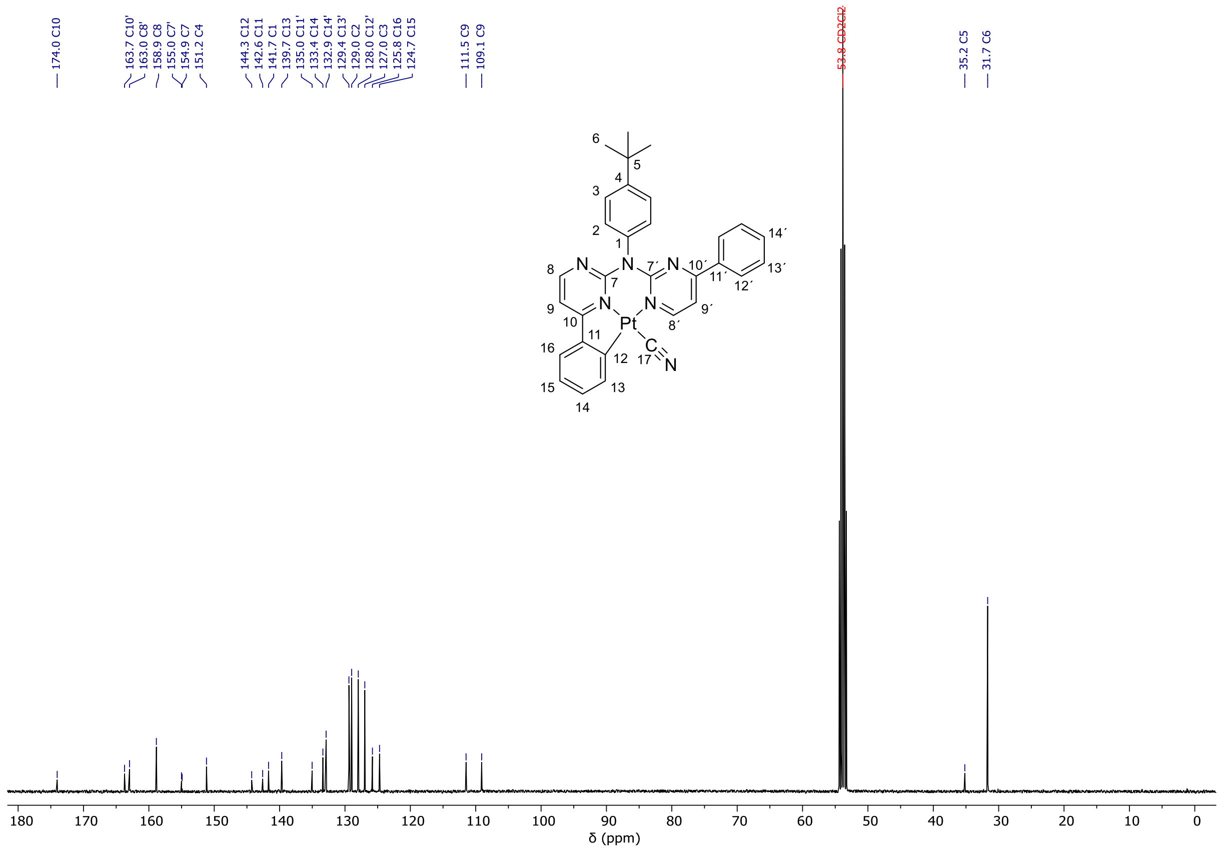

Figure S42: ^{13}C{^{1}H}-NMR spectrum (101 MHz, CD_2Cl_2) of [**PtLHCN**].

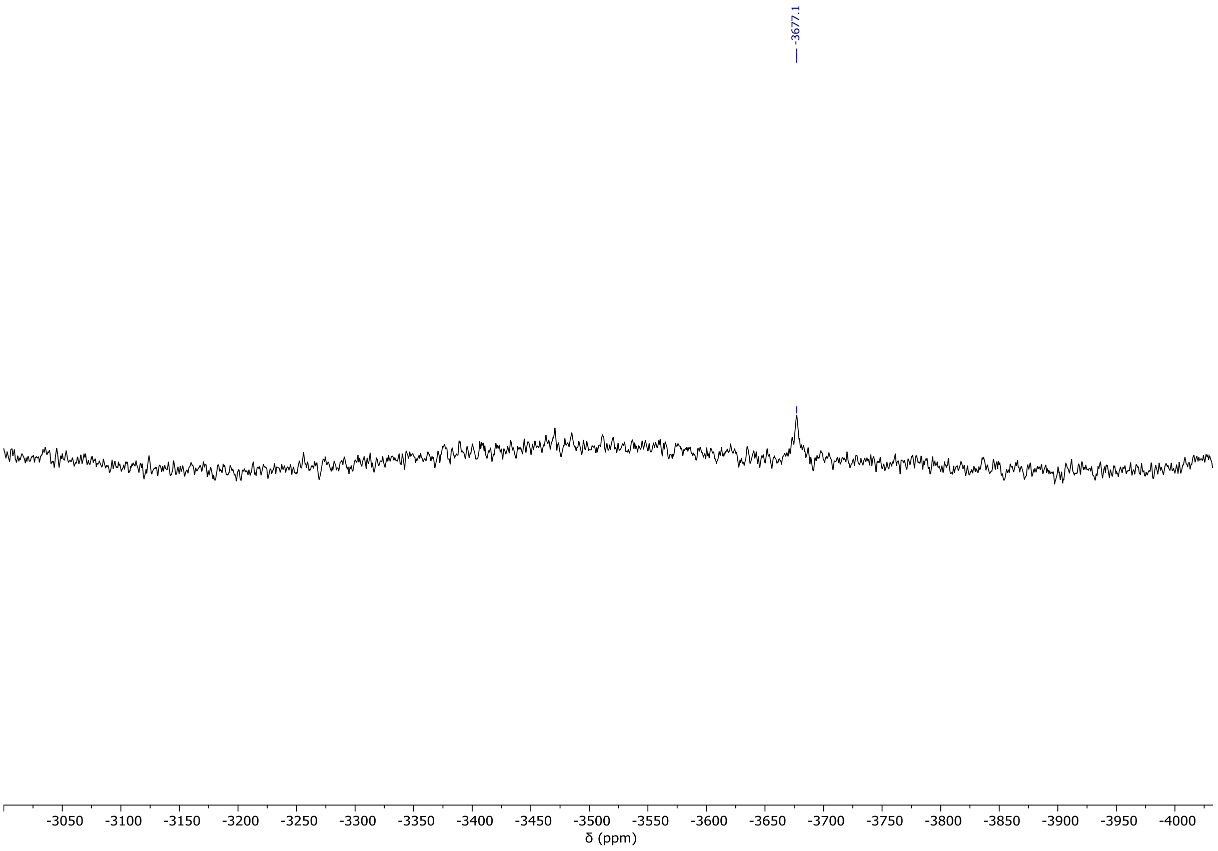

Figure S43: ^{195}Pt{^{1}H}-NMR spectrum (86 MHz, CD_2Cl_2) of [**PtLHCN**].

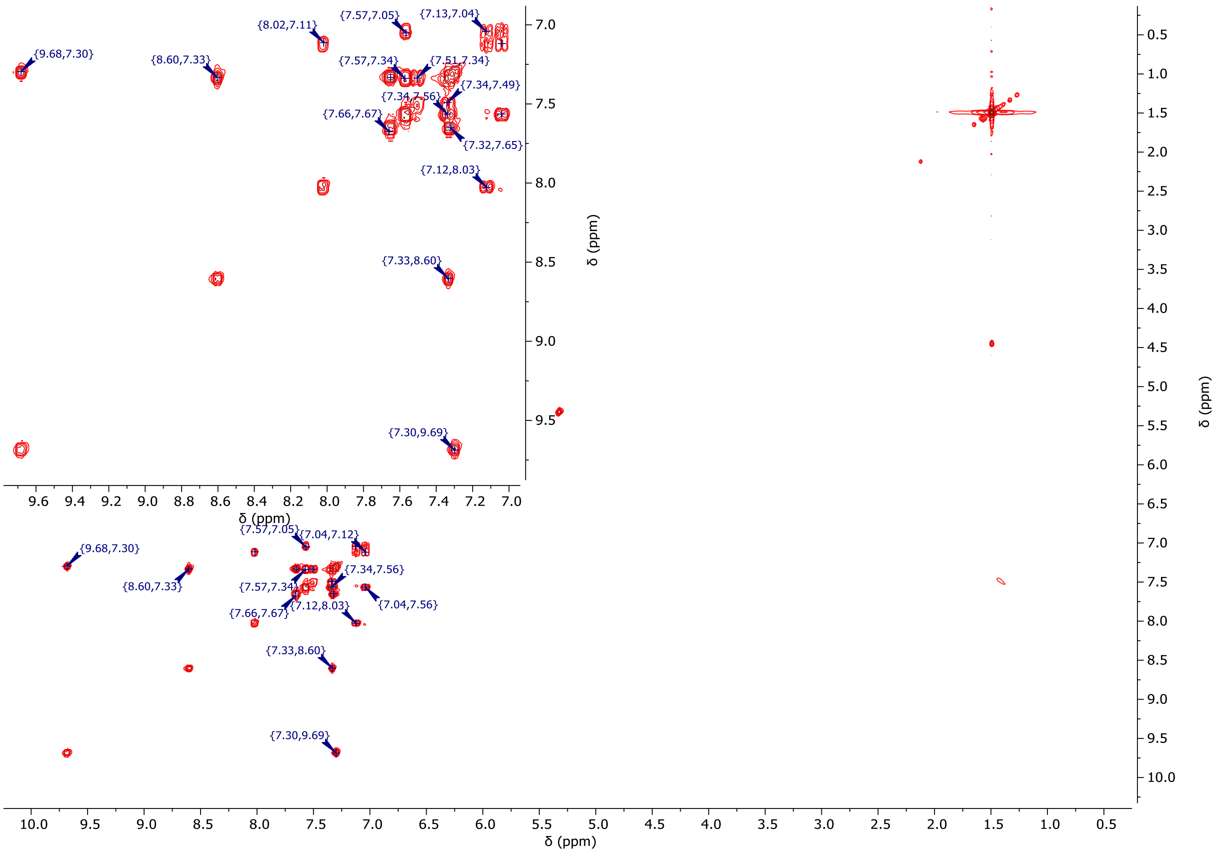

Figure S44: ^{1}H/^{1}H-COSY-NMR spectrum (400 MHz/400 MHz, CD_2Cl_2) of [**PtLHCN**].

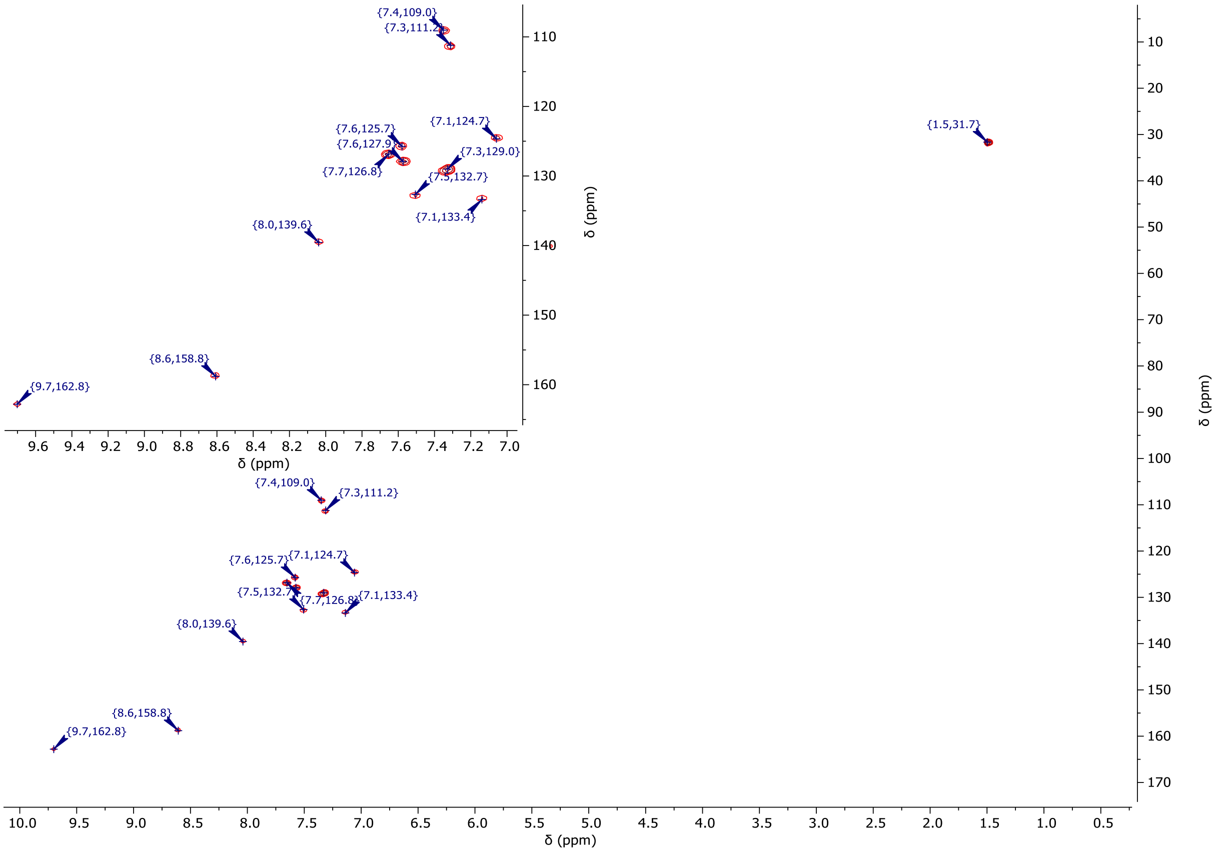

Figure S45: $^{1}H/^{13}C$-gHSQC-NMR spectrum (400 MHz/101 MHz, CD_2Cl_2) of [**PtLHCN**].

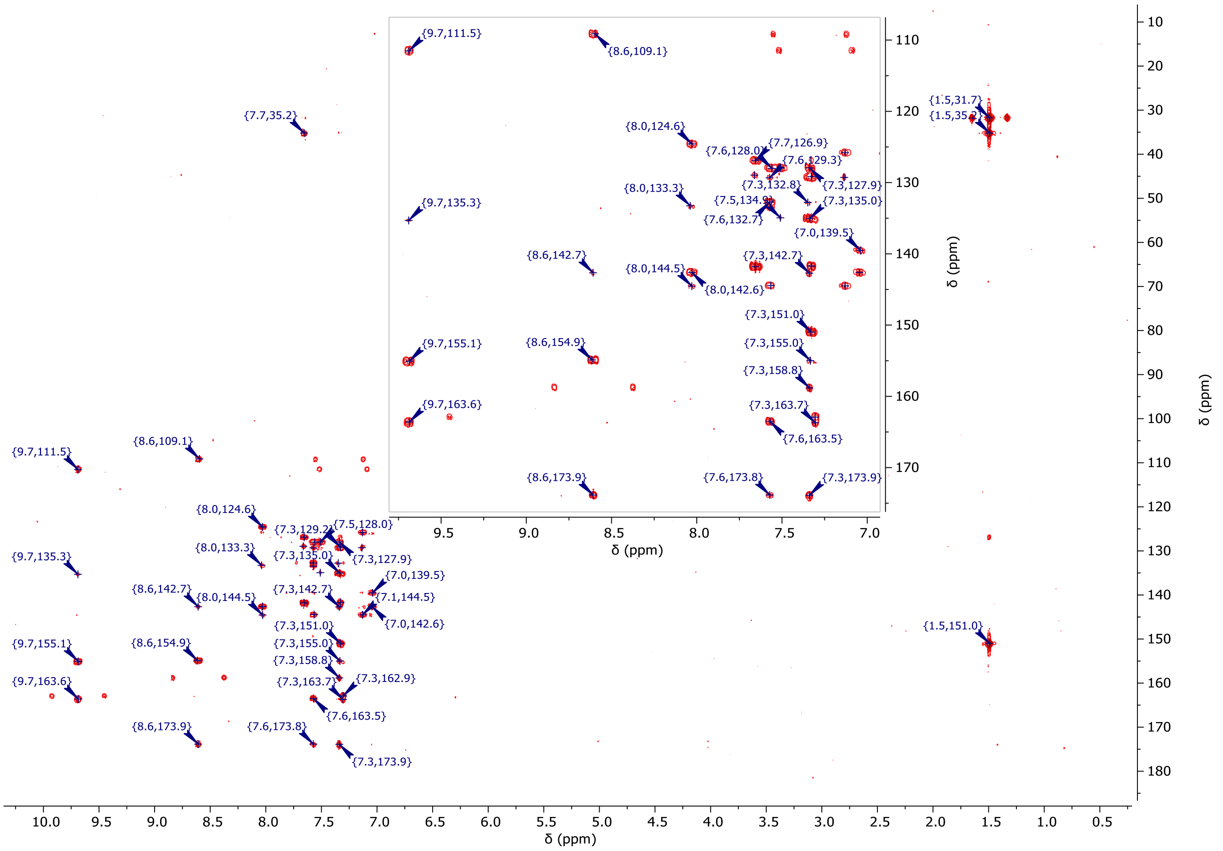

Figure S46: $^{1}H/^{13}C$-gHMBC-NMR spectrum (400 MHz/101 MHz, CD_2Cl_2) of [**PtLHCN**].

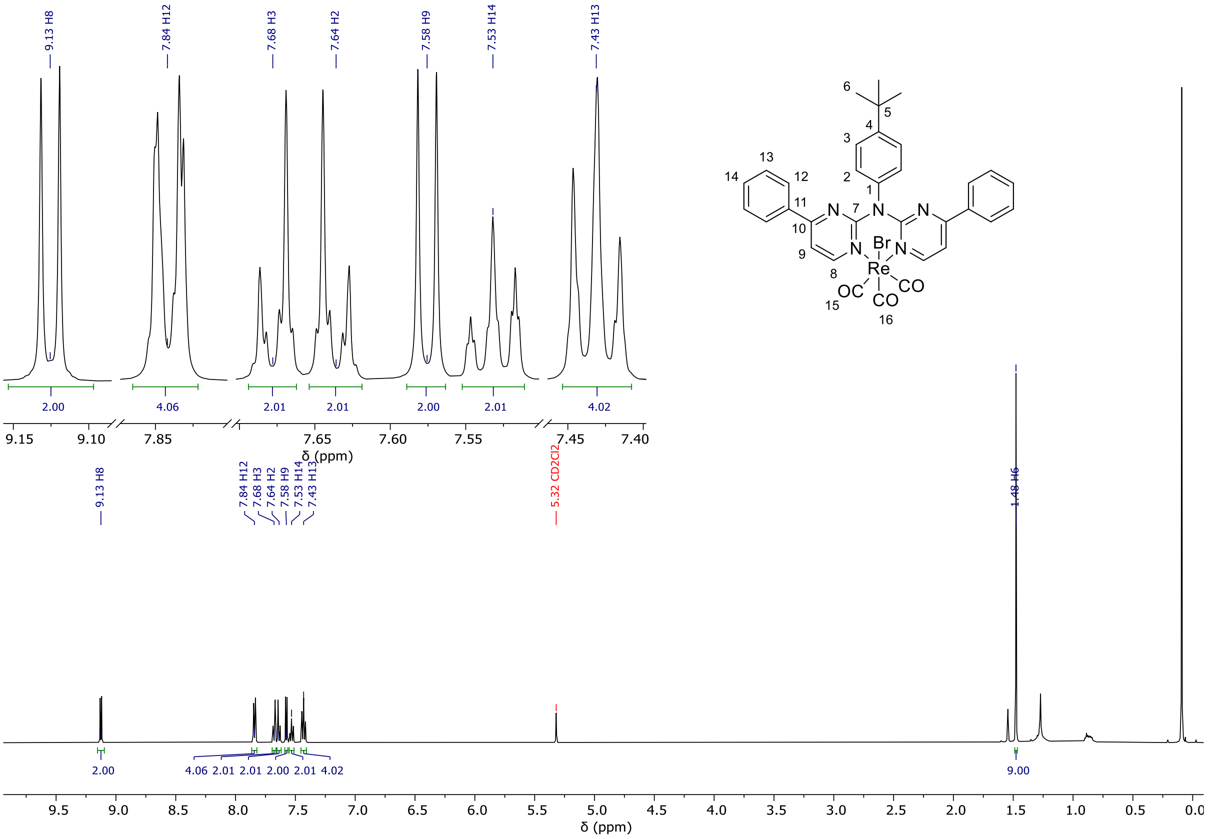

Figure S47: ^{1}H-NMR spectrum (500 MHz, CD_2Cl_2) of [**$ReLH_2(CO)_3Br$**].

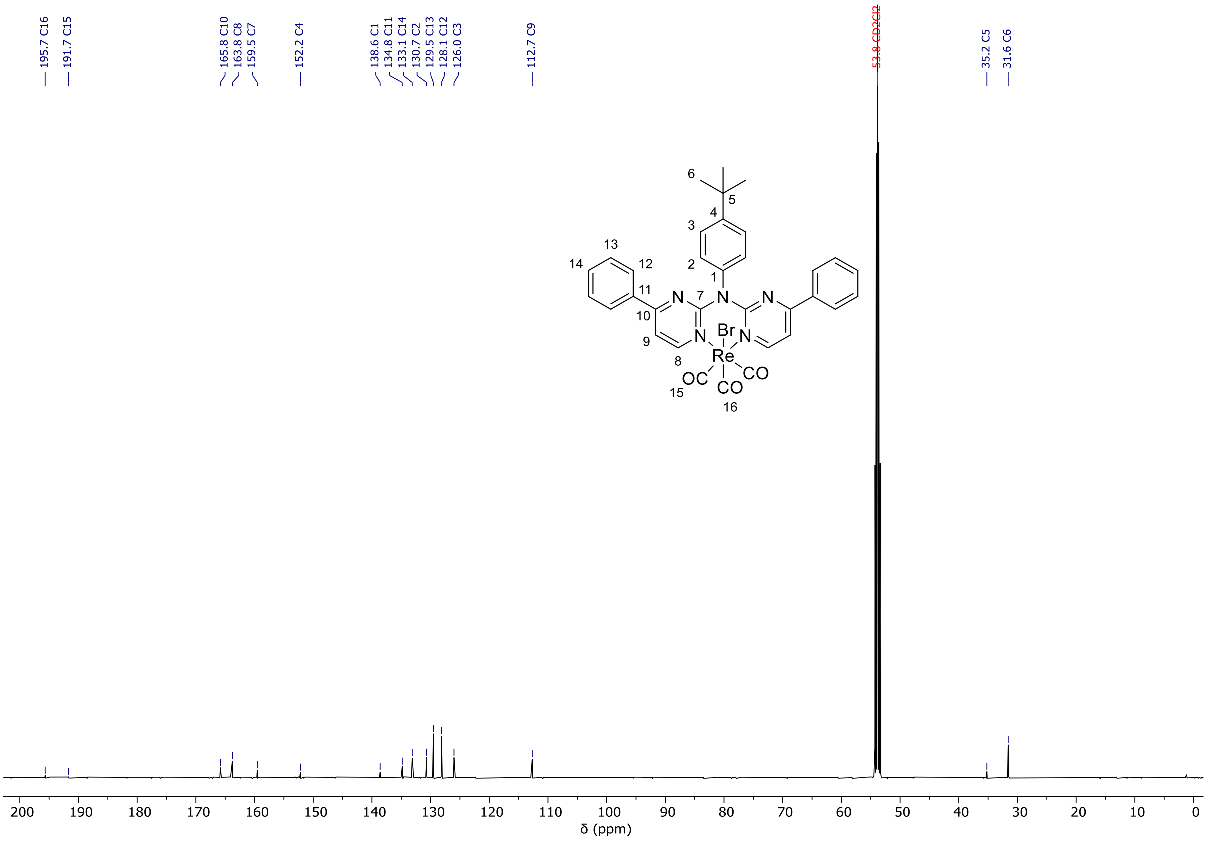

Figure S48: $^{13}C\{^{1}H\}$-NMR spectrum (126 MHz, CD_2Cl_2) of [**$ReLH_2(CO)_3Br$**].

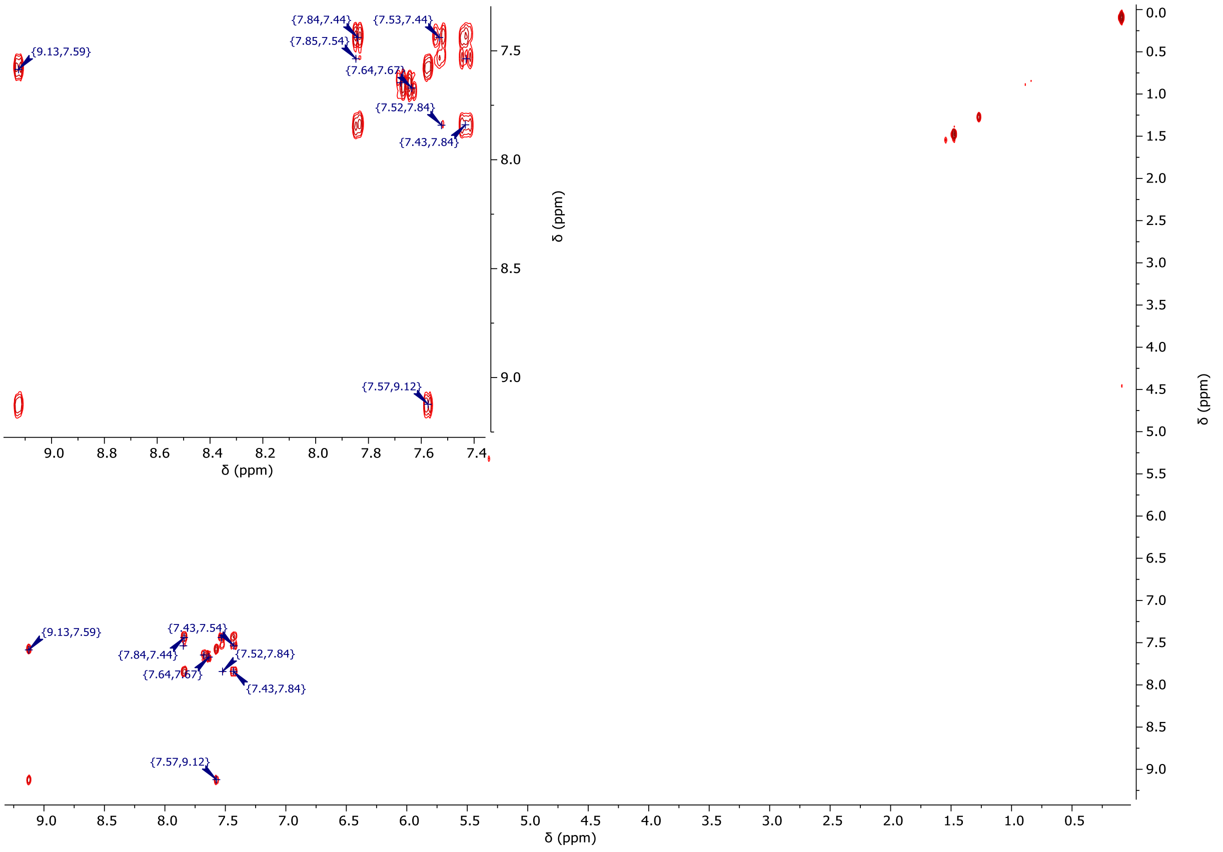

Figure S49: $^{1}H/^{1}H$-COSY-NMR spectrum (500 MHz/500 MHz, CD_2Cl_2) of [**$ReLH_2(CO)_3Br$**].

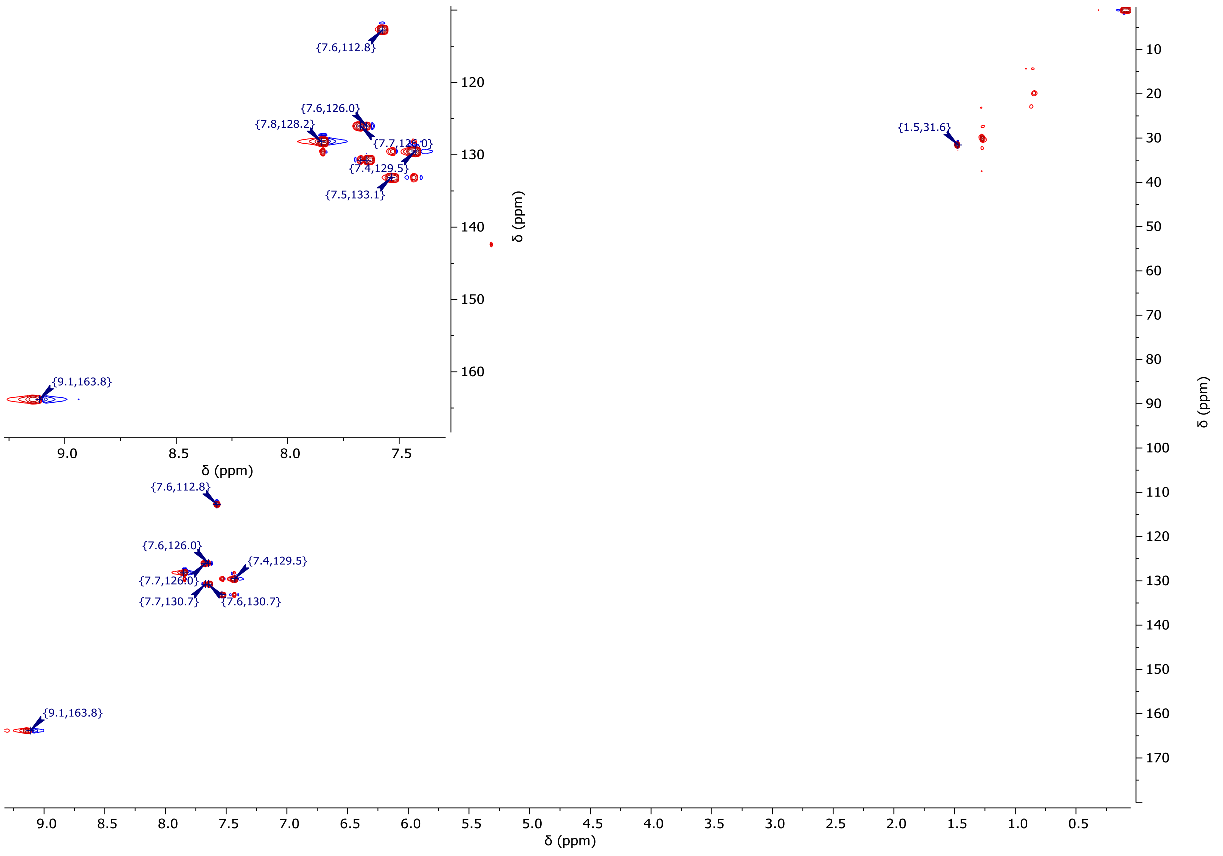

Figure S50: $^{1}H/^{13}C$-gHSQC-NMR spectrum (500 MHz/126 MHz, CD_2Cl_2) of [**$ReLH_2(CO)_3Br$**].

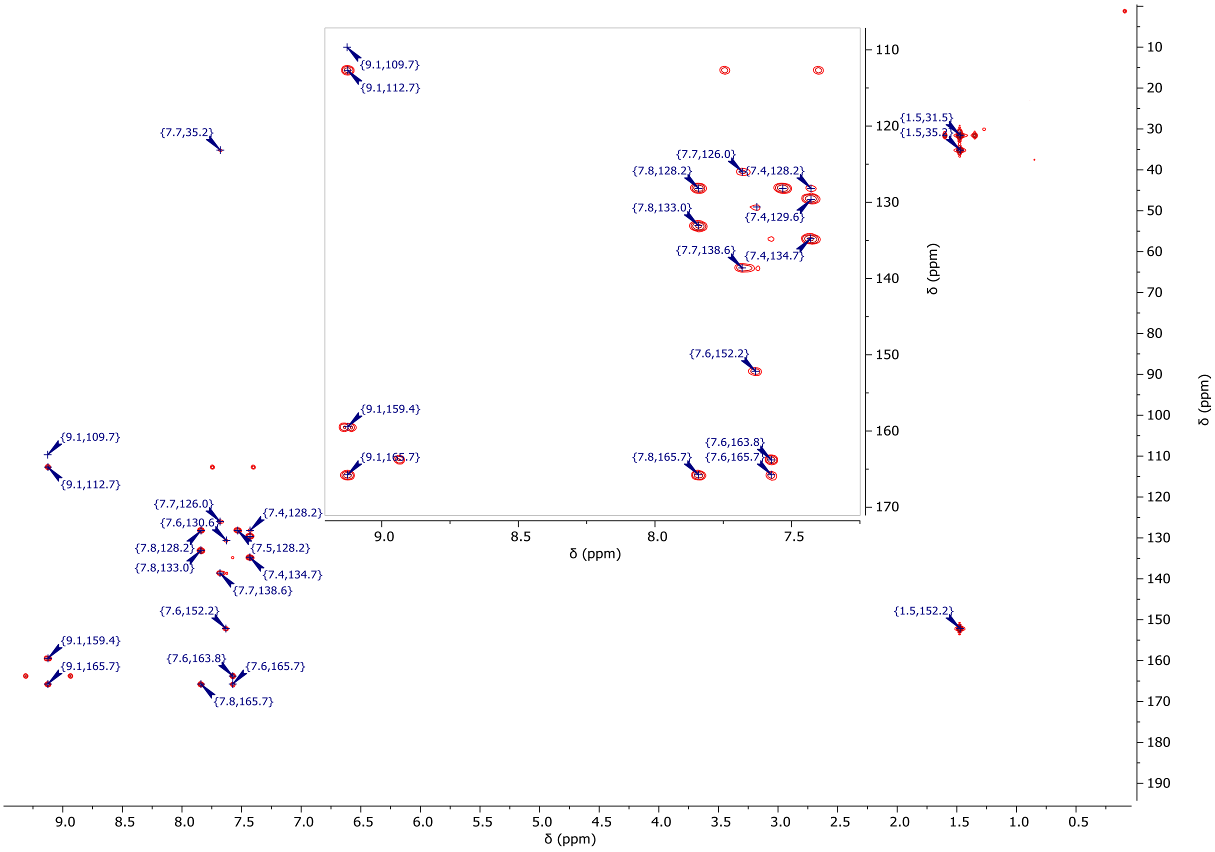

Figure S51: $^{1}H/^{13}C$-gHMBC-NMR spectrum (500 MHz/126 MHz, CD_2Cl_2) of [**$ReLH_2(CO)_3Br$**].

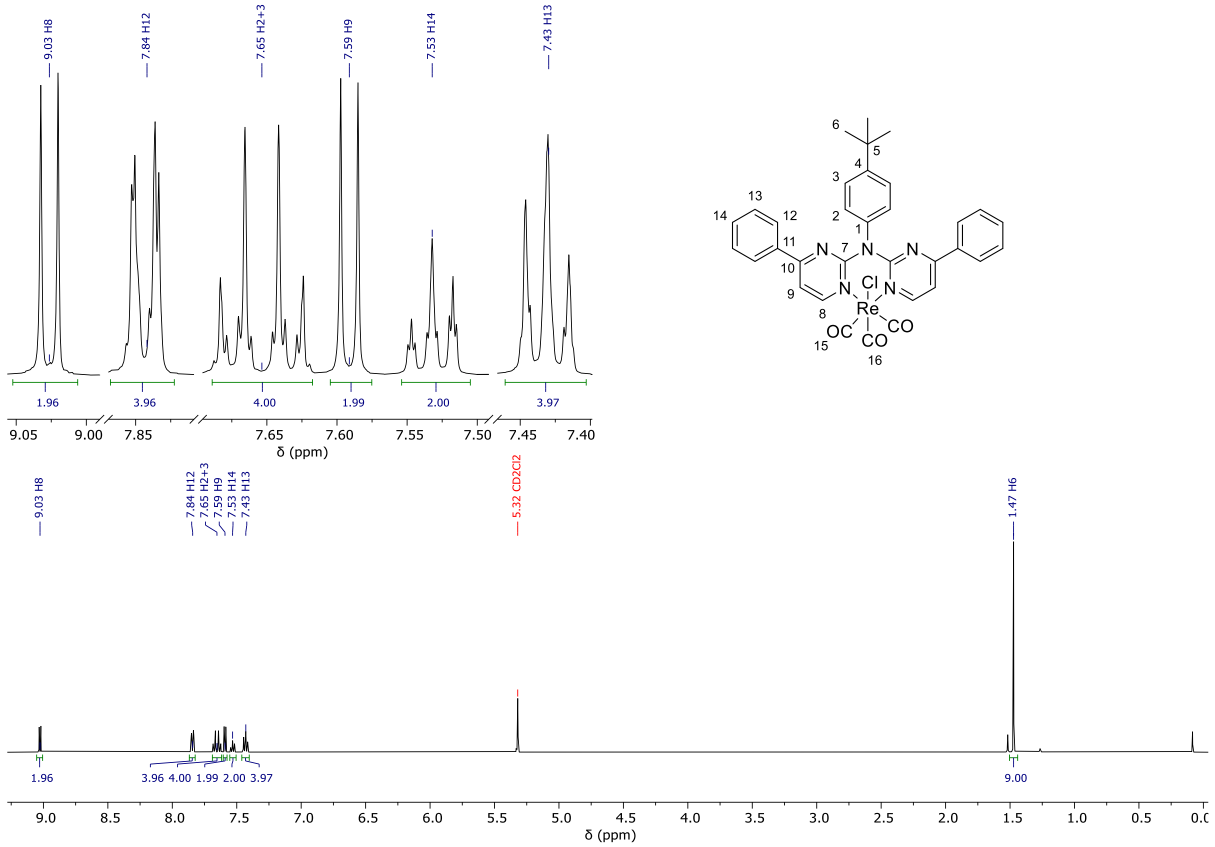

Figure S52: ^{1}H-NMR spectrum (500 MHz, CD_2Cl_2) of [**$ReLH_2(CO)_3Cl$**].

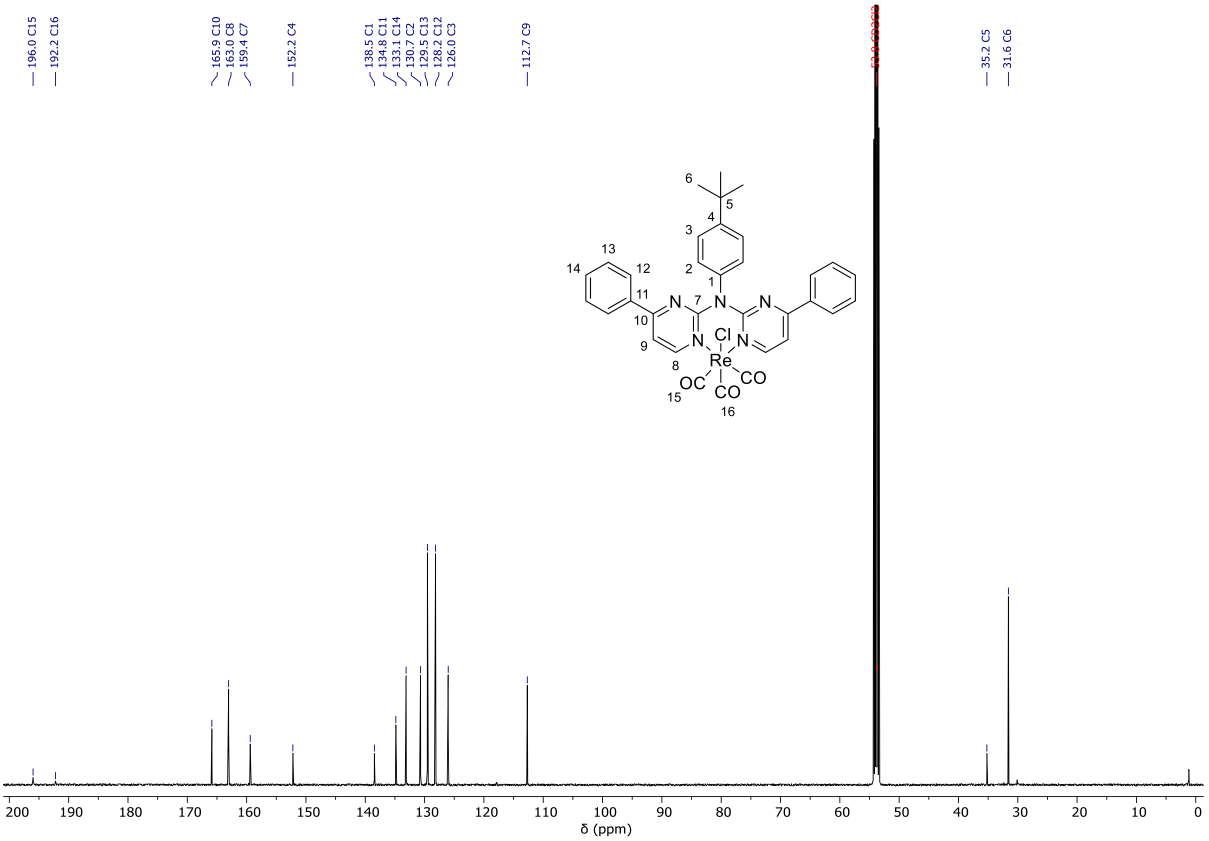

Figure S53: $^{13}C\{^{1}H\}$-NMR spectrum (126 MHz, CD_2Cl_2) of [**ReLH$_2$(CO)$_3$Cl**].

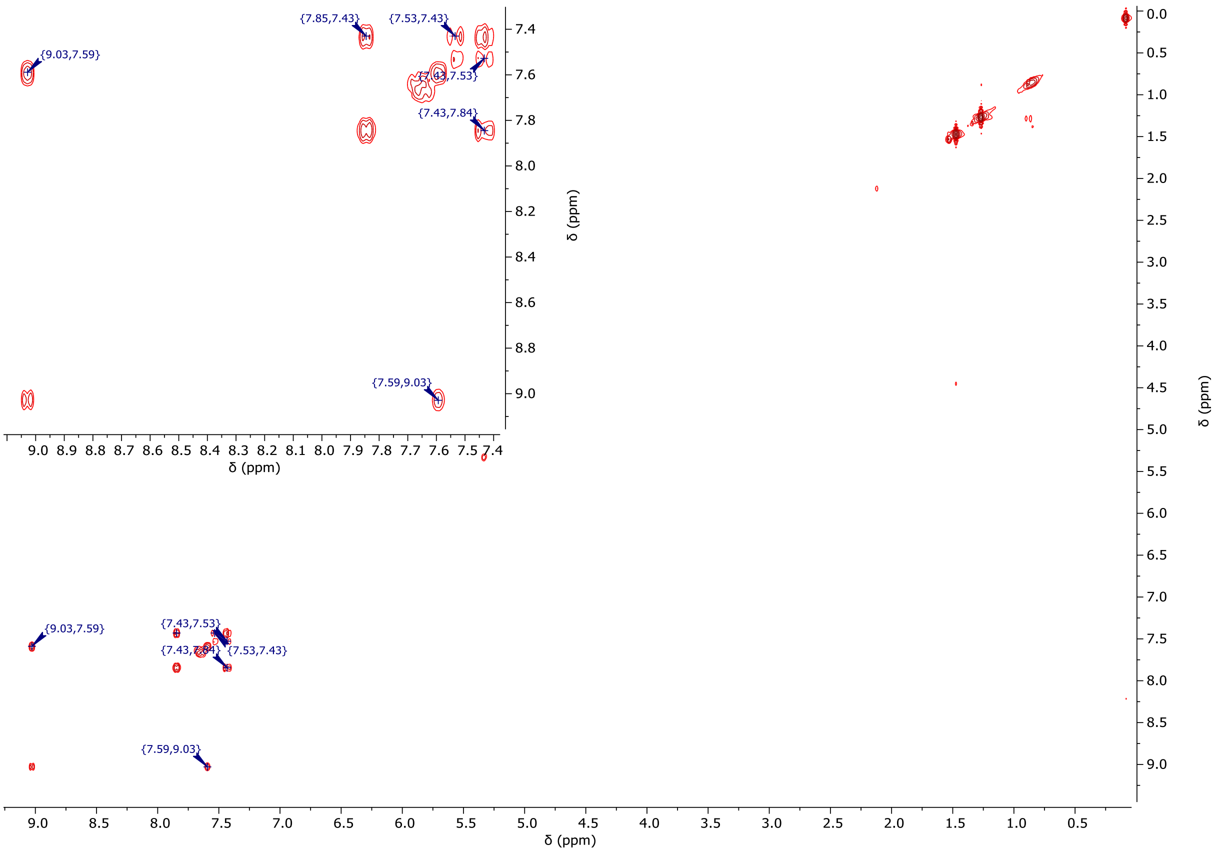

Figure S54: $^{1}H/^{1}H$-COSY-NMR spectrum (400 MHz/400 MHz, CD_2Cl_2) of [**ReLH$_2$(CO)$_3$Cl**].

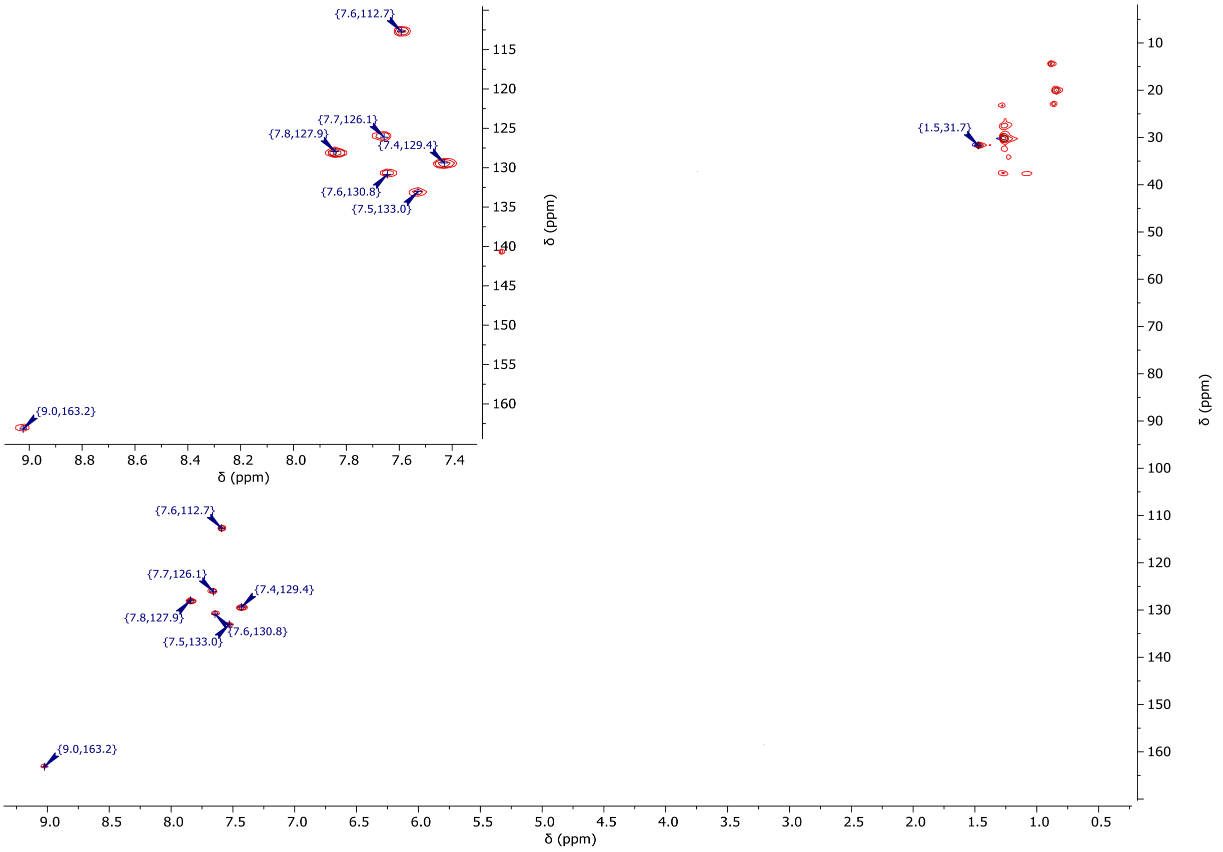

Figure S55: $^{1}H/^{13}C$-gHSQC-NMR spectrum (400 MHz/101 MHz, CD_2Cl_2) of [$\mathbf{ReLH_2(CO)_3Cl}$].

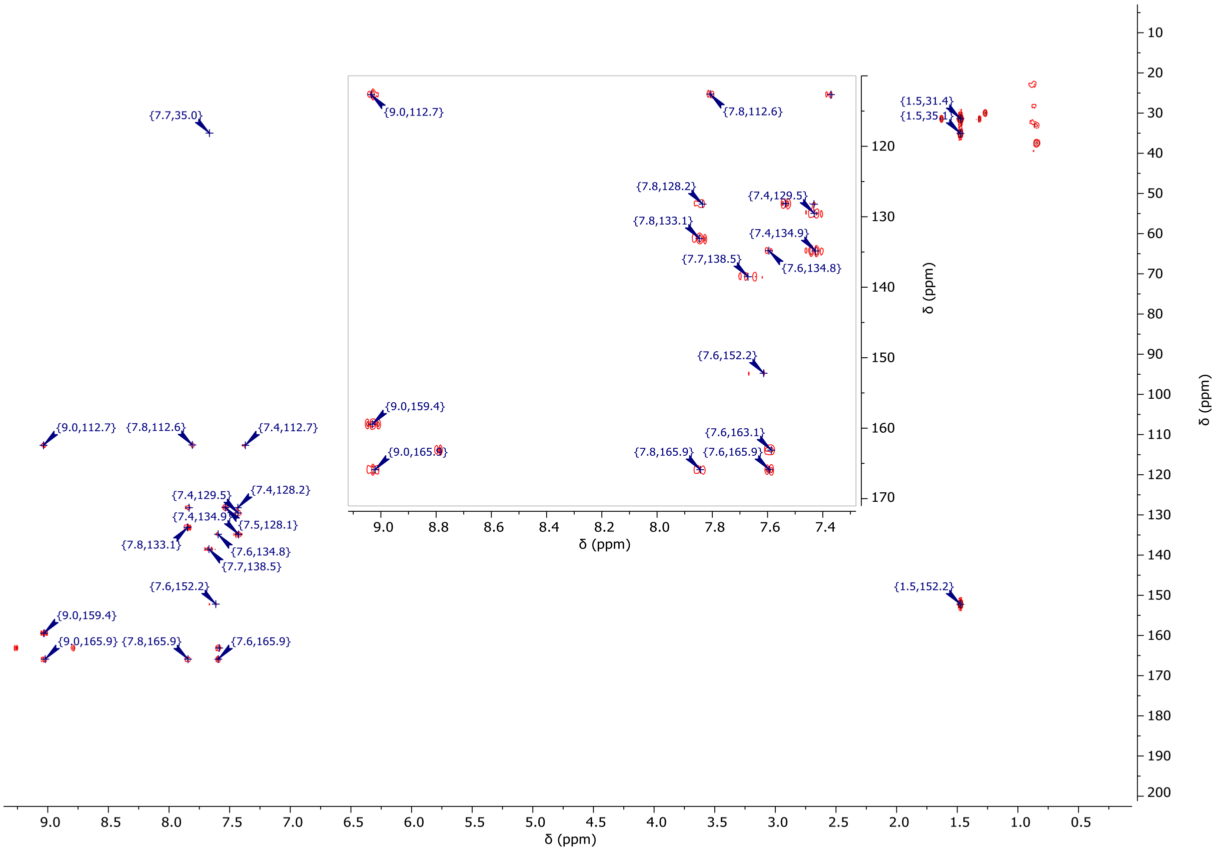

Figure S56: $^{1}H/^{13}C$-gHMBC-NMR spectrum (400 MHz/101 MHz, CD_2Cl_2) of [$\mathbf{ReLH_2(CO)_3Cl}$].

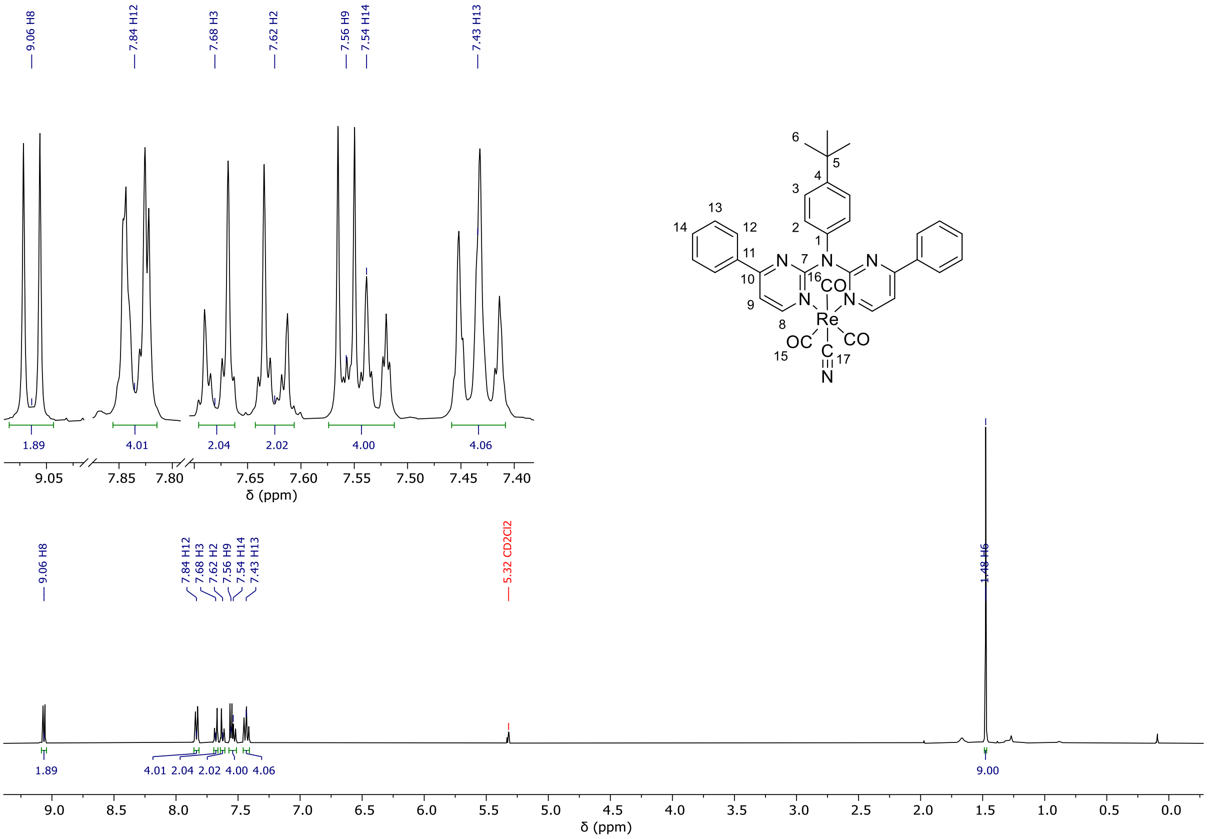

Figure S57: ^{1}H-NMR spectrum (400 MHz, CD_2Cl_2) of [**ReLH$_2$(CO)$_3$CN**].

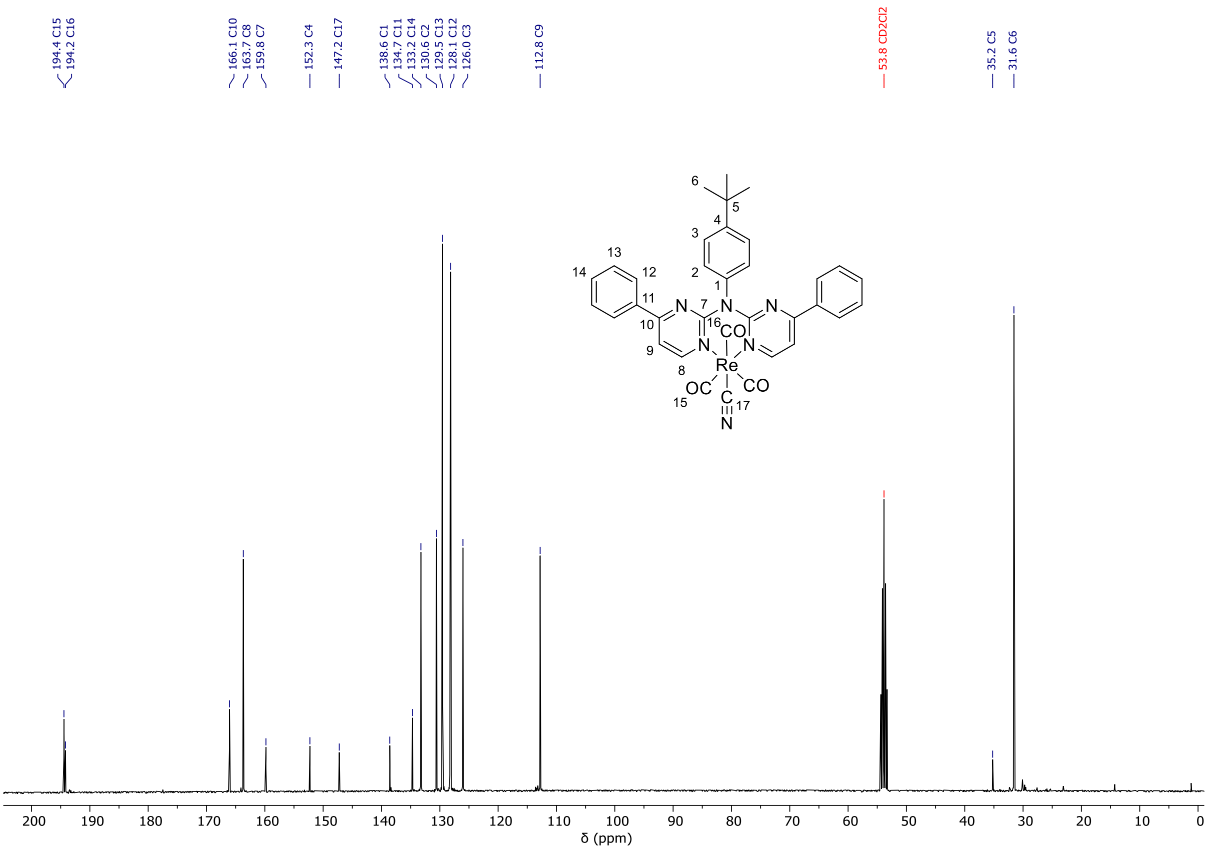

Figure S58: ^{13}C{^{1}H}-NMR spectrum (101 MHz, CD_2Cl_2) of [**ReLH$_2$(CO)$_3$CN**].

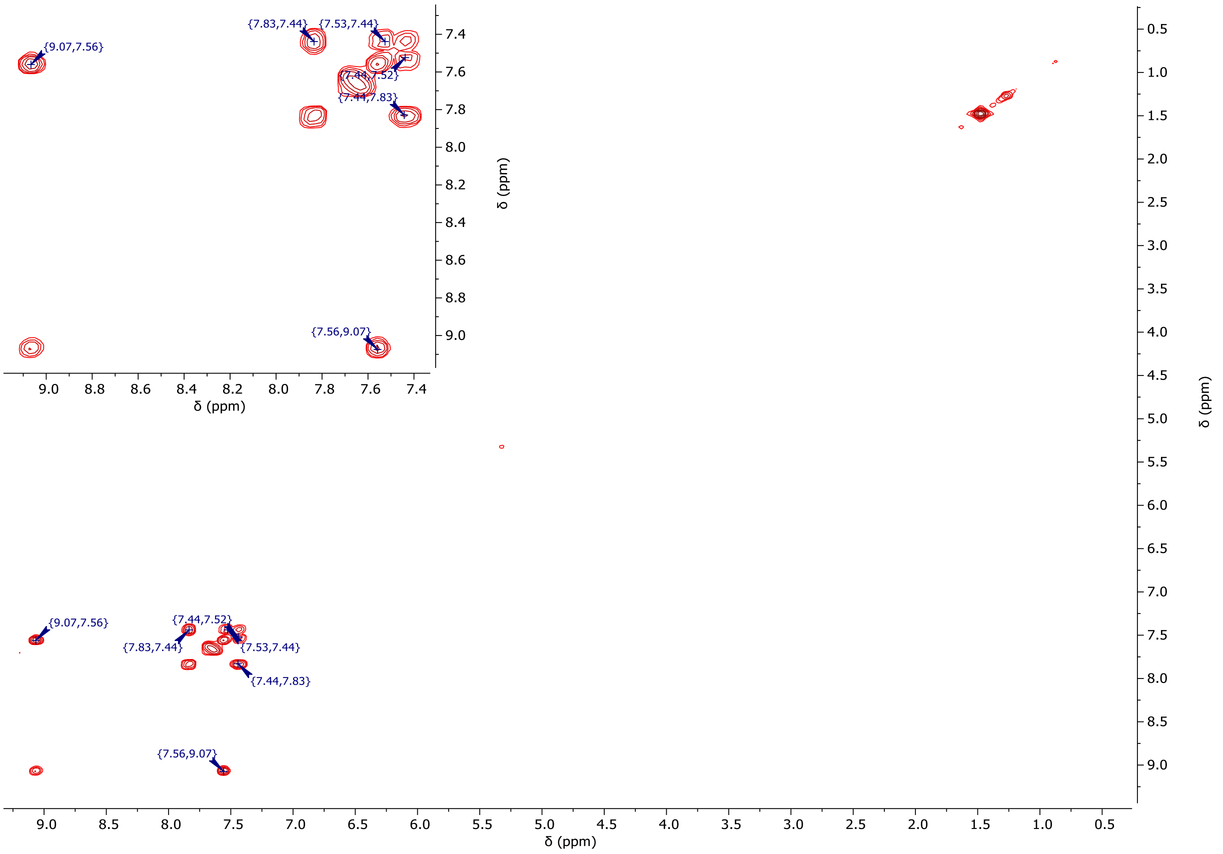

Figure S59: ${}^{1}H/{}^{1}H$-COSY-NMR spectrum (400 MHz/400 MHz, CD_2Cl_2) of [$\mathbf{ReLH_2(CO)_3CN}$].

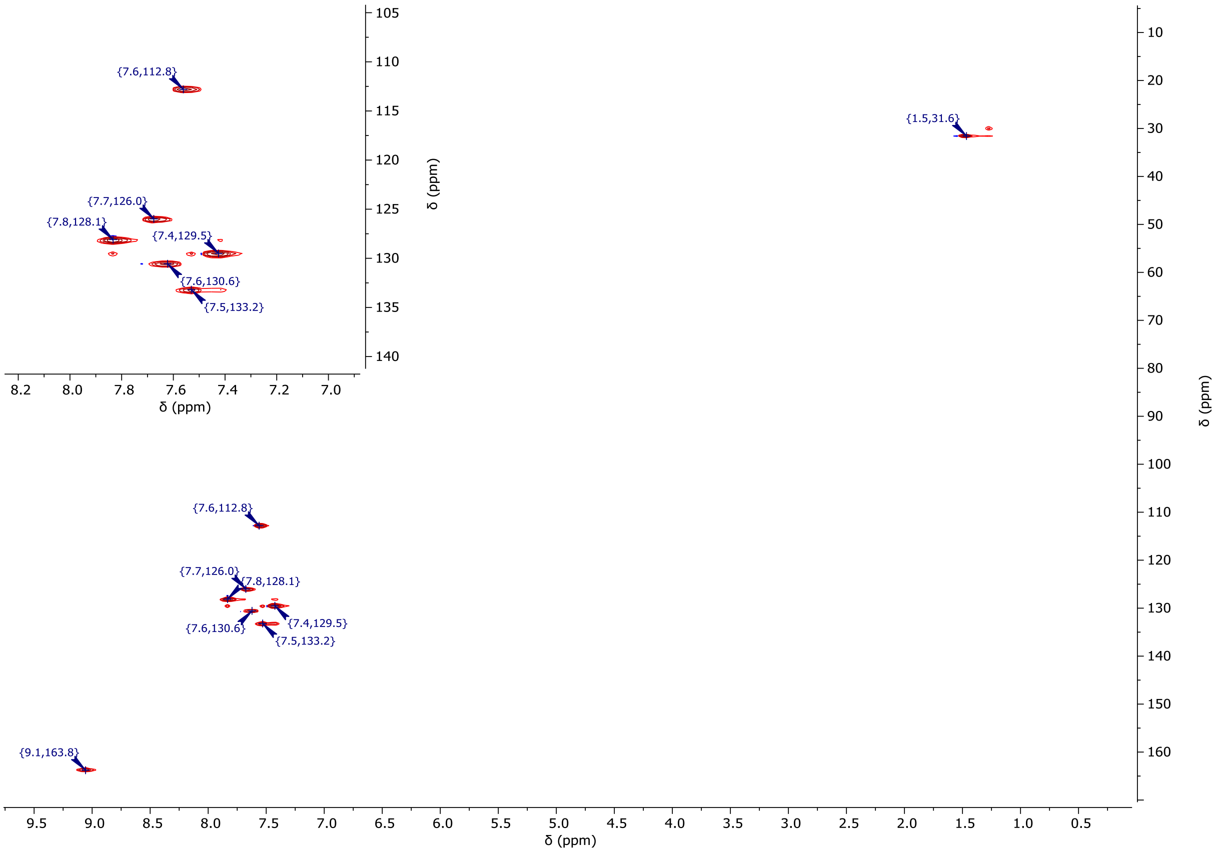

Figure S60: ${}^{1}H/{}^{13}C$-gHSQC-NMR spectrum (400 MHz/101 MHz, CD_2Cl_2) of [$\mathbf{ReLH_2(CO)_3CN}$].

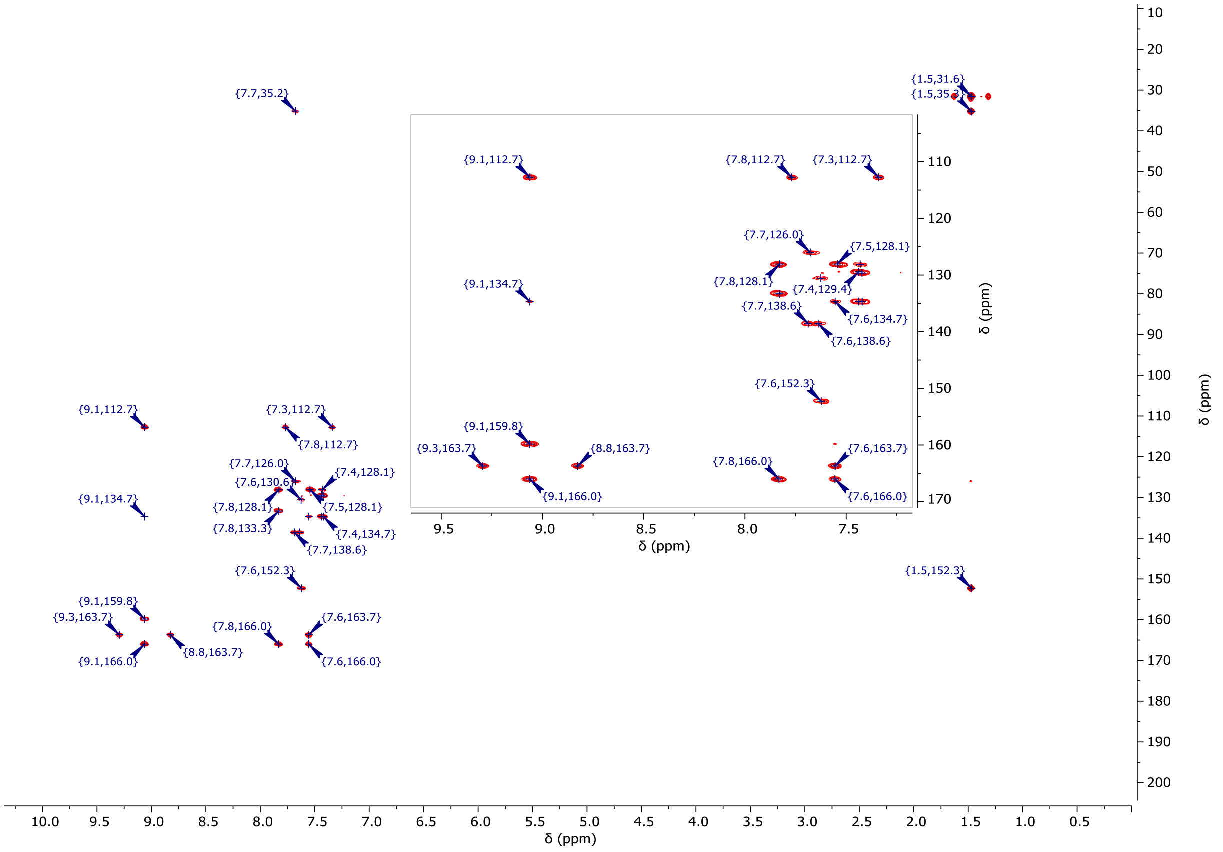

Figure S61: $^{1}H/^{13}C$-gHMBC-NMR spectrum (400 MHz/101 MHz, CD_2Cl_2) of [**$ReLH_2(CO)_3CN$**].

I.3 *FTIR spectra*

FTIR spectra were obtained on a Bruker Tensor 37 FT-IR spectrometer equipped with a A225 platinum ATR diamond unit, and acquired in the range 4000-550 cm^{-1}.

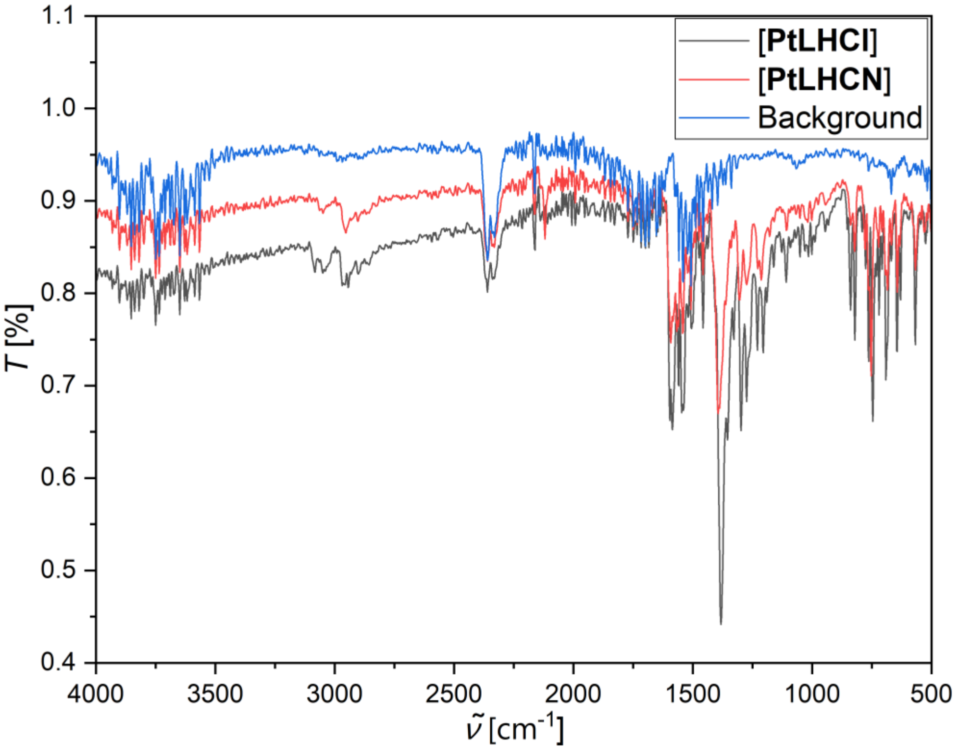

Figure S62: AT-FTIR-spectra of [**PtLHCl**] (black), [**PtLHCN**] (red) with the background (blue).

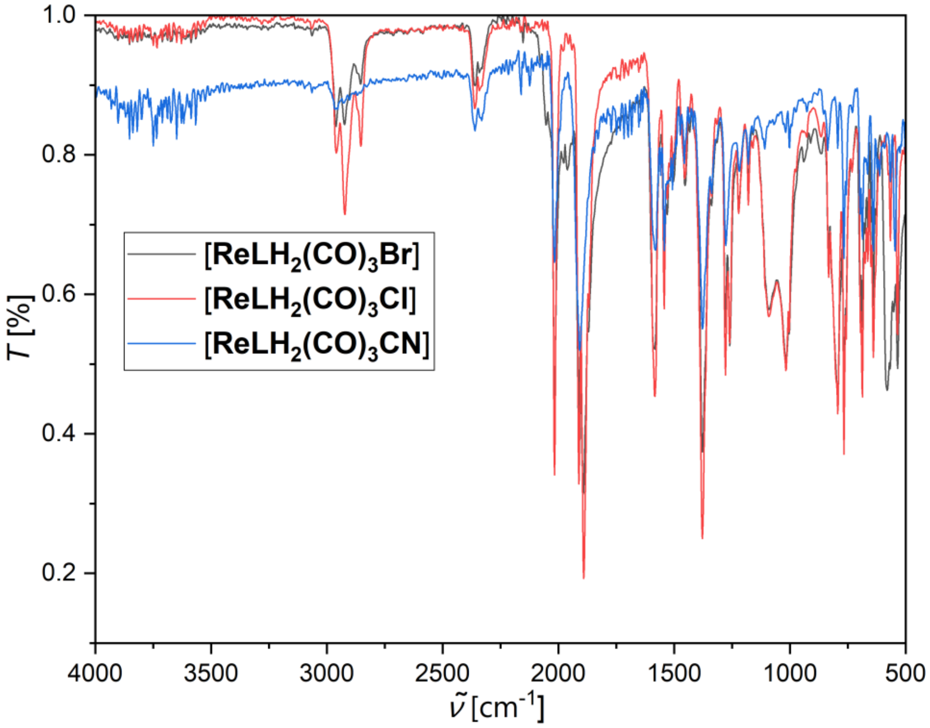

Figure S63: AT-FTIR-spectra of [**ReLH$_2$(CO)$_3$Br**] (black), [**ReLH$_2$(CO)$_3$Cl**] (red) and [**ReLH$_2$(CO)$_3$CN**] (blue).

II. X-ray diffractometry on single crystals

Single crystals suitable for diffractometry were obtained by slowly evaporating the solvent of a saturated DCM solution of the compound or by slowly diffusing cyclohexane into such a solution. The single crystals for [**$PtLH_2Cl_2$**] were obtained by cooling a hot saturated DMSO solution of the complex. The single crystals for the yellow modification of [**PtLHCN**](yellow) were obtained by slowly diffusing cyclohexane in a saturated EtOAc solution of the complex. The single crystals for [**$ReLH_2(CO)_3Br$**] were obtained by slowly evaporating the solvent of a saturated $CDCl_3$ solution of the compound.

X-ray diffractometry: The data sets for all compounds were collected with Bruker D8 Venture equipped with PHOTON III CMOS diffractometer. Diffraction frames were recorded with APEX4 software package.[3] Data integration and adsorption correction were achieved with SAINT and SADABS.[4,5] Structures were solved by intrinsic phase method and refined with Shelxtl 2019/1.[6] Software used to prepare material for publication: Mercury (Version 4.1.0).[7]

Table S1: Parameters and data from X-ray diffractometry on single crystals of **1** and **LH_2**.

**Complex**	**1**	**LH_2**
**Formula**	$C_{20}H_{21}N_3$	$2\ (C_{30}H_{27}N_5) \cdot CH_2Cl_2$
**Fw**	303.40	1000.05
**CCDC No.**	2394603	2394610
**Temperature/K**	100(2)	100(2)
**Crystal color**	rod, colourless	block, colourless
**Crystal system**	monoclinic	monoclinic
Space group	$P2_1/n$	$P2_1/n$
***a*/Å**	12.3044(4)	19.055(8)
***b*/Å**	6.5420(2)	11.489(5)
***c*/Å**	21.081(1)	24.161(17)
***α*/°**	90	90
***β*/°**	97.081(1)	101.068(17)
***γ*/°**	90	90
**V/Å^3**	1683.52(10)	5191 (4)
***Z* value**	4	4
**Calculated density/g cm^{-3}**	1.197	1.280
**Crystal size/mm^3**	0.44 × 0.11 × 0.08	0.76 × 0.31 × 0.13
**Radiation/wavelength/pm**	Mo*K*α / 71.073	Mo*K*α / 71.073
**μ(MoKα)/mm^{-1}**	0.07	0.18
**F000**	648	2104
**θ range, deg**	3.18 – 32.04	2.17 – 32.52
**h,k,l_{max}**	±16, ±8, ±27	±25, ±15, ±31
**T_{min},T_{max}**	0.691, 0.746	0.664, 0.746
**Total no. reflections**	24855	79022
**Independent reflections / R_{int}**	4021 / 0.0321	12378 / 0.041
**Reflections with $I > 2\sigma(I)$ / R_σ**	3550 / 0.0261	11332 / 0.0280
**Data/parameters**	4021 / 211	12378 / 664
**Goodness-of-fit on F^2**	1.043	1.076
**$R1/wR2$ for $I > 2\sigma(I)$**	0.0429 / 0.1076	0.0491 / 0.1206
**$R1/wR2$ for all data**	0.0485 / 0.1111	0.0535 / 0.1206
**Larg. diff. peak/hole/e Å^{-3}**	0.30 / -0.25	0.69 / -1.00

Table S2: Parameters and data from X-ray diffractometry on single crystals of [**$PtLH_2Cl_2$**] and [**$PtLH_2$cbda**].

**Complex**	[**$PtLH_2Cl_2$**]	[**$PtLH_2$cbda**]
**Formula**	$C_{30}H_{27}N_5Pt·C_2H_6SO$	$C_{36}H_{33}N_5O_4Pt·2(CH_2Cl_2)$
**Fw**	801.68	964.61
**CCDC No.**	2394604	2394611
**Temperature/K**	100(2)	240(2)
**Crystal color**	plate, colourless	plate, yellow
**Crystal system**	triclinic	triclinic
Space group	$P\bar{1}$	$P\bar{1}$
***a*/Å**	11.5288(9)	10.2994(3)
***b*/Å**	11.6217(8)	10.6978(4)
***c*/Å**	12.1029(9)	17.7259(6)
***α*/°**	96.818(3)	91.060(1)
***β*/°**	95.204(3)	91.998(1)
***γ*/°**	103.244(2)	95.093(1)
**V/Å^3**	1555.6(2)	1943.70(11)
***Z* value**	2	2
**Calculated density/g cm^{-3}**	1.712	1.648
**Crystal size/mm^3**	0.30 × 0.15 × 0.01	0.44 × 0.18 × 0.04
**Radiation/wavelength/pm**	Mo*K*α / 71.073	Mo*K*α / 71.073
**μ(MoKα)/mm^{-1}**	4.78	3.93
**F000**	792	656
**θ range, deg**	2.24-32.01	2.25-30.08
**h,k,l_{max}**	±15, ±15, ±15	±14, ±15, ±24
**T_{min},T_{max}**	0.607, 0.746	0.530, 0.746
**Total no. reflections**	19165	29495
**Independent reflections / R_{int}**	7403 / 0.0334	11306 / 0.0314
**Reflections with $I > 2\sigma(I)$ / R_σ**	6792 / 0.0406	10254 / 0.0386
**Data/parameters**	7403 / 384	11306 / 560
**Goodness-of-fit on F^2**	1.066	1.057
**$R1$/$wR2$ for $I > 2\sigma(I)$**	0.0232 / 0.0552	0.0250 / 0.0636
**$R1$/$wR2$ for all data**	0.0270 / 0.0565	0.0295 / 0.061
**Larg. diff. peak/hole/e Å^{-3}**	1.36 / -0.81	1.00 / -0.93

Table S3: Parameters and data from X-ray diffractometry on single crystals of [**PtLHCl**], [**PtLHCN**](yellow) and [**PtLHCN**](orange).

**Complex**	[**PtLHCl**]	[**PtLHCN**](yellow)	[**PtLHCN**](orange)
**Formula**	$C_{30}H_{26}N_5PtCl\cdot 2(CH_2Cl_2)$	$C_{31}H_{26}N_6Pt$	$C_{31}H_{26}N_6Pt\cdot CH_2Cl_2$
**Fw**	856.94	677.66	762.58
**CCDC No.**	2394607	2394606	2394609
**Temperature/K**	100(2)	120(2)	100(2)
**Crystal color**	block, yellow	block, yellow	rod, orange
**Crystal system**	triclinic	monoclinic	monoclinic
Space group	$P\bar{1}$	$P2_1/c$	$P2_1/c$
***a*/Å**	14.0359(4)	12.9827(6)	21.4307(5)
***b*/Å**	15.4018(5)	9.8751(5)	19.9835(4)
***c*/Å**	16.5005(5)	20.0451(8)	13.5344(3)
**α/°**	96.013(1)	90	90
**β/°**	109.062(1)	101.950(2)	94.012(1)
**γ/°**	105.081(1)	90	90
**V/Å^3**	3184.21(17)	2514.2(2)	5782.0(2)
***Z* value**	4	4	8
**Calculated density/g cm^{-3}**	1.788	1.790	1.752
**Crystal size/mm^3**	0.36 × 0.15 × 0.10	0.34 × 0.17 × 0.09	0.41 × 0.10 × 0.10
**Radiation/wavelength/pm**	Mo*K*α / 71.073	Mo*K*α / 71.073	Mo*K*α / 71.073
**μ(MoKα)/mm^{-1}**	4.86	5.62	5.07
**F000**	1680	1328	2992
**θ range, deg**	2.33-32.00	2.61-32.02	2.56-32.04
**h,k,l_{max}**	±20, ±22, ±24	±17, ±12, ±26	±28, ±26, ±17
**T_{min},T_{max}**	0.580, 0.746	0.328, 0.457	0.433, 0.746
**Total no. reflections**	60179	32587	83838
**Independent reflections / R_{int}**	21943 / 0.0202	5867 / 0.0231	13789 / 0.250
**Reflections with $I > 2\sigma(I)$ / R_σ**	18619 / 0.0240	5703 / 0.0202	13035 / 0.0185
**Data/parameters**	21943 / 781	5867 / 346	13789 / 745
**Goodness-of-fit on F^2**	1.039	1.151	1.080
**$R1/wR2$ for $I > 2\sigma(I)$**	0.0205 / 0.0499	0.0163 / 0.0385	0.0245 / 0.0680
**$R1/wR2$ for all data**	0.0263 / 0.0531	0.0166 / 0.0387	0.0260 / 0.0688
**Larg. diff. peak/hole/e Å^{-3}**	1.35 / -1.11	0.54 / -1.50	2.43 / -1.71

Table S4: Parameters and data from X-ray diffractometry on single crystals of [**ReLH$_2$(CO)$_3$Br**] and [**ReLH$_2$(CO)$_3$Cl**].

**Complex**	[**ReLH$_2$(CO)$_3$Br**]	[**ReLH$_2$(CO)$_3$Cl**]
**Formula**	$C_{33}H_{27}N_5O_3ReBr\cdot CHCl_3$	$C_{33}H_{27}N_5O_3ReBr\cdot 0.5\ CHCl_3\cdot 0.5\ C_6H_{12}$
**Fw**	927.07	847.81
**CCDC No.**	2394605	2394608
**Temperature/K**	101(2)	100(2)
**Crystal color**	block, colourless	plate, colourless
**Crystal system**	triclinic	triclinic
Space group	$P\bar{1}$	$P\bar{1}$
***a*/Å**	10.8176(2)	10.9546(4)
***b*/Å**	11.3687(3)	11.2680(4)
***c*/Å**	15.7988(4)	15.9604(5)
***α*/°**	79.695(1)	78.384(1)
***β*/°**	72.681(1)	72.036(1)
***γ*/°**	67.934(1)	68.128(1)
**V/Å^3**	1714.07(7)	1730.80(10)
***Z* value**	2	2
**Calculated density/g cm^{-3}**	1.796	1.627
**Crystal size/mm^3**	0.31 × 0.16 × 0.07	0.41 × 0.11 × 0.01
**Radiation/wavelength/pm**	Mo*K*α / 71.073	Mo*K*α / 71.073
**μ(MoKα)/mm^{-1}**	4.99	3.71
**F000**	904	842
**θ range, deg**	2.28-32.01	2.30-32.00
**h,k,l$_{max}$**	±16, ±16, ±23	±16, ±16, ±23
**T$_{min}$,T$_{max}$**	0.463, 0.589	0.609, 0.746
**Total no. reflections**	33291	32685
**Independent reflections / R_{int}**	11823 / 0.0182	12019 / 0.0274
**Reflections with $I > 2\sigma(I)$ / R_σ**	11480 / 0.0220	10958 / 0.0333
**Data/parameters**	11823 / 427	12019 / 528
**Goodness-of-fit on F^2**	1.053	1.160
**$R1$/$wR2$ for $I > 2\sigma(I)$**	0.0146 / 0.0356	0.0301 / 0.0645
**$R1$/$wR2$ for all data**	0.0152 / 0.0359	0.0345 / 0.0659
**Larg. diff. peak/hole/e Å^{-3}**	1.08 / -0.90	2.37 / -2.00

Structure of 1 (VeS28; CCDC-Nr.: 2394603):

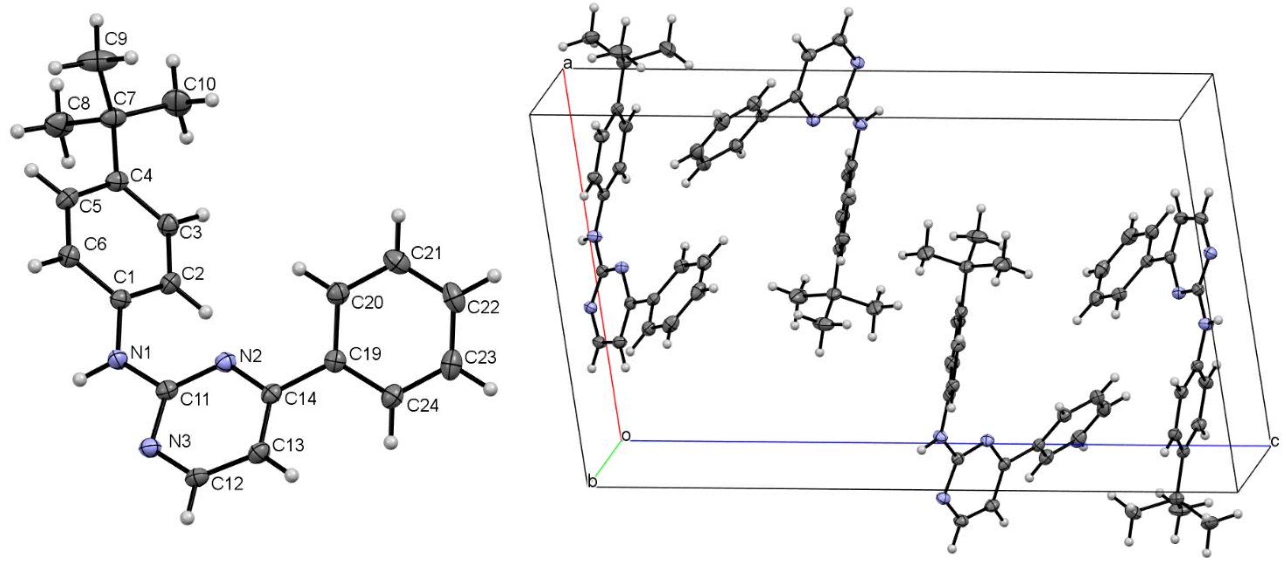

Figure S64: Molecular structure in the single crystal (left) and unit cell (right) in the crystal structure of **1**. Displacement ellipsoids are shown at 50% probability.

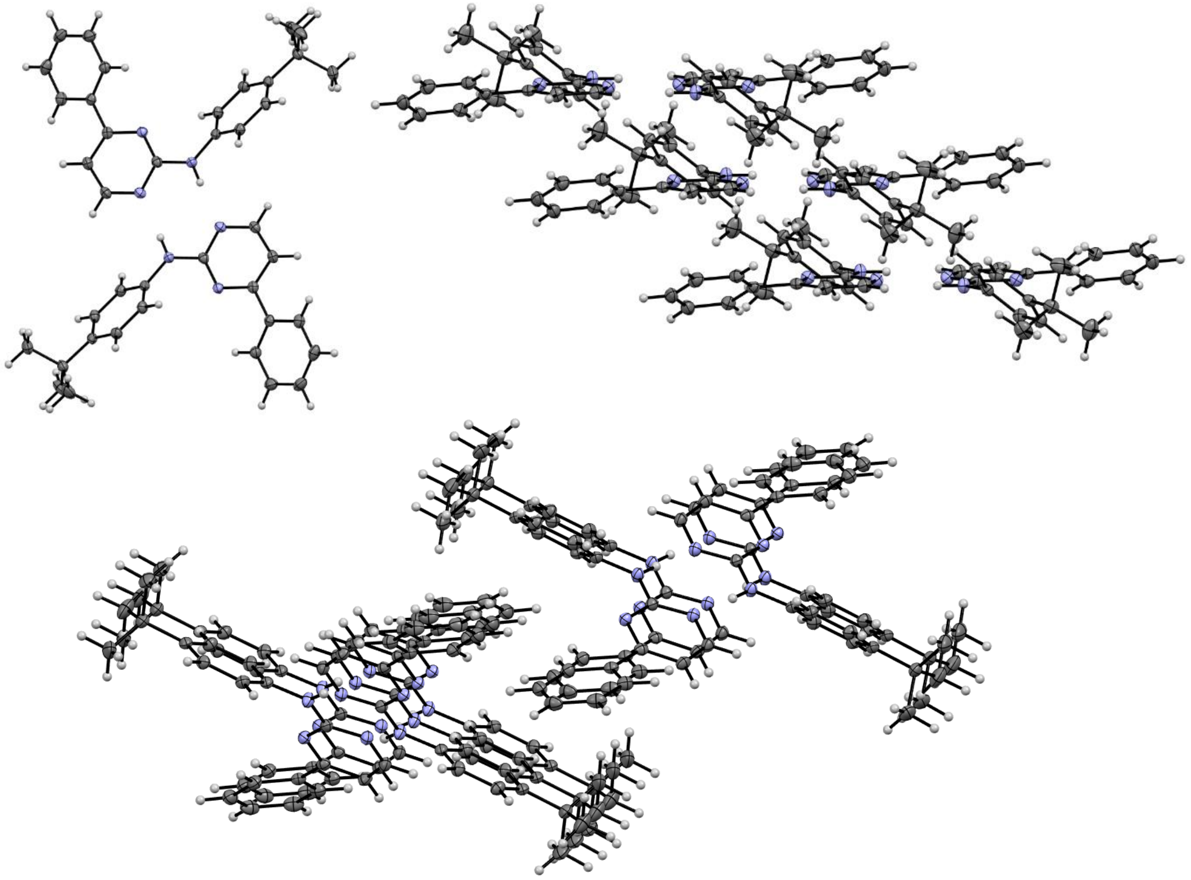

Figure S65: Display of the dimer formation *via* H-N interactions (top left), chain building of the dimers (top right) and interaction of the chains (bottom) in the crystal structure of [**PtLH$_2$cbda**]. Displacement ellipsoids are shown at 50 % probability.

Structure of LH_2 (VeS30; CCDC-Nr.: 2394610):

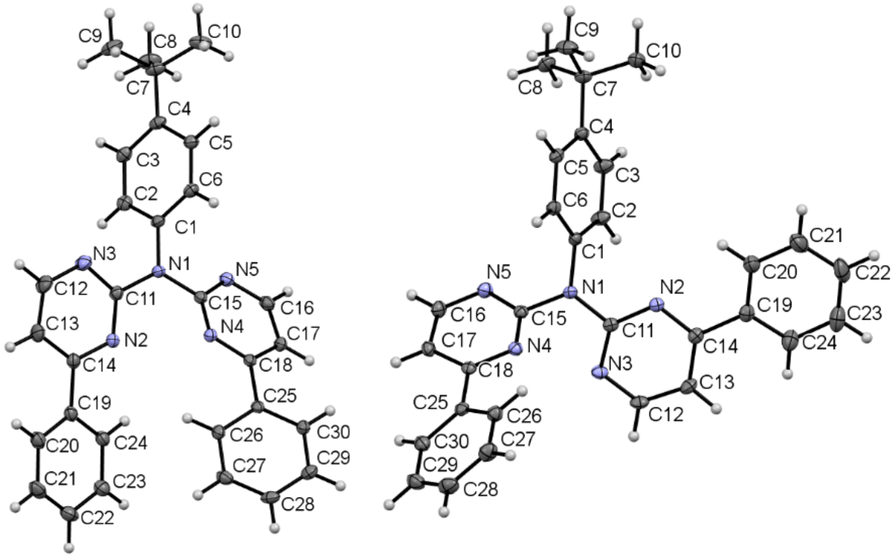

Figure S66: Molecular structure in the single crystal of molecule **A** (left) and molecule **B** (right) for the crystal structure of **LH_2**. Displacement ellipsoids are shown at 50 % probability.

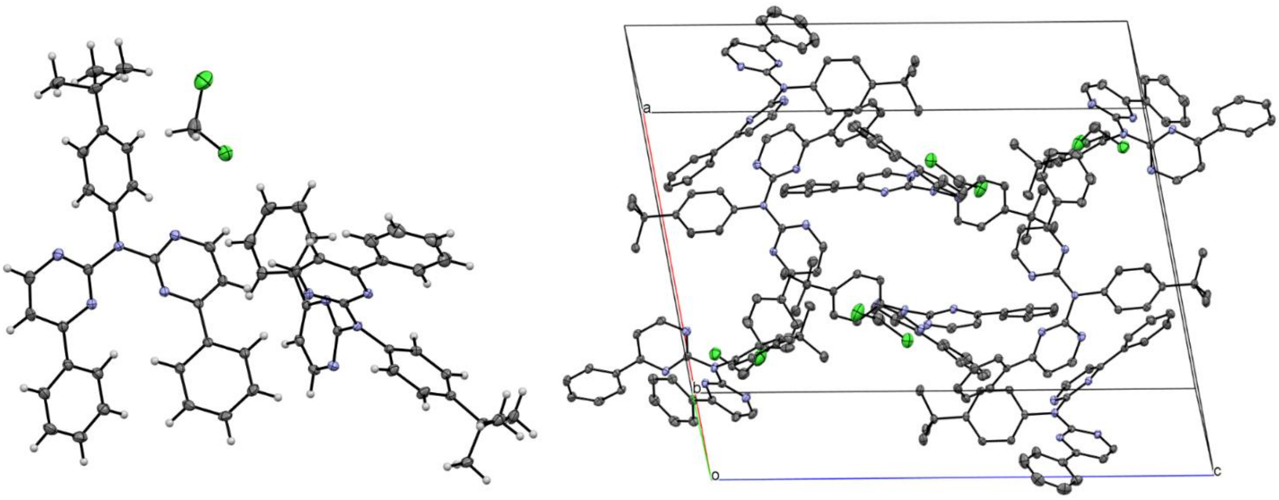

Figure S67: Asymmetric unit (left) and unit cell (right) for the crystal structure of **LH_2**. Displacement ellipsoids are shown at 50 % probability.

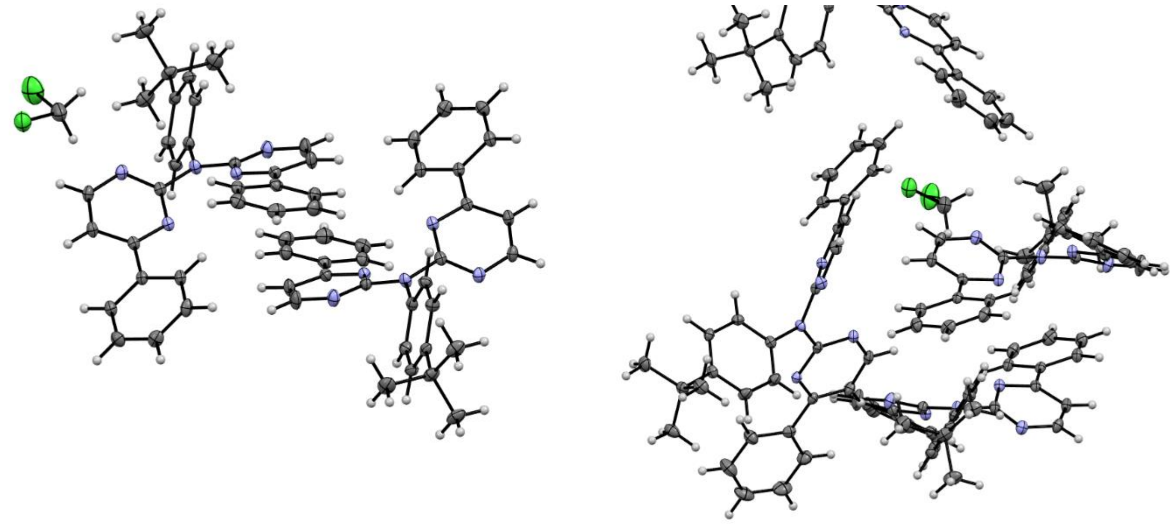

Figure S68: Display of the π-π interactions (left) and H/π-H interactions (right) in the crystal structure of **LH₂**. Displacement ellipsoids are shown at 50 % probability.

Structure of [PtLH$_2$Cl$_2$] (VeS27; CCDC-Nr.: 2394604):

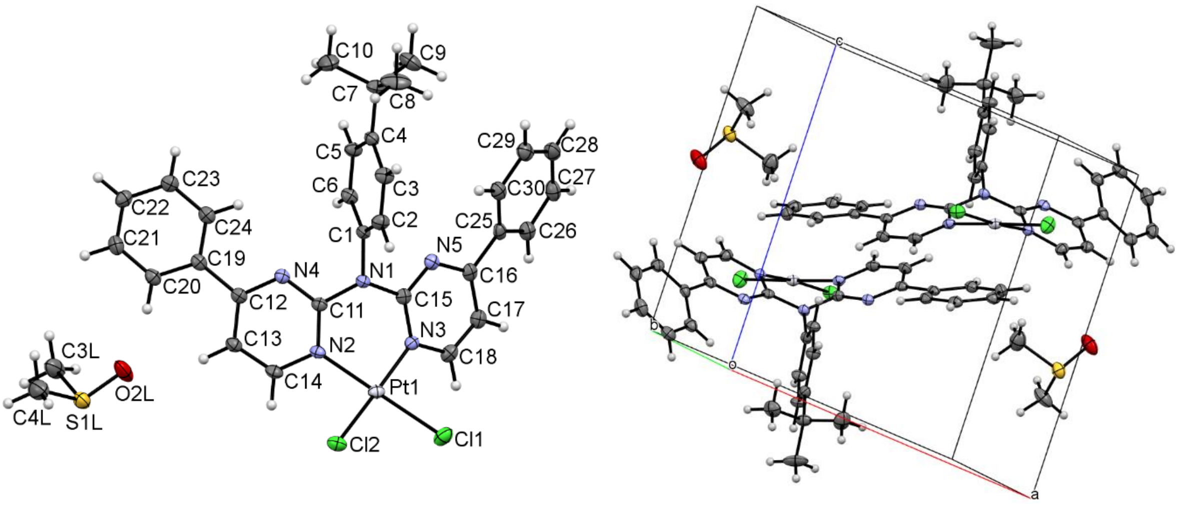

Figure S69: Molecular structure in the single crystal (left) and unit cell (right) in the crystal structure of [**PtLH$_2$Cl$_2$**]. Displacement ellipsoids are shown at 50 % probability.

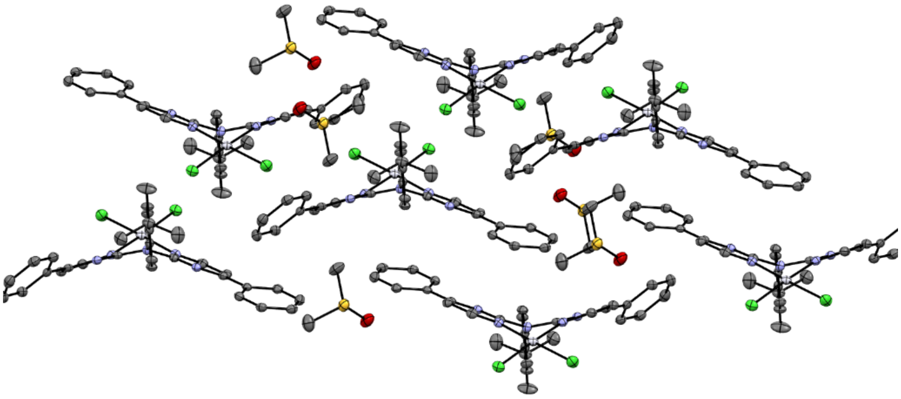

Figure S70: Display of 2D layer formation of [**PtLH$_2$L$_2$**]. Hydrogen atoms omitted for clarity. Displacement ellipsoids are shown at 50 % probability.

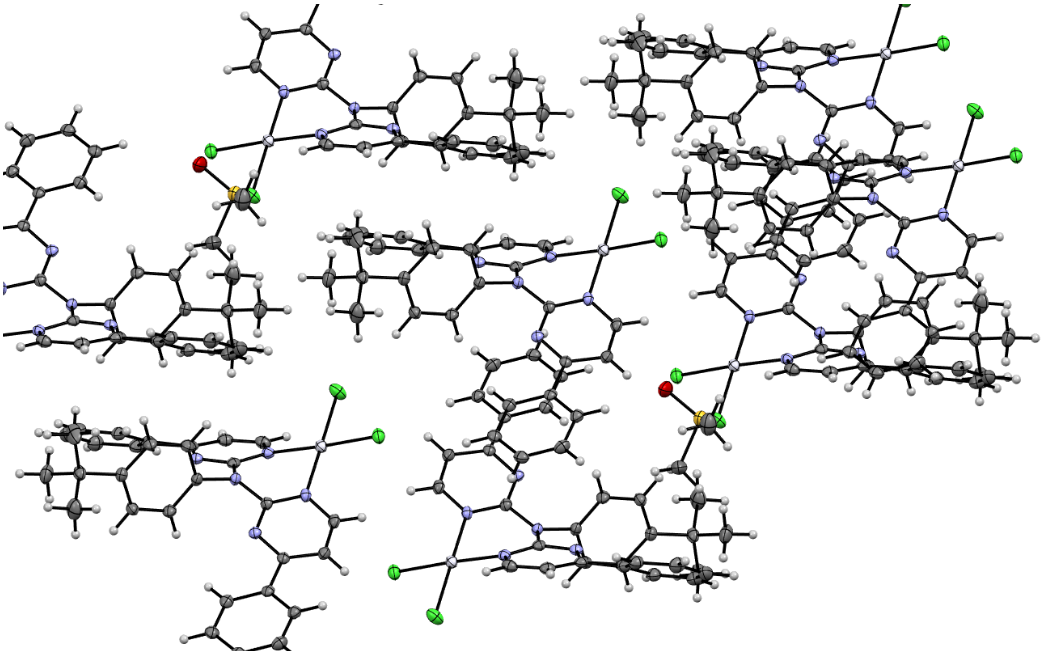

Figure S71: Display of 3D interactions of [**PtLH$_2$L$_2$**]. Displacement ellipsoids are shown at 50 % probability.

Table S5: Selected bond lengths and angles for [**PtLH$_2$Cl$_2$**].

X-Y	*d*(X-Y) in Å
Pt1-N2	2.014(2)
Pt1-N3	2.019(2)
Pt1-Cl1	2.2846(7)
Pt1-Cl2	2.2934(7)

X-Y-Z	∠(XYZ) in °
N2-Pt1-N3	88.23(9)
N3-Pt1-Cl1	90.53(6)
Cl1-Pt1-Cl2	89.95(3)
Cl2-Pt1-N2	91.35(7)
N2-Pt1-Cl1	178.47(6)
N3-Pt1-Cl2	175.55(6)

Structure of [PtLH$_2$cbda] (VeS35; CCDC-Nr.: 2394611):

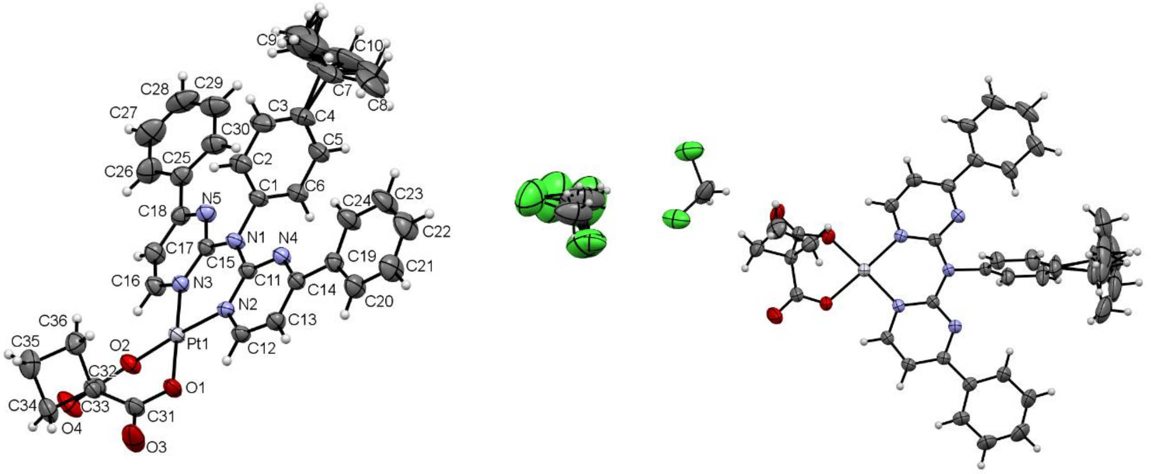

Figure S72: Molecular structure in the single crystal (left) and asymmetric unit (right) in the crystal structure of [**PtLH$_2$cbda**]. Displacement ellipsoids are shown at 50 % probability.

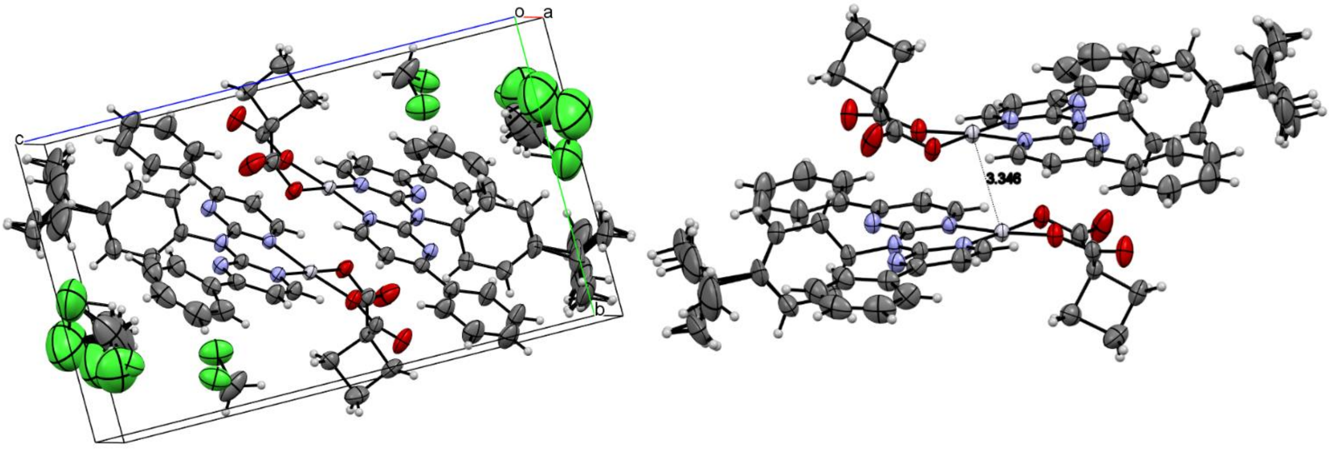

Figure S73: Unit cell (left) and display of the head-to-tail dimer (right) in the crystal structure of [**PtLH$_2$cbda**]. Displacement ellipsoids are shown at 50 % probability.

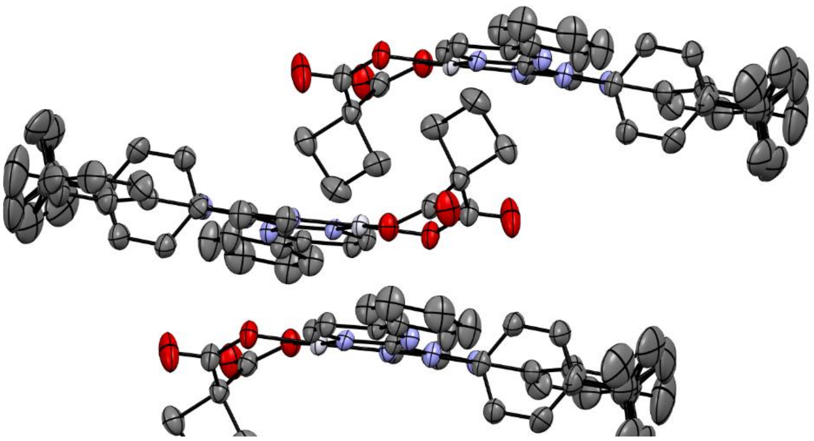

Figure S74: Display of the interactions between cbda ligands in the crystal structure of [**PtLH$_2$cbda**]. Hydrogen atoms omitted for clarity. Displacement ellipsoids are shown at 50 % probability.

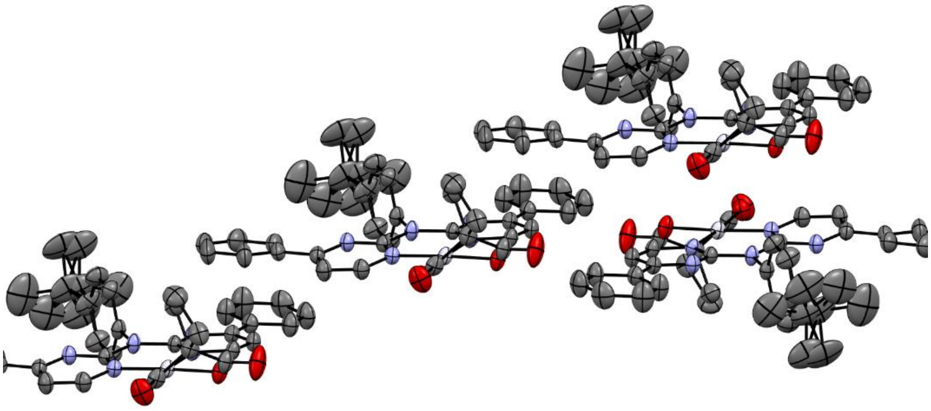

Figure S75: Display of the π-π interactions in the crystal structure of [**PtLH$_2$cbda**]. Hydrogen atoms omitted for clarity. Displacement ellipsoids are shown at 50 % probability.

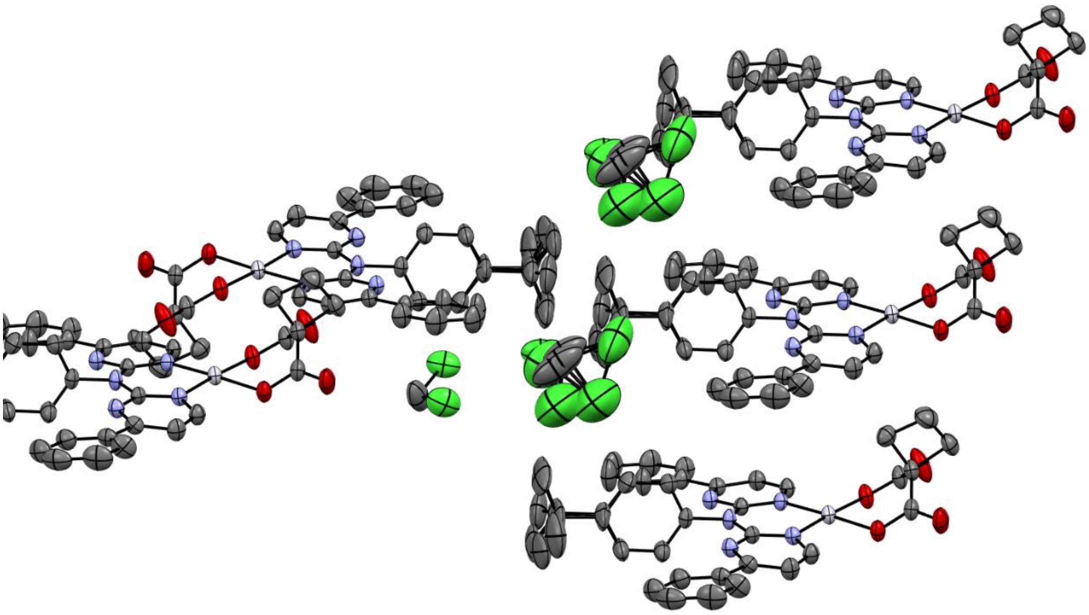

Figure S76: Display of the interactions between the ^{t}butyl groups and the incorporated CH_2Cl_2 in the crystal structure of [**PtLH$_2$cbda**]. Hydrogen atoms omitted for clarity. Displacement ellipsoids are shown at 50 % probability.

Table S6: Selected bond lengths and angles for [**PtLH$_2$cbda**].

X-Y	*d*(X-Y) in Å
Pt1-N2	2.001(2)
Pt1-N3	2.0004(19)
Pt1-O1	1.9984(8)
Pt1-O2	2.0047(17)
Pt-Pt	3.346

X-Y-Z	∠(XYZ) in °
N2-Pt1-N3	91.69(8)
N3-Pt1-O1	177.00(7)
O1-Pt1-O2	87.29(7)
O2-Pt1-N2	175.85(7)
N2-Pt1-O1	90.51(8)
N3-Pt1-O2	90.38(8)

Structure of [PtLHCl] (ViS6; CCDC-Nr.: 2394607):

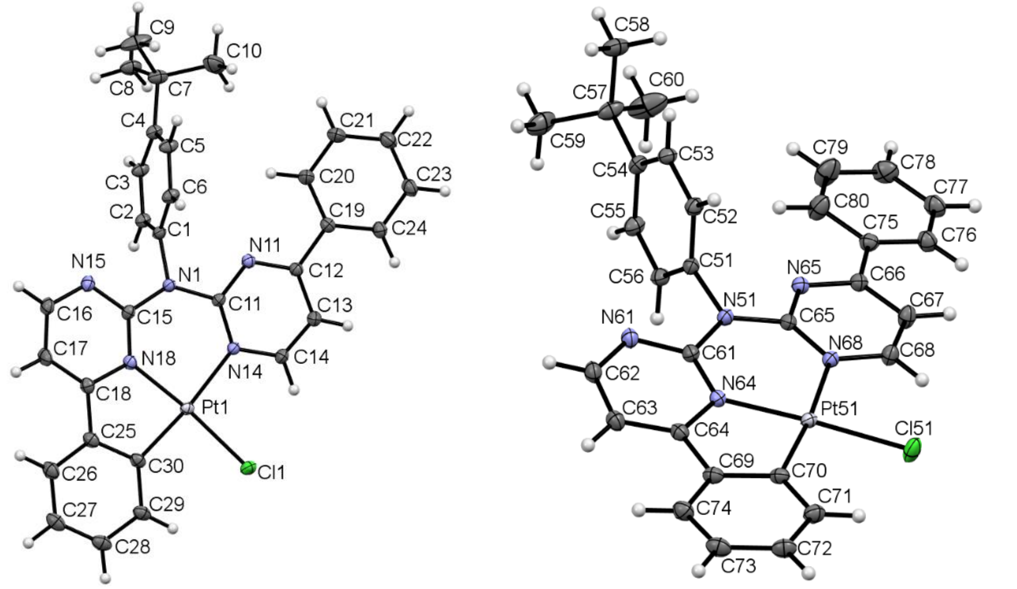

Figure S77: Molecular structure in the single crystal of molecule **A** (left) and molecule **B** (right) for the crystal structure of [**PtLHCl**]. Displacement ellipsoids are shown at 50 % probability.

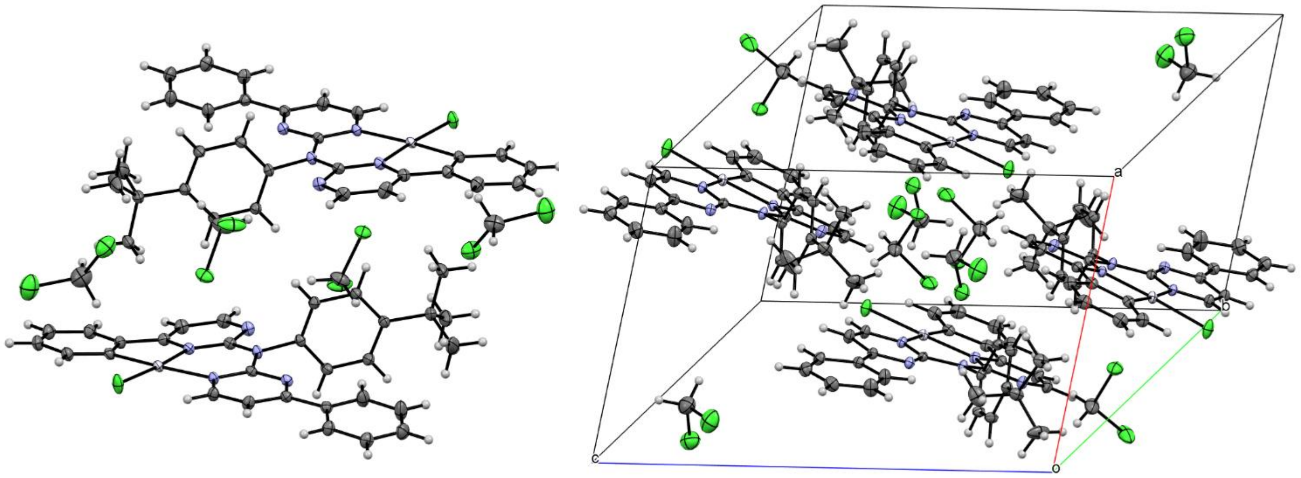

Figure S78: Asymmetric unit (left) and unit cell (right) in the crystal structure of [**PtLHCl**]. Displacement ellipsoids are shown at 50 % probability.

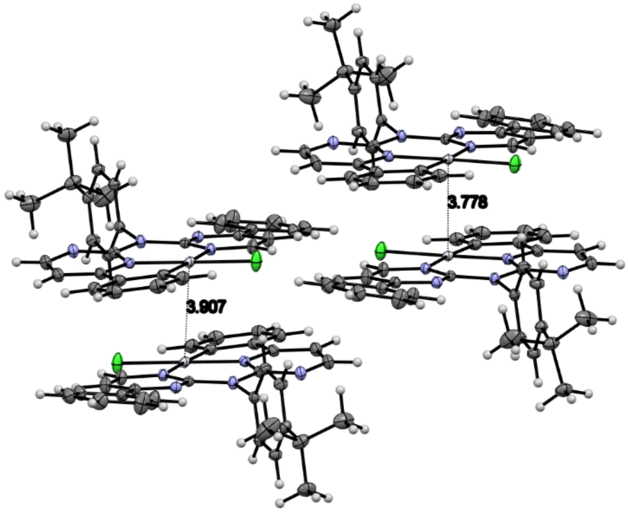

Figure S79: Display of dimer interactions and interaction between dimers in the crystal structure of [**PtLHCl**]. Displacement ellipsoids are shown at 50 % probability.

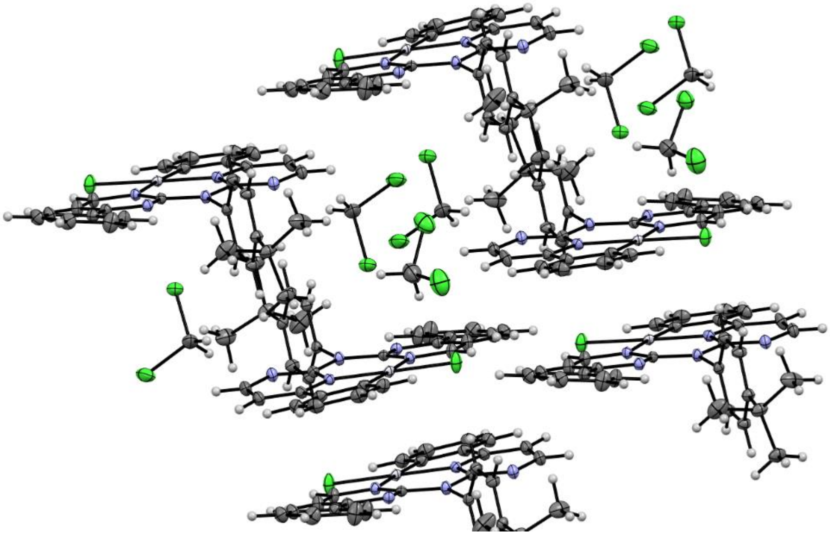

Figure S80: Display of the interaction between dimers and CH_2Cl_2 in the crystal structure of [**PtLHCl**]. Displacement ellipsoids are shown at 50 % probability.

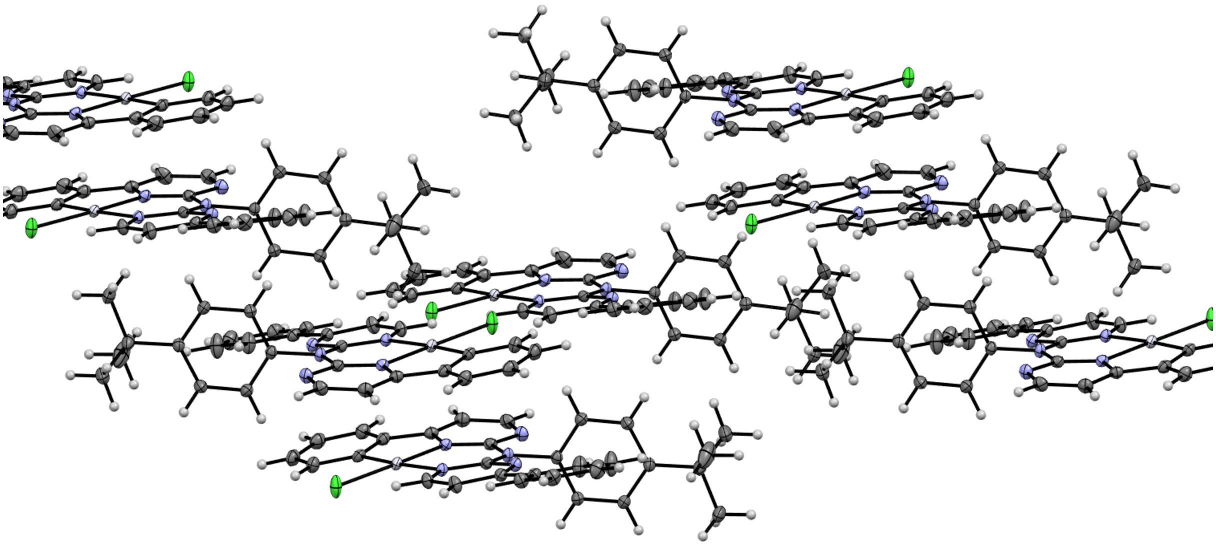

Figure S81: Display of the 3D-interaction in the crystal structure of [**PtLHCl**]. Displacement ellipsoids are shown at 50 % probability.

Table S7: Selected bond lengths and angles for [**PtLHCl**].

X-Y	*d*(X-Y) in Å
Pt1-N14	2.0939(14)
Pt1-N18	1.9908(14)
Pt1-C30	1.9839(17)
Pt1-Cl1	2.3033(4)
Molecule B	
Pt51-N64	1.9900(14)
Pt51-N68	2.0941(14)
Pt51-C70	1.9813(17)
Pt51-Cl51	2.2993(4)

X-Y-Z	∠(XYZ) in °
N14-Pt1-N18	91.64(5)
N18-Pt1-C30	82.58(6)
C30-Pt1-Cl1	92.19(5)
C30-Pt1-N14	174.21(6)
N14-Pt1-Cl1	93.59(4)
N18-Pt1-Cl1	174.76(4)
N64-Pt51-N68	91.73(5)
N68-Pt51-C70	174.37(6)
C70-Pt51-Cl51	92.14(5)
C70-Pt51-N64	82.66(6)
N64-Pt51-Cl51	174.67(4)
N68-Pt51-Cl51	93.48(4)

Structure of [PtLHCN](yellow) (ViS17; CCDC-Nr.: 2394606):

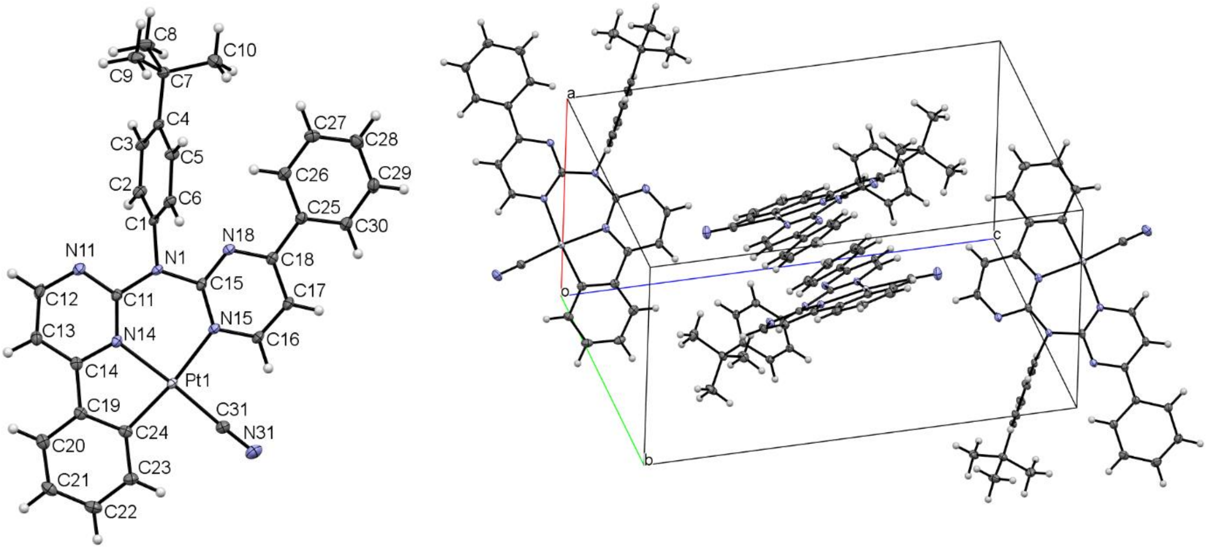

Figure S82: Molecular structure in the single crystal (left) and unit cell (right) in the crystal structure of [**PtLHCN**](yellow). Displacement ellipsoids are shown at 50 % probability.

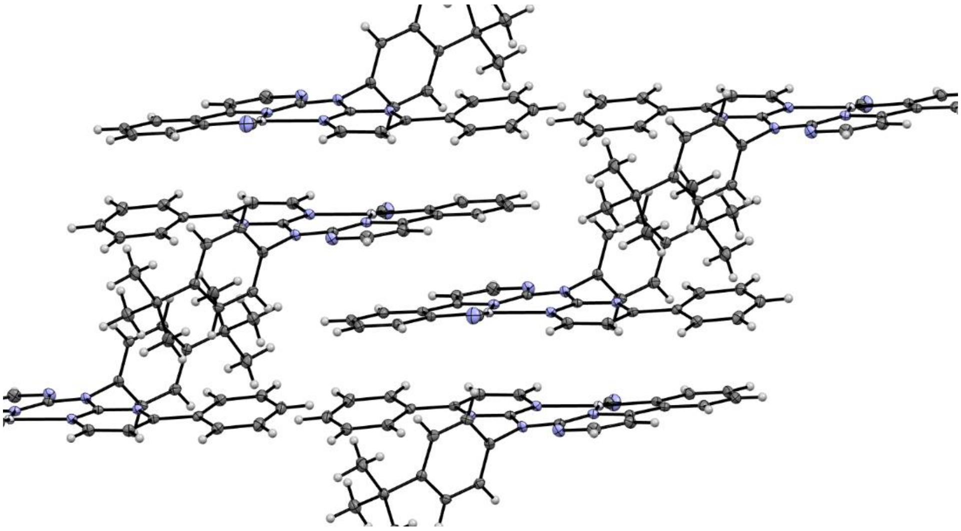

Figure S83: Display of *π-π* interactions for the layer formation in the crystal structure of [**PtLHCN**](yellow). Displacement ellipsoids are shown at 50 % probability.

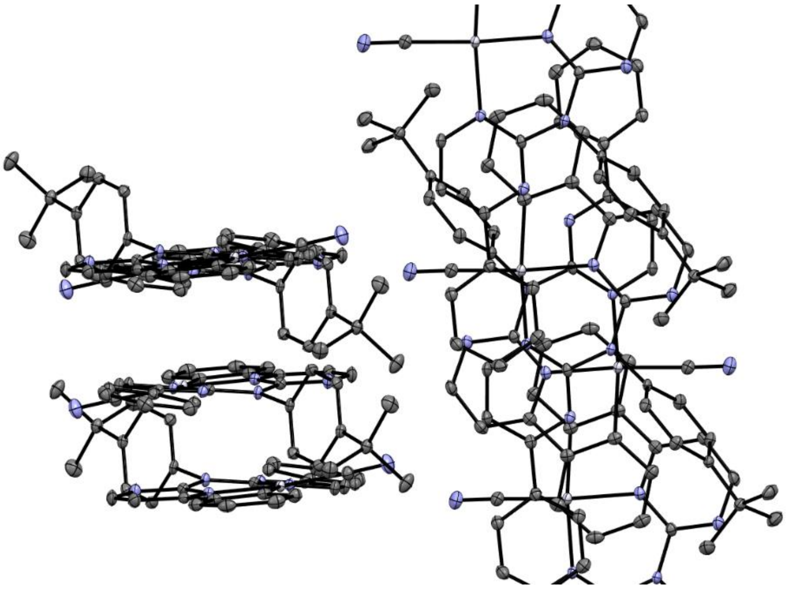

Figure S84: Display of H-N interactions between the layers in the crystal structure of [**PtLHCN**](yellow). Hydrogen atoms omitted for clarity. Displacement ellipsoids are shown at 50 % probability.

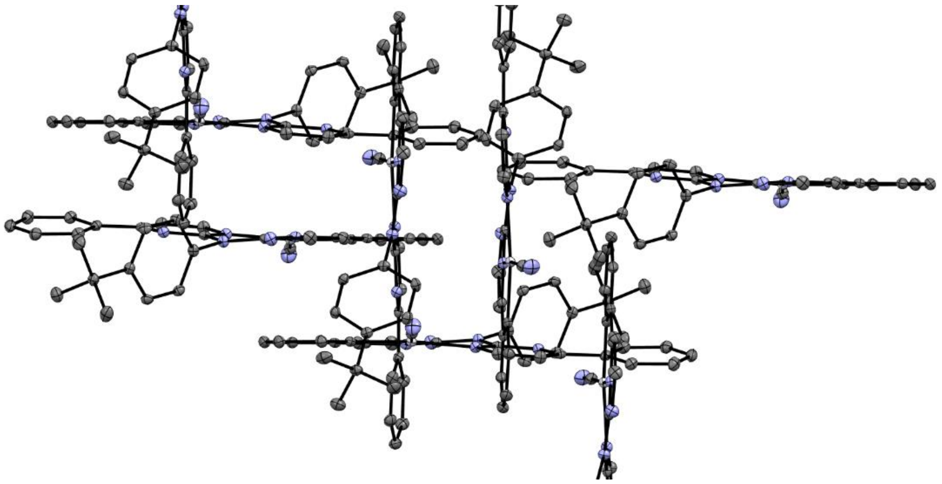

Figure S85: Display of H-N interactions between the layers in the crystal structure of [**PtLHCN**](yellow) from a different point of view. Hydrogen atoms omitted for clarity. Displacement ellipsoids are shown at 50 % probability.

Table S8: Selected bond lengths and angles for [**PtLHCN**](yellow).

X-Y	*d*(X-Y) in Å
Pt1-N14	2.0256(15)
Pt1-N15	2.0801(14)
Pt1-C24	1.9824(16)
Pt1-C31	1.9451(18)
C31-N31	1.155(3)

X-Y-Z	∠(XYZ) in °
N14-Pt1-N15	90.70(6)
N15-Pt1-C24	171.95(6)
C24-Pt1-C31	92.70(7)
C31-Pt1-N14	172.82(6)
N14-Pt1-C24	82.74(7)
N15-Pt1-C31	94.26(7)
Pt1-C31-N31	177.45(17)

Structure of [PtLHCN](orange) (ViS9; CCDC-Nr.: 2394609):

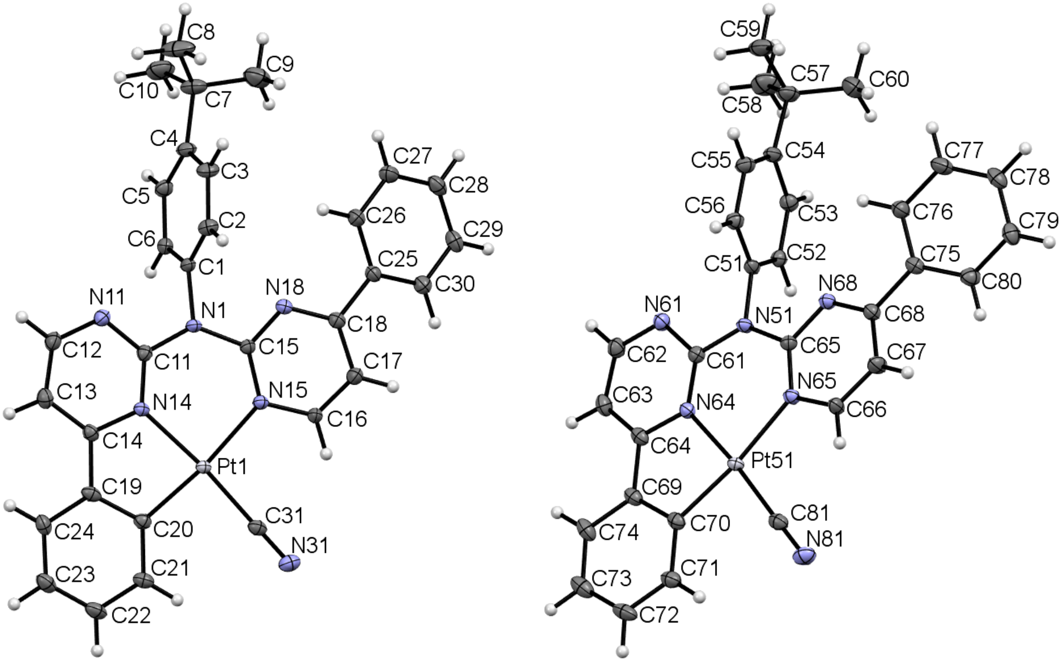

Figure S86: Molecular structure in the single crystal of molecule **A** (left) and molecule **B** (right) for the crystal structure of [**PtLHCN**](orange). Displacement ellipsoids are shown at 50 % probability.

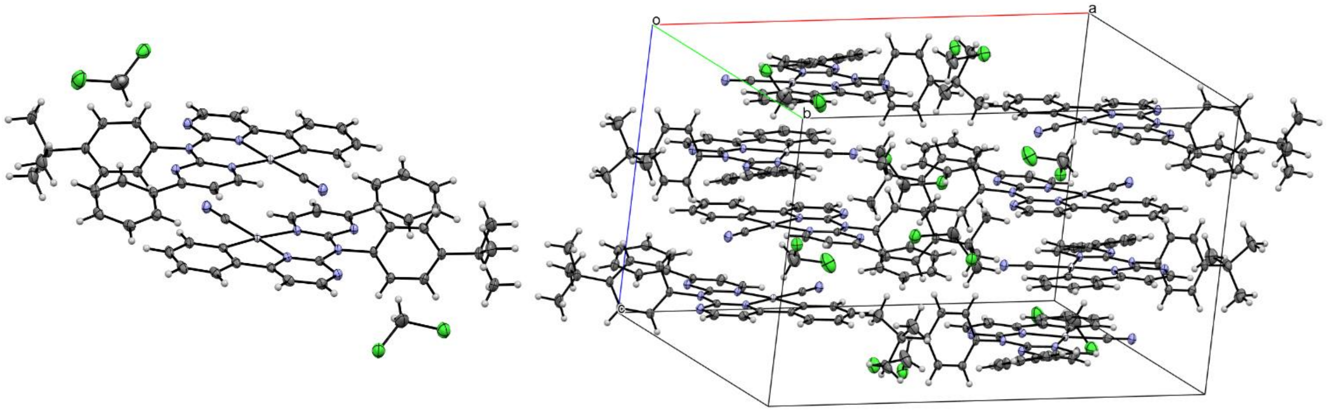

Figure S87: Asymmetric unit (left) and unit cell (right) in the crystal structure of [**PtLHCl**](orange). Displacement ellipsoids are shown at 50 % probability.

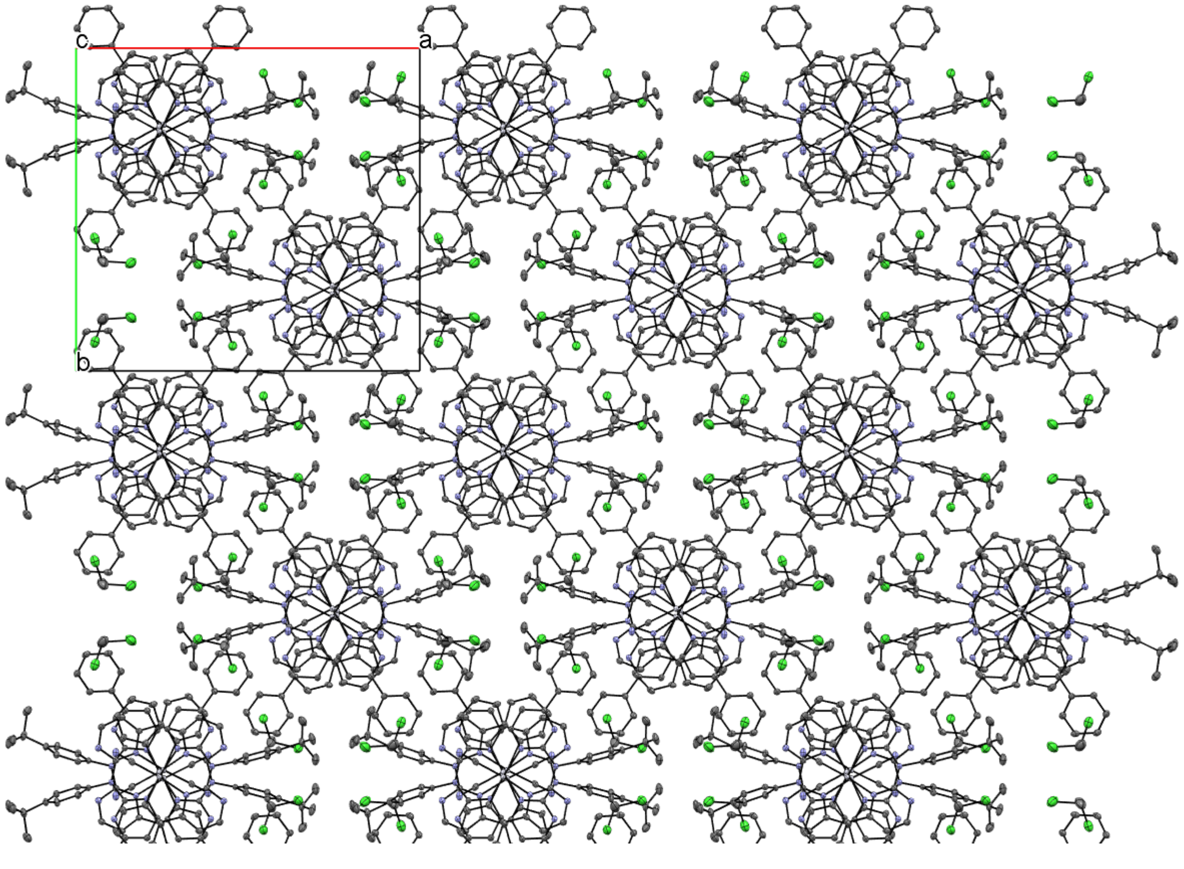

Figure S88: Display of the interactions of the Pt-Pt chains in the crystal structure of [**PtLHCN**](orange) from a different view. Hydrogen atoms omitted for clarity. Displacement ellipsoids are shown at 50 % probability.

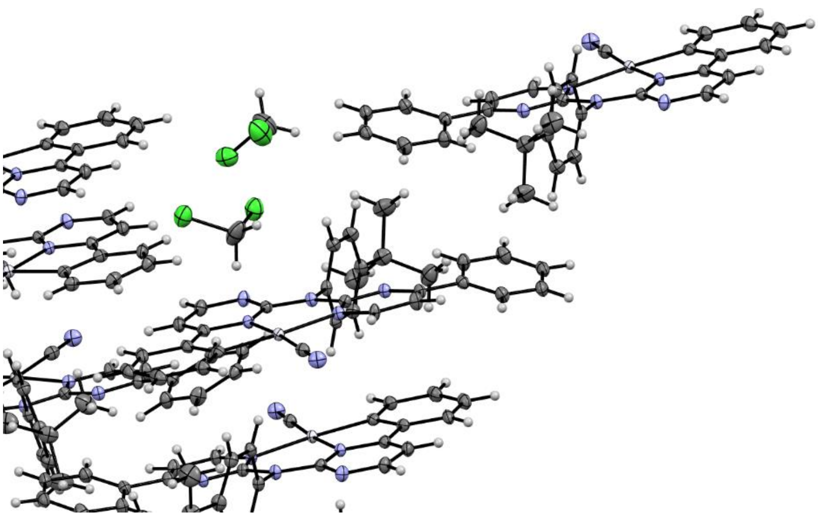

Figure S89: Additional display of the interactions of the Pt-Pt chains in the crystal structure of [**PtLHCN**](orange) from a different point of view. Displacement ellipsoids are shown at 50 % probability.

Table S9: Selected bond lengths and angles for [**PtLHCN**](orange).

X-Y	*d*(X-Y) in Å	X-Y-Z	∠(XYZ) in °
Pt1-N14	2.034(2)	N14-Pt1-N15	90.33(9)
Pt1-N15	2.084(2)	N15-Pt1-C20	172.95(10)
Pt1-C20	1.987(3)	C20-Pt1-C31	92.12(11)
Pt1-C31	1.947(3)	C31-Pt1-N14	174.71(10)
C31-N31	1.154(4)	N14-Pt1-C20	82.70(10)
		N15-Pt1-C31	94.87(10)
Molecule B		Pt1-C31-N31	178.2(3)
Pt1-N64	2.032(2)	N64-Pt1-N65	90.39(9)
Pt1-N65	2.085(2)	N65-Pt1-C70	172.99(10)
Pt1-C70	1.985(3)	C70-Pt1-C81	92.00(12)
Pt1-C81	1.952(3)	C81-Pt1-N64	174.39(10)
C81-N81	1.153(4)	N64-Pt1-C70	82.66(10)
Pt1-Pt51	3.389	N65-Pt1-C81	94.98(10)
		Pt51-C81-N81	187.7(3)

Structure of [$ReLH_2(CO)_3Br$] (ViS11; CCDC-Nr.: 2394605):

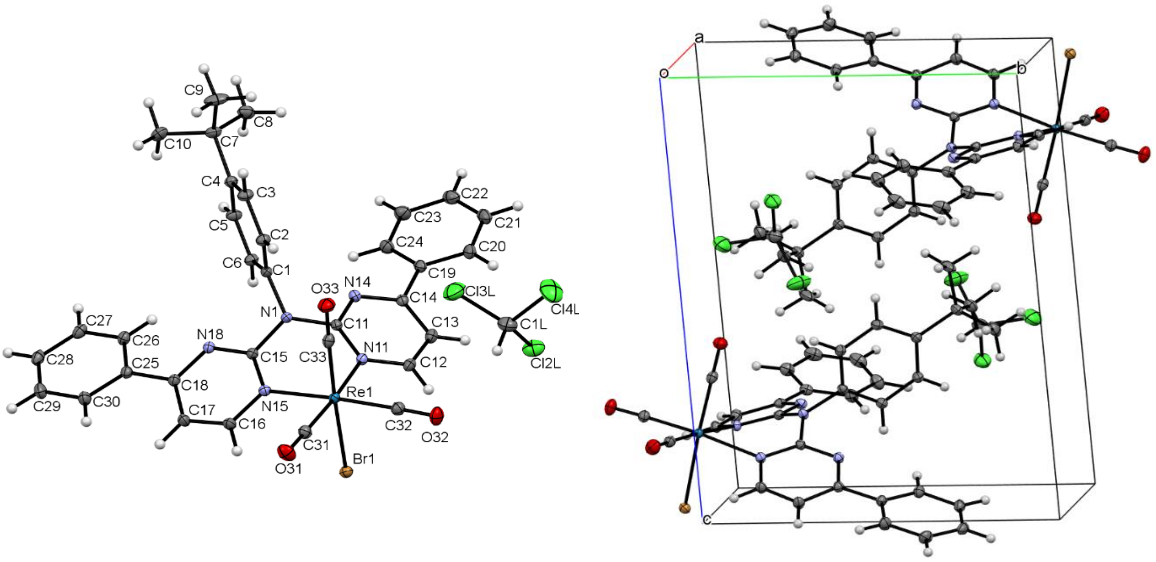

Figure S90: Molecular structure in the single crystal (left) and unit cell (right) in the crystal structure of [$\mathbf{ReLH_2(CO)_3Br}$]. Displacement ellipsoids are shown at 50 % probability.

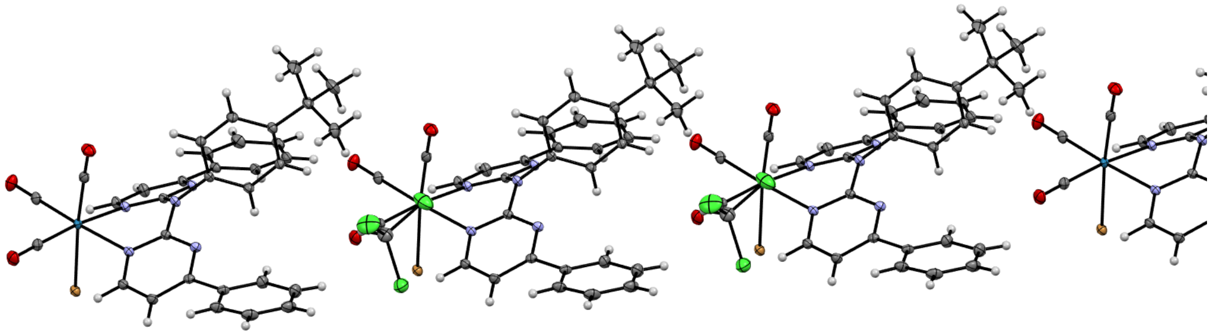

Figure S91: Display of the formation of 1D-complex chains in the crystal structure of [**$ReLH_2(CO)_3Br$**]. Displacement ellipsoids are shown at 50 % probability.

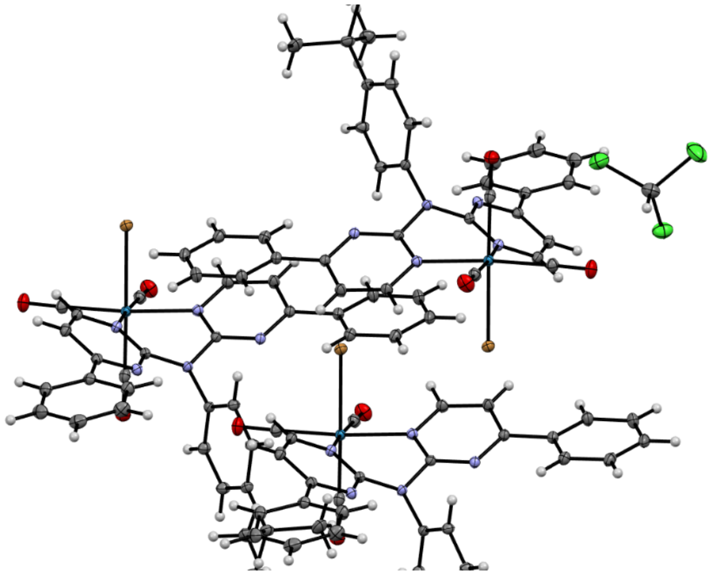

Figure S92: Display of the interactions of 1D-complex chains in the crystal structure of [**$ReLH_2(CO)_3Br$**]. Displacement ellipsoids are shown at 50 % probability.

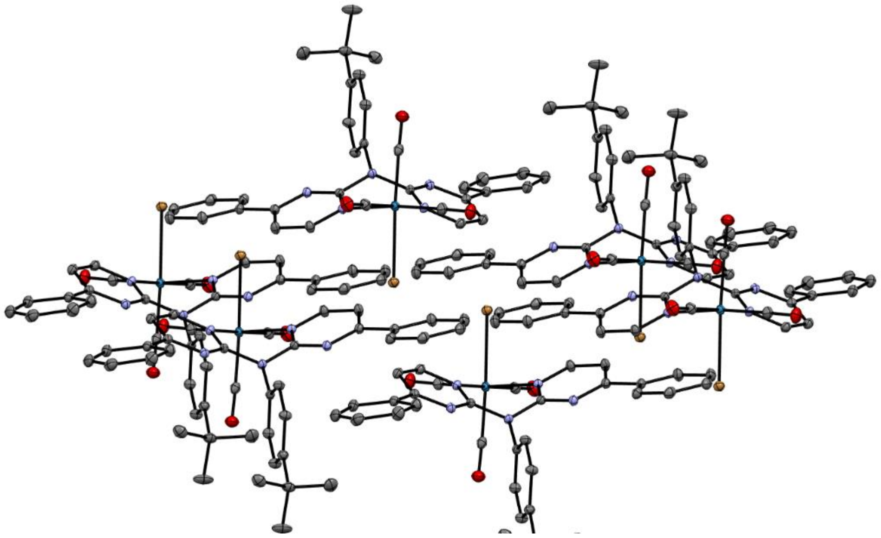

Figure S93: Display of the interactions of two 1D-complex chains in the crystal structure of [**$ReLH_2(CO)_3Br$**]. Hydrogen atoms are omitted for clarity. Displacement ellipsoids are shown at 50 % probability.

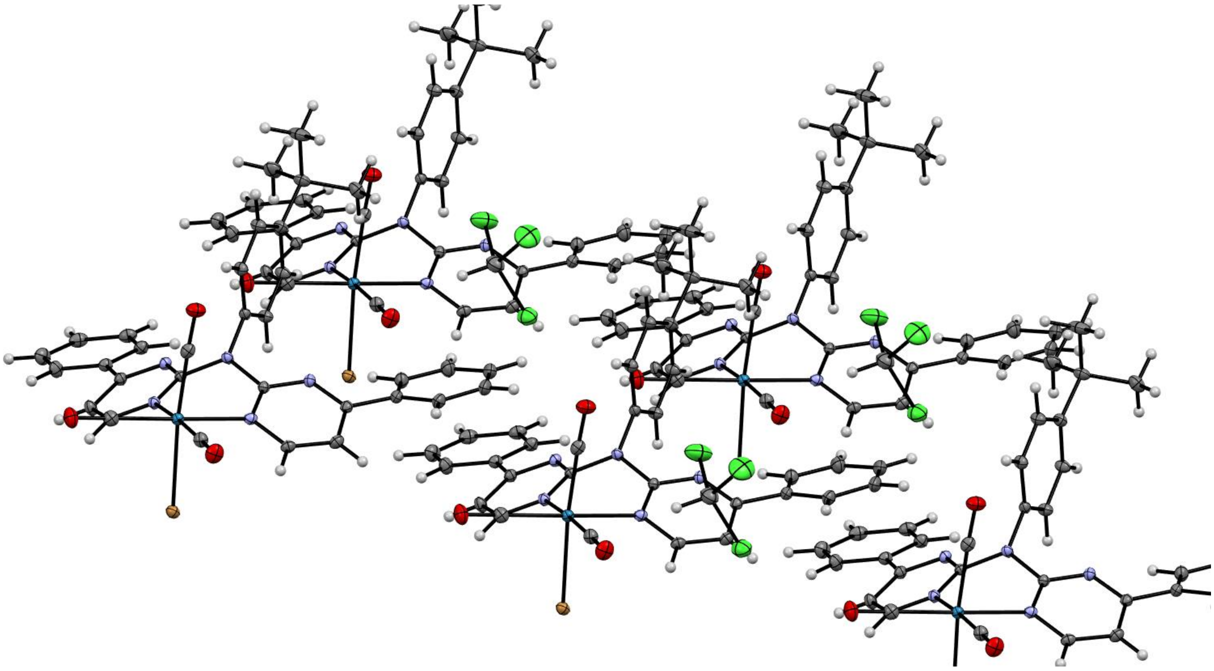

Figure S94: Display of the interactions of two 1D-complex chains in the crystal structure of [**ReLH$_2$(CO)$_3$Br**]. Displacement ellipsoids are shown at 50 % probability.

Table S10: Selected bond lengths and angles for [**ReLH$_2$(CO)$_3$Br**].

X-Y	*d*(X-Y) in Å
Re1-N11	2.1780(11)
Re1-N15	2.1767(10)
Re1-Br1	2.6075(1)
Re1-C31	1.9169(13)
Re1-C32	1.9125(13)
Re1-C33	1.9090(13)
C31-O31	1.1535(17)
C32-O32	1.1567(16)
C33-O33	1.1495(17)

X-Y-Z	∠(XYZ) in °
N11-Re1-N15	81.66(4)
N11-Re1-Br1	85.56(3)
N11-Re1-C31	175.27(5)
N11-Re1-C32	95.80(5)
N11-Re1-C33	88.68(5)
N15-Re1-Br1	84.51(3)
N15-Re1-C31	93.61(5)
N15-Re1-C32	174.02(5)
N15-Re1-C33	94.85(5)
Br1-Re1-C31	93.91(4)
Br1-Re1-C32	89.91(4)
Br1-Re1-C33	174.25(4)
C31-Re1-C32	88.90(5)
C31-Re1-C33	91.84(6)
C32-Re1-C33	90.48(5)
Re1-C31-O31	178.72(12)
Re1-C32-O32	178.29(12)
Re1-C33-O33	177.42(12)

Structure of [ReLH2(CO)3Cl] (VeS31; CCDC-Nr.: 2394608):

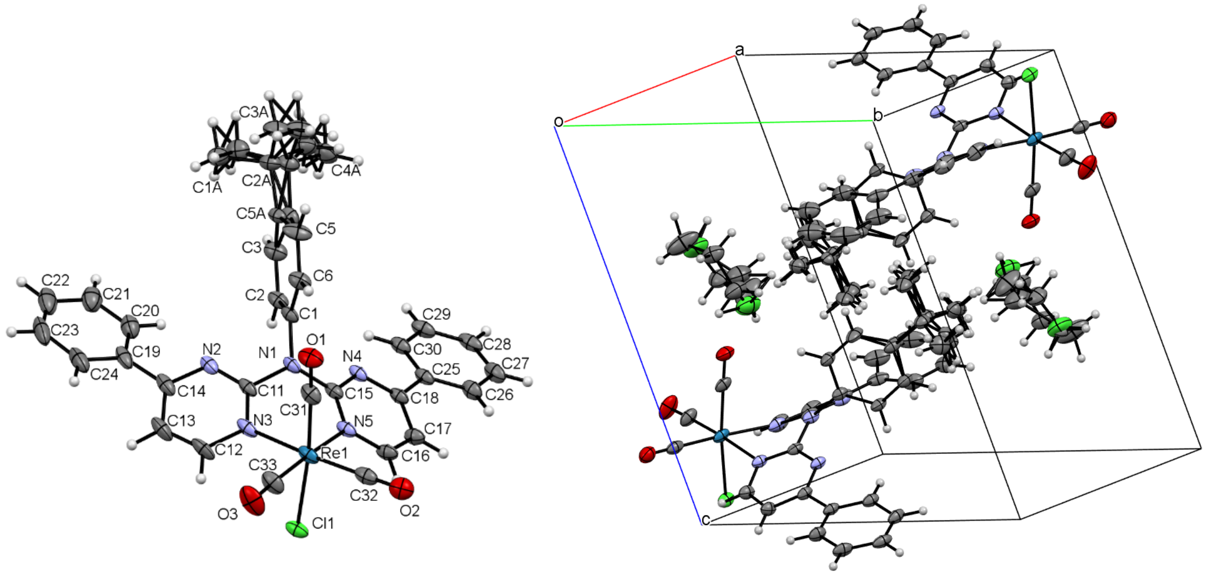

Figure S95: Molecular structure in the single crystal (left) and unit cell (right) in the crystal structure of [**ReLH2(CO)3Cl**]. Displacement ellipsoids are shown at 50 % probability.

Table S11: Selected bond lengths and angles for [**ReLH2(CO)3Br**].

X-Y	*d*(X-Y) in Å
Re1-N3	2.176(3)
Re1-N5	2.1786(19)
Re1-Cl1	2.4706(6)
Re1-C31	1.911(3)
Re1-C32	1.914(3)
Re1-C33	1.919(3)
C31-O1	1.145(3)
C32-O2	1.157(4)
C33-O3	1.148(3)

X-Y-Z	∠(XYZ) in °
N3-Re1-N5	80.91(8)
N3-Re1-Cl1	84.48(6)
N3-Re1-C31	88.67(11)
N3-Re1-C32	174.74(9)
N3-Re1-C33	97.43(11)
N5-Re1-Cl1	84.26(6)
N5-Re1-C31	94.81(9)
N5-Re1-C32	93.85(10)
N5-Re1-C33	174.27(10)
Cl1-Re1-C31	173.15(9)
Cl1-Re1-C32	95.49(8)
Cl1-Re1-C33	90.13(9)
C31-Re1-C32	91.34(12)
C31-Re1-C33	90.62(12)
C32-Re1-C33	87.83(12)
Re1-C31-O1	177.7(3)
Re1-C32-O2	179.0(2)
Re1-C33-O3	178.0(3)

III. Photophysical characterization

Table S12: Complete photoluminescence data, as well as exited state lifetime data for each complex in DCM at 298 K and in frozen glassy matrix of DCM/MeOH (V:V = 1:1) at 77 K. For multiexponential decays, the amplitude-weighted average lifetimes are given as well as the different components in square brackets with relative amplitudes as percentages in parentheses.

Complex	Medium (*T* / K)	λ_{em} / nm	λ_{exc} / nm	τ_{av} / µs	*Φ*
[**PtLH$_2$Cl$_2$**]	DCM, air (298)	n.d.	n.d.	n.d.	< 0.02
	DCM, Ar (298)			n.d.	< 0.02
	Glassy matrix (77)	n.d.	n.d.	n.d.	n.d.
[**PtLH$_2$Gly**]	DCM, air (298)	501 sh, 531	384 sh, 352	n.d.	< 0.02
	DCM, Ar (298)			2.62 ± 0.03 [4.3 ± 0.2 (36); 1.7 ± 0.1 (64)]	< 0.02
	Glassy matrix (77)	452, 484, 514	352sh, 327	121.8 ± 0.7 [210 ± 20 (11); 101 ± 4 (65); 24 ± 2 (24)]	n.d.
[**PtLH$_2$cbda**]	DCM, air (298)	n.d.	n.d.	n.d.	< 0.02
	DCM, Ar (298)			n.d.	< 0.02
	Glassy matrix (77)	450, 486, 518	357sh, 344, 328	120.0 ± 0.4 [145 ± 7 (53); 90 ± 10 (48)]	n.d.
[**PtLH$_2$Tsgly**]	DCM, air (298)	n.d.	n.d.	n.d.	< 0.02
	DCM, Ar (298)			n.d.	< 0.02
	Glassy matrix (77)	468 sh, 496	358, 322	86.3 ± 0.2 [100.8 ± 0.5 (74); 46 ± 2 (26)]	n.d.
[**PtLHCl**]	DCM, air (298)	509, 537	442sh, 392, 346	0.02306 ± 0.00001 [0.02319 ± 0.00001 (96); 0.0041 ± 0.0006 (4)]	< 0.02
	DCM, Ar (298)			0.02346 ± 0.00005 [0.02430 ± 0.00006 (94); 0.009 ± 0.002 (6)]	< 0.02
	Glassy matrix (77)	484, 522, 556	412sh, 384, 344, 296	8.02 ± 0.02 [8.56 ± 0.05 (84); 5.3 ± 0.3 (16)]	n.d.
[**PtLHCN**]	DCM, air (298)	494, 522	418sh, 398, 346, 310	1.181 ± 0.001	0.03
	DCM, Ar (298)			10.34 ± 0.02	0.55 ± 0.03
	Glassy matrix (77)	477, 510, 550	410sh, 388, 338, 300	19.26 ± 0.03	n.d.
[**ReLH$_2$(CO)$_3$Br**]	DCM, air (298)	600	362, 302	0.1024 ± 0.0004	< 0.02
	DCM, Ar (298)			0.1373 ± 0.0003	< 0.02
	Glassy matrix (77)	520	350, 298	28.28 ± 0.06 [37.5 ± 0.5 (44); 21.0 ± 0.4 (56)]	n.d.
[**ReLH$_2$(CO)$_3$Cl**]	DCM, air (298)	596	376	0.1146 ± 0.0002	< 0.02
	DCM, Ar (298)			0.1414 ± 0.0003	< 0.02
	Glassy matrix (77)	522	346, 292	26.33 ± 0.03 [36.9 ± 0.1 (39); 19.5 ± 0.1 (61)]	n.d.
[**ReLH$_2$(CO)$_3$CN**]	DCM, air (298)	584	370	0.4046 ± 0.0008	< 0.02
	DCM, Ar (298)			0.721 ± 0.02	< 0.02
	Glassy matrix (77)	512	346, 292	49.5 ± 0.1 [75 ± 1 (35); 35.6 ± 0.6 (65)]	n.d.

All solvents used were of spectrometric grade (Uvasol®, Merck).UV-visible absorption spectra were measured with a Shimadzu UV-VIS spectrophotometer UV-1900i equipped with a 20-W halogen lamp and deuterium lamp, silicon photodiode detector, LO-RAY-LIGH grade blazed holographic grating in Czerny-Turner mounting monochromator with a spectral bandwidth of 1 nm in the range of 190 nm to 1100 nm, an employing LabSolutions UV-Vis software.

Photoluminescence quantum yields were measured with a Hamamatsu Photonics absolute PL quantum yield measurement system (C9920-02) equipped with a L9799-01 CW Xe light source (150 W), a monochromator, a C7473 photonic multi-channel analyser, an integrating sphere and employing U6039-05 software (Hamamatsu Photonics, Ltd., Shizuoka, Japan).

Steady-state excitation and emission spectra were recorded on a FluoTime 300 spectrometer from PicoQuant equipped with a 300 W ozone-free Xe lamp (200-1100 nm), a 10 W Xe flash-lamp (200-1100 nm, pulse width ca. 1 µs) with repetition rates of 1 – 300 Hz, a single-grating excitation monochromator (Czerny-Turner type, grating with 1200 lines/mm, blaze wavelength: 300 nm), diode lasers (pulse width < 20 ps) operated by a computer-controlled laser driver PDL-820 "Sepia II" (repetition rate up to 80 MHz, burst mode for slow and weak decays), two emission monochromators (Czerny-Turner, selectable between single-grating blazed at 500 nm with 2.7 nm/mm dispersion and 1200 lines/mm, or single-grating blazed at 1250 nm with 5.4 nm/mm dispersion and 600

lines/mm) with adjustable slit width between 20 µm and 4 mm, Glan-Thompson polarizers for excitation (after the Xe-lamps) and emission (after the sample). A Peltier-thermostatized sample holder from Quantum Northwest (-40 °C – 105 °C), along two detectors (namely a PMA Hybrid-40 from PicoQuant with transit time spread FWHM < 120 ps, 300 – 720 nm, or a R5509-43 NIR detector with transit time spread FWHM 1.5 ns, 300-1400 nm from Hamamatsu) were used. Steady-state spectra and photoluminescence lifetimes were recorded in TCSPC mode by a PicoHarp 300 (minimum base resolution 4 ps) or in MCS mode by a TimeHarp 260 (where up to several ms can be traced). Emission and excitation spectra were corrected for source intensity (lamp and grating) by standard correction curves. For samples with lifetimes in the ns order, an instrument response function calibration (IRF) was performed using a diluted Ludox® dispersion. Lifetime analysis was performed using the commercial EasyTau 2 software (PicoQuant). The quality of the fit was assessed by minimizing the reduced chi squared function (χ2) and visual inspection of the weighted residuals and their autocorrelation.

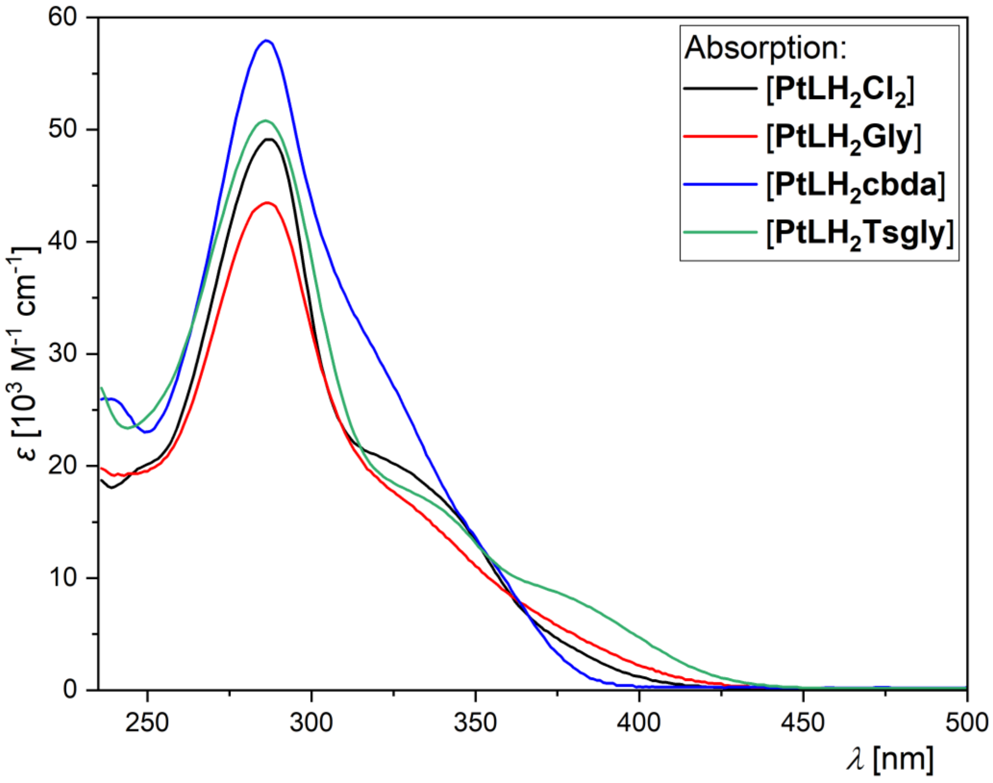

Figure S96: Molar absorption coefficients as a function of wavelength for [**$PtLH_2Cl_2$**] (black), [**$PtLH_2Gly$**] (red), [**$PtLH_2cbda$**] (blue) and [**$PtLH_2Tsgly$**] (green) (validity range: $c = 1x10^{-5} – 5x10^{-7}$ M in DCM at 298 K).

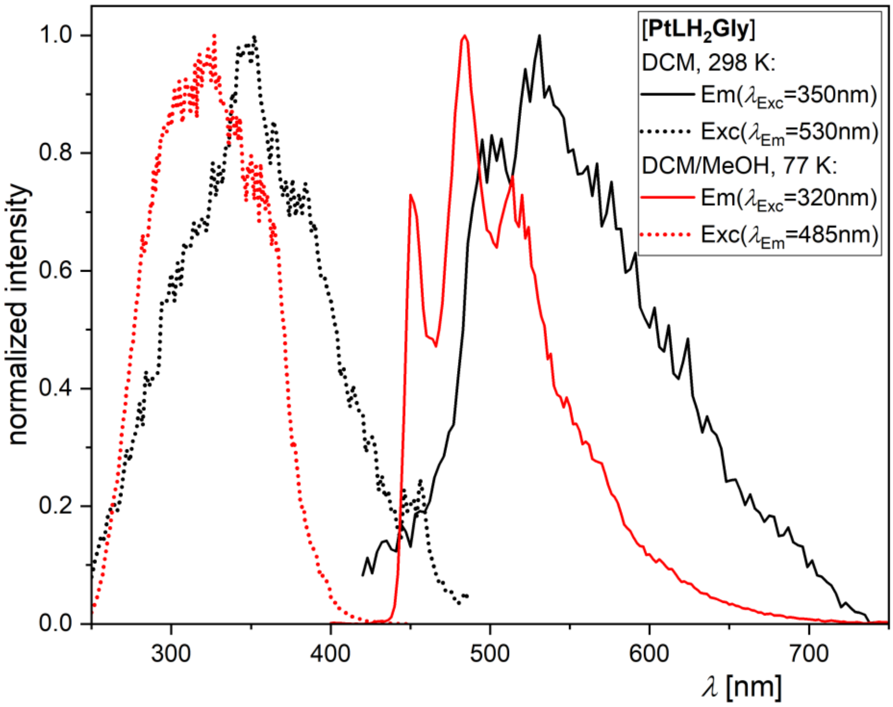

Figure S97: Excitation (dotted line) and emission spectra (solid line) of [**PtLH$_2$Gly**] at 298 K (black) in liquid DCM and at 77 K (red) in a frozen glassy DCM/MeOH matrix (V:V = 1:1). All solutions were optically diluted (A < 0.1). Spectra normalized to the highest intensity.

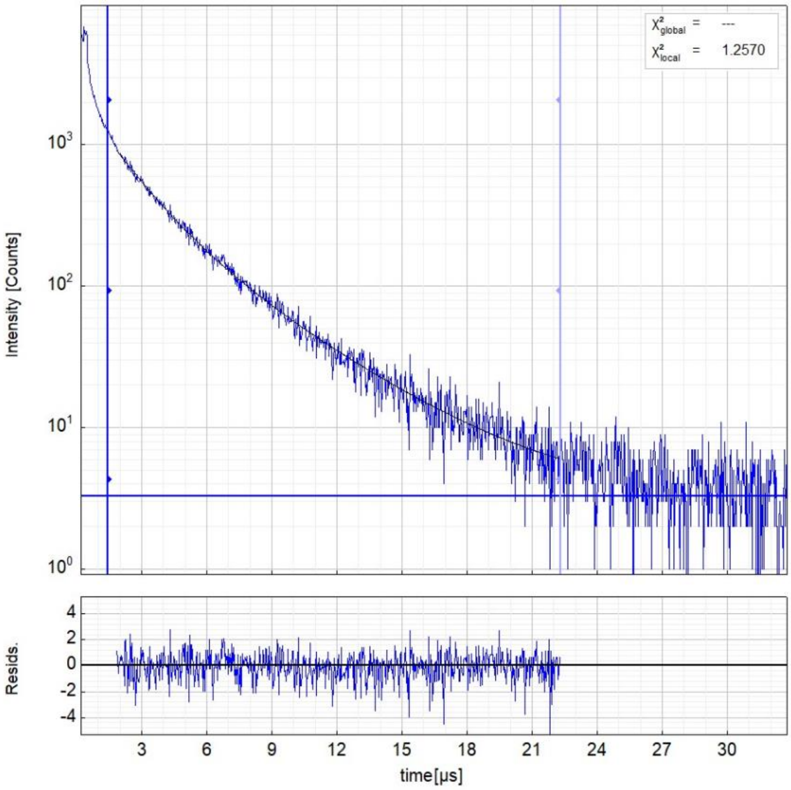

Parameter	Value	Δ	δ
A_1[kCnts/Chnl]	0.332	±0.039	12%
τ_1[ns]	4 260	±170	3.9%
I_1[kCnts]	44.1	±3.6	8.1%
A_{Rel1}[%]	36.4	±4.0	11%
I_{Rel1}[%]	59.2	±5.0	8.3%
A_2[kCnts/Chnl]	0.580	±0.033	5.6%
τ_2[ns]	1 690	±110	6.5%
I_2[kCnts]	30.5	±3.8	12%
A_{Rel2}[%]	63.7	±4.0	6.3%
I_{Rel2}[%]	40.9	±5.0	12%
$Bkgr_{Dec}$[kCnts]	0.0033	---	---
τ_{AvInt}[ns]	3 204	±25	0.8%
τ_{AvAmp}[ns]	2 620	±25	0.9%

Figure S98: Left: Raw (experimental) time-resolved photoluminescence decay of [**PtLH$_2$Gly**] in liquid DCM (Ar-purged) at 298 K, including the residuals (λ_{exc} = 376.7 nm, λ_{em} = 530 nm). Right: Fitting parameters including pre-exponential factors and confidence limits.

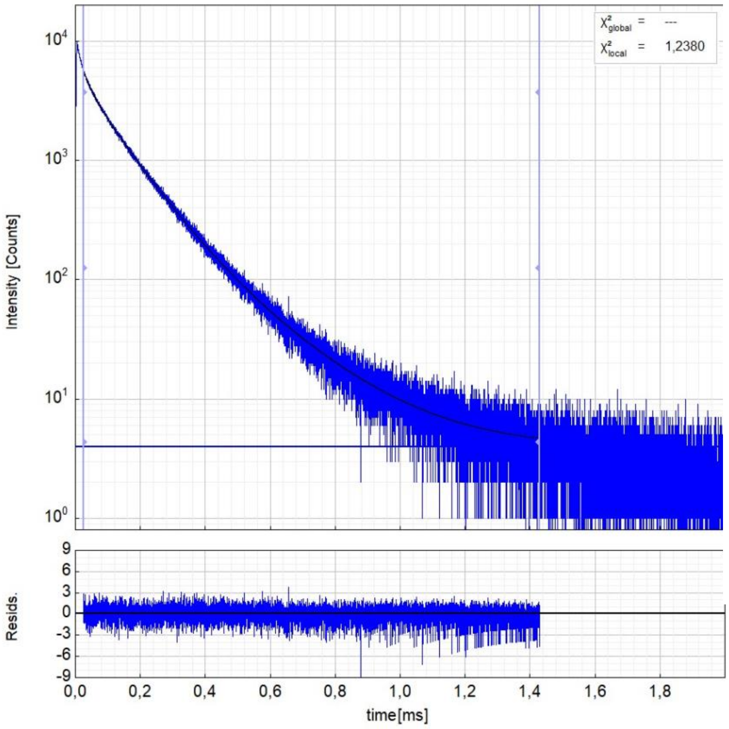

Parameter	Value	Δ	δ
A_1[kCnts/Chnl]	0,61	±0,18	30%
τ_1[ns]	209 000	±21 000	10%
I_1[kCnts]	990	±230	23%
A_{Rel1}[%]	10,7	±3,2	30%
I_{Rel1}[%]	23,8	±5,4	23%
A_2[kCnts/Chnl]	3,693	±0,090	2,4%
τ_2[ns]	100 900	±4 300	4,2%
I_2[kCnts]	2 920	±200	6,7%
A_{Rel2}[%]	65,3	±1,6	2,4%
I_{Rel2}[%]	70,3	±4,7	6,7%
A_3[kCnts/Chnl]	1,363	±0,092	6,7%
τ_3[ns]	23 600	±1 700	6,8%
I_3[kCnts]	251	±31	12%
A_{Rel3}[%]	24,1	±1,7	6,7%
I_{Rel3}[%]	6,1	±0,8	12%
$Bkgr_{Dec}$[kCnts]	0,0040	±0,0004	7,7%
τ_{AvInt}[ns]	121 840	±710	0,6%
τ_{AvAmp}[ns]	93 770	±650	0,7%

Figure S99: Left: Raw (experimental) time-resolved photoluminescence decay of [**PtLH$_2$Gly**] in a frozen glassy DCM/MeOH (V:V = 1:1) at 77 K, including the residuals (λ_{exc} = 376.7 nm, λ_{em} = 485 nm). Right: Fitting parameters including pre-exponential factors and confidence limits.

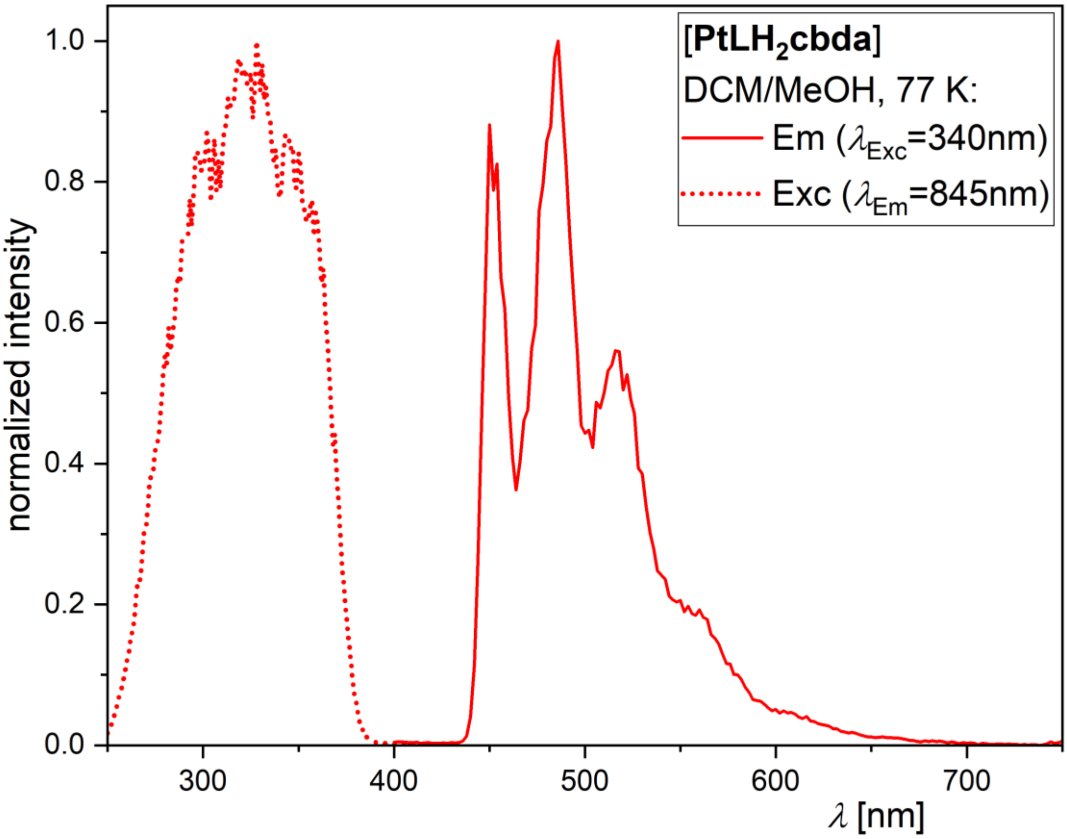

Figure S100: Excitation (dotted line) and emission spectra (solid line) of [**PtLH$_2$cbda**] at 77 K (red) in a frozen glassy DCM/MeOH matrix (V:V = 1:1). All solutions were optically diluted (A < 0.1). Spectra normalized to the highest intensity.

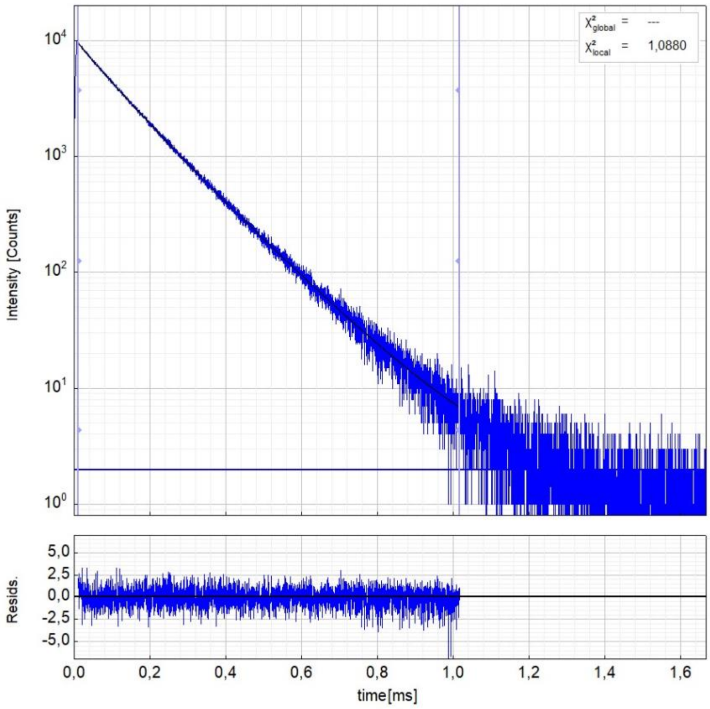

Parameter	Value	Δ	δ
A_1[kCnts/Chnl]	4,9	±1,4	28%
τ_1[ns]	145 200	±7 000	4,8%
I_1[kCnts]	2 770	±600	22%
A_{Rel1}[%]	53	±15	27%
I_{Rel1}[%]	64	±14	22%
A_2[kCnts/Chnl]	4,5	±1,4	30%
τ_2[ns]	92 500	±10 000	11%
I_2[kCnts]	1 620	±610	37%
A_{Rel2}[%]	48	±15	30%
I_{Rel2}[%]	37	±14	37%
$Bkgr_{Dec}$[kCnts]	0,0020	±0,0009	45%
τ_{AvInt}[ns]	125 750	±380	0,3%
τ_{AvAmp}[ns]	119 980	±390	0,3%

Figure S101: Left: Raw (experimental) time-resolved photoluminescence decay of [**PtLH$_2$cbda**] in a frozen glassy DCM/MeOH (V:V = 1:1) at 77 K, including the residuals (λ_{exc} = 376.7 nm, λ_{em} = 485 nm). Right: Fitting parameters including pre-exponential factors and confidence limits.

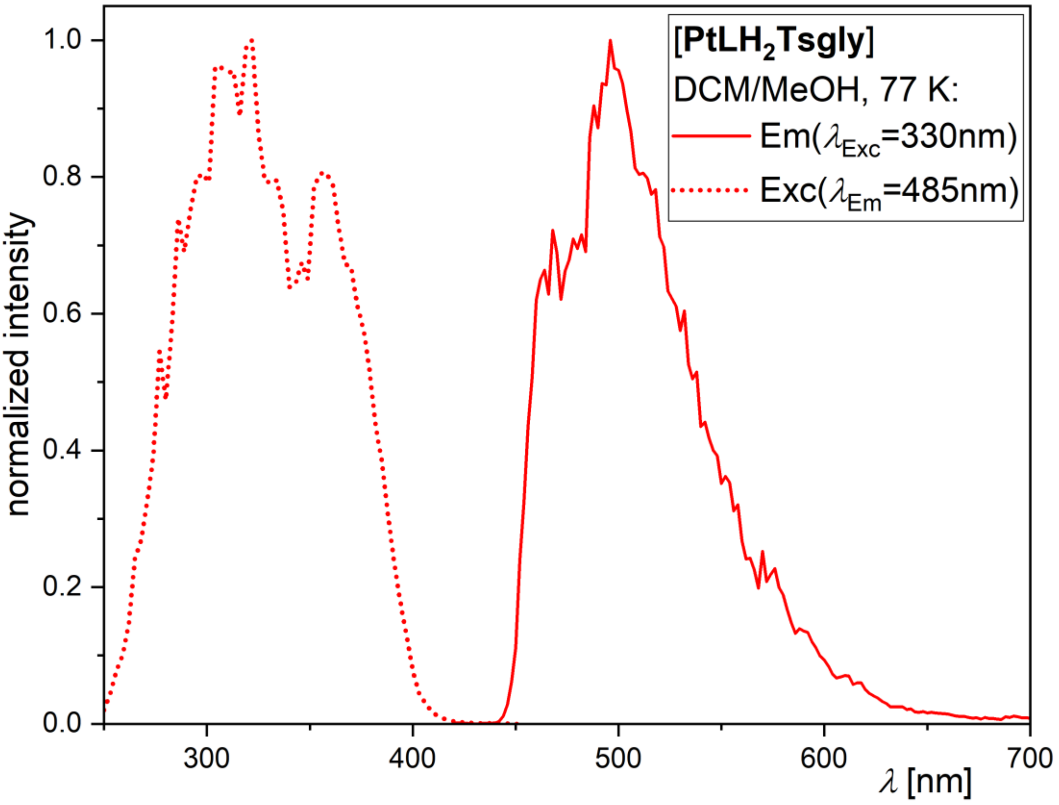

Figure S102: Excitation (dotted line) and emission spectra (solid line) of [**PtLH$_2$Tsgly**] at 77 K (red) in a frozen glassy DCM/MeOH matrix (V:V = 1:1). All solutions were optically diluted (A < 0.1). Spectra normalized to the highest intensity.

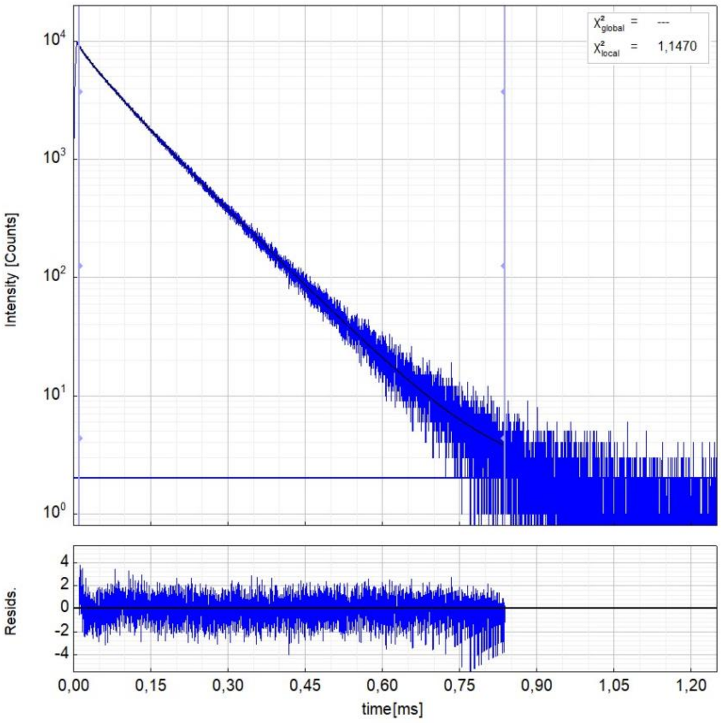

Parameter	Value	Δ	δ
A_1[kCnts/Chnl]	6,58	±0,14	2,0%
τ_1[ns]	100 840	±510	0,5%
I_1[kCnts]	5 182	±81	1,5%
A_{Rel1}[%]	73,8	±1,4	1,8%
I_{Rel1}[%]	86,2	±1,4	1,5%
A_2[kCnts/Chnl]	2,35	±0,12	5,0%
τ_2[ns]	45 600	±2 000	4,4%
I_2[kCnts]	835	±80	9,6%
A_{Rel2}[%]	26,3	±1,4	5,2%
I_{Rel2}[%]	13,9	±1,4	9,6%
$Bkgr_{Dec}$[kCnts]	0,0020	±0,0002	5,7%
τ_{AvInt}[ns]	93 163	±49	0,1%
τ_{AvAmp}[ns]	86 300	±170	0,2%

Figure S103: Left: Raw (experimental) time-resolved photoluminescence decay of [**PtLH2Tsgly**] in a frozen glassy DCM/MeOH (V:V = 1:1) at 77 K, including the residuals (λ_{exc} = 376.7 nm, λ_{em} = 485 nm). Right: Fitting parameters including pre-exponential factors and confidence limits.

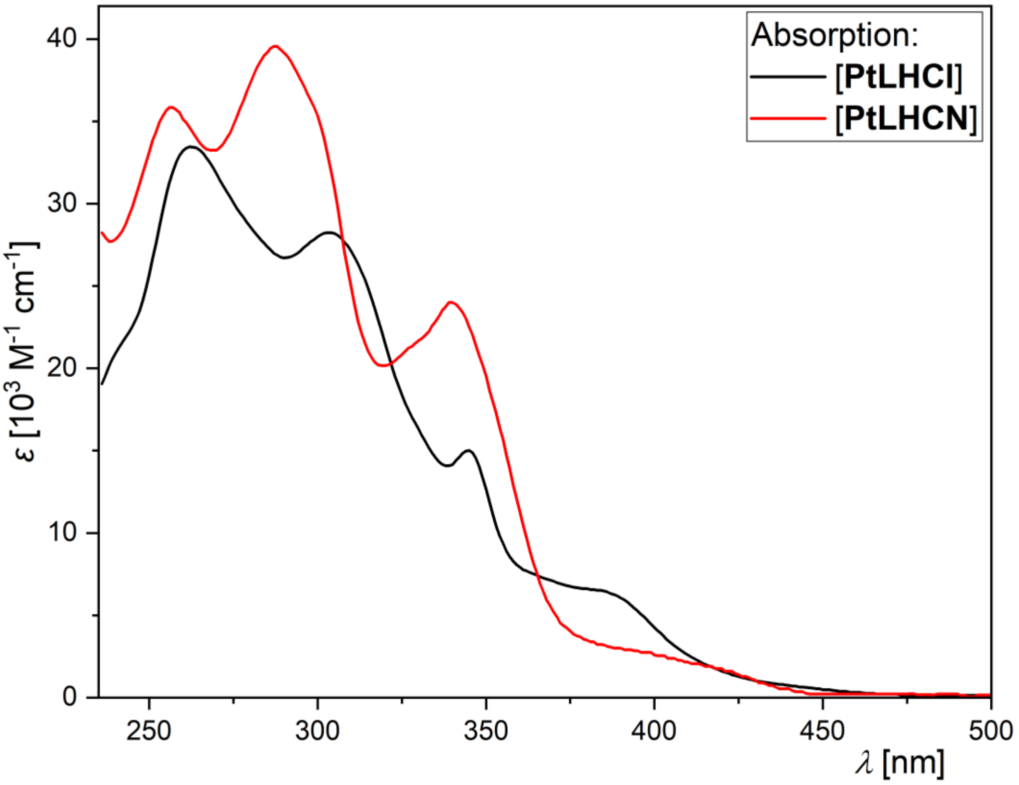

Figure S104: Molar absorption coefficients as a function of wavelength for [**PtLHCl**] (black) and [**PtLHCN**] (red) (validity range: c = 1x10^{-5} – 5x10^{-7} M in DCM at 298 K).

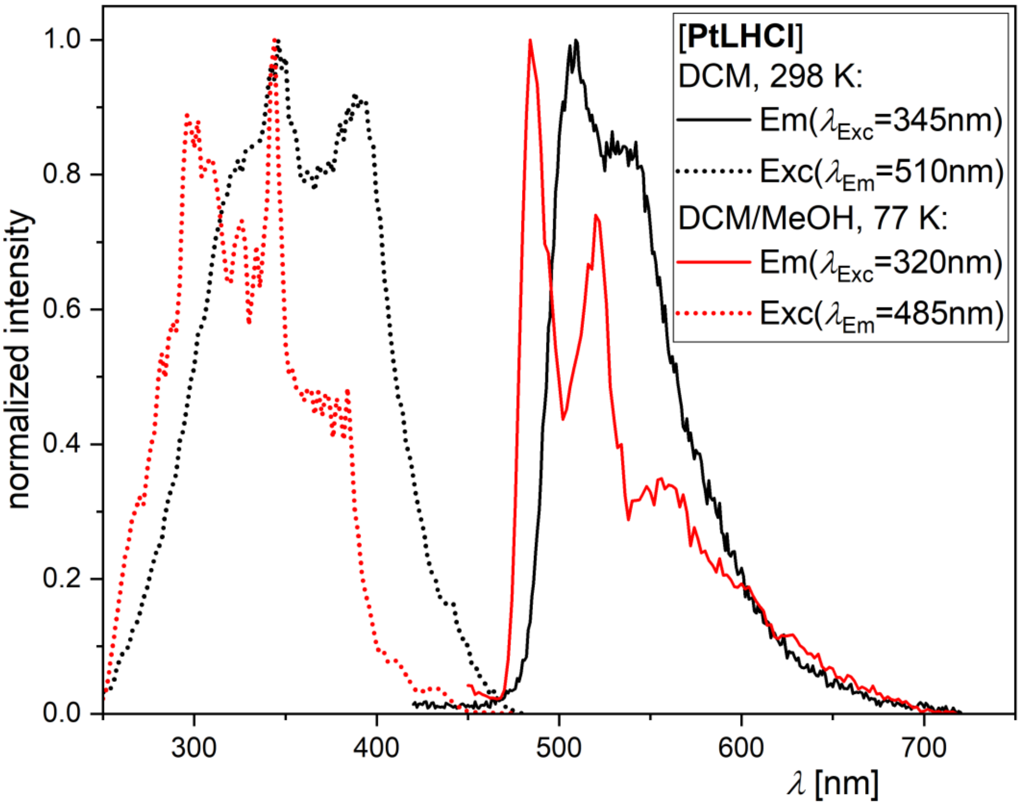

Figure S105: Excitation (dotted line) and emission spectra (solid line) of [**PtLHCl**] at 298 K (black) in liquid DCM and at 77 K (red) in a frozen glassy DCM/MeOH matrix (V:V = 1:1). All solutions were optically diluted (A < 0.1). Spectra normalized to the highest intensity.

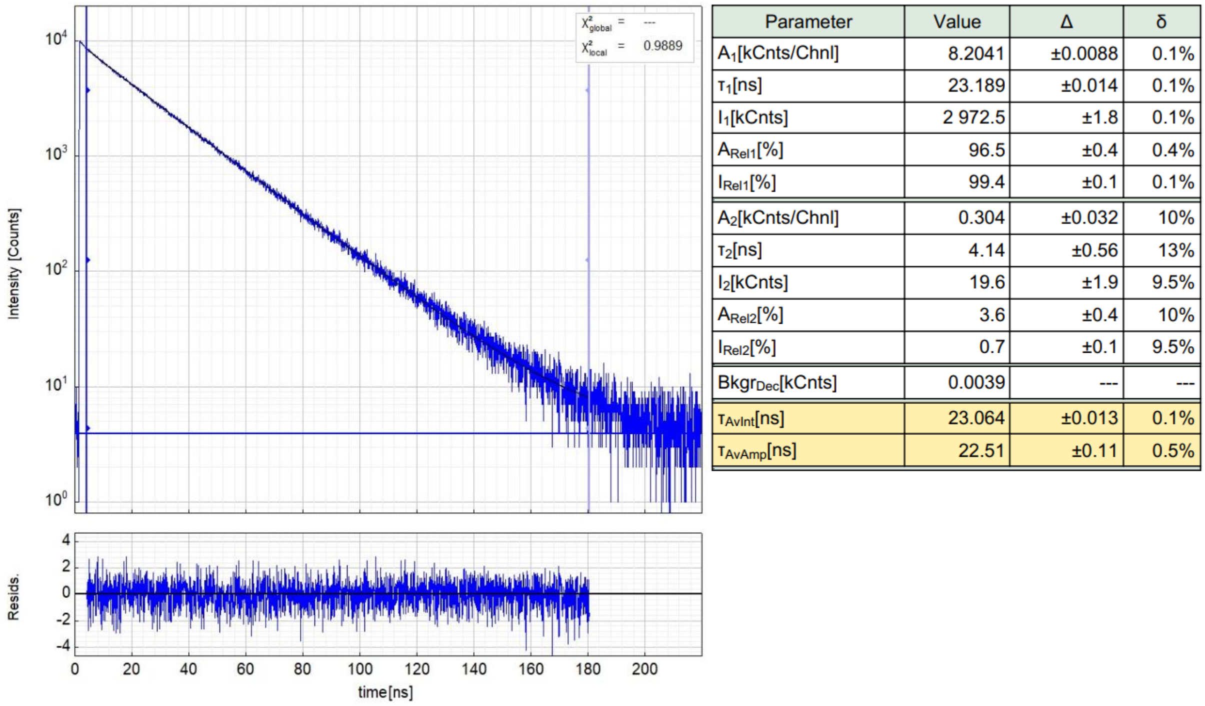

Parameter	Value	Δ	δ
A_1[kCnts/Chnl]	8.2041	±0.0088	0.1%
τ_1[ns]	23.189	±0.014	0.1%
I_1[kCnts]	2 972.5	±1.8	0.1%
A_{Rel1}[%]	96.5	±0.4	0.4%
I_{Rel1}[%]	99.4	±0.1	0.1%
A_2[kCnts/Chnl]	0.304	±0.032	10%
τ_2[ns]	4.14	±0.56	13%
I_2[kCnts]	19.6	±1.9	9.5%
A_{Rel2}[%]	3.6	±0.4	10%
I_{Rel2}[%]	0.7	±0.1	9.5%
$Bkgr_{Dec}$[kCnts]	0.0039	---	---
τ_{AvInt}[ns]	23.064	±0.013	0.1%
τ_{AvAmp}[ns]	22.51	±0.11	0.5%

Figure S106: Left: Raw (experimental) time-resolved photoluminescence decay of [**PtLHCl**] in liquid DCM (air-equilibrated) at 298 K, including the residuals (λ_{exc} = 376.7 nm, λ_{em} = 510 nm). Right: Fitting parameters including pre-exponential factors and confidence limits.

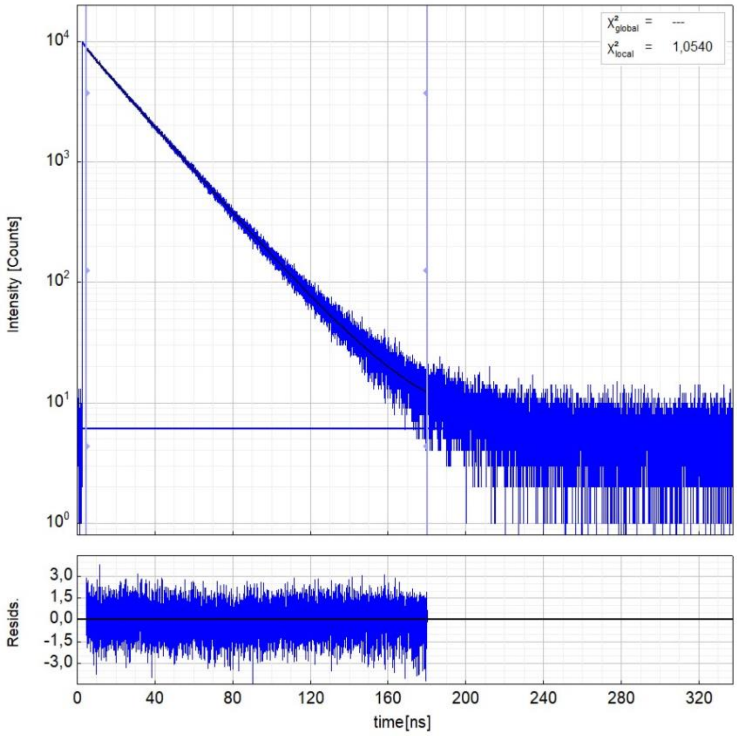

Parameter	Value	Δ	δ
A_1[kCnts/Chnl]	8,286	±0,070	0,8%
τ_1[ns]	24,301	±0,062	0,3%
I_1[kCnts]	12 583	±76	0,6%
A_{Rel1}[%]	94,5	±0,8	0,8%
I_{Rel1}[%]	97,8	±0,6	0,6%
A_2[kCnts/Chnl]	0,492	±0,062	13%
τ_2[ns]	9,4	±1,7	17%
I_2[kCnts]	286	±76	26%
A_{Rel2}[%]	5,6	±0,8	13%
I_{Rel2}[%]	2,3	±0,6	26%
$Bkgr_{Dec}$[kCnts]	0,0061	---	---
τ_{AvInt}[ns]	23,968	±0,015	0,1%
τ_{AvAmp}[ns]	23,462	±0,047	0,2%

Figure S107: Left: Raw (experimental) time-resolved photoluminescence decay of [**PtLHCl**] in liquid DCM (Ar-purged) at 298 K, including the residuals (λ_{exc} = 376.7 nm, λ_{em} = 510 nm). Right: Fitting parameters including pre-exponential factors and confidence limits.

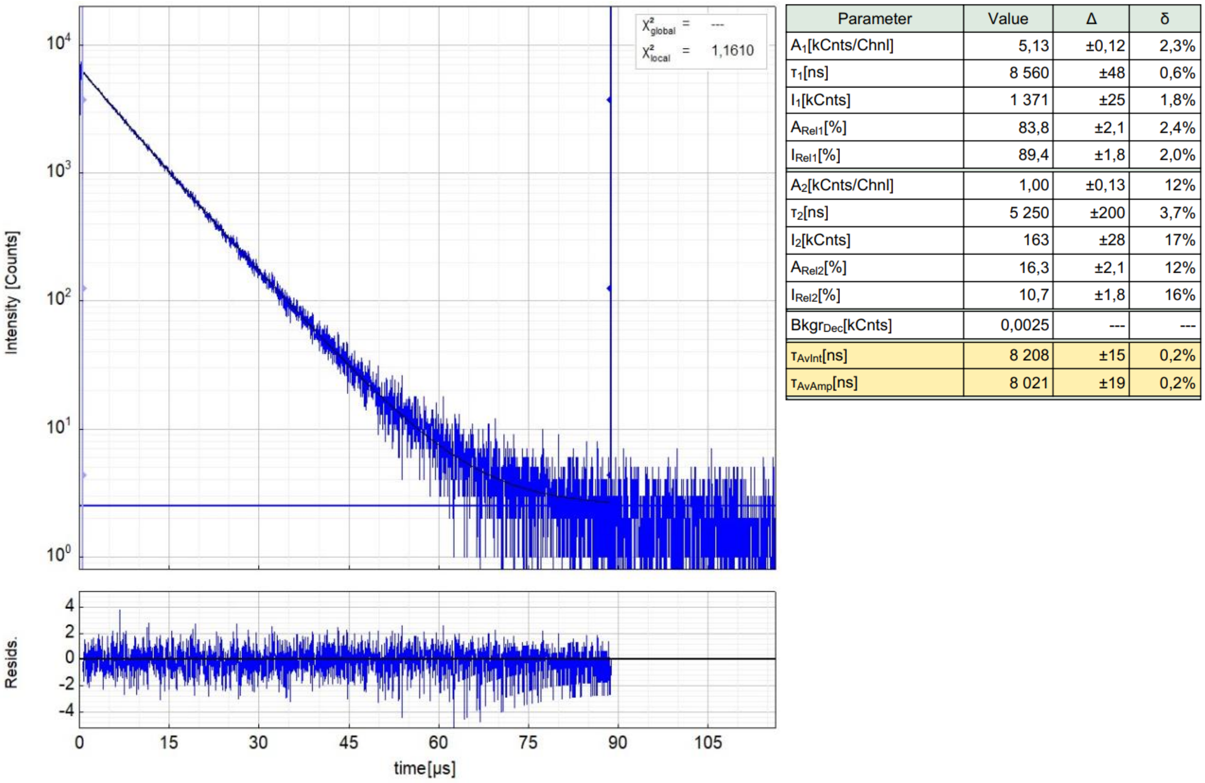

Parameter	Value	Δ	δ
A_1[kCnts/Chnl]	5,13	±0,12	2,3%
τ_1[ns]	8 560	±48	0,6%
I_1[kCnts]	1 371	±25	1,8%
A_{Rel1}[%]	83,8	±2,1	2,4%
I_{Rel1}[%]	89,4	±1,8	2,0%
A_2[kCnts/Chnl]	1,00	±0,13	12%
τ_2[ns]	5 250	±200	3,7%
I_2[kCnts]	163	±28	17%
A_{Rel2}[%]	16,3	±2,1	12%
I_{Rel2}[%]	10,7	±1,8	16%
$Bkgr_{Dec}$[kCnts]	0,0025	---	---
τ_{AvInt}[ns]	8 208	±15	0,2%
τ_{AvAmp}[ns]	8 021	±19	0,2%

Figure S108: Left: Raw (experimental) time-resolved photoluminescence decay of [**PtLHCl**] in a frozen glassy DCM/MeOH (V:V = 1:1) at 77 K, including the residuals (λ_{exc} = 376.7 nm, λ_{em} = 485 nm). Right: Fitting parameters including pre-exponential factors and confidence limits.

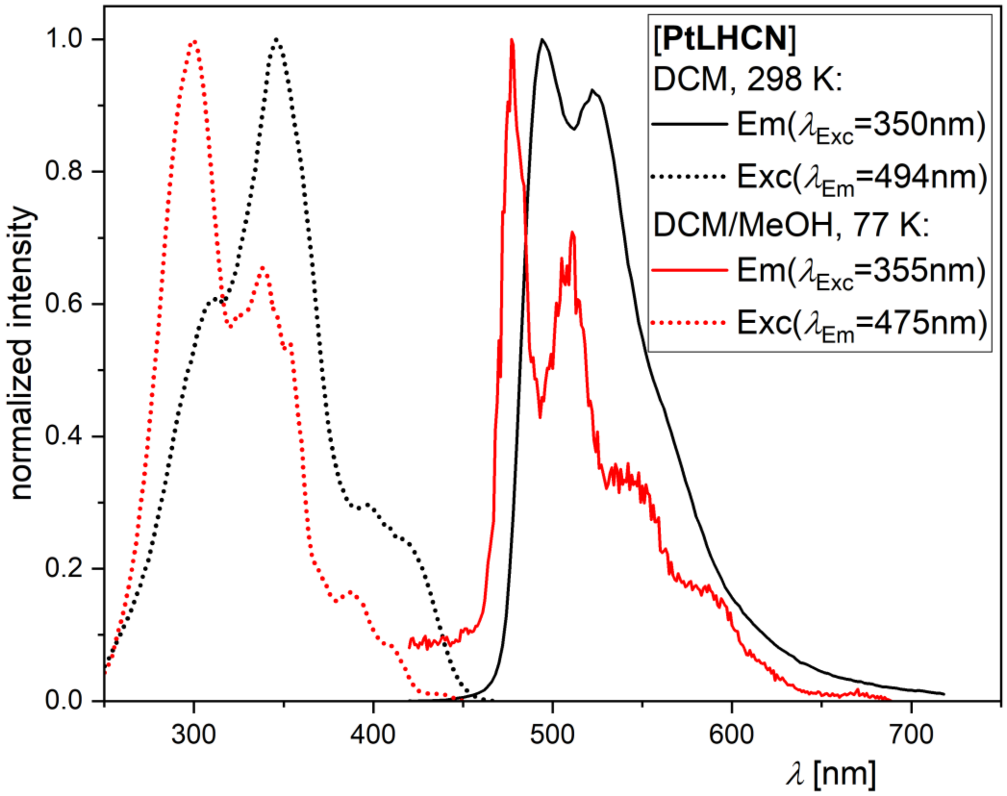

Figure S109: Excitation (dotted line) and emission spectra (solid line) of [**PtLHCN**] at 298 K (black) in liquid DCM and at 77 K (red) in a frozen glassy DCM/MeOH matrix (V:V = 1:1). All solutions were optically diluted (A < 0.1). Spectra normalized to the highest intensity.

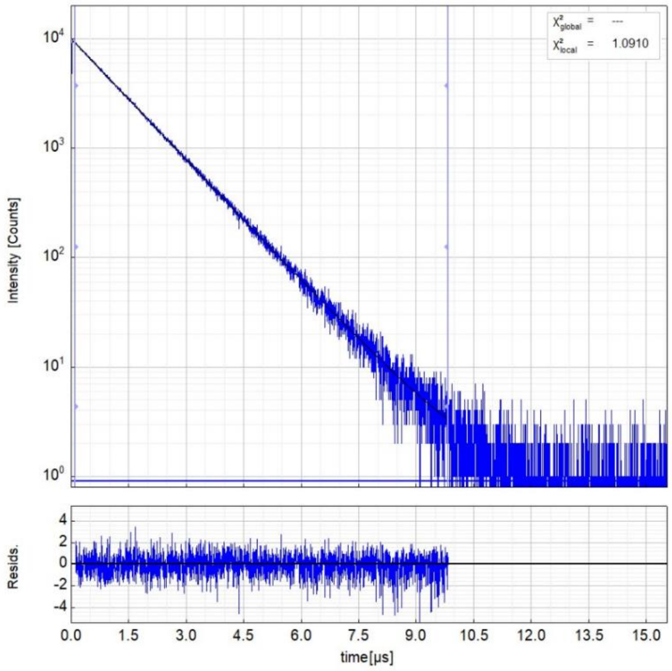

Parameter	Value	Δ	δ
A_1[kCnts/Chnl]	9.090	±0.013	0.1%
τ_1[ns]	1 180.6	±1.1	0.1%
I_1[kCnts]	2 682.8	±2.7	0.1%
A_{Rel1}[%]	100.0	---	---
I_{Rel1}[%]	100.0	---	---
$Bkgr_{Dec}$[kCnts]	0.0010	±0.0002	14%
τ_{AvInt}[ns]	1 180.6	±1.1	0.1%
τ_{AvAmp}[ns]	1 180.6	±1.1	0.1%

Figure S110: Left: Raw (experimental) time-resolved photoluminescence decay of [**PtLHCN**] in liquid DCM (air-equilibrated) at 298 K, including the residuals (λ_{exc} = 376.7 nm, λ_{em} = 494 nm). Right: Fitting parameters including pre-exponential factors and confidence limits.

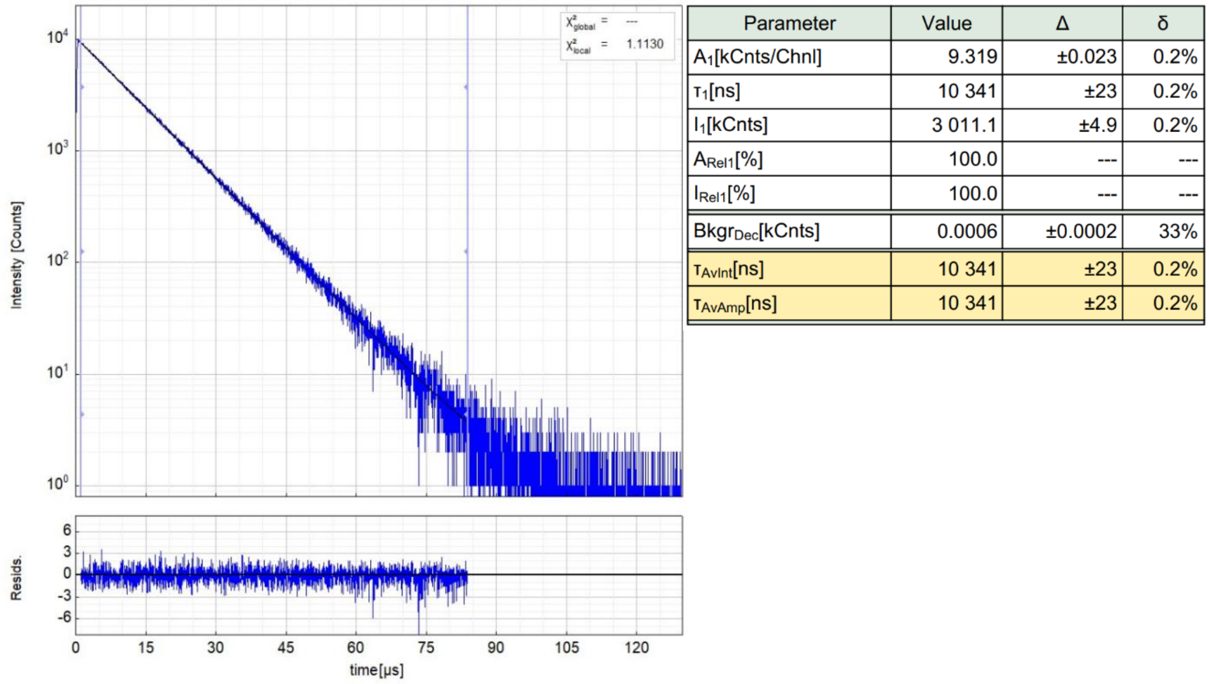

Parameter	Value	Δ	δ
A_1[kCnts/Chnl]	9.319	±0.023	0.2%
τ_1[ns]	10 341	±23	0.2%
I_1[kCnts]	3 011.1	±4.9	0.2%
A_{Rel1}[%]	100.0	---	---
I_{Rel1}[%]	100.0	---	---
$Bkgr_{Dec}$[kCnts]	0.0006	±0.0002	33%
τ_{AvInt}[ns]	10 341	±23	0.2%
τ_{AvAmp}[ns]	10 341	±23	0.2%

Figure S111: Left: Raw (experimental) time-resolved photoluminescence decay of [**PtLHCN**] in liquid DCM (Ar-purged) at 298 K, including the residuals (λ_{exc} = 376.7 nm, λ_{em} = 494 nm). Right: Fitting parameters including pre-exponential factors and confidence limits.

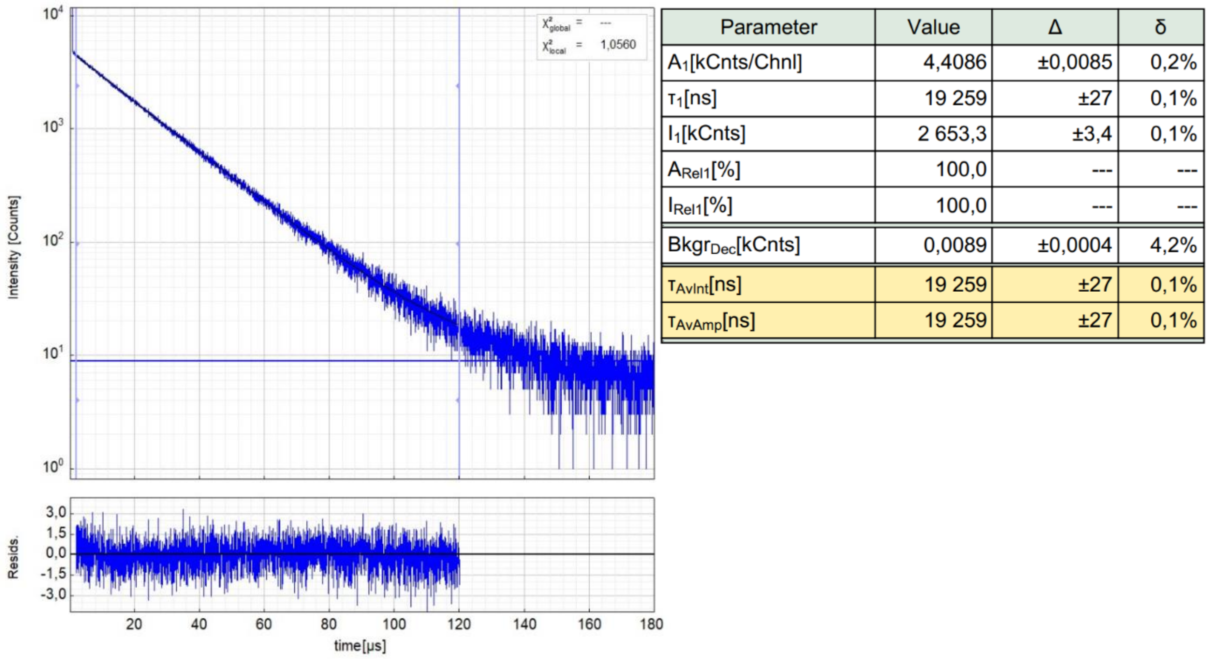

Parameter	Value	Δ	δ
A_1[kCnts/Chnl]	4,4086	±0,0085	0,2%
τ_1[ns]	19 259	±27	0,1%
I_1[kCnts]	2 653,3	±3,4	0,1%
A_{Rel1}[%]	100,0	---	---
I_{Rel1}[%]	100,0	---	---
$Bkgr_{Dec}$[kCnts]	0,0089	±0,0004	4,2%
τ_{AvInt}[ns]	19 259	±27	0,1%
τ_{AvAmp}[ns]	19 259	±27	0,1%

Figure S112: Left: Raw (experimental) time-resolved photoluminescence decay of [**PtLHCN**] in a frozen glassy DCM/MeOH (V:V = 1:1) at 77 K, including the residuals (λ_{exc} = 376.7 nm, λ_{em} = 485 nm). Right: Fitting parameters including pre-exponential factors and confidence limits.

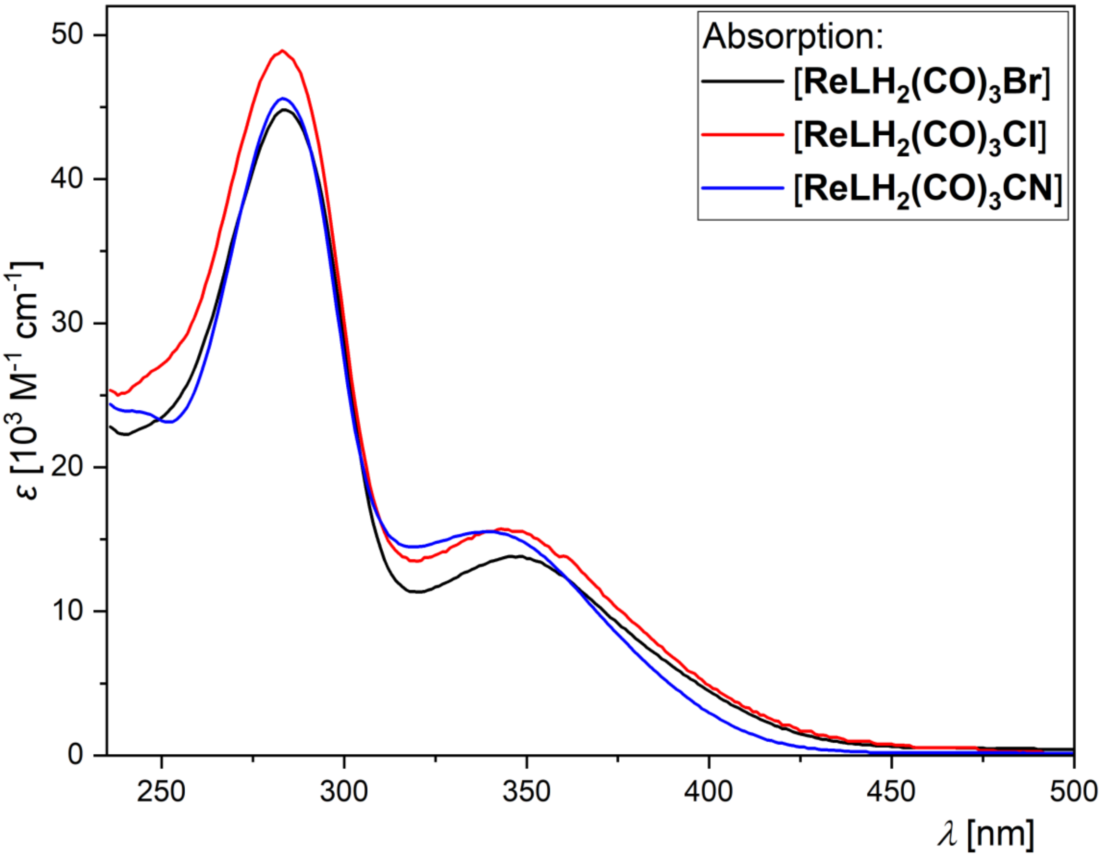

Figure S113: Molar absorption coefficients as a function of wavelength for [**$ReLH_2(CO)_3Br$**] (black), [**$ReLH_2(CO)_3Cl$**] (red) and [**$ReLH_2(CO)_3CN$**] (blue) (validity range: c = 1x10^{-5} – 5x10^{-7} M in DCM at 298 K).

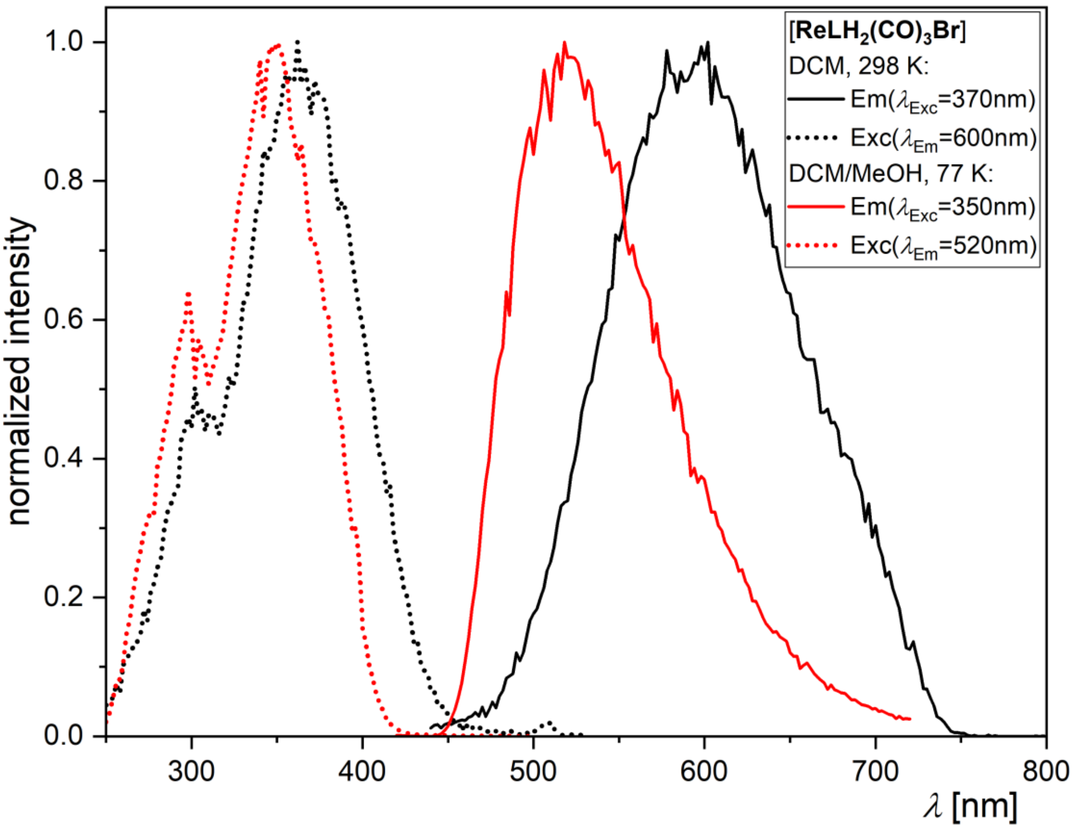

Figure S114: Excitation (dotted line) and emission spectra (solid line) of [**$ReLH_2(CO)_3Br$**] at 298 K (black) in liquid DCM and at 77 K (red) in a frozen glassy DCM/MeOH matrix (V:V = 1:1). All solutions were optically diluted (A < 0.1). Spectra normalized to the highest intensity.

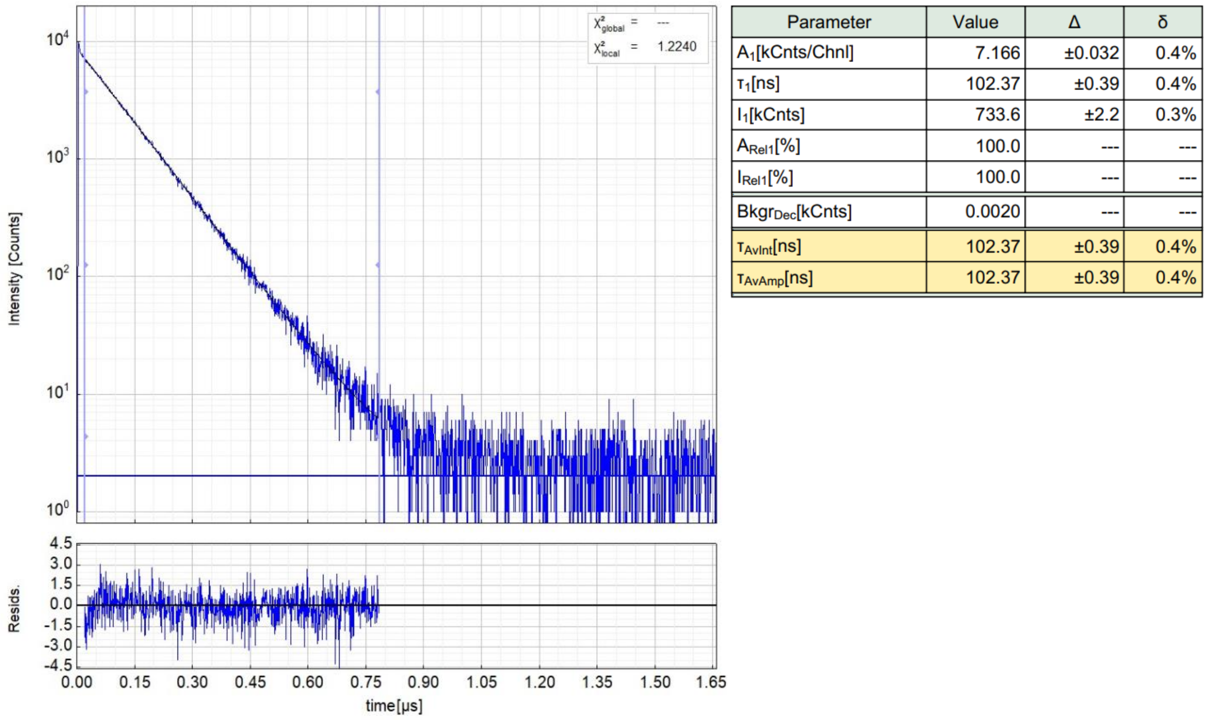

Parameter	Value	Δ	δ
A_1[kCnts/Chnl]	7.166	±0.032	0.4%
$τ_1$[ns]	102.37	±0.39	0.4%
I_1[kCnts]	733.6	±2.2	0.3%
A_{Rel1}[%]	100.0	---	---
I_{Rel1}[%]	100.0	---	---
$Bkgr_{Dec}$[kCnts]	0.0020	---	---
$τ_{AvInt}$[ns]	102.37	±0.39	0.4%
$τ_{AvAmp}$[ns]	102.37	±0.39	0.4%

Figure S115: Left: Raw (experimental) time-resolved photoluminescence decay of [**$ReLH_2(CO)_3Br$**] in liquid DCM (air-equilibrated) at 298 K, including the residuals ($λ_{exc}$ = 376.7 nm, $λ_{em}$ = 600 nm). Right: Fitting parameters including pre-exponential factors and confidence limits.

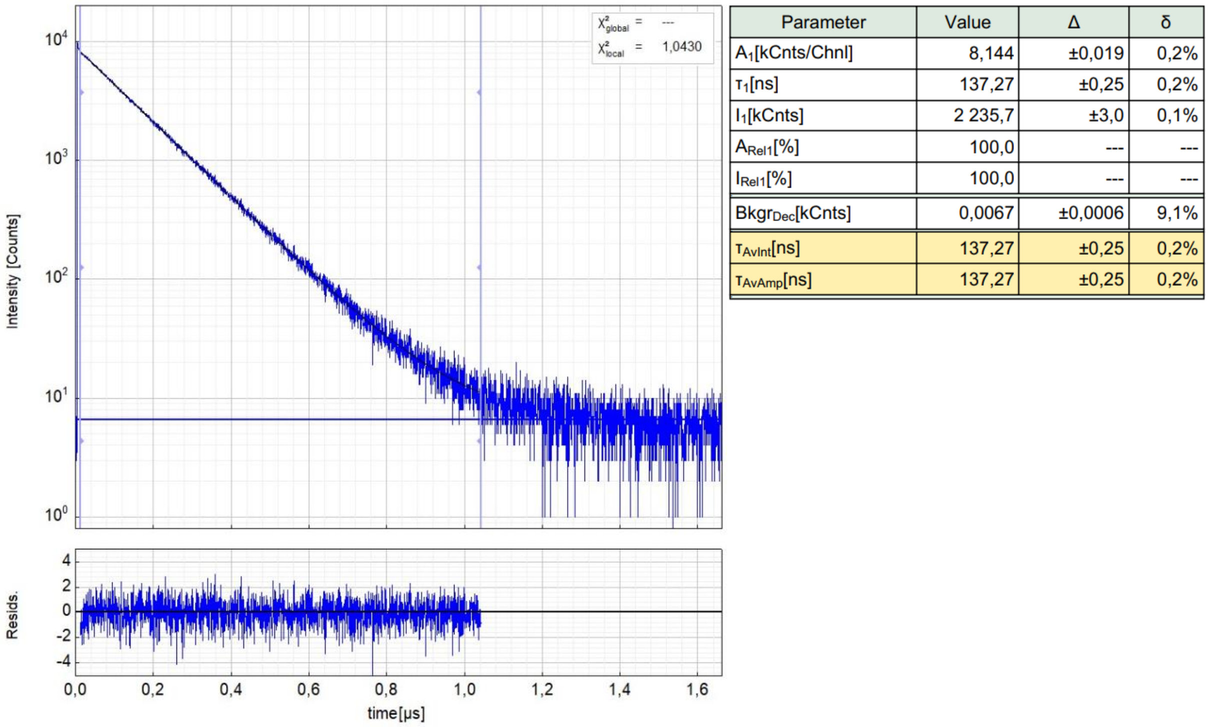

Parameter	Value	Δ	δ
A_1[kCnts/Chnl]	8,144	±0,019	0,2%
τ_1[ns]	137,27	±0,25	0,2%
I_1[kCnts]	2 235,7	±3,0	0,1%
A_{Rel1}[%]	100,0	---	---
I_{Rel1}[%]	100,0	---	---
$Bkgr_{Dec}$[kCnts]	0,0067	±0,0006	9,1%
τ_{AvInt}[ns]	137,27	±0,25	0,2%
τ_{AvAmp}[ns]	137,27	±0,25	0,2%

Figure S116: Left: Raw (experimental) time-resolved photoluminescence decay of [**$ReLH_2(CO)_3Br$**] in liquid DCM (Ar-purged) at 298 K, including the residuals (λ_{exc} = 376.7 nm, λ_{em} = 600 nm). Right: Fitting parameters including pre-exponential factors and confidence limits.

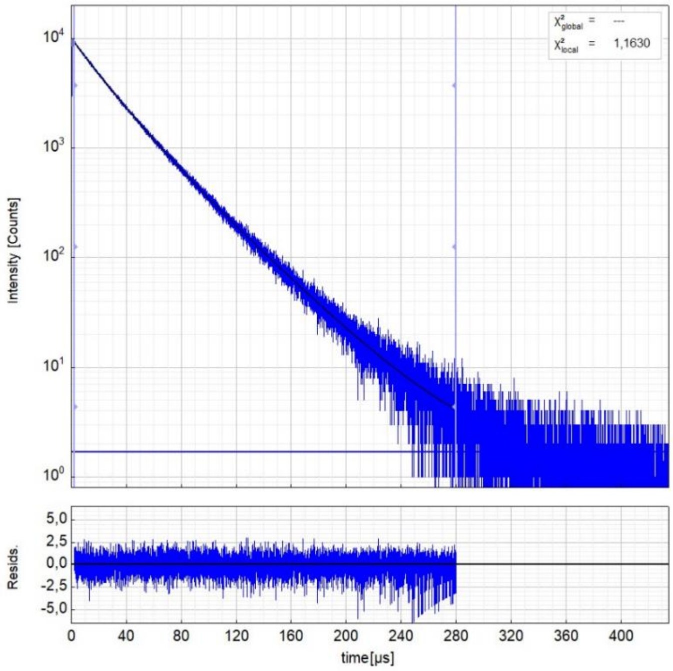

Parameter	Value	Δ	δ
A_1[kCnts/Chnl]	4,06	±0,22	5,2%
τ_1[ns]	37 470	±460	1,2%
I_1[kCnts]	4 760	±200	4,0%
A_{Rel1}[%]	44,4	±2,3	5,0%
I_{Rel1}[%]	58,7	±2,4	4,0%
A_2[kCnts/Chnl]	5,11	±0,20	3,8%
τ_2[ns]	20 990	±400	1,9%
I_2[kCnts]	3 350	±200	5,7%
A_{Rel2}[%]	55,7	±2,3	4,0%
I_{Rel2}[%]	41,4	±2,4	5,7%
$Bkgr_{Dec}$[kCnts]	0,0017	±0,0002	9,4%
τ_{AvInt}[ns]	30 652	±41	0,1%
τ_{AvAmp}[ns]	28 283	±57	0,2%

Figure S117: Left: Raw (experimental) time-resolved photoluminescence decay of [**$ReLH_2(CO)_3Br$**] in a frozen glassy DCM/MeOH (V:V = 1:1) at 77 K, including the residuals (λ_{exc} = 376.7 nm, λ_{em} = 520 nm). Right: Fitting parameters including pre-exponential factors and confidence limits.

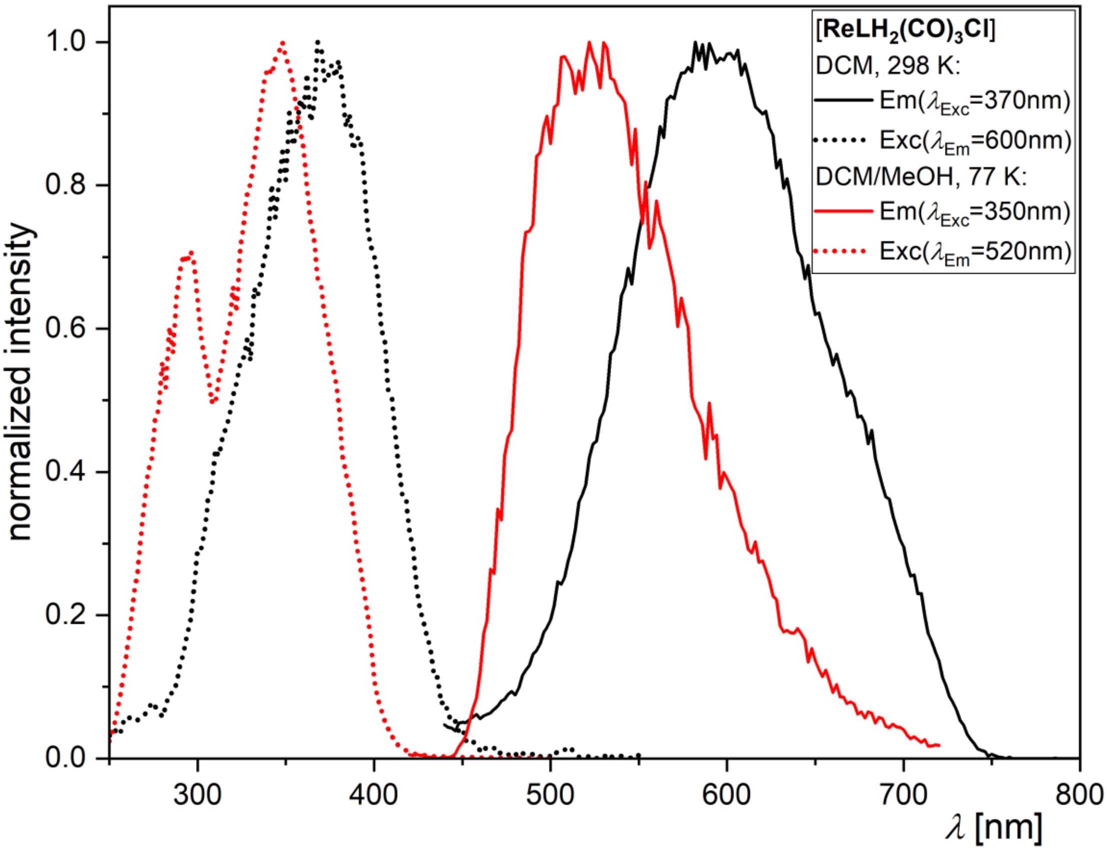

Figure S118: Excitation (dotted line) and emission spectra (solid line) of [**ReLH$_2$(CO)$_3$Cl**] at 298 K (black) in liquid DCM and at 77 K (red) in a frozen glassy DCM/MeOH matrix (V:V = 1:1). All solutions were optically diluted (A < 0.1). Spectra normalized to the highest intensity.

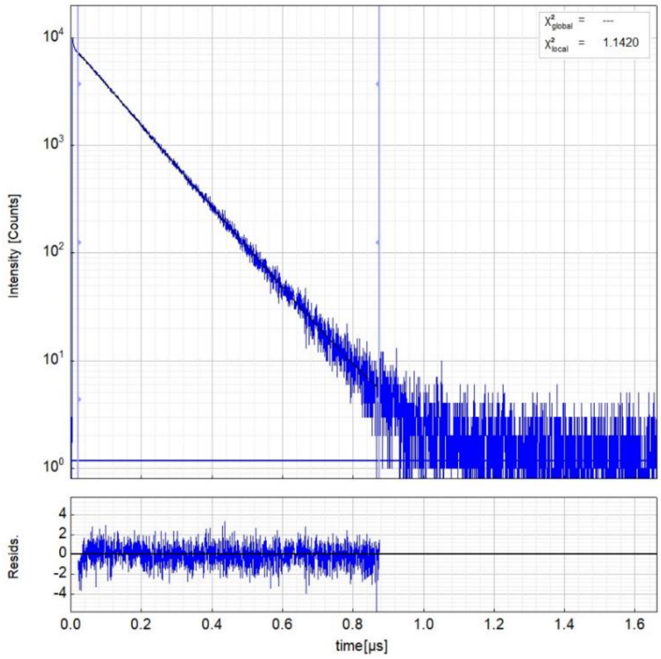

Parameter	Value	Δ	δ
A_1[kCnts/Chnl]	7.366	±0.026	0.4%
τ_1[ns]	114.64	±0.17	0.1%
I_1[kCnts]	1 688.6	±3.6	0.2%
A_{Rel1}[%]	100.0	---	---
I_{Rel1}[%]	100.0	---	---
$Bkgr_{Dec}$[kCnts]	0.0012	±0.0005	36%
τ_{AvInt}[ns]	114.64	±0.17	0.1%
τ_{AvAmp}[ns]	114.64	±0.17	0.1%

Figure S119: Left: Raw (experimental) time-resolved photoluminescence decay of [**ReLH$_2$(CO)$_3$Cl**] in liquid DCM (air-equilibrated) at 298 K, including the residuals (λ_{exc} = 376.7 nm, λ_{em} = 600 nm). Right: Fitting parameters including pre-exponential factors and confidence limits.

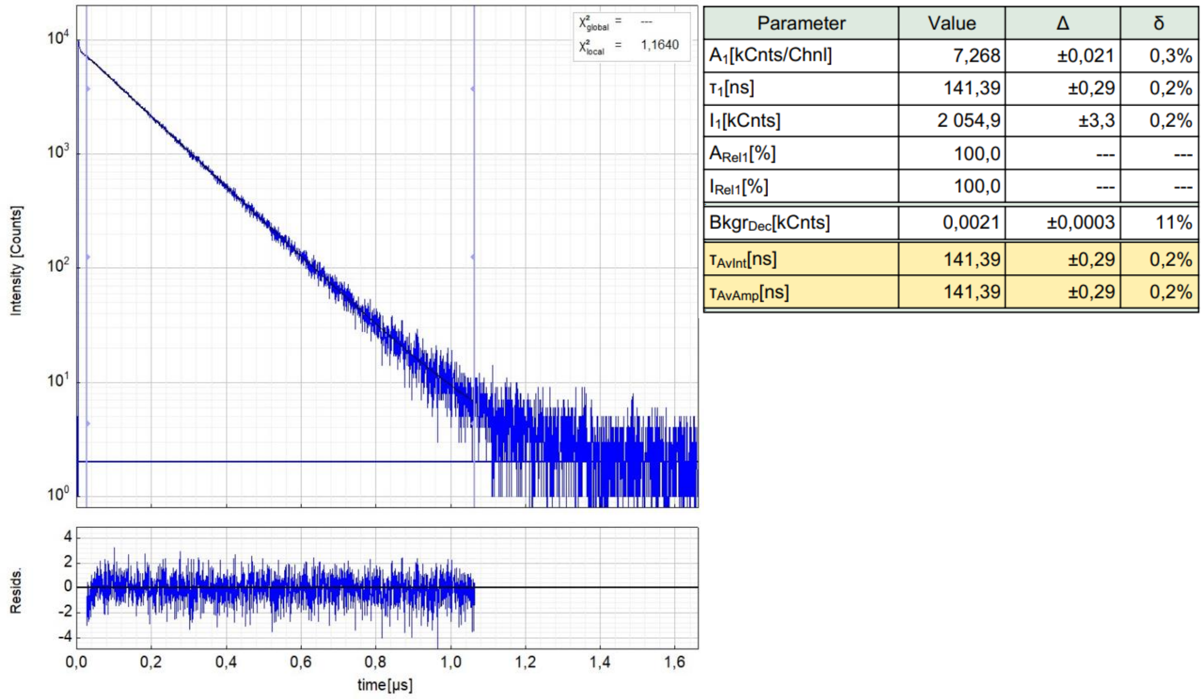

Parameter	Value	Δ	δ
A_1[kCnts/Chnl]	7,268	±0,021	0,3%
τ_1[ns]	141,39	±0,29	0,2%
I_1[kCnts]	2 054,9	±3,3	0,2%
A_{Rel1}[%]	100,0	---	---
I_{Rel1}[%]	100,0	---	---
$Bkgr_{Dec}$[kCnts]	0,0021	±0,0003	11%
τ_{AvInt}[ns]	141,39	±0,29	0,2%
τ_{AvAmp}[ns]	141,39	±0,29	0,2%

Figure S120: Left: Raw (experimental) time-resolved photoluminescence decay of [**ReLH$_2$(CO)$_3$Cl**] in liquid DCM (Ar-purged) at 298 K, including the residuals (λ_{exc} = 376.7 nm, λ_{em} = 600 nm). Right: Fitting parameters including pre-exponential factors and confidence limits.

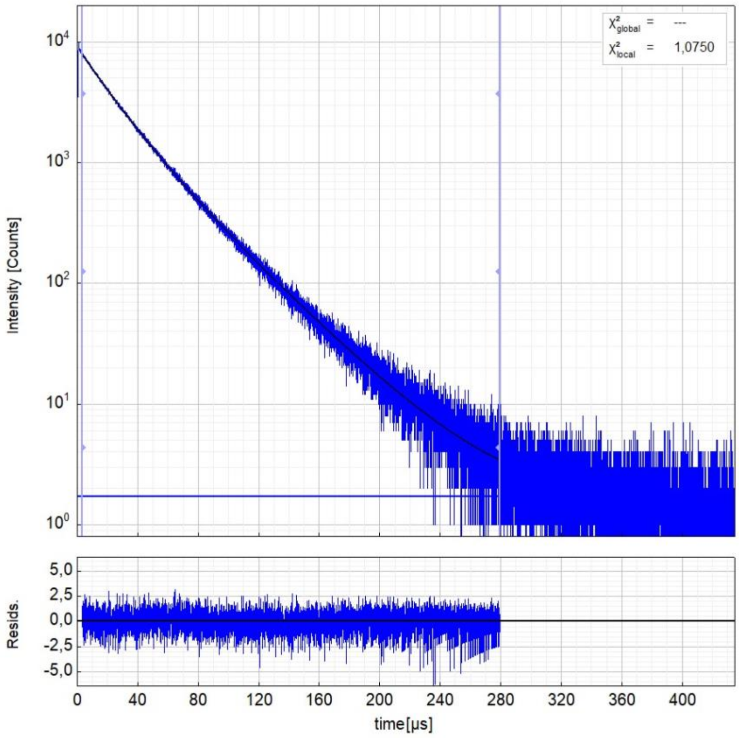

Parameter	Value	Δ	δ
A_1[kCnts/Chnl]	3,108	±0,051	1,6%
τ_1[ns]	36 850	±120	0,3%
I_1[kCnts]	3 579	±47	1,3%
A_{Rel1}[%]	39,5	±0,7	1,6%
I_{Rel1}[%]	55,3	±0,8	1,3%
A_2[kCnts/Chnl]	4,766	±0,049	1,0%
τ_2[ns]	19 470	±130	0,6%
I_2[kCnts]	2 899	±45	1,5%
A_{Rel2}[%]	60,6	±0,7	1,0%
I_{Rel2}[%]	44,8	±0,8	1,6%
$Bkgr_{Dec}$[kCnts]	0,0017	---	---
τ_{AvInt}[ns]	29 067	±13	0,0%
τ_{AvAmp}[ns]	26 326	±25	0,1%

Figure S121: Left: Raw (experimental) time-resolved photoluminescence decay of [**ReLH$_2$(CO)$_3$Cl**] in a frozen glassy DCM/MeOH (V:V = 1:1) at 77 K, including the residuals (λ_{exc} = 376.7 nm, λ_{em} = 520 nm). Right: Fitting parameters including pre-exponential factors and confidence limits.

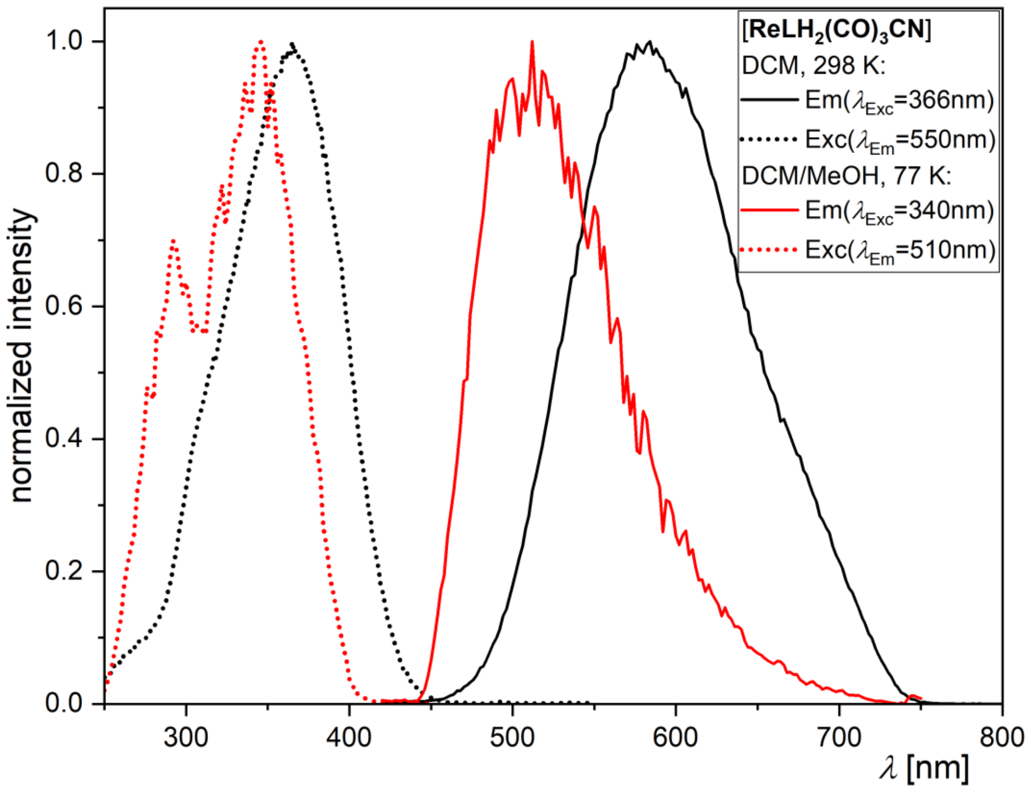

Figure S122: Excitation (dotted line) and emission spectra (solid line) of [**$ReLH_2(CO)_3CN$**] at 298 K (black) in liquid DCM and at 77 K (red) in a frozen glassy DCM/MeOH matrix (V:V = 1:1). All solutions were optically diluted (A < 0.1). Spectra normalized to the highest intensity.

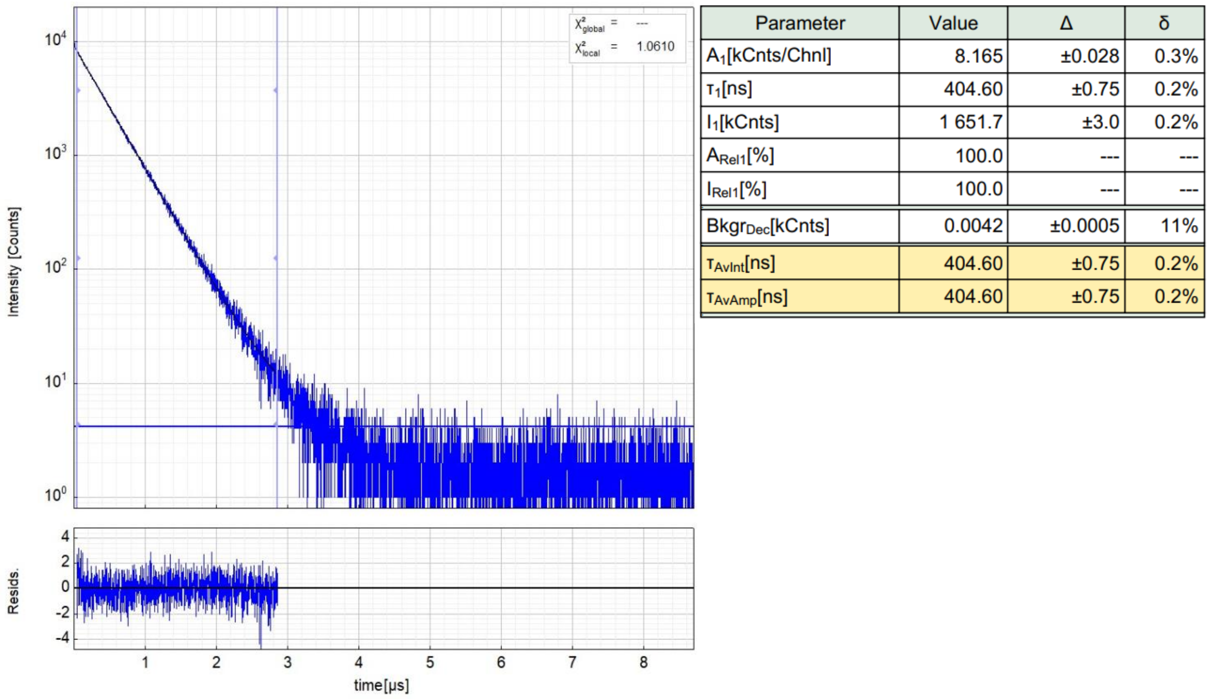

Parameter	Value	Δ	δ
A_1[kCnts/Chnl]	8.165	±0.028	0.3%
$τ_1$[ns]	404.60	±0.75	0.2%
I_1[kCnts]	1 651.7	±3.0	0.2%
A_{Rel1}[%]	100.0	---	---
I_{Rel1}[%]	100.0	---	---
$Bkgr_{Dec}$[kCnts]	0.0042	±0.0005	11%
$τ_{AvInt}$[ns]	404.60	±0.75	0.2%
$τ_{AvAmp}$[ns]	404.60	±0.75	0.2%

Figure S123: Left: Raw (experimental) time-resolved photoluminescence decay of [**$ReLH_2(CO)_3CN$**] in DCM (air-equilibrated) at 298 K, including the residuals ($λ_{exc}$ = 376.7 nm, $λ_{em}$ = 550 nm). Right: Fitting parameters including pre-exponential factors and confidence limits.

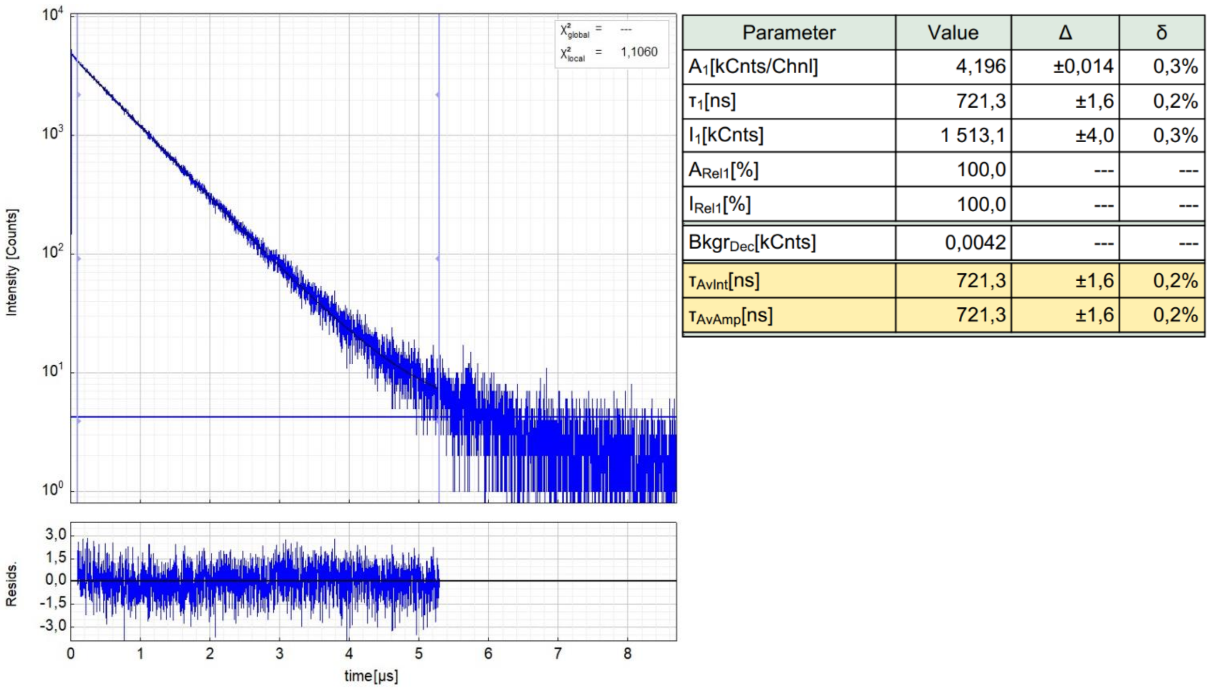

Parameter	Value	Δ	δ
A_1[kCnts/Chnl]	4,196	±0,014	0,3%
τ_1[ns]	721,3	±1,6	0,2%
I_1[kCnts]	1 513,1	±4,0	0,3%
A_{Rel1}[%]	100,0	---	---
I_{Rel1}[%]	100,0	---	---
$Bkgr_{Dec}$[kCnts]	0,0042	---	---
τ_{AvInt}[ns]	721,3	±1,6	0,2%
τ_{AvAmp}[ns]	721,3	±1,6	0,2%

Figure S124: Left: Raw (experimental) time-resolved photoluminescence decay of [**ReLH$_2$(CO)$_3$CN**] in liquid DCM (Ar-purged) at 298 K, including the residuals (λ_{exc} = 376.7 nm, λ_{em} = 550 nm). Right: Fitting parameters including pre-exponential factors and confidence limits.

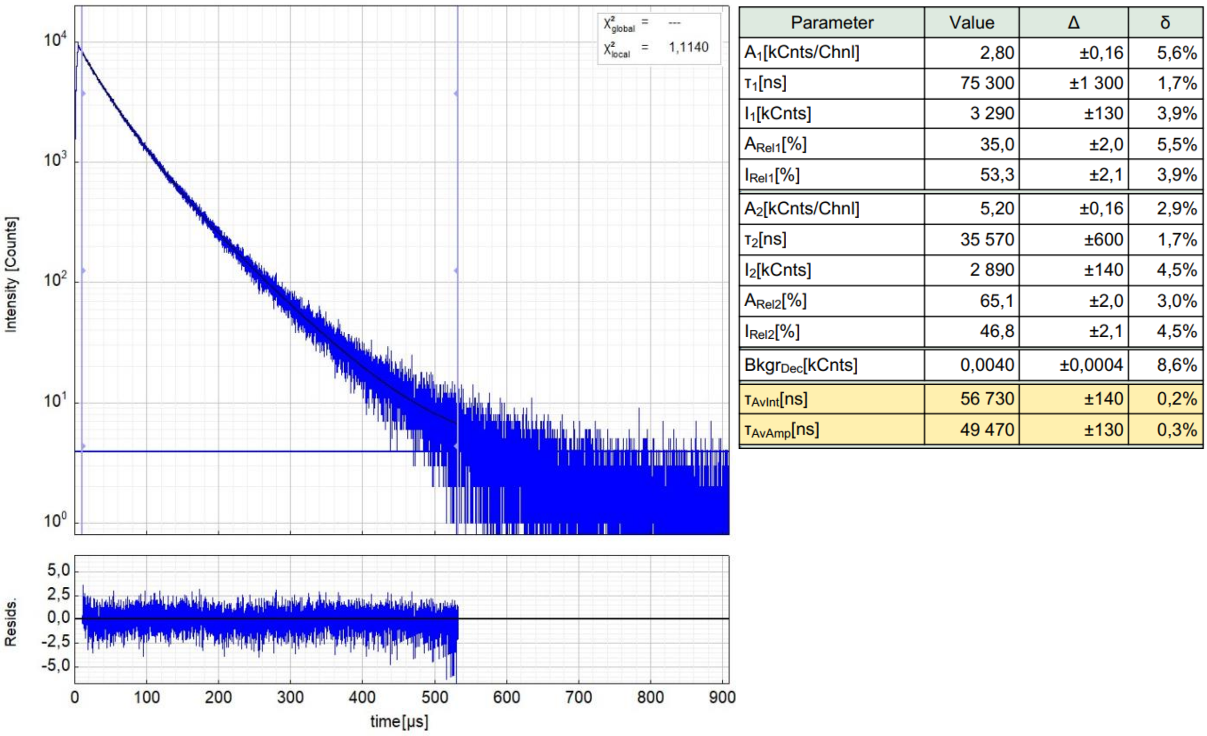

Parameter	Value	Δ	δ
A_1[kCnts/Chnl]	2,80	±0,16	5,6%
τ_1[ns]	75 300	±1 300	1,7%
I_1[kCnts]	3 290	±130	3,9%
A_{Rel1}[%]	35,0	±2,0	5,5%
I_{Rel1}[%]	53,3	±2,1	3,9%
A_2[kCnts/Chnl]	5,20	±0,16	2,9%
τ_2[ns]	35 570	±600	1,7%
I_2[kCnts]	2 890	±140	4,5%
A_{Rel2}[%]	65,1	±2,0	3,0%
I_{Rel2}[%]	46,8	±2,1	4,5%
$Bkgr_{Dec}$[kCnts]	0,0040	±0,0004	8,6%
τ_{AvInt}[ns]	56 730	±140	0,2%
τ_{AvAmp}[ns]	49 470	±130	0,3%

Figure S125: Left: Raw (experimental) time-resolved photoluminescence decay of [**ReLH$_2$(CO)$_3$CN**] in a frozen glassy DCM/MeOH (V:V = 1:1) at 77 K, including the residuals (λ_{exc} = 376.7 nm, λ_{em} = 510 nm). Right: Fitting parameters including pre-exponential factors and confidence limits.

IV. Aggregation study

Photophysical measurements of the crystalline solids

Time-resolved multiphoton micro(spectro)scopy

Time-resolved multiphoton micro(spectro)scopy was performed using a fluorescence microscope (IX 73 from Olympus) equipped with a complete confocal system, a laser combining unit (LCU), an inverted microscope body, and a multichannel detection unit (Multiharp 150, PicoQuant) equipped with diode lasers. These lasers provide adjustable output power and repetition rates up to 80 MHz, all within a compact fiber-coupled unit with wavelengths ranging from 375 to 640 nm). A FLIMbee galvo scanner is positioned between the main optical unit (MOU) and the microscope to achieve extremely stable scanning speeds while maintaining high positioning precision, enabling applications ranging from fast fluorescence lifetime imaging (rapidFLIM) to phosphorescence lifetime imaging measurements (PLIM).

For beam diagnostics, a charge-couple device (CCD) camera and a photodiode are available in the MOU of the microscope are used. The MOU is equipped with two detectors: a hybrid photomultiplier-based single-photon counting module (PMA Hybrid 40, PicoQuant) and a SPAD-based photon counting module (SPCM-AQR-14, Perkin-Elmer). Depending on the emission of the sample, various band-pass (BP) and low-pass (LP) filters were placed in front of these detectors as needed to acquire lifetime maps. Data acquisition was conducted using the unique time-tagged time-resolved (TTTR) measurement mode, allowing for simultaneous data acquisition on two channels. The data were processed and analyzed with the SymphoTime 64 (PicoQuant) software.

To couple the MicroTime 200 and the FluoTime 300 instruments, a fiber coupler was employed, enabling the spectrometer to record either steady-state or time-resolved luminescence spectra and decays from a sample mounted on the microscope. Luminescence micrographs were acquired using the aforementioned microscope, equipped with a X-CiteQ Lamp module (Excelitas Technologies) as the excitation source and a UI-5580SE (IDS) digital camera. Depending on the photophysical properties of the sample, different band pass (BP) and low pass (LP) cubes were using accordingly.

Table S13: Collected luminescence decay data of [**PtLHX**] as crystalline solids at 298 K using the luminescence microscope.

**Sample**	**Monomer / ns**	**Aggregate / ns**
[**PtLHCl**]	τ_1 = 1760 ± 20 (30%)<br>τ_2 = 646 ± 4 (70%)<br>τ_{av_amp} = 972 ± 4	τ_1 = 279 ± 9 (53%)<br>τ_2 = 77 ± 2 (47%)<br>τ_{av_amp} = 184 ± 2
[**PtLHCN**]	τ_1 = 8300 ± 400 (21%)<br>τ_2 = 2230 ± 20 (79%)<br>τ_{av_amp} = 3510 ± 50	τ_1 = 330 ± 20 (26%)<br>τ_2 = 91 ± 5 (74%)<br>τ_{av_amp} = 154 ± 3

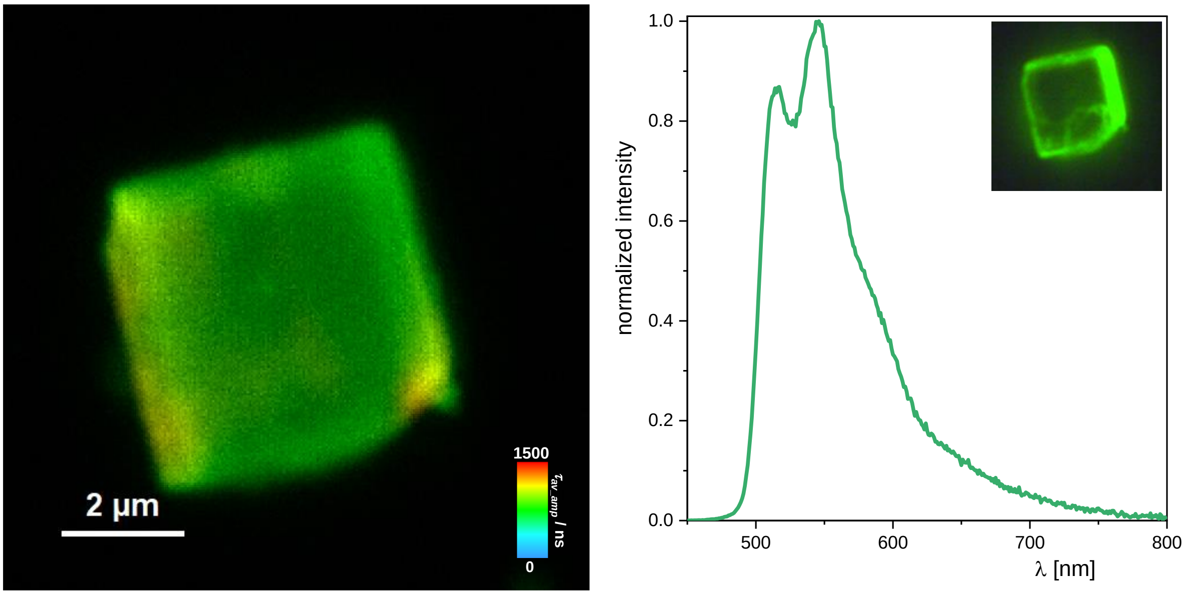

Figure S126: Left: Lifetime map measured by PLIM of the green-crystalline phase of [**PtLHCl**]. Right: Experimental photoluminescence spectrum of the complex in the crystalline state at 298 K (normalized to the highest intensity), measured using a photoluminescence spectrometer (FT300, PicoQuant) coupled to the confocal microscope. Luminescence micrograph of the crystal is shown as inset (λ_{exc} = 375 ± 20 nm).

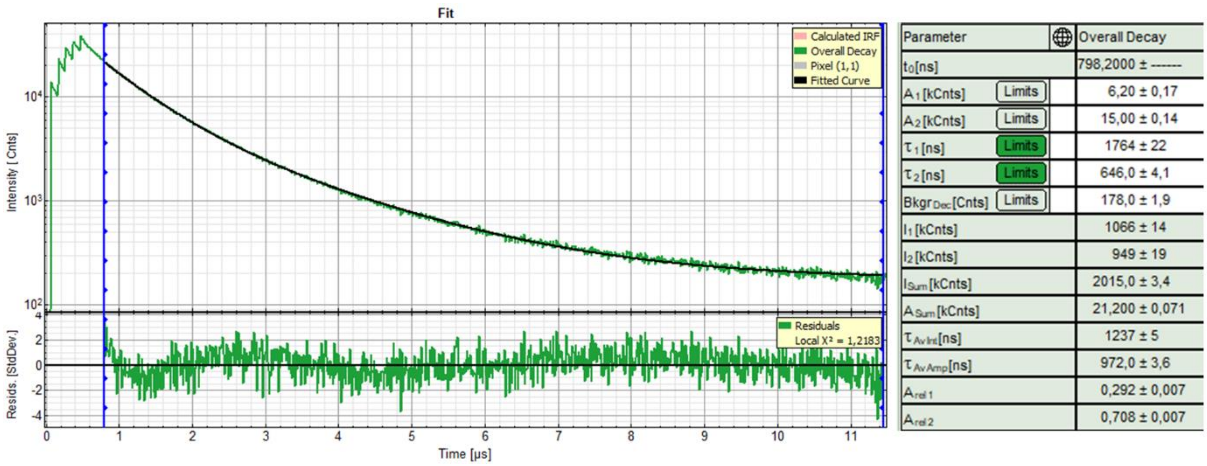

Parameter		Overall Decay
t0[ns]		798,2000 ± ------
A1 [kCnts]	Limits	6,20 ± 0,17
A2 [kCnts]	Limits	15,00 ± 0,14
τ1 [ns]	Limits	1764 ± 22
τ2 [ns]	Limits	646,0 ± 4,1
BkgrDec[Cnts]	Limits	178,0 ± 1,9
I1 [kCnts]		1066 ± 14
I2 [kCnts]		949 ± 19
ISum [kCnts]		2015,0 ± 3,4
ASum [kCnts]		21,200 ± 0,071
τAvInt[ns]		1237 ± 5
τAvAmp[ns]		972,0 ± 3,6
Arel1		0,292 ± 0,007
Arel2		0,708 ± 0,007

Figure S127: Left: Time-resolved photoluminescence decay of green-crystalline [**PtLHCl**] at 298 K, including the residuals (λ_{exc} = 376 nm). Right: Fitting parameters including pre-exponential factors and confidence limits.

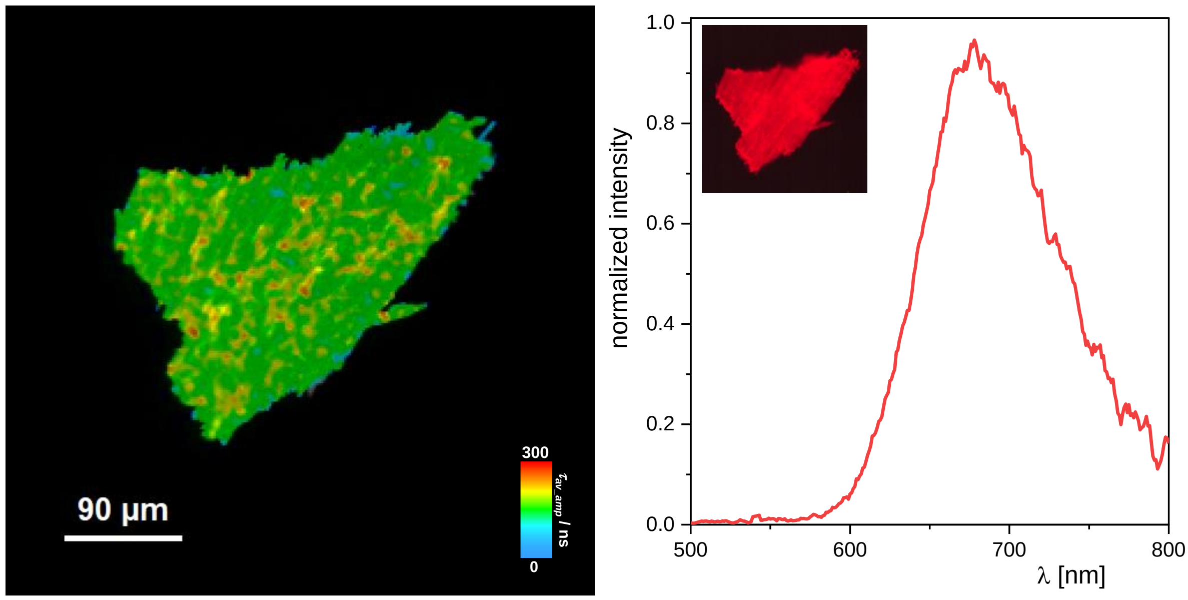

Figure S128: Left: Lifetime map measured by PLIM of the red-crystalline phase of [**PtLHCl**]. Right: Experimental photoluminescence spectrum of the complex in the crystalline state at 298 K (normalized to the highest intensity), measured using a photoluminescence spectrometer (FT300, PicoQuant) coupled to the confocal microscope. Luminescence micrograph of the crystal is shown as inset (λ_{exc} = 375 ± 20 nm).

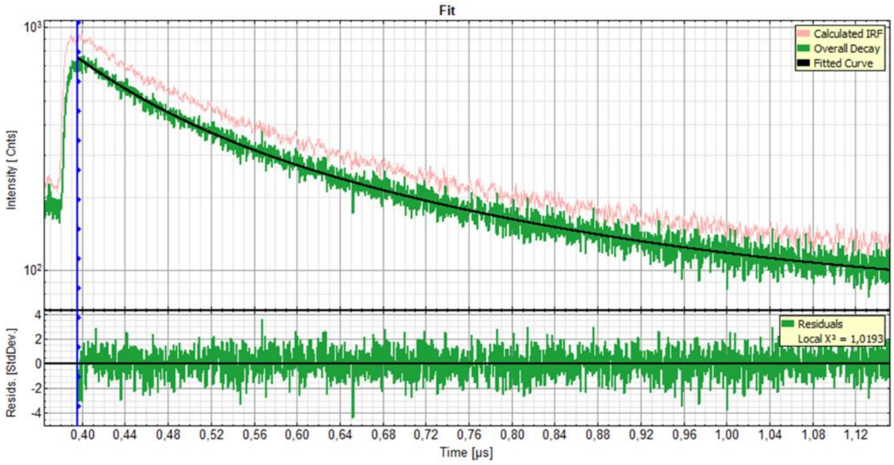

Parameter		Overall Decay
t_0[ns]		395,6000 ± ------
A_1[kCnts]	Limits	0,360 ± 0,014
A_2[kCnts]	Limits	0,315 ± 0,013
τ_1[ns]	Limits	279,0 ± 9,4
τ_2[ns]	Limits	77,0 ± 2,2
$Bkgr_{Dec}$[Cnts]	Limits	78,00 ± 0,81
I_1[kCnts]		313,0 ± 1,9
I_2[kCnts]		75,0 ± 6,5
I_{Sum}[kCnts]		389,0 ± 3,8
A_{Sum}[kCnts]		0,6743 ± 0,0046
τ_{AvInt}[ns]		240,0 ± 5,2
τ_{AvAmp}[ns]		184,0 ± 1,5
A_{rel1}		0,533 ± 0,023
A_{rel2}		0,467 ± 0,023

Figure S129: Left: Time-resolved photoluminescence decay of red-crystalline [**PtLHCl**] at 298 K, including the residuals (λ_{exc} = 376 nm). Right: Fitting parameters including pre-exponential factors and confidence limits.

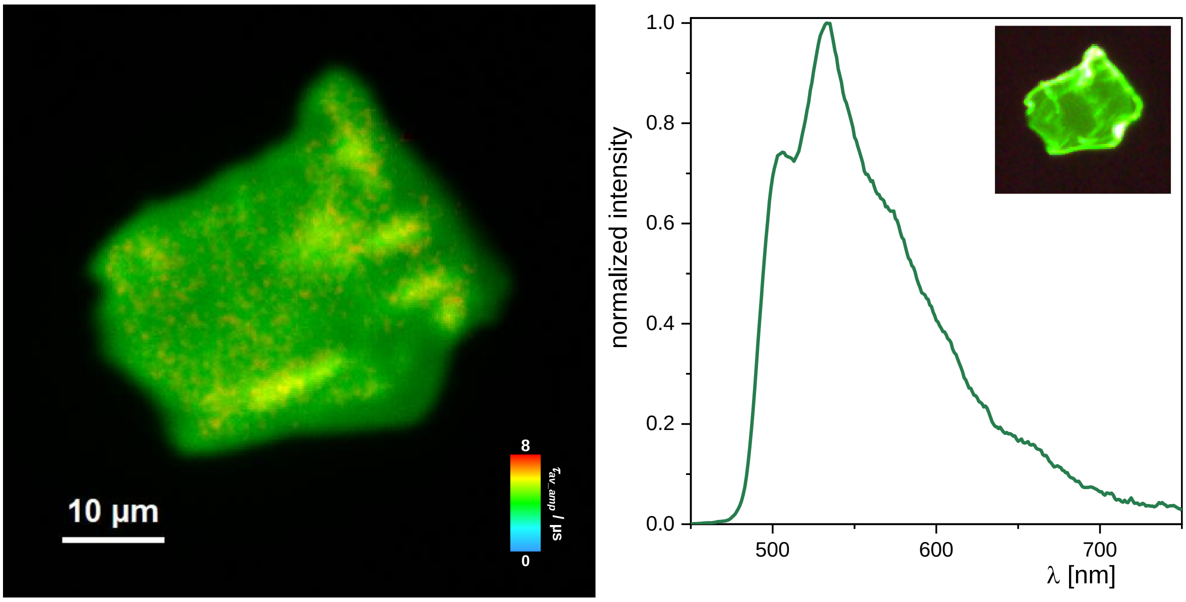

Figure S130: Left: Lifetime map measured by PLIM of the green-crystalline phase of [**PtLHCN**]. Right: Experimental photoluminescence spectrum of the complex in the crystalline state at 298 K (normalized to the highest intensity), measured using a photoluminescence spectrometer (FT300, PicoQuant) coupled to the confocal microscope. Luminescence micrograph of the crystal is shown as inset (λ_{exc} = 375 ± 20 nm).

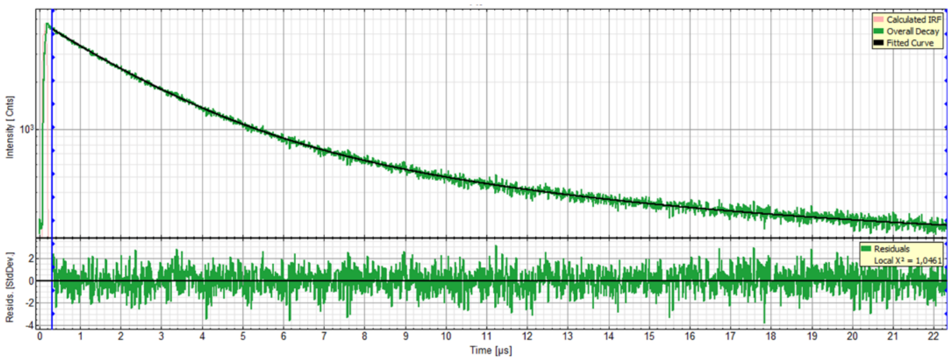

Parameter	Overall Decay
A_1 [kCnts]	0,873 ± 0,036
A_2 [kCnts]	3,240 ± 0,035
τ_1 [ns]	8270 ± 374
τ_2 [ns]	2230 ± 22
$Bkgr_{Dec}$ [Cnts]	187,0 ± 6,4
I_1 [kCnts]	705,0 ± 4,4
I_2 [kCnts]	706 ± 16
I_{Sum} [kCnts]	1411 ± 19
A_{Sum} [kCnts]	4,1150 ± 0,0064
τ_{AvInt} [ns]	5248 ± 177
τ_{AvAmp} [ns]	3512 ± 47
A_{rel1}	0,2122 ± 0,0086
A_{rel2}	0,7878 ± 0,0086

Figure S131: Left: Time-resolved photoluminescence decay of green-crystalline [**PtLHCN**] at 298 K, including the residuals (λ_{exc} = 376 nm). Right: Fitting parameters including pre-exponential factors and confidence limits.

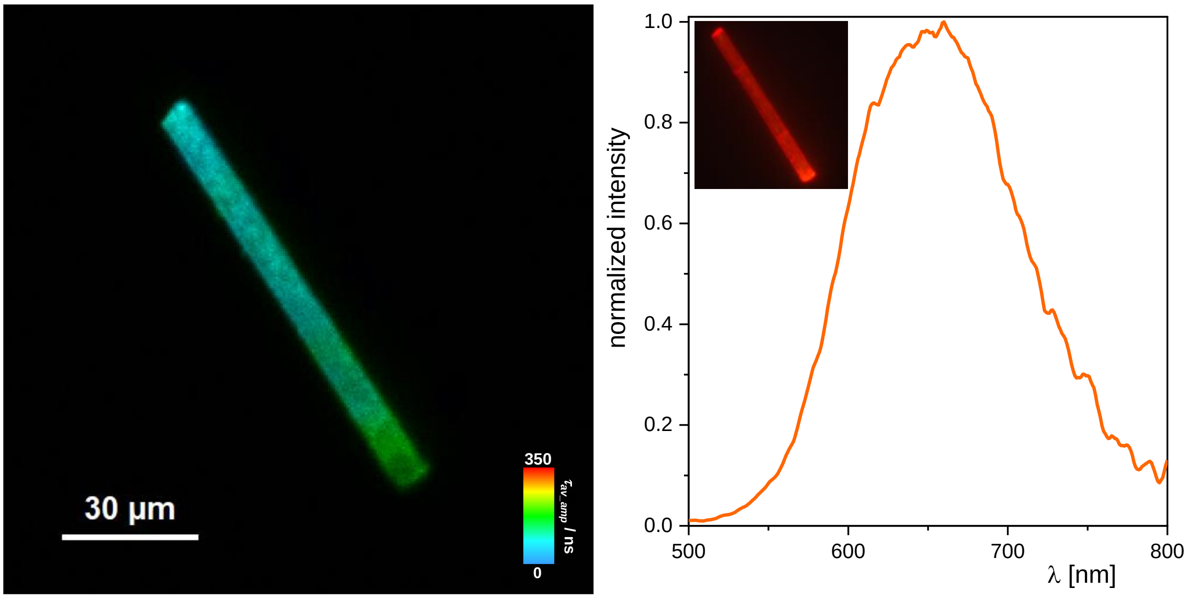

Figure S132: Left: Lifetime map measured by PLIM of the red-crystalline phase of [**PtLHCN**]. Right: Experimental photoluminescence spectrum of the complex in the crystalline state at 298 K (normalized to the highest intensity), measured using a photoluminescence spectrometer (FT300, PicoQuant) coupled to the confocal microscope. Luminescence micrograph of the crystal is shown as inset (λ_{exc} = 375 ± 20 nm).

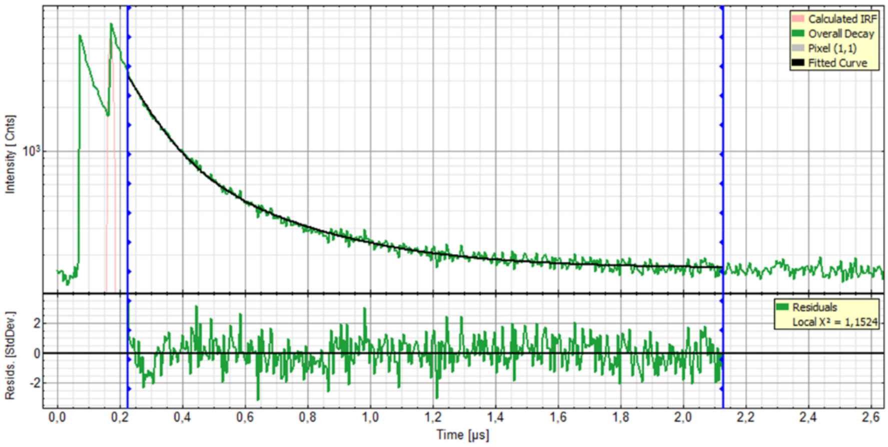

Parameter		Overall Decay
t_0[ns]		225,3000 ± ------
A_1[kCnts]	Limits	0,805 ± 0,083
A_2[kCnts]	Limits	2,340 ± 0,059
τ_1[ns]	Limits	334 ± 21
τ_2[ns]	Limits	91 ± 5
$Bkgr_{Dec}$[Cnts]	Limits	162,0 ± 2,2
I_1[kCnts]		52,0 ± 2,6
I_2[kCnts]		42,0 ± 2,6
I_{Sum}[kCnts]		94,0 ± 0,9
A_{Sum}[kCnts]		3,140 ± 0,047
τ_{AvInt}[ns]		226,0 ± 8,8
τ_{AvAmp}[ns]		154,0 ± 2,9
A_{rel1}		0,256 ± 0,024
A_{rel2}		0,744 ± 0,024

Figure S133: Left: Time-resolved photoluminescence decay of red-crystalline [**PtLHCN**] at 298 K, including the residuals (λ_{exc} = 376 nm). Right: Fitting parameters including pre-exponential factors and confidence limits.

Concentration-dependent photophysical measurements

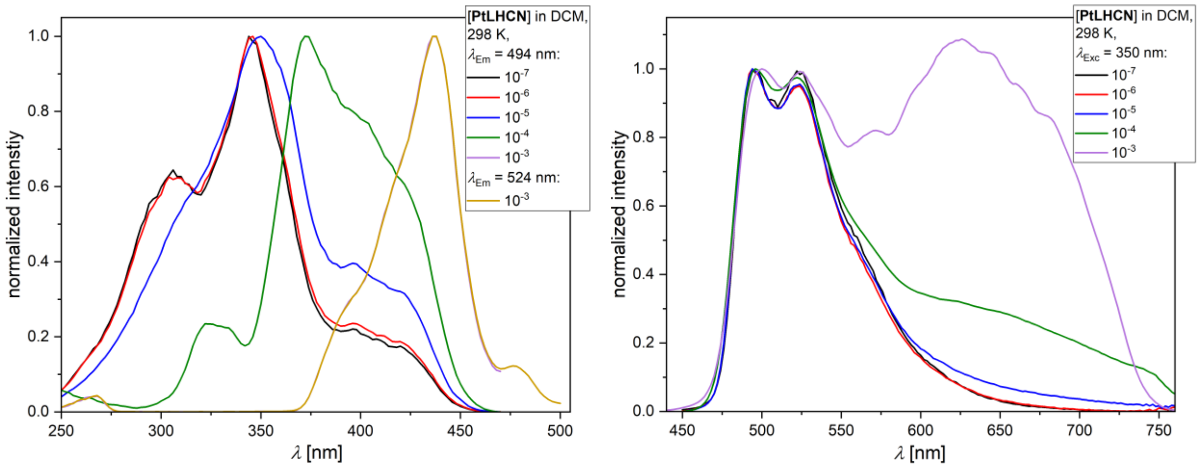

Figure S134: Excitation (left; λ_{Em} = 494 nm; λ_{Em} = 524 nm (orange)) and emission spectra (right) of [**PtLHCN**] at 298 K in DCM at different concentrations (c = 10^{-7} M (black); 10^{-6} (red); 10^{-5} M (blue); 10^{-4} M (green); 10^{-3} M (violet)). Spectra normalized to the highest intensity (left) or at the most blue-shifted maximum (right).

Table S14: Collected luminescence decay data of [**PtLHCN**] in DCM air-equilibrated and Ar-purged at 298 K. Values given in µs and rise-times as negative lifetimes. Values given in µs and rise-times in parentheses.

concentration	494 nm		600 nm	
	air	Ar	air	Ar
10^{-7}	1.158 ± 0.003	14.38 ± 0.01	-	14.41 ± 0.06
10^{-6}	1.087 ± 0.002	13.06 ± 0.01	1.085 ± 0.005	13.03 ± 0.01
10^{-5}	1.216 ± 0.002	7.73 ± 0.01	1.181 ± 0.003 (0.290 ± 0.020)	7.670 ± 0.007 (0.34 ± 0.05)
10^{-4}	0.875 ± 0.001	2.310 ± 0.002	0.888 ± 0.002 (0.237 ± 0.002)	2.313 ± 0.002 (0.381 ± 0.002)
10^{-3}	0.2837 ± 0.0002	0.3855 ± 0.0002	0.280 ± 0.002 (0.170 ± 0.003)	0.379 ± 0.003 (0.248 ± 0.004)

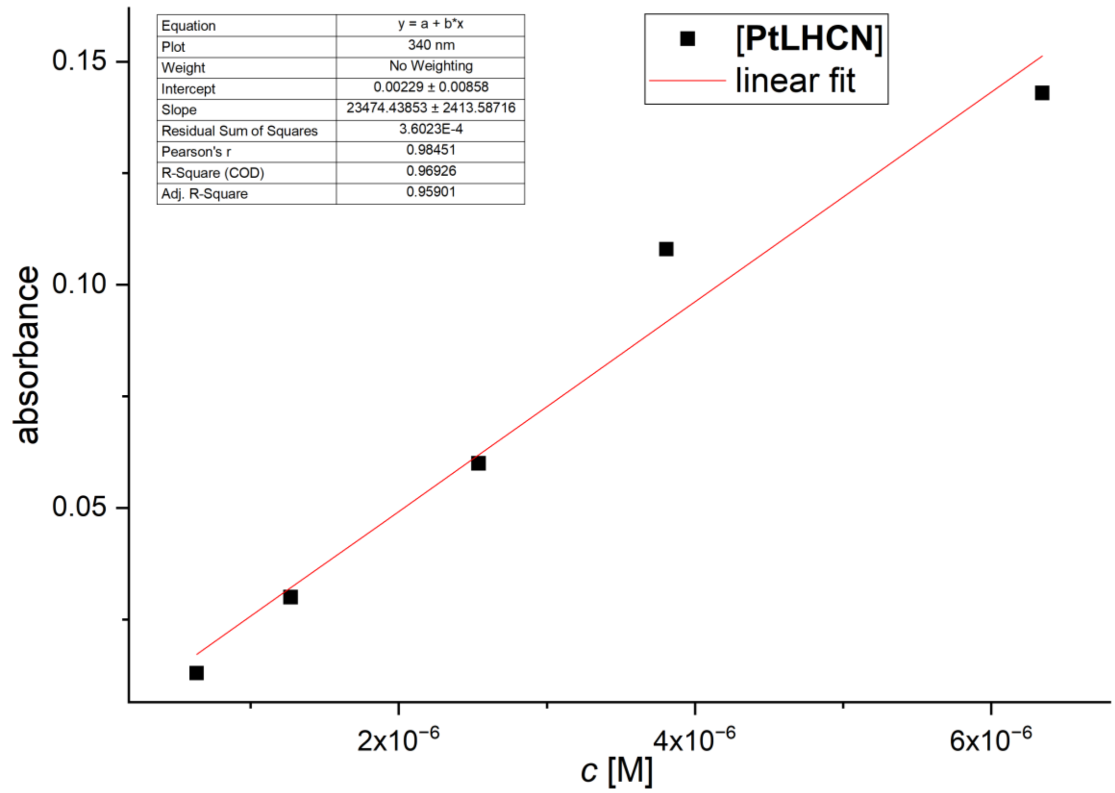

Figure S135: Lambert-Beer plot of the absorbance at 340 nm *vs*. the concentration of [**PtLHCN**].

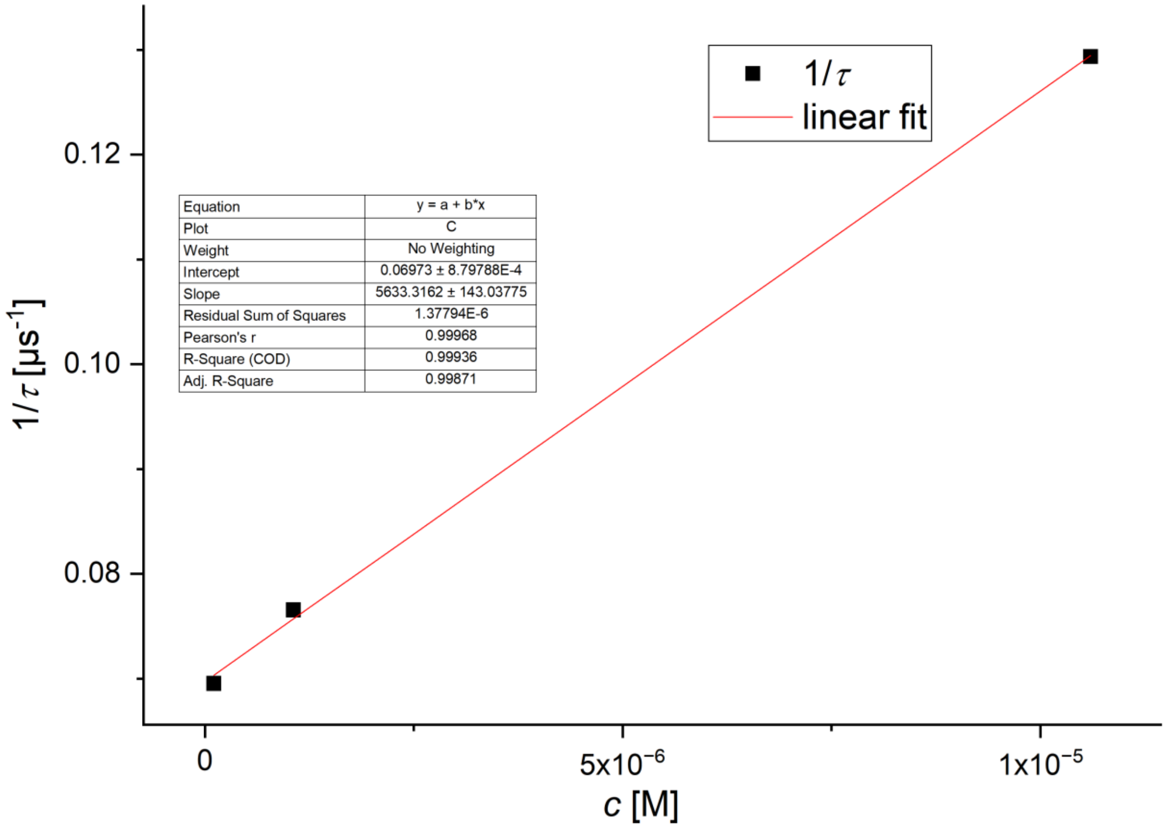

Figure S136: Plot of $1/\tau$ (measured at 494 nm) *vs*. the concentration of [**PtLHCN**] to determine the excimer formation rate constant.

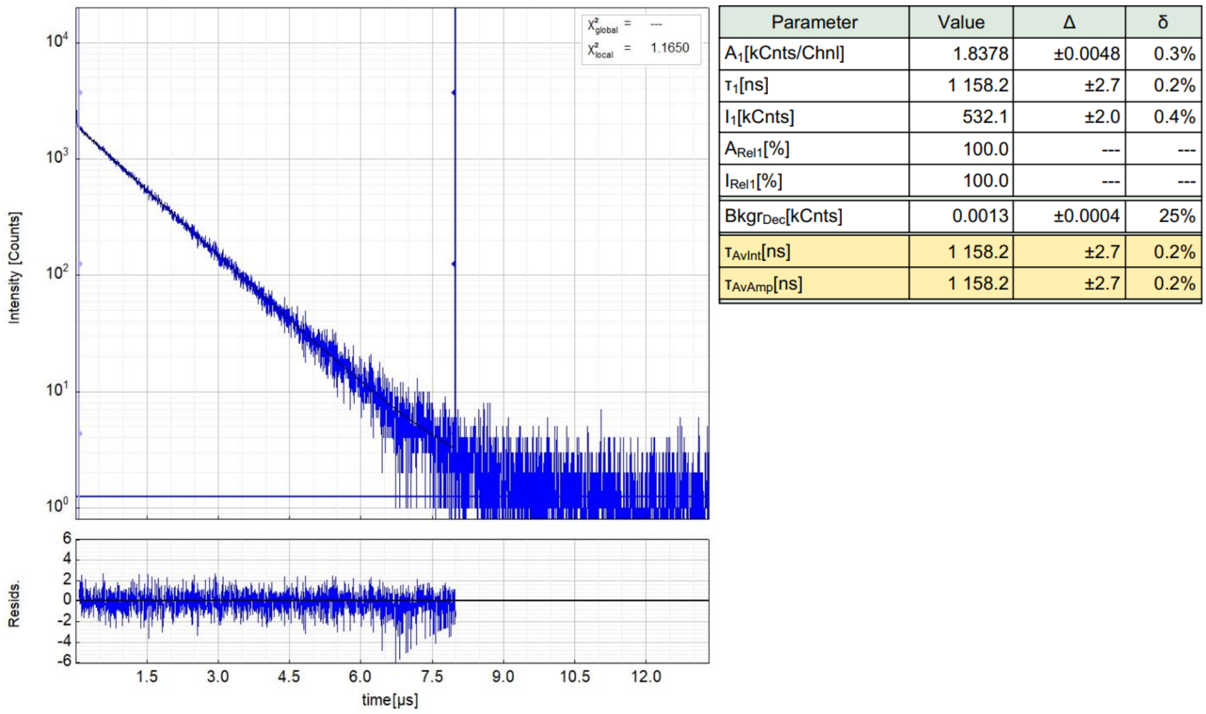

Parameter	Value	Δ	δ
A_1[kCnts/Chnl]	1.8378	±0.0048	0.3%
τ_1[ns]	1 158.2	±2.7	0.2%
I_1[kCnts]	532.1	±2.0	0.4%
A_{Rel1}[%]	100.0	---	---
I_{Rel1}[%]	100.0	---	---
$Bkgr_{Dec}$[kCnts]	0.0013	±0.0004	25%
τ_{AvInt}[ns]	1 158.2	±2.7	0.2%
τ_{AvAmp}[ns]	1 158.2	±2.7	0.2%

Figure S137: Left: Raw (experimental) time-resolved photoluminescence decay of [**PtLHCN**] in liquid DCM (air-equilibrated) at 298 K, including the residuals (c = 10^{-7} M; λ_{exc} = 376.7 nm, λ_{em} = 494 nm). Right: Fitting parameters including pre-exponential factors and confidence limits.

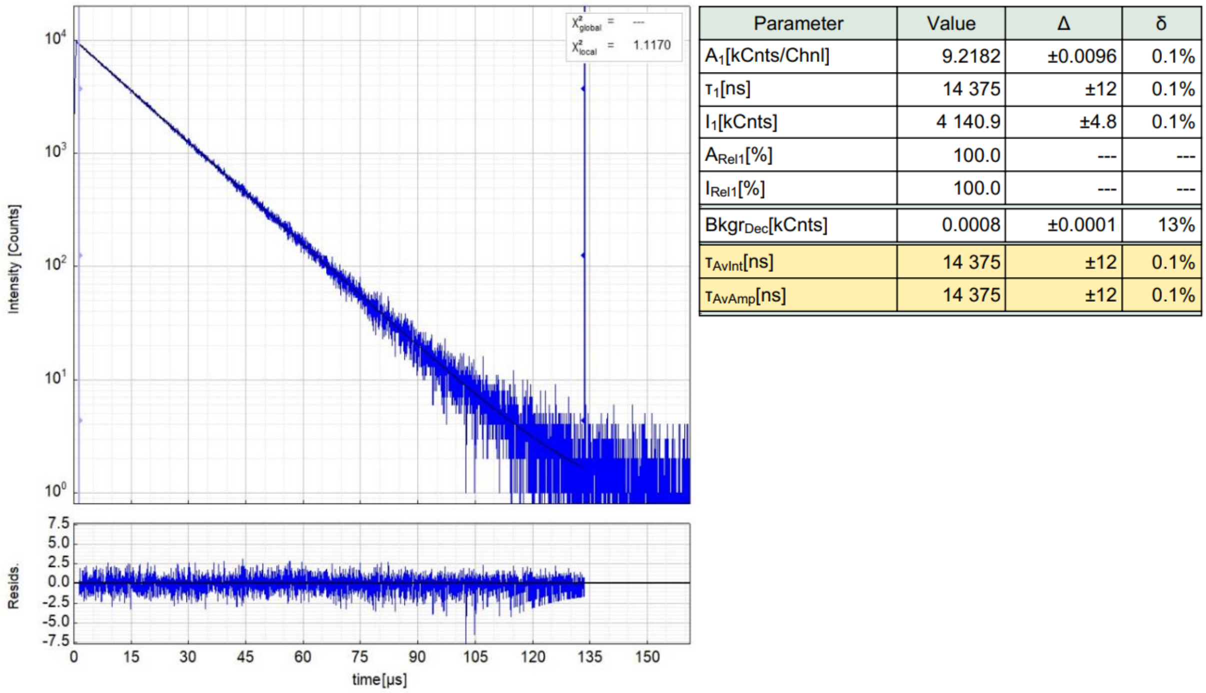

Parameter	Value	Δ	δ
A_1[kCnts/Chnl]	9.2182	±0.0096	0.1%
τ_1[ns]	14 375	±12	0.1%
I_1[kCnts]	4 140.9	±4.8	0.1%
A_{Rel1}[%]	100.0	---	---
I_{Rel1}[%]	100.0	---	---
$Bkgr_{Dec}$[kCnts]	0.0008	±0.0001	13%
τ_{AvInt}[ns]	14 375	±12	0.1%
τ_{AvAmp}[ns]	14 375	±12	0.1%

Figure S138: Left: Raw (experimental) time-resolved photoluminescence decay of [**PtLHCN**] in liquid DCM (Ar-purged) at 298 K, including the residuals (c = 10^{-7} M; λ_{exc} = 376.7 nm, λ_{em} = 494 nm). Right: Fitting parameters including pre-exponential factors and confidence limits.

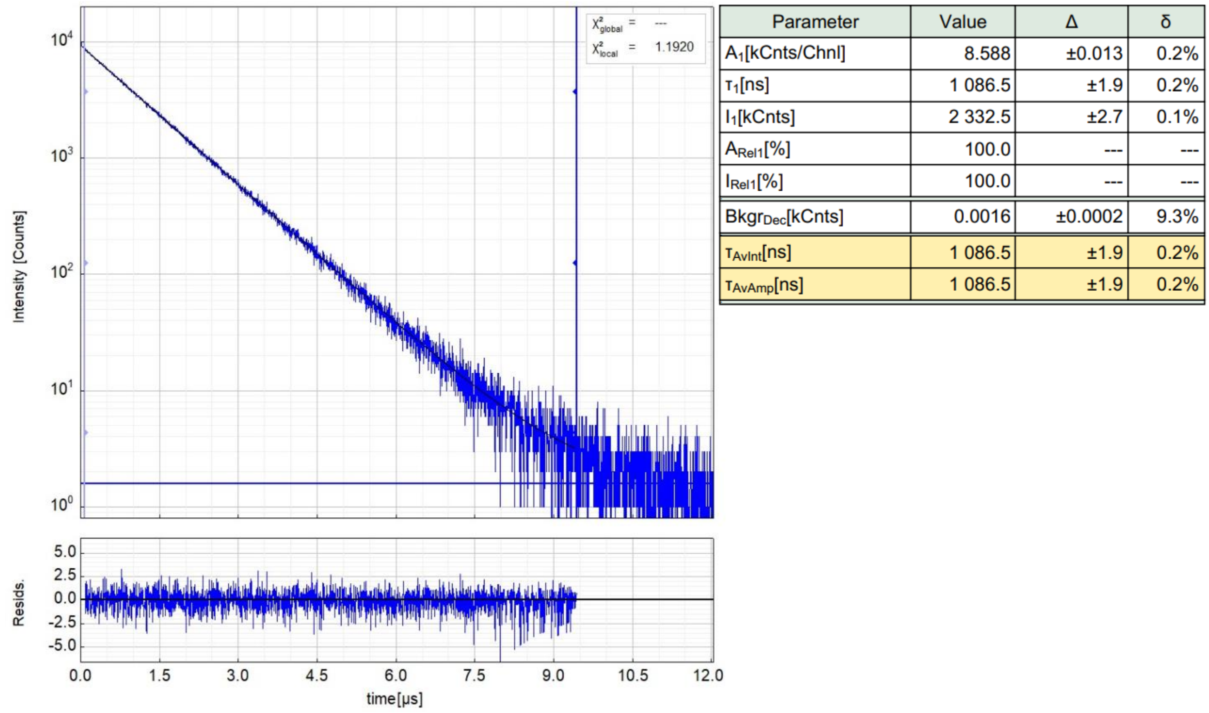

Parameter	Value	Δ	δ
A_1[kCnts/Chnl]	8.588	±0.013	0.2%
τ_1[ns]	1 086.5	±1.9	0.2%
I_1[kCnts]	2 332.5	±2.7	0.1%
A_{Rel1}[%]	100.0	---	---
I_{Rel1}[%]	100.0	---	---
$Bkgr_{Dec}$[kCnts]	0.0016	±0.0002	9.3%
τ_{AvInt}[ns]	1 086.5	±1.9	0.2%
τ_{AvAmp}[ns]	1 086.5	±1.9	0.2%

Figure S139: Left: Raw (experimental) time-resolved photoluminescence decay of [**PtLHCN**] in liquid DCM (air-equilibrated) at 298 K, including the residuals ($c = 10^{-6}$ M; λ_{exc} = 376.7 nm, λ_{em} = 494 nm). Right: Fitting parameters including pre-exponential factors and confidence limits.

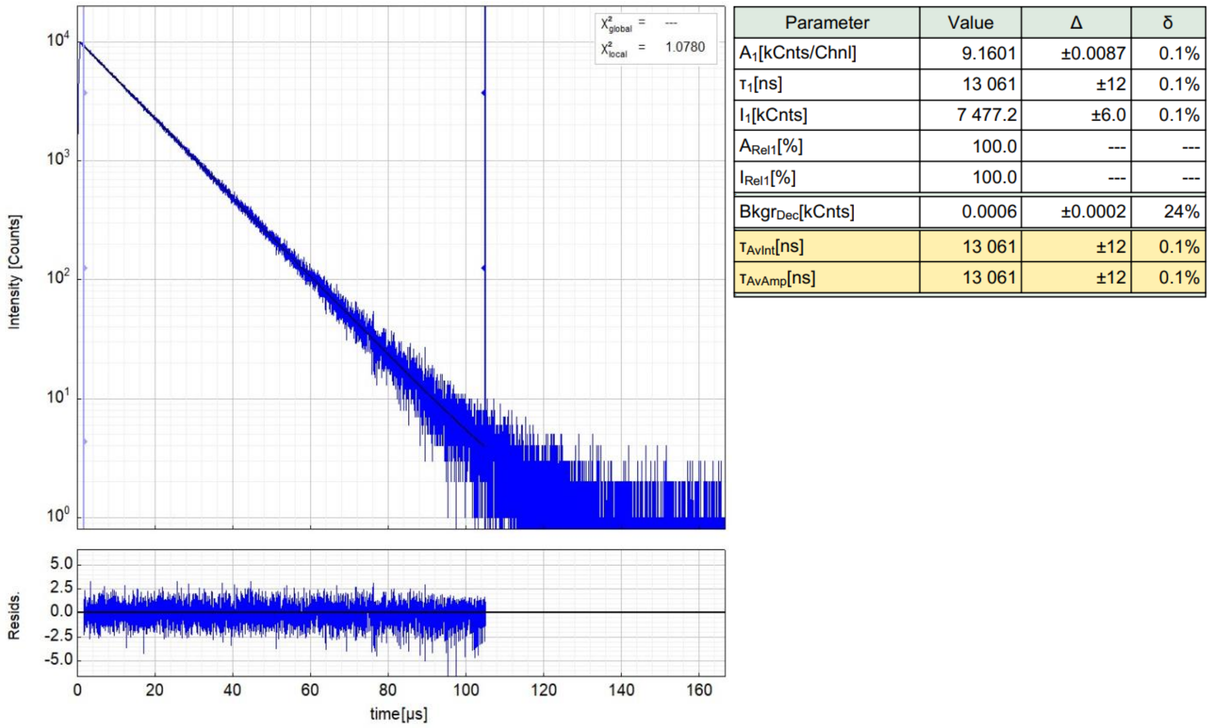

Parameter	Value	Δ	δ
A_1[kCnts/Chnl]	9.1601	±0.0087	0.1%
τ_1[ns]	13 061	±12	0.1%
I_1[kCnts]	7 477.2	±6.0	0.1%
A_{Rel1}[%]	100.0	---	---
I_{Rel1}[%]	100.0	---	---
$Bkgr_{Dec}$[kCnts]	0.0006	±0.0002	24%
τ_{AvInt}[ns]	13 061	±12	0.1%
τ_{AvAmp}[ns]	13 061	±12	0.1%

Figure S140: Left: Raw (experimental) time-resolved photoluminescence decay of [**PtLHCN**] in liquid DCM (Ar-purged) at 298 K, including the residuals ($c = 10^{-6}$ M; λ_{exc} = 376.7 nm, λ_{em} = 494 nm). Right: Fitting parameters including pre-exponential factors and confidence limits.

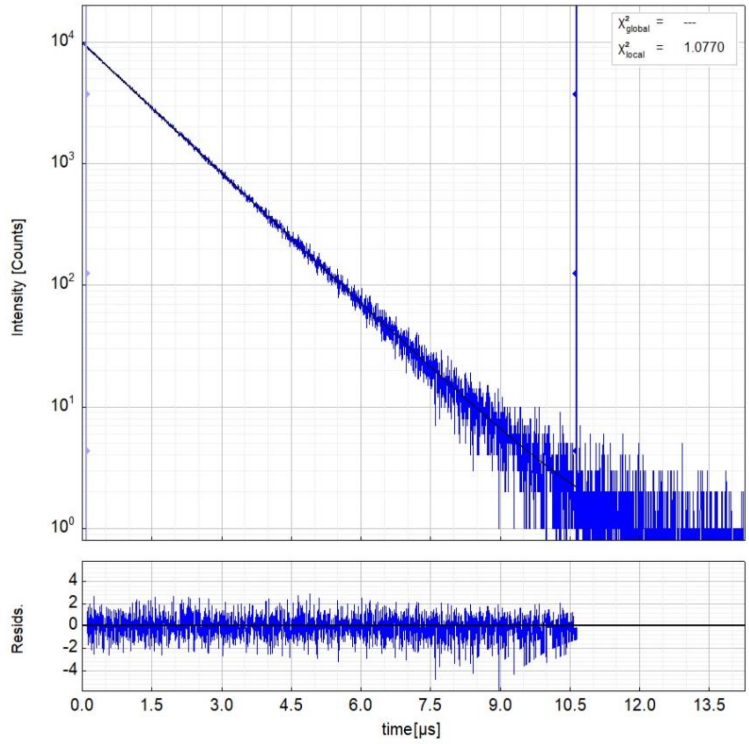

Parameter	Value	Δ	δ
A_1[kCnts/Chnl]	9.083	±0.017	0.2%
$τ_1$[ns]	1 216.1	±1.6	0.1%
I_1[kCnts]	2 761.3	±4.9	0.2%
A_{Rel1}[%]	100.0	---	---
I_{Rel1}[%]	100.0	---	---
$Bkgr_{Dec}$[kCnts]	0.0007	±0.0002	19%
$τ_{AvInt}$[ns]	1 216.1	±1.6	0.1%
$τ_{AvAmp}$[ns]	1 216.1	±1.6	0.1%

Figure S141: Left: Raw (experimental) time-resolved photoluminescence decay of [**PtLHCN**] in liquid DCM (air-equilibrated) at 298 K, including the residuals ($c = 10^{-5}$ M; λ_{exc} = 376.7 nm, λ_{em} = 494 nm). Right: Fitting parameters including pre-exponential factors and confidence limits.

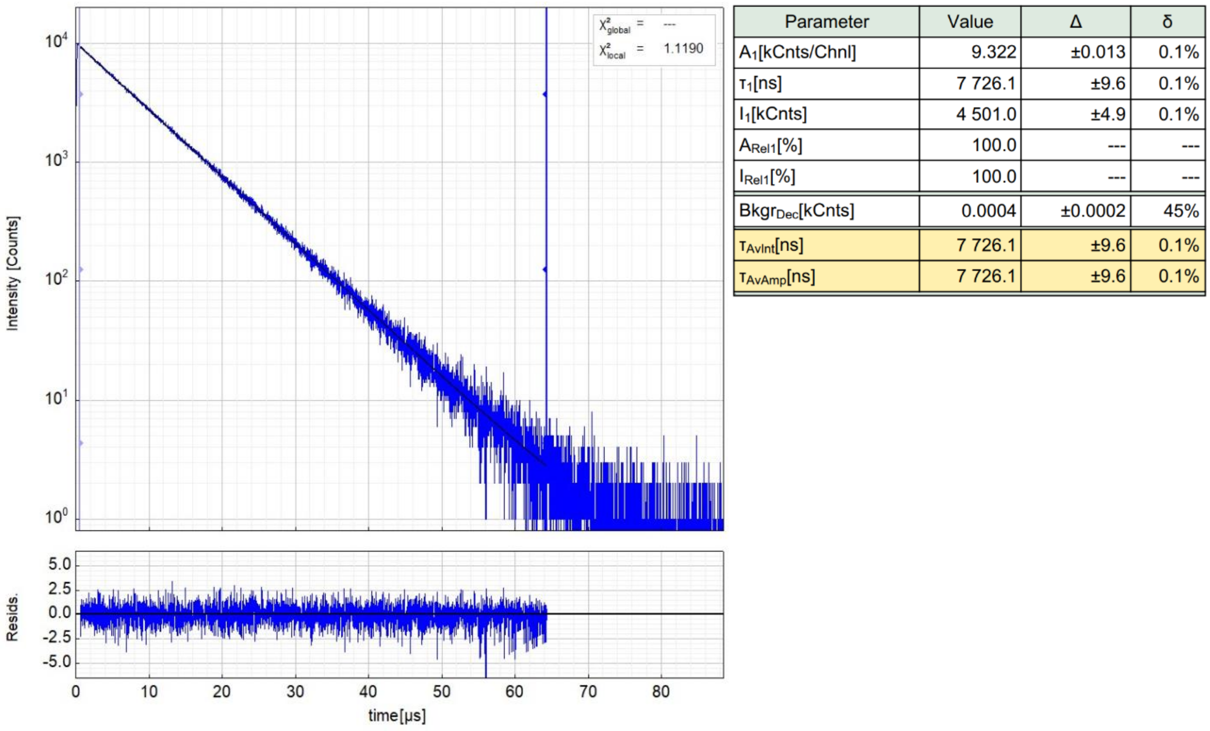

Parameter	Value	Δ	δ
A_1[kCnts/Chnl]	9.322	±0.013	0.1%
$τ_1$[ns]	7 726.1	±9.6	0.1%
I_1[kCnts]	4 501.0	±4.9	0.1%
A_{Rel1}[%]	100.0	---	---
I_{Rel1}[%]	100.0	---	---
$Bkgr_{Dec}$[kCnts]	0.0004	±0.0002	45%
$τ_{AvInt}$[ns]	7 726.1	±9.6	0.1%
$τ_{AvAmp}$[ns]	7 726.1	±9.6	0.1%

Figure S142: Left: Raw (experimental) time-resolved photoluminescence decay of [**PtLHCN**] in liquid DCM (Ar-purged) at 298 K, including the residuals ($c = 10^{-5}$ M; λ_{exc} = 376.7 nm, λ_{em} = 494 nm). Right: Fitting parameters including pre-exponential factors and confidence limits.

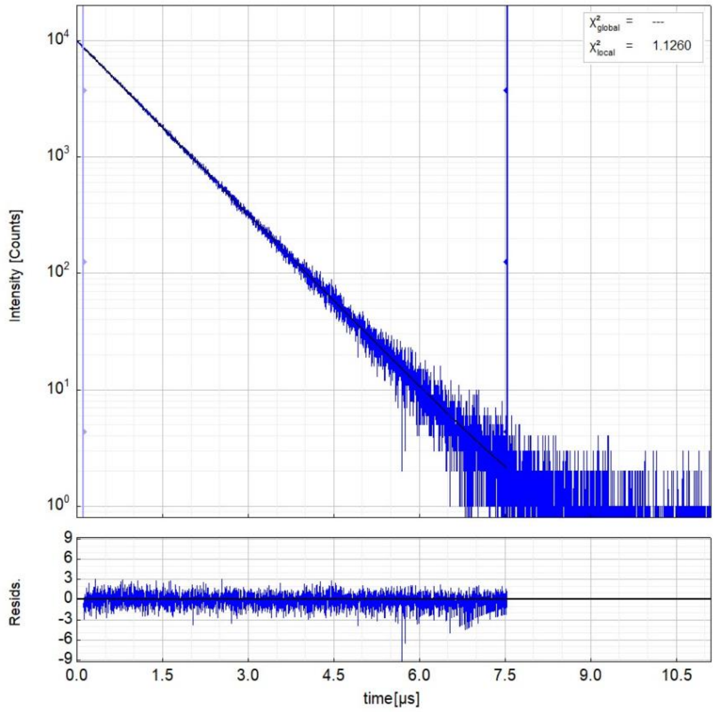

Parameter	Value	Δ	δ
A_1[kCnts/Chnl]	8.713	±0.011	0.1%
τ_1[ns]	875.11	±0.71	0.1%
I_1[kCnts]	3 812.4	±4.1	0.1%
A_{Rel1}[%]	100.0	---	---
I_{Rel1}[%]	100.0	---	---
$Bkgr_{Dec}$[kCnts]	0.0004	±0.0002	59%
τ_{AvInt}[ns]	875.11	±0.71	0.1%
τ_{AvAmp}[ns]	875.11	±0.71	0.1%

Figure S143: Left: Raw (experimental) time-resolved photoluminescence decay of [**PtLHCN**] in liquid DCM (air-equilibrated) at 298 K, including the residuals (c = 10^{-4} M; λ_{exc} = 376.7 nm, λ_{em} = 494 nm). Right: Fitting parameters including pre-exponential factors and confidence limits.

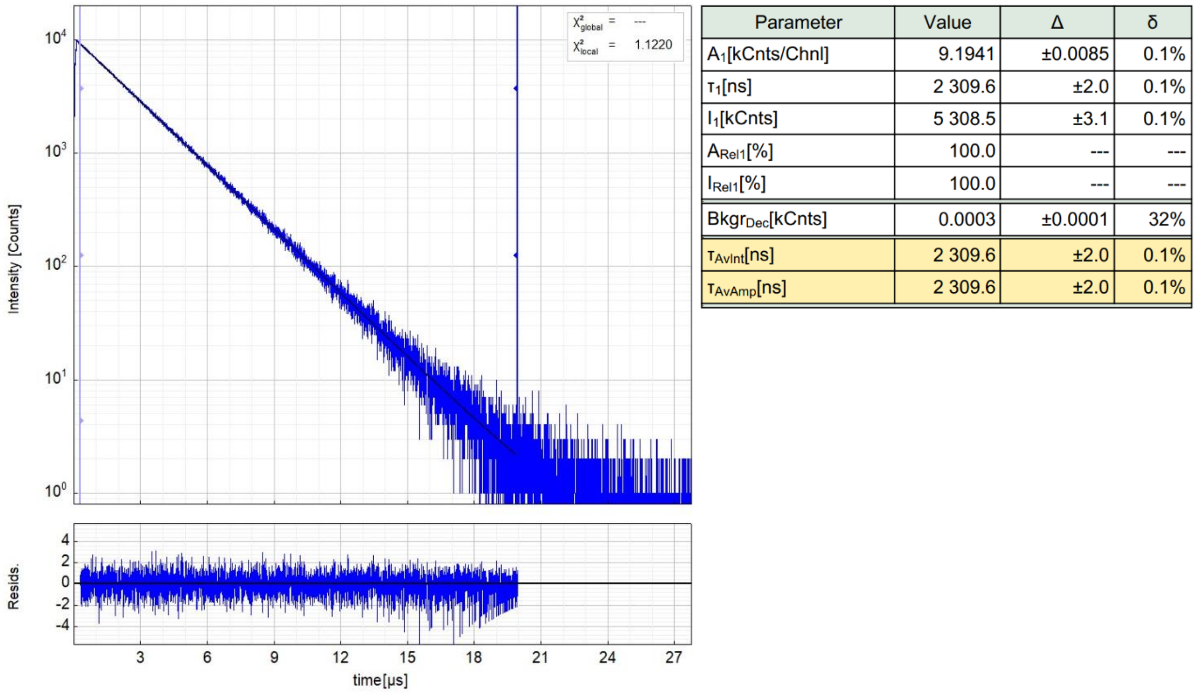

Parameter	Value	Δ	δ
A_1[kCnts/Chnl]	9.1941	±0.0085	0.1%
τ_1[ns]	2 309.6	±2.0	0.1%
I_1[kCnts]	5 308.5	±3.1	0.1%
A_{Rel1}[%]	100.0	---	---
I_{Rel1}[%]	100.0	---	---
$Bkgr_{Dec}$[kCnts]	0.0003	±0.0001	32%
τ_{AvInt}[ns]	2 309.6	±2.0	0.1%
τ_{AvAmp}[ns]	2 309.6	±2.0	0.1%

Figure S144: Left: Raw (experimental) time-resolved photoluminescence decay of [**PtLHCN**] in liquid DCM (Ar-purged) at 298 K, including the residuals (c = 10^{-4} M; λ_{exc} = 376.7 nm, λ_{em} = 494 nm). Right: Fitting parameters including pre-exponential factors and confidence limits.

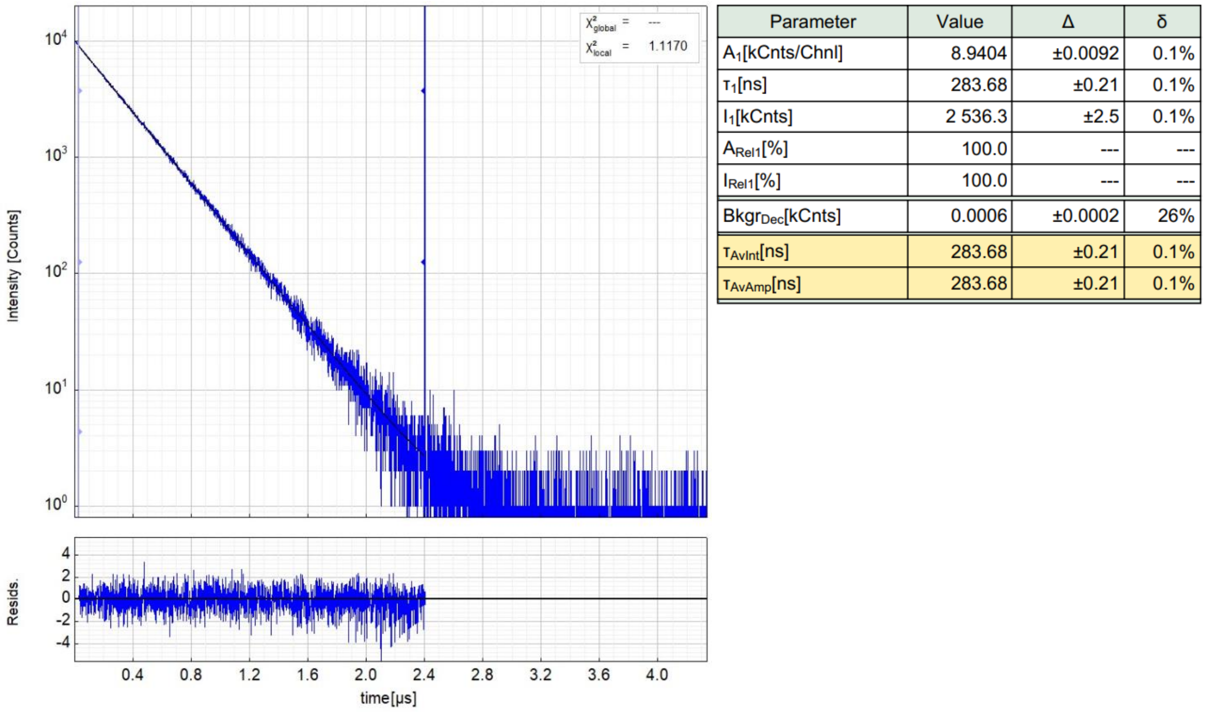

Parameter	Value	Δ	δ
A_1[kCnts/Chnl]	8.9404	±0.0092	0.1%
τ_1[ns]	283.68	±0.21	0.1%
I_1[kCnts]	2 536.3	±2.5	0.1%
A_{Rel1}[%]	100.0	---	---
I_{Rel1}[%]	100.0	---	---
$Bkgr_{Dec}$[kCnts]	0.0006	±0.0002	26%
τ_{AvInt}[ns]	283.68	±0.21	0.1%
τ_{AvAmp}[ns]	283.68	±0.21	0.1%

Figure S145: Left: Raw (experimental) time-resolved photoluminescence decay of [**PtLHCN**] in liquid DCM (air-equilibrated) at 298 K, including the residuals (c = 10^{-3} M; λ_{exc} = 376.7 nm, λ_{em} = 494 nm). Right: Fitting parameters including pre-exponential factors and confidence limits.

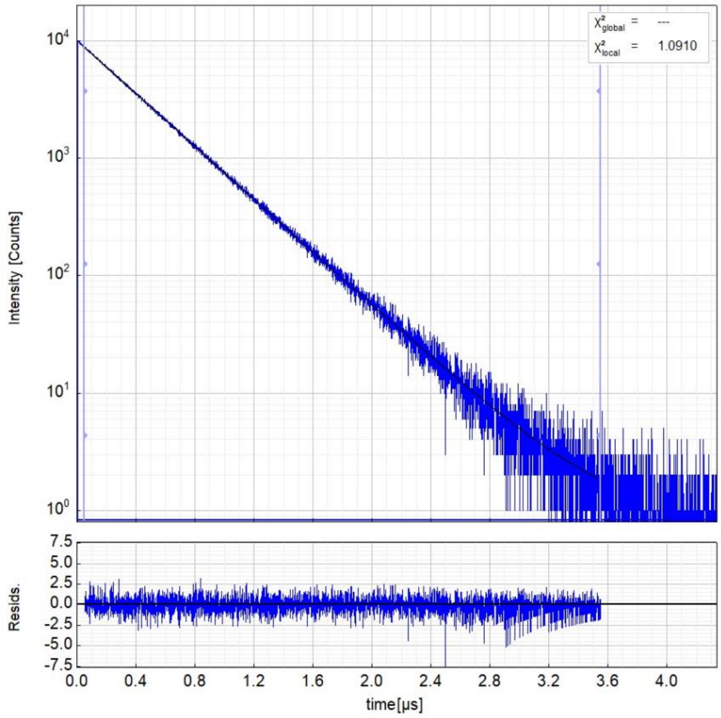

Parameter	Value	Δ	δ
A_1[kCnts/Chnl]	8.731	±0.014	0.2%
τ_1[ns]	385.51	±0.42	0.1%
I_1[kCnts]	3 365.7	±3.3	0.1%
A_{Rel1}[%]	100.0	---	---
I_{Rel1}[%]	100.0	---	---
$Bkgr_{Dec}$[kCnts]	0.0009	±0.0002	21%
τ_{AvInt}[ns]	385.51	±0.42	0.1%
τ_{AvAmp}[ns]	385.51	±0.42	0.1%

Figure S146: Left: Raw (experimental) time-resolved photoluminescence decay of [**PtLHCN**] in liquid DCM (Ar-purged) at 298 K, including the residuals (c = 10^{-3} M; λ_{exc} = 376.7 nm, λ_{em} = 494 nm). Right: Fitting parameters including pre-exponential factors and confidence limits.

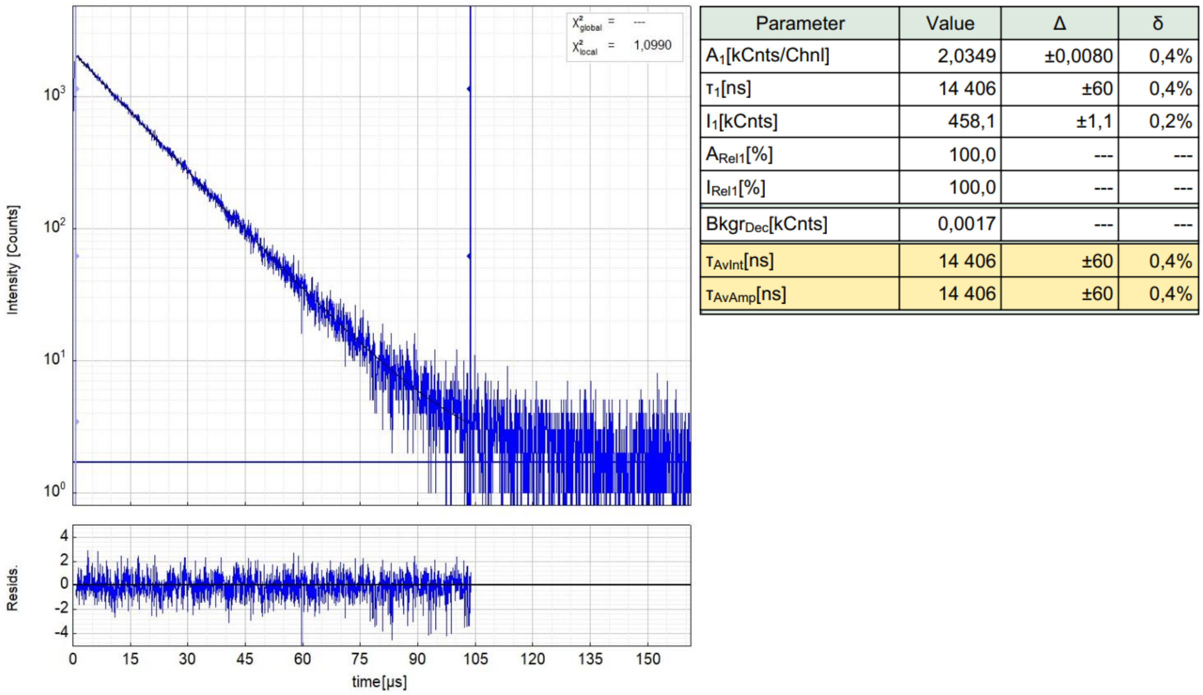

Parameter	Value	Δ	δ
A_1[kCnts/Chnl]	2,0349	±0,0080	0,4%
τ_1[ns]	14 406	±60	0,4%
I_1[kCnts]	458,1	±1,1	0,2%
A_{Rel1}[%]	100,0	---	---
I_{Rel1}[%]	100,0	---	---
$Bkgr_{Dec}$[kCnts]	0,0017	---	---
τ_{AvInt}[ns]	14 406	±60	0,4%
τ_{AvAmp}[ns]	14 406	±60	0,4%

Figure S147: Left: Raw (experimental) time-resolved photoluminescence decay of [**PtLHCN**] in liquid DCM (Ar-purged) at 298 K, including the residuals (c = 10^{-7} M; λ_{exc} = 376.7 nm, λ_{em} = 600 nm). Right: Fitting parameters including pre-exponential factors and confidence limits.

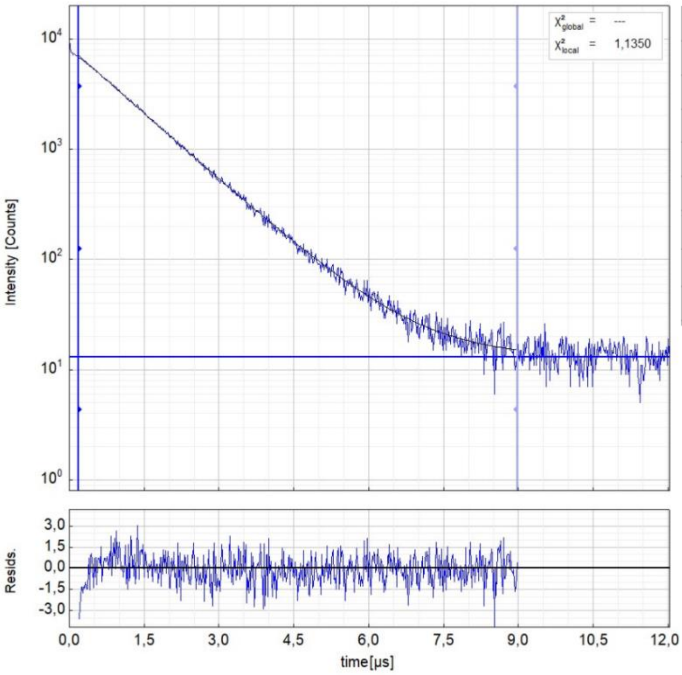

Parameter	Value	Δ	δ
A_1[kCnts/Chnl]	7,008	±0,053	0,8%
τ_1[ns]	1 084,9	±5,2	0,5%
I_1[kCnts]	475,2	±2,0	0,4%
A_{Rel1}[%]	100,0	---	---
I_{Rel1}[%]	100,0	---	---
$Bkgr_{Dec}$[kCnts]	0,0130	±0,0012	8,7%
τ_{AvInt}[ns]	1 084,9	±5,2	0,5%
τ_{AvAmp}[ns]	1 084,9	±5,2	0,5%

Figure S148: Left: Raw (experimental) time-resolved photoluminescence decay of [**PtLHCN**] in liquid DCM (air-equilibrated) at 298 K, including the residuals (c = 10^{-6} M; λ_{exc} = 376.7 nm, λ_{em} = 600 nm). Right: Fitting parameters including pre-exponential factors and confidence limits.

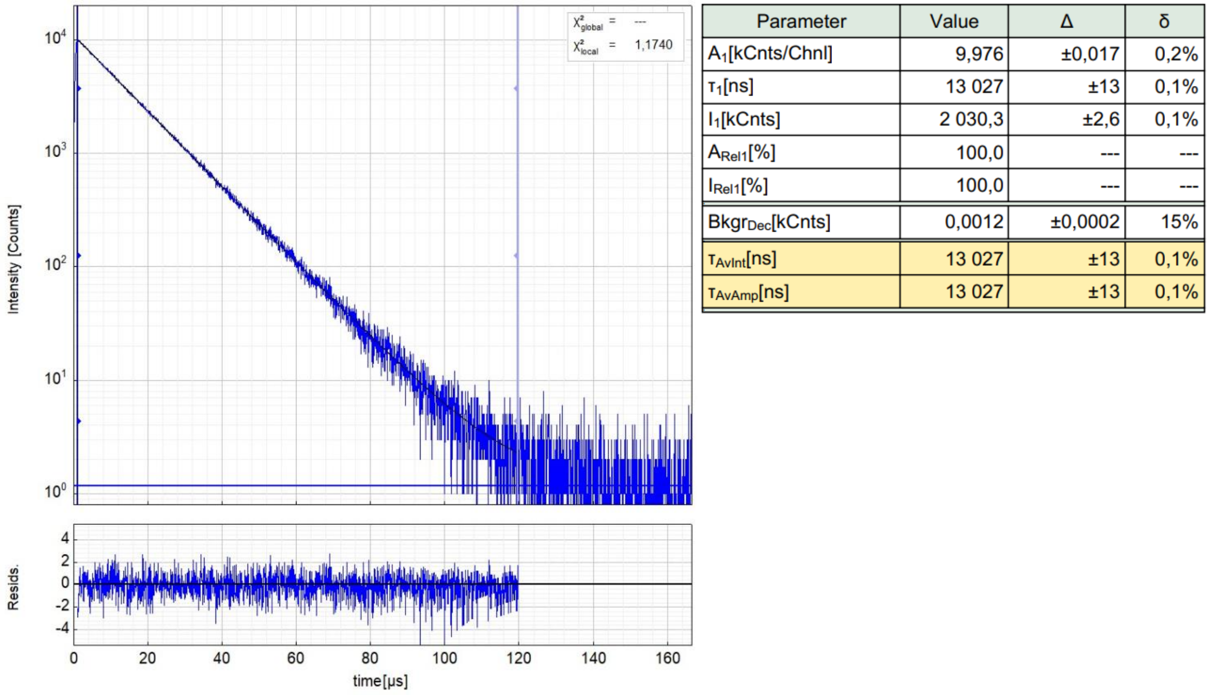

Parameter	Value	Δ	δ
A_1[kCnts/Chnl]	9,976	±0,017	0,2%
τ_1[ns]	13 027	±13	0,1%
I_1[kCnts]	2 030,3	±2,6	0,1%
A_{Rel1}[%]	100,0	---	---
I_{Rel1}[%]	100,0	---	---
$Bkgr_{Dec}$[kCnts]	0,0012	±0,0002	15%
τ_{AvInt}[ns]	13 027	±13	0,1%
τ_{AvAmp}[ns]	13 027	±13	0,1%

Figure S149: Left: Raw (experimental) time-resolved photoluminescence decay of [**PtLHCN**] in liquid DCM (Ar-purged) at 298 K, including the residuals ($c = 10^{-6}$ M; λ_{exc} = 376.7 nm, λ_{em} = 600 nm). Right: Fitting parameters including pre-exponential factors and confidence limits.

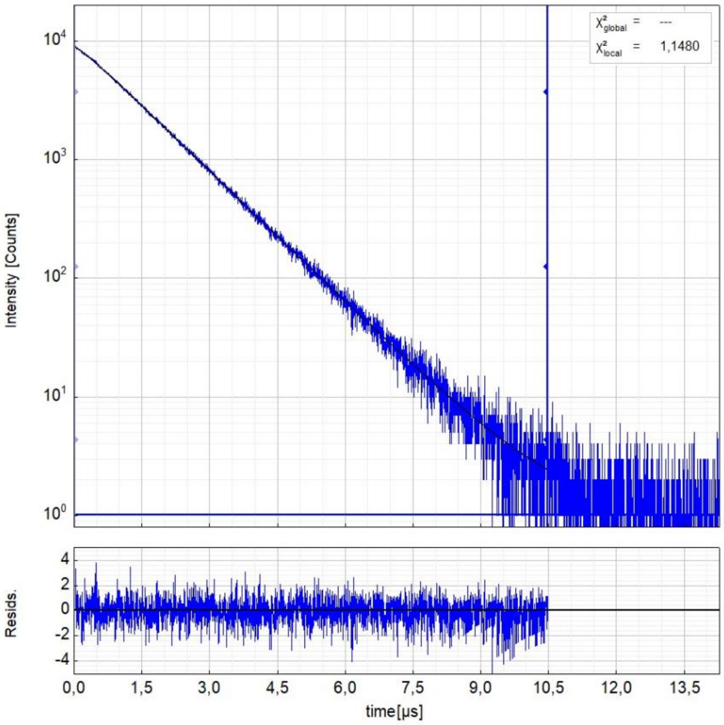

Parameter	Value	Δ	δ
A_1[kCnts/Chnl]	9,901	±0,059	0,6%
τ_1[ns]	1 181,4	±2,9	0,2%
I_1[kCnts]	2 924	±11	0,3%
A_{Rel1}[%]	100,0	---	---
I_{Rel1}[%]	100,0	---	---
A_2[kCnts/Chnl]	-1,065	±0,060	5,6%
τ_2[ns]	288	±23	7,9%
I_2[kCnts]	-76	±11	14%
A_{Rel2}[%]	0,0	---	---
I_{Rel2}[%]	0,0	---	---
$Bkgr_{Dec}$[kCnts]	0,0011	±0,0003	21%
τ_{AvInt}[ns]	1 181,4	±2,9	0,2%
τ_{AvAmp}[ns]	1 181,4	±2,9	0,2%

Figure S150: Left: Raw (experimental) time-resolved photoluminescence decay of [**PtLHCN**] in liquid DCM (air-equilibrated) at 298 K, including the residuals ($c = 10^{-5}$ M; λ_{exc} = 376.7 nm, λ_{em} = 600 nm). Right: Fitting parameters including pre-exponential factors and confidence limits.

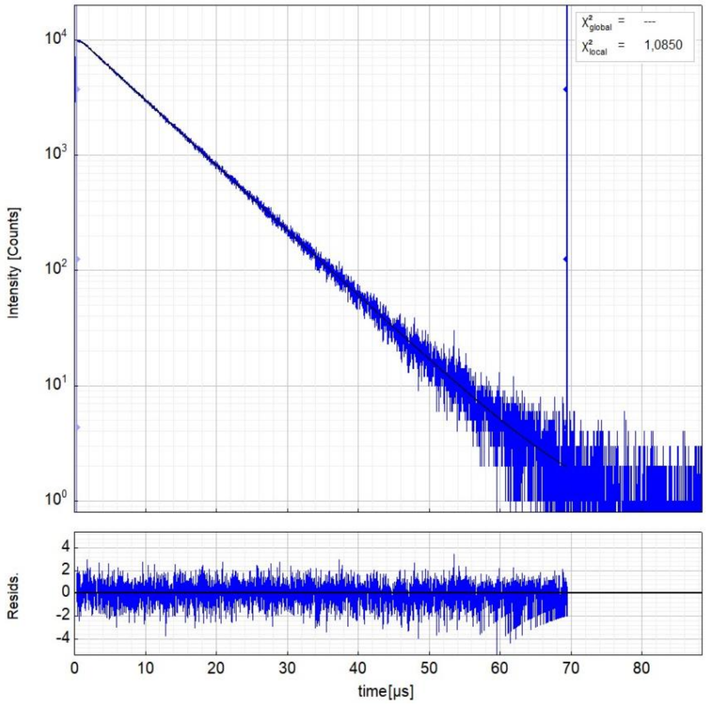

Parameter	Value	Δ	δ
A_1[kCnts/Chnl]	10,635	±0,011	0,1%
τ_1[ns]	7 670,4	±6,6	0,1%
I_1[kCnts]	5 098,1	±6,0	0,1%
A_{Rel1}[%]	100,0	---	---
I_{Rel1}[%]	100,0	---	---
A_2[kCnts/Chnl]	-0,993	±0,100	10%
τ_2[ns]	337	±48	14%
I_2[kCnts]	-20,9	±1,9	9,1%
A_{Rel2}[%]	0,0	---	---
I_{Rel2}[%]	0,0	---	---
$Bkgr_{Dec}$[kCnts]	0,0007	±0,0002	19%
τ_{AvInt}[ns]	7 670,4	±6,6	0,1%
τ_{AvAmp}[ns]	7 670,4	±6,6	0,1%

Figure S151: Left: Raw (experimental) time-resolved photoluminescence decay of [**PtLHCN**] in liquid DCM (Ar-purged) at 298 K, including the residuals ($c = 10^{-5}$ M; $\lambda_{exc} = 376.7$ nm, $\lambda_{em} = 600$ nm). Right: Fitting parameters including pre-exponential factors and confidence limits.

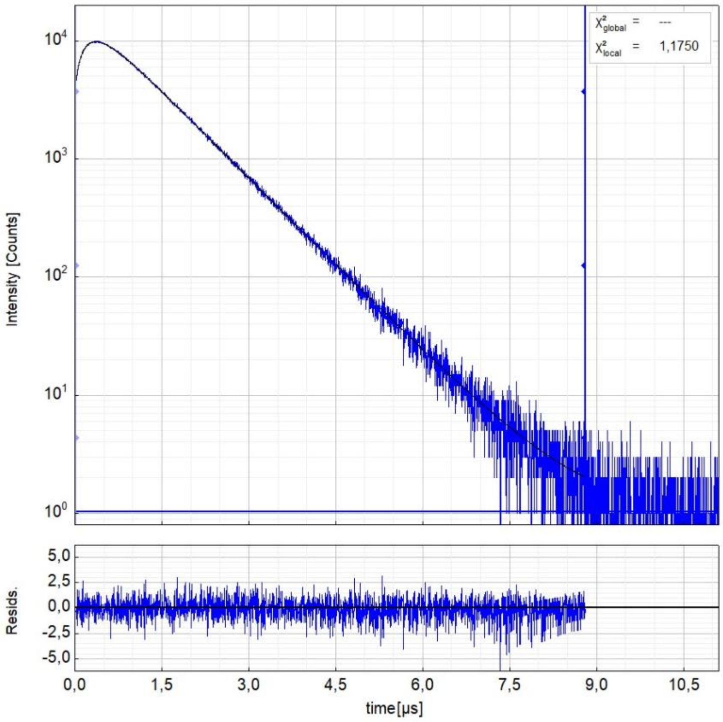

Parameter	Value	Δ	δ
A_1[kCnts/Chnl]	19,758	±0,098	0,5%
τ_1[ns]	888,0	±1,8	0,2%
I_1[kCnts]	4 386	±13	0,3%
A_{Rel1}[%]	100,0	---	---
I_{Rel1}[%]	100,0	---	---
A_2[kCnts/Chnl]	-15,229	±0,079	0,5%
τ_2[ns]	236,9	±2,2	0,9%
I_2[kCnts]	- 901	±13	1,4%
A_{Rel2}[%]	0,0	---	---
I_{Rel2}[%]	0,0	---	---
$Bkgr_{Dec}$[kCnts]	0,0011	±0,0001	8,2%
τ_{AvInt}[ns]	888,0	±1,8	0,2%
τ_{AvAmp}[ns]	888,0	±1,8	0,2%

Figure S152: Left: Raw (experimental) time-resolved photoluminescence decay of [**PtLHCN**] in liquid DCM (air-equilibrated) at 298 K, including the residuals ($c = 10^{-4}$ M; $\lambda_{exc} = 376.7$ nm, $\lambda_{em} = 600$ nm). Right: Fitting parameters including pre-exponential factors and confidence limits.

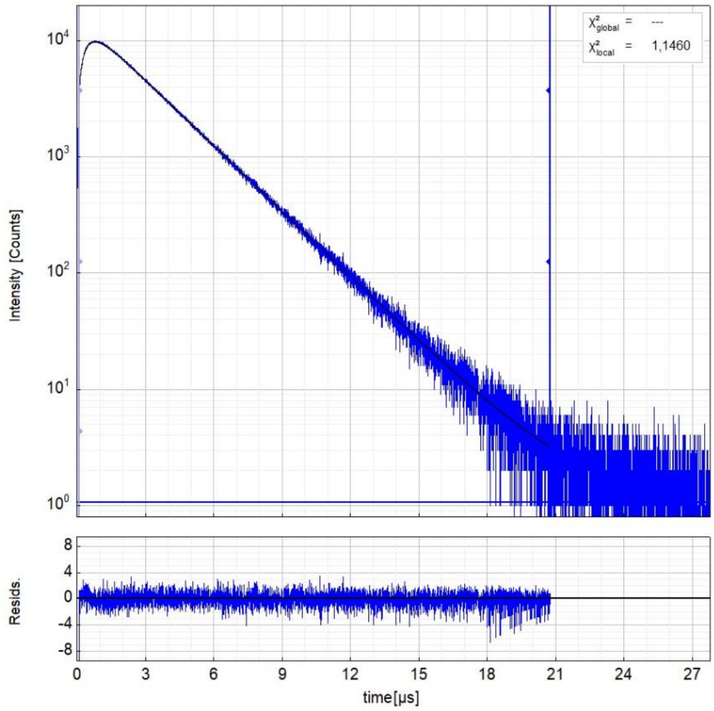

Parameter	Value	Δ	δ
A_1[kCnts/Chnl]	15,942	±0,022	0,1%
τ_1[ns]	2 312,9	±2,0	0,1%
I_1[kCnts]	9 217,7	±7,0	0,1%
A_{Rel1}[%]	100,0	---	---
I_{Rel1}[%]	100,0	---	---
A_2[kCnts/Chnl]	-12,282	±0,054	0,4%
τ_2[ns]	380,8	±1,9	0,5%
I_2[kCnts]	-1 169,1	±3,2	0,3%
A_{Rel2}[%]	0,0	---	---
I_{Rel2}[%]	0,0	---	---
$Bkgr_{Dec}$[kCnts]	0,0011	±0,0002	14%
τ_{AvInt}[ns]	2 312,9	±2,0	0,1%
τ_{AvAmp}[ns]	2 312,9	±2,0	0,1%

Figure S153: Left: Raw (experimental) time-resolved photoluminescence decay of [**PtLHCN**] in liquid DCM (Ar-purged) at 298 K, including the residuals ($c = 10^{-4}$ M; $\lambda_{exc} = 376.7$ nm, $\lambda_{em} = 600$ nm). Right: Fitting parameters including pre-exponential factors and confidence limits.

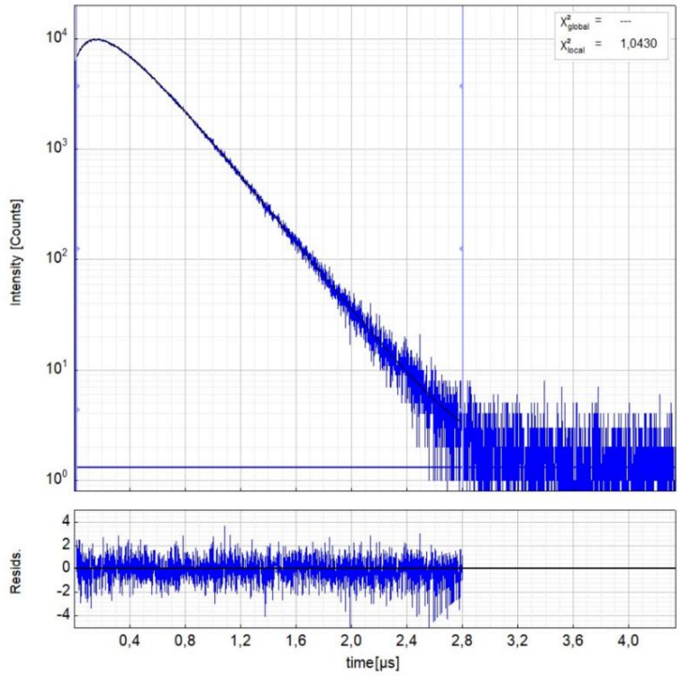

Parameter	Value	Δ	δ
A_1[kCnts/Chnl]	41,4	±1,7	3,9%
τ_1[ns]	280,1	±2,2	0,8%
I_1[kCnts]	11 600	±360	3,1%
A_{Rel1}[%]	100,0	---	---
I_{Rel1}[%]	100,0	---	---
A_2[kCnts/Chnl]	-34,8	±1,7	4,6%
τ_2[ns]	170,0	±2,5	1,4%
I_2[kCnts]	-5 900	±370	6,1%
A_{Rel2}[%]	0,0	---	---
I_{Rel2}[%]	0,0	---	---
$Bkgr_{Dec}$[kCnts]	0,0014	±0,0003	19%
τ_{AvInt}[ns]	280,1	±2,2	0,8%
τ_{AvAmp}[ns]	280,1	±2,2	0,8%

Figure S154: Left: Raw (experimental) time-resolved photoluminescence decay of [**PtLHCN**] in liquid DCM (air-equilibrated) at 298 K, including the residuals ($c = 10^{-3}$ M; $\lambda_{exc} = 376.7$ nm, $\lambda_{em} = 600$ nm). Right: Fitting parameters including pre-exponential factors and confidence limits.

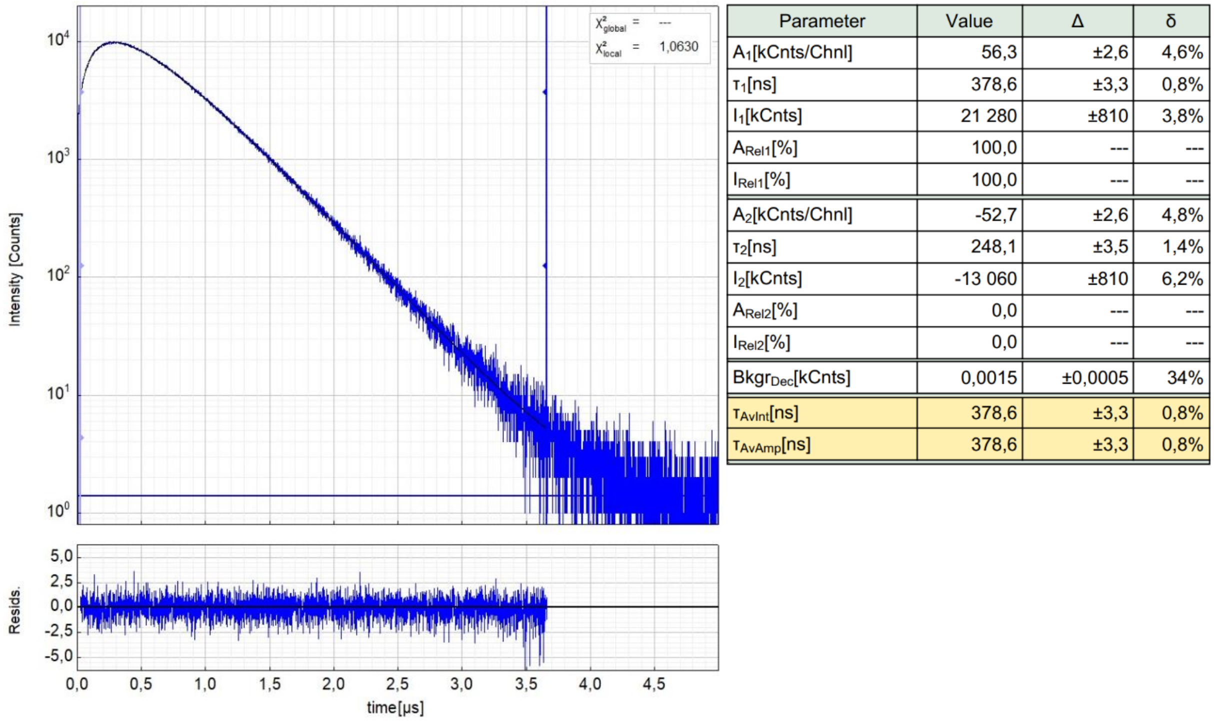

Parameter	Value	Δ	δ
A_1[kCnts/Chnl]	56,3	±2,6	4,6%
τ_1[ns]	378,6	±3,3	0,8%
I_1[kCnts]	21 280	±810	3,8%
A_{Rel1}[%]	100,0	---	---
I_{Rel1}[%]	100,0	---	---
A_2[kCnts/Chnl]	-52,7	±2,6	4,8%
τ_2[ns]	248,1	±3,5	1,4%
I_2[kCnts]	-13 060	±810	6,2%
A_{Rel2}[%]	0,0	---	---
I_{Rel2}[%]	0,0	---	---
$Bkgr_{Dec}$[kCnts]	0,0015	±0,0005	34%
τ_{AvInt}[ns]	378,6	±3,3	0,8%
τ_{AvAmp}[ns]	378,6	±3,3	0,8%

Figure S155: Left: Raw (experimental) time-resolved photoluminescence decay of [**PtLHCN**] in liquid DCM (Ar-purged) at 298 K, including the residuals (c = 10^{-3} M; λ_{exc} = 376.7 nm, λ_{em} = 600 nm). Right: Fitting parameters including pre-exponential factors and confidence limits.

Concentration- and temperature-dependent NMR measurements

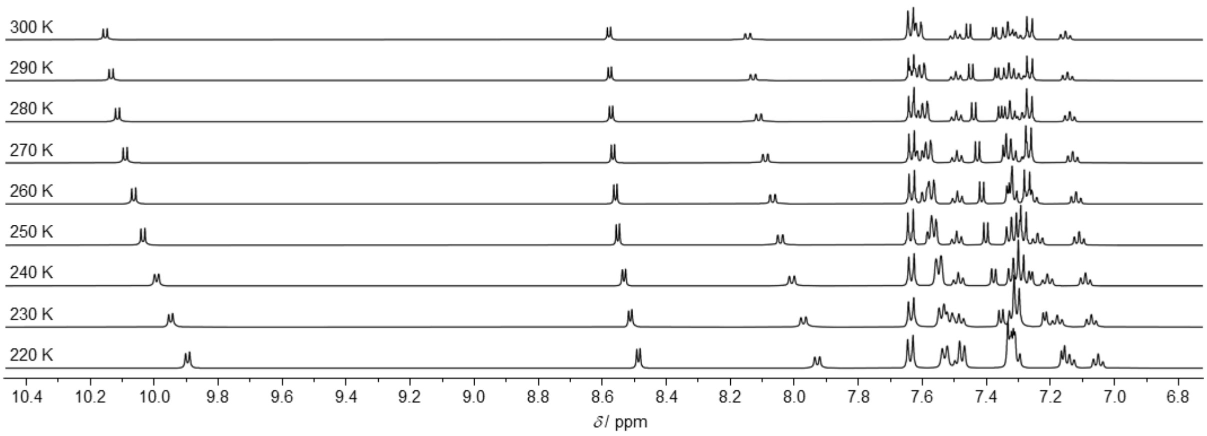

Figure S156: VT-^{1}H-NMR (400 MHz, CD_2Cl_2) of [**PtLHCl**] at 8 mM cooling from 300 K to 220 K.

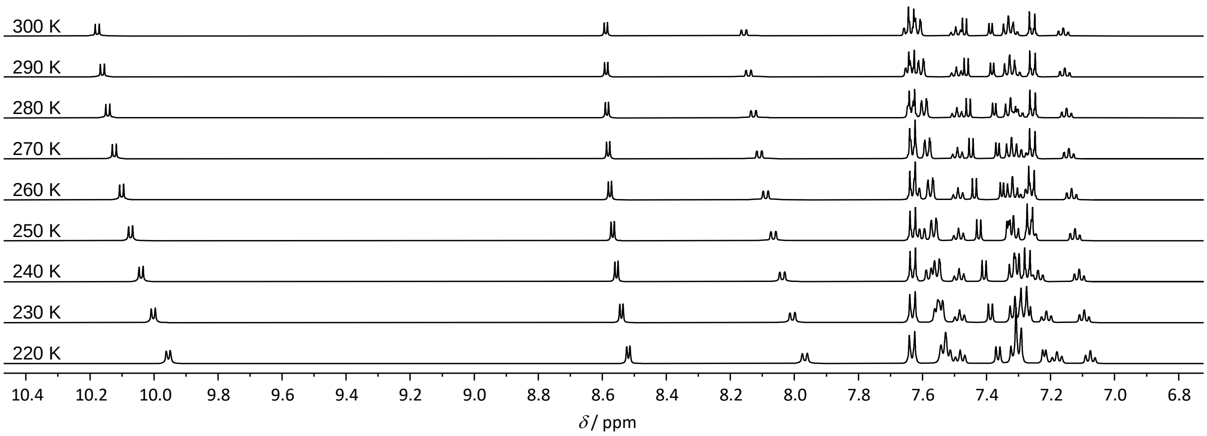

Figure S157: VT-^{1}H-NMR (400 MHz, CD_2Cl_2) of [**PtLHCl**] at 5 mM cooling from 300 K to 220 K.

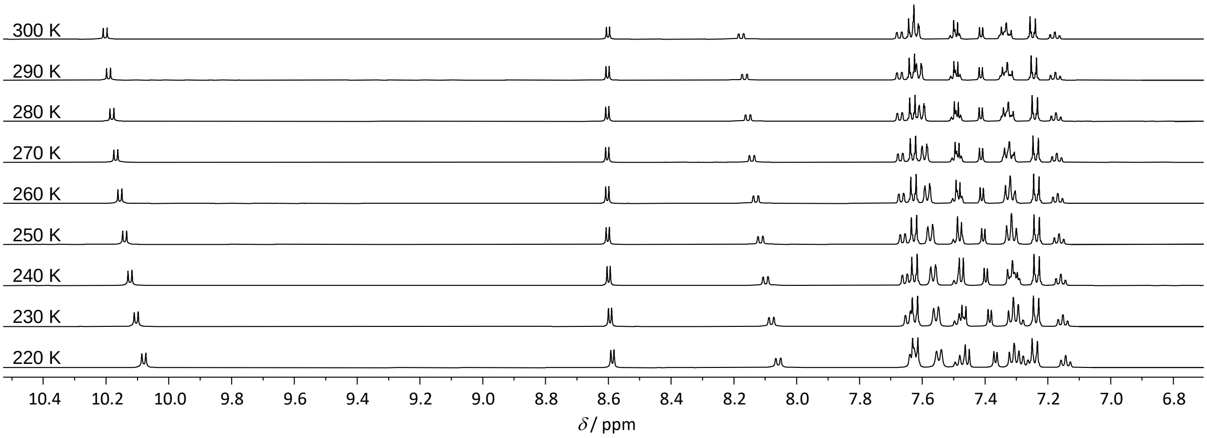

Figure S158: VT-^{1}H-NMR (400 MHz, CD_2Cl_2) of [**PtLHCl**] at 1 mM cooling from 300 K to 220 K.

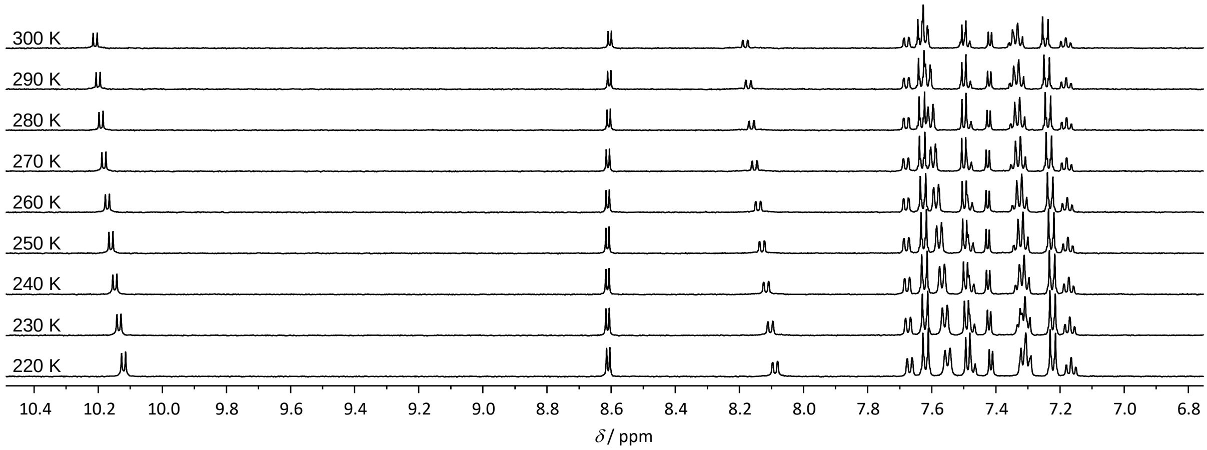

Figure S159: VT-^{1}H-NMR (400 MHz, CD_2Cl_2) of [**PtLHCl**] at 0.5 mM cooling from 300 K to 220 K.

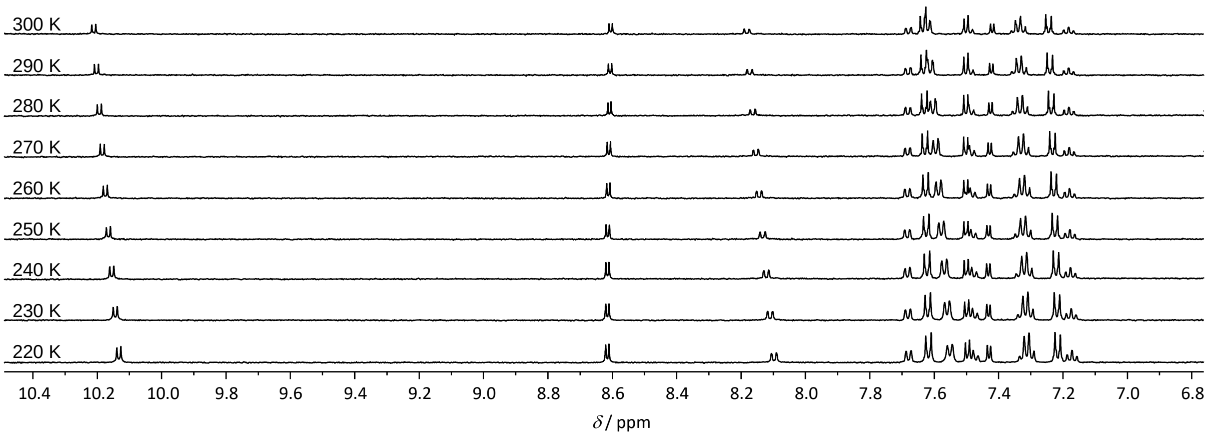

Figure S160: VT-^{1}H-NMR (400 MHz, CD_2Cl_2) of [**PtLHCl**] at 0.2 mM cooling from 300 K to 220 K.

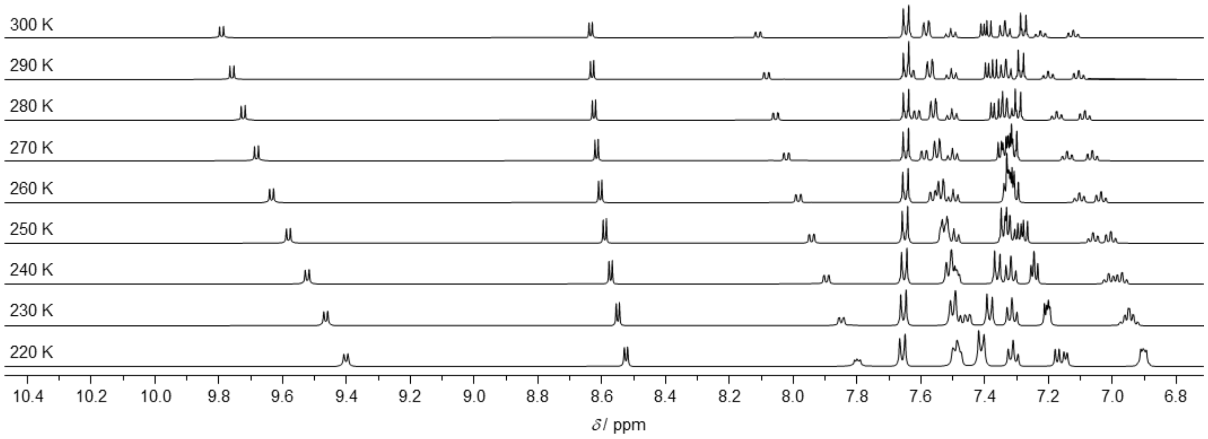

Figure S161: VT-^{1}H-NMR (400 MHz, CD_2Cl_2) of [**PtLHCN**] at 8 mM cooling from 300 K to 220 K.

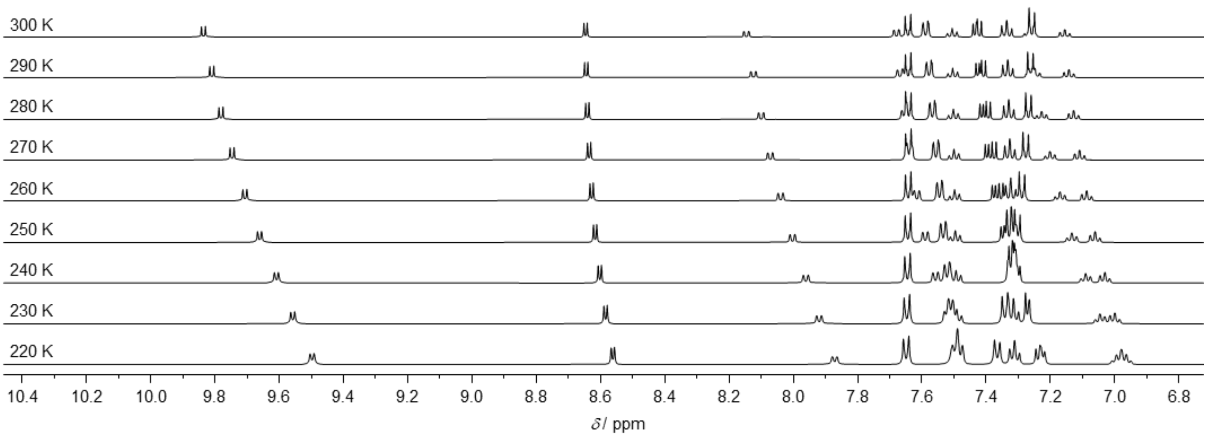

Figure S162: VT-^{1}H-NMR (400 MHz, CD_2Cl_2) of [**PtLHCN**] at 5 mM cooling from 300 K to 220 K.

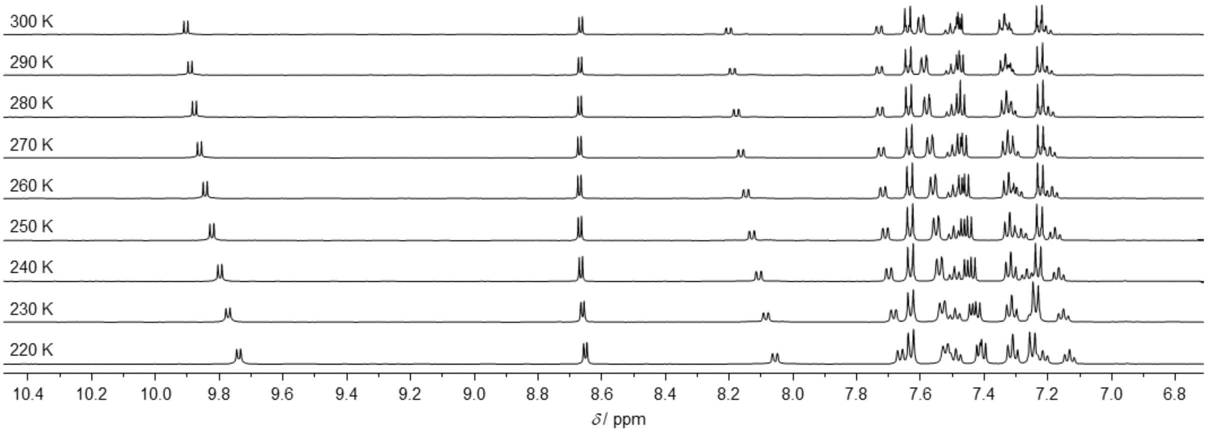

Figure S163: VT-^{1}H-NMR (400 MHz, CD_2Cl_2) of [**PtLHCN**] at 1 mM cooling from 300 K to 220 K.

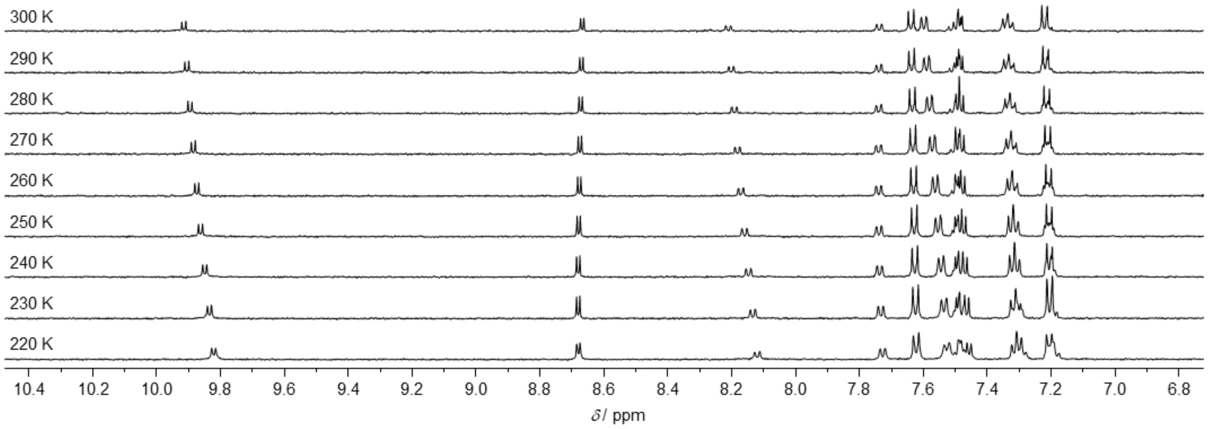

Figure S164: VT-^{1}H-NMR (400 MHz, CD_2Cl_2) of [**PtLHCN**] at 0.2 mM cooling from 300 K to 220 K.

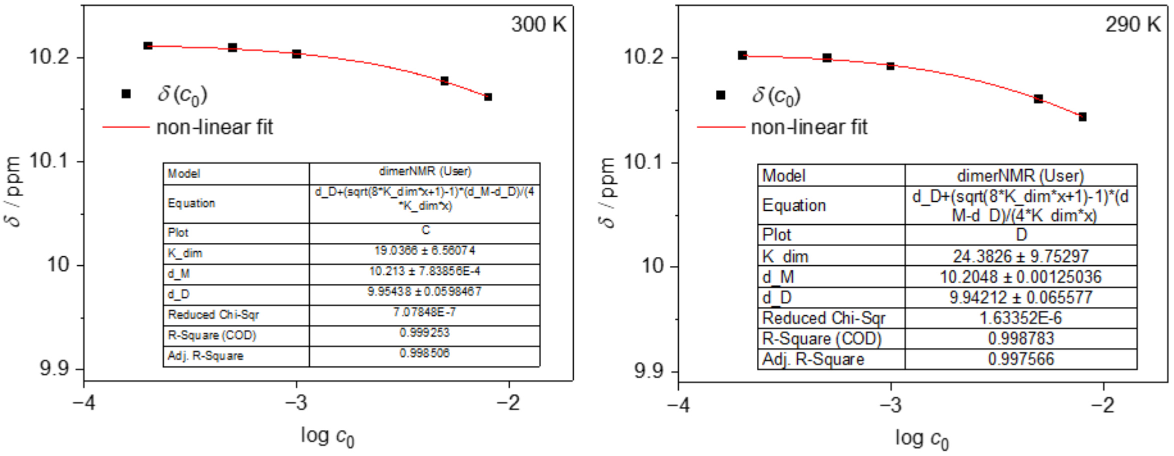

Figure S165: Chemical shifts δ *vs.* concentration c_0 for solutions of [**PtLHCl**] in DCM-d_2 at T = 300 K (left) and 290 K (right) fitted to the non-linear model shown in equation 2.

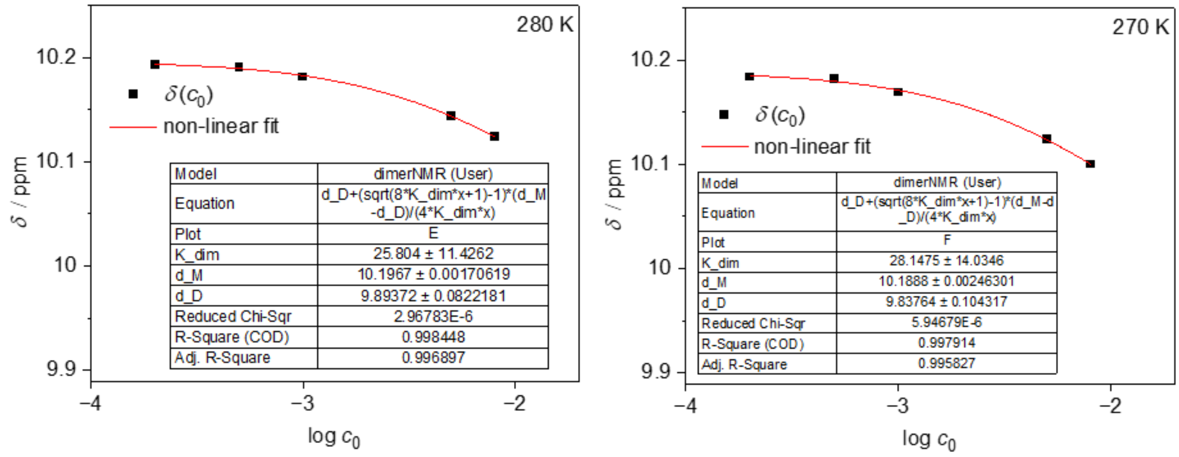

Figure S166: Chemical shifts δ *vs.* concentration c_0 for solutions of [**PtLHCl**] in DCM-d_2 at T = 280 K (left) and 270 K (right) fitted to the non-linear model shown in equation 2.

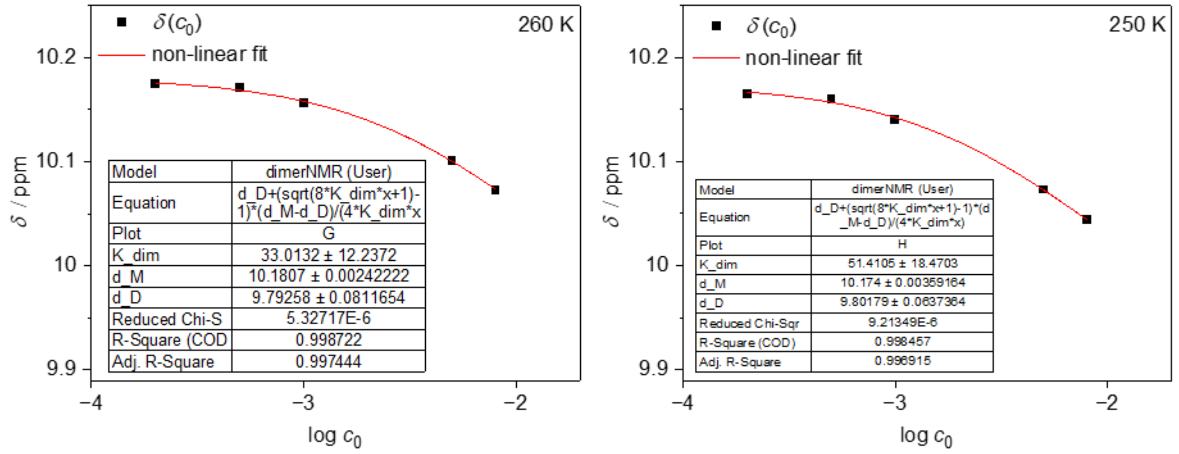

Figure S167: Chemical shifts δ *vs.* concentration c_0 for solutions of [**PtLHCl**] in DCM-d_2 at T = 260 K (left) and 250 K (right) fitted to the non-linear model shown in equation 2.

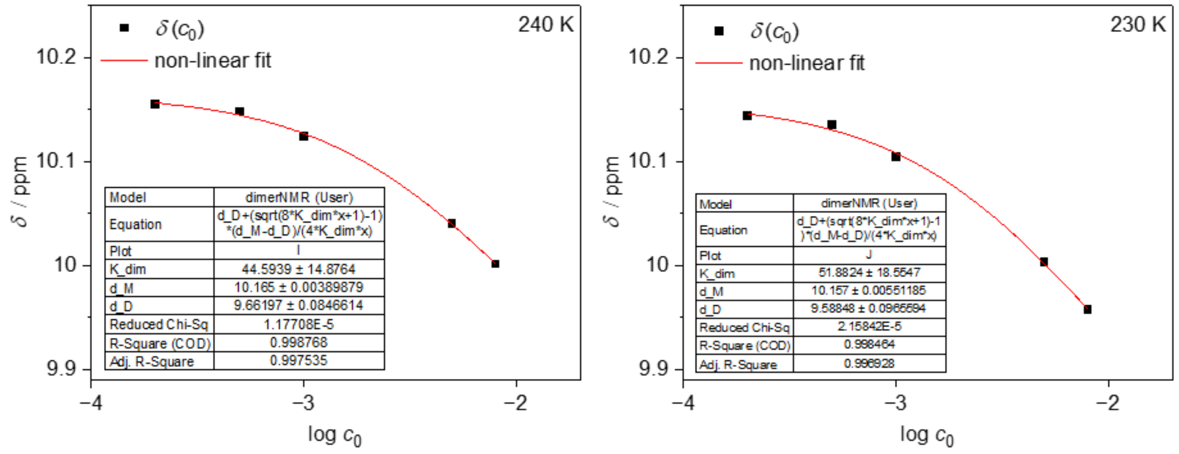

Figure S168: Chemical shifts δ *vs.* concentration c_0 for solutions of [**PtLHCl**] in DCM-d_2 at T = 240 K (left) and 230 K (right) fitted to the non-linear model shown in equation 2.

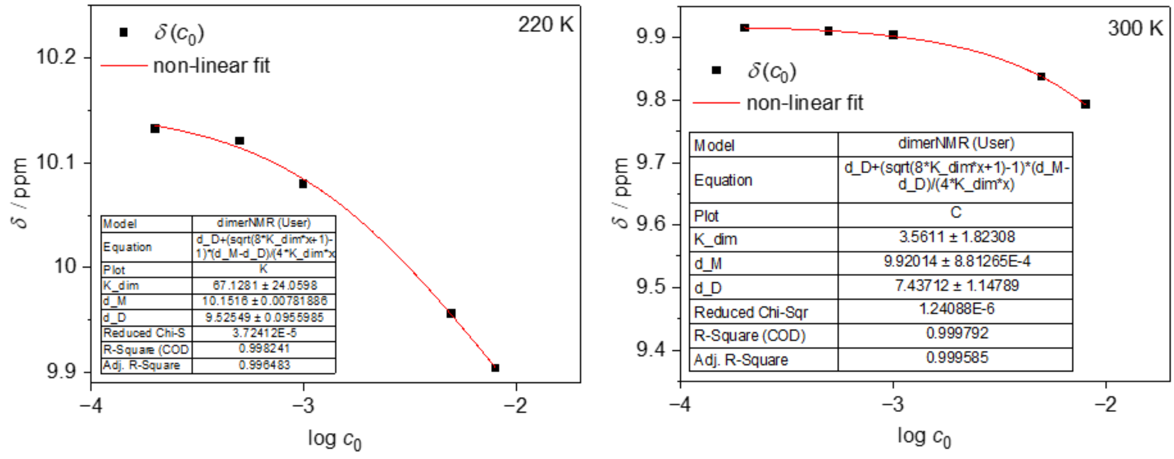

Figure S169: Chemical shifts δ *vs.* concentration c_0 for solutions of [**PtLHCl**] in DCM-d_2 at T = 220 K (left) and [**PtLHCN**] in DCM-d_2 300 K (right) fitted to the non-linear model shown in equation 2.

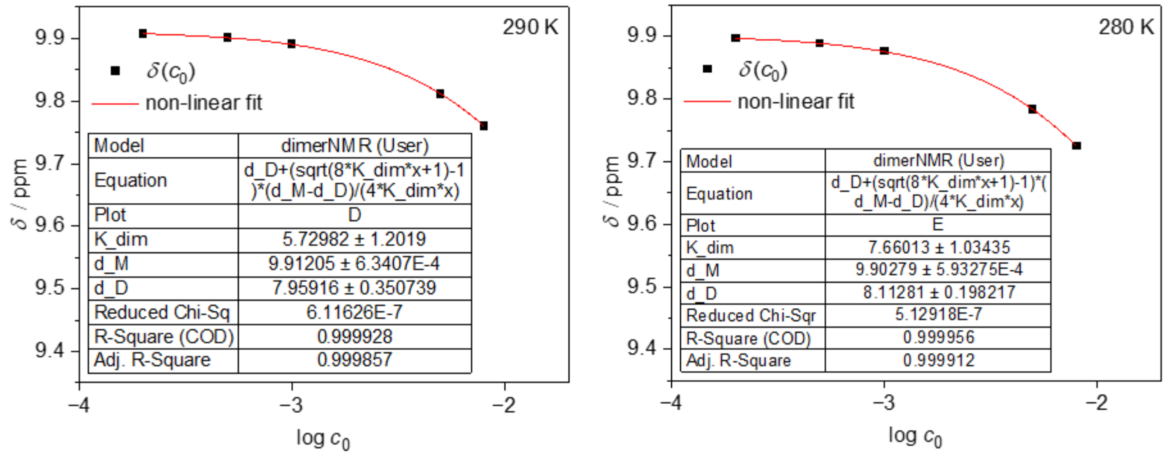

Figure S170: Chemical shifts δ *vs.* concentration c_0 for solutions of [**PtLHCN**] in DCM-d_2 at T = 290 K (left) and 280 K (right) fitted to the non-linear model shown in equation 2.

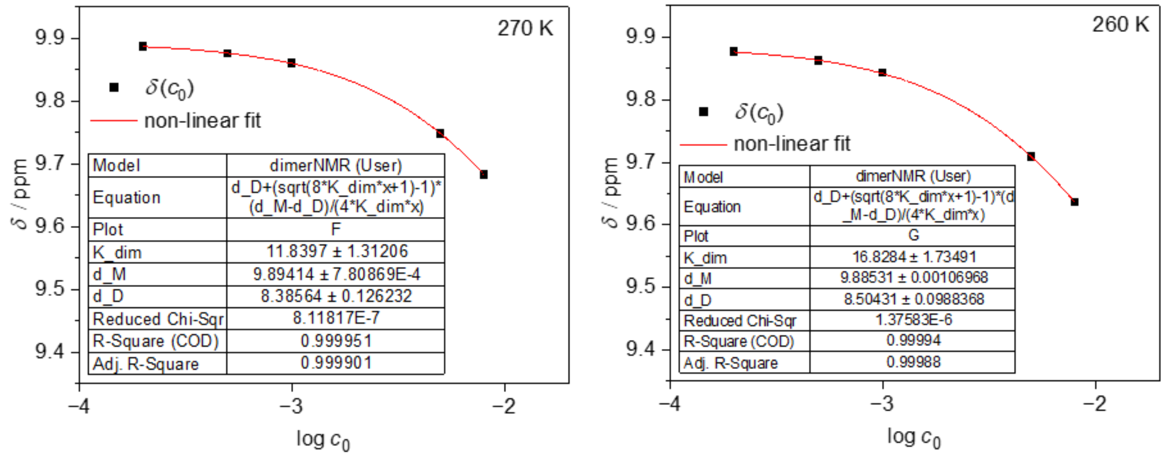

Figure S171: Chemical shifts δ *vs.* concentration c_0 for solutions of [**PtLHCN**] in DCM-d_2 at T = 270 K (left) and 260 K (right) fitted to the non-linear model shown in equation 2.

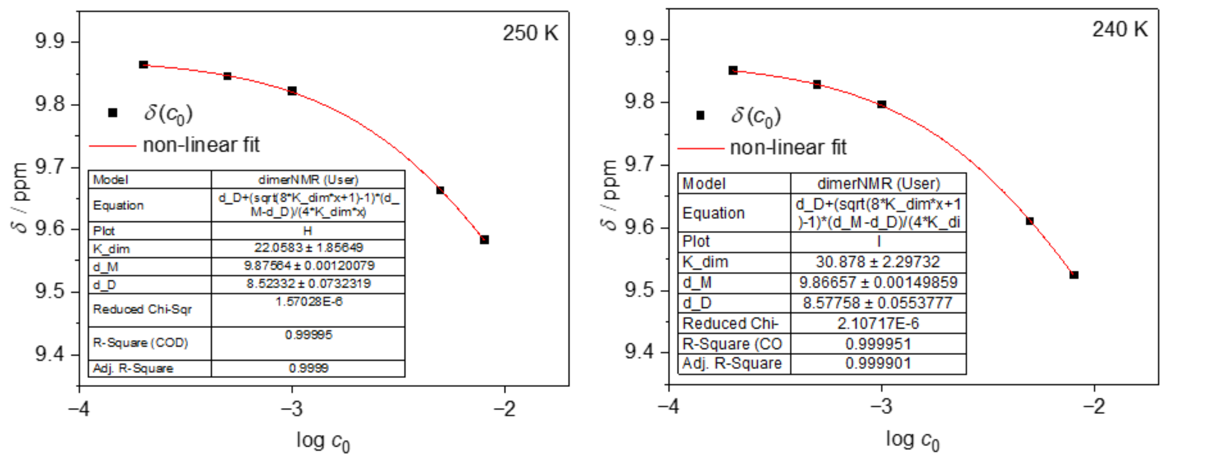

Figure S172: Chemical shifts δ *vs.* concentration c_0 for solutions of [**PtLHCN**] in DCM-d_2 at T = 250 K (left) and 240 K (right) fitted to the non-linear model shown in equation 2.

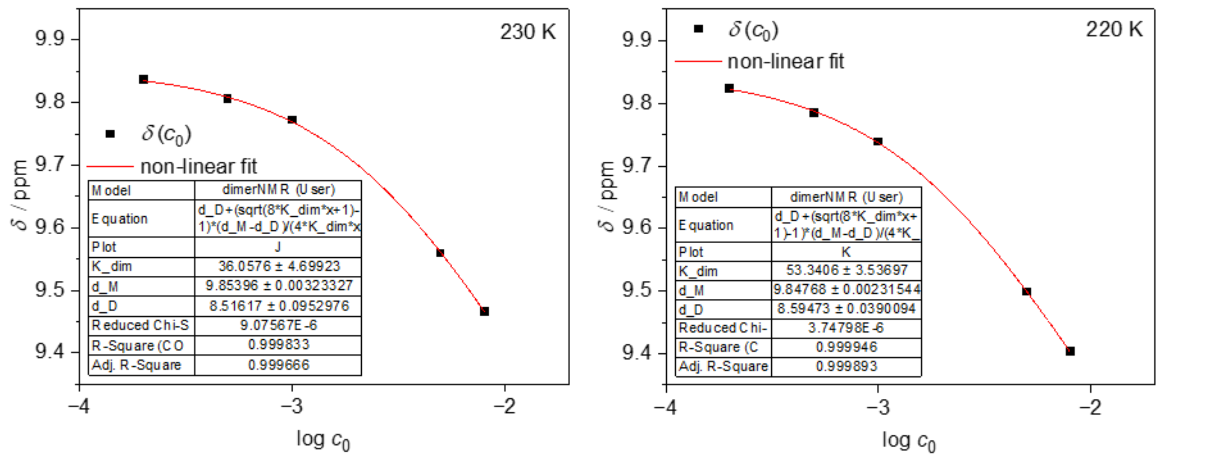

Figure S173: Chemical shifts δ *vs.* concentration c_0 for solutions of [**PtLHCN**] in DCM-d$_2$ at T = 230 K (left) and 220 K (right) fitted to the non-linear model shown in equation 2.

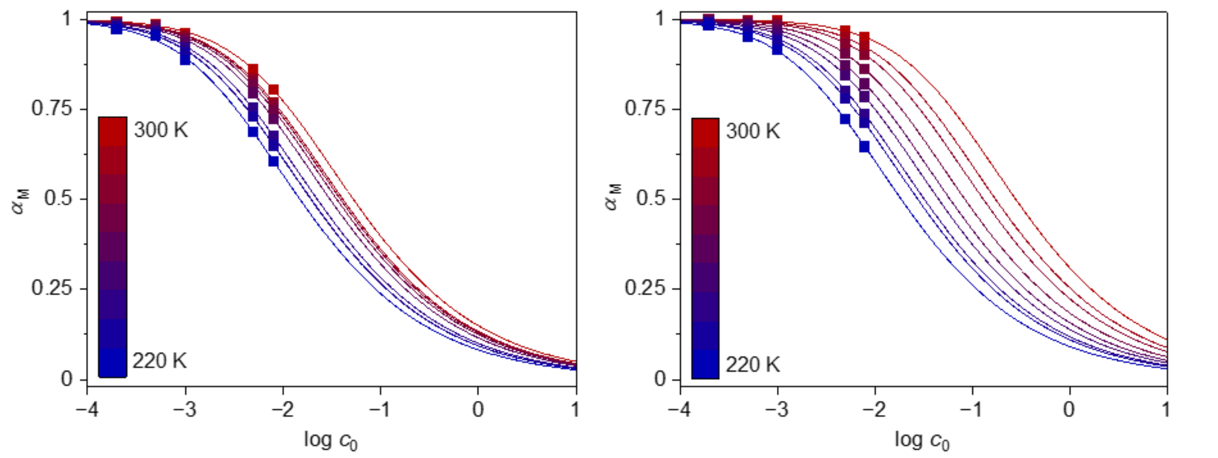

Figure S174: α_M (c_0) plots of [**PtLHCl**] (left) and [**PtLHCN**] (right) at 220 to 300 K (squares) and non-linear fits based on equation 2 (curves).

Table S15: Fitting parameters of [**PtLHCl**] calculated from chemical shifts δ (Figures S165-169).

Fitting parameter	T = 300 K	T = 290 K	T = 280 K	T = 270 K	T = 260 K	T = 250 K	T = 240 K	T = 230 K	T = 220 K
K_{dim} · M	19.037	24.383	25.804	28.148	33.013	51.411	44.594	51.882	67.128
δ_M / ppm	10.213	10.205	10.197	10.189	10.181	10.174	10.165	10.157	10.152
δ_D / ppm	9.954	9.942	9.894	9.838	9.793	9.802	9.662	9.588	9.525

Table S16: Fitting parametersof [**PtLHCN**] calculated from chemical shifts δ (Figures S169-173).

Fitting parameter	T = 300 K	T = 290 K	T = 280 K	T = 270 K	T = 260 K	T = 250 K	T = 240 K	T = 230 K	T = 220 K
K_{dim} · M	3.561	5.730	7.660	11.840	16.828	22.058	30.878	36.058	53.341
δ_M / ppm	9.920	9.912	9.903	9.894	9.885	9.876	9.867	9.854	9.848
δ_D / ppm	7.437	7.959	8.113	8.386	8.504	8.523	8.578	8.516	8.595

Table S17: α_M values of [**PtLHCl**] calculated from chemical shifts δ, δ_M and δ_D *via* equation 3.

c_0 / mM	T = 300 K	T = 290 K	T = 280 K	T = 270 K	T = 260 K	T = 250 K	T = 240 K	T = 230 K	T = 220 K
8	0.802767	0.768392	0.760164	0.747136	0.722489	0.650779	0.673944	0.648179	0.604496
5	0.860763	0.833098	0.826185	0.815483	0.794629	0.728697	0.751469	0.729087	0.687542
1	0.961291	0.951092	0.948326	0.943634	0.936333	0.908716	0.918447	0.906732	0.885574
0.5	0.98449	0.981542	0.981337	0.980656	0.97498	0.962453	0.966155	0.961257	0.951053
0.2	0.992223	0.989155	0.987939	0.986351	0.985286	0.975887	0.98007	0.977086	0.970217

Table S18: α_M values of [**PtLHCN**] calculated from chemical shifts δ, δ_M and δ_D *via* equation 3.

c_0 / mM	T = 300 K	T = 290 K	T = 280 K	T = 270 K	T = 260 K	T = 250 K	T = 240 K	T = 230 K	T = 220 K
8	0.948795	0.922139	0.900677	0.860031	0.81947	0.783601	0.734231	0.709999	0.645093
5	0.966515	0.948254	0.933079	0.90312	0.871606	0.842758	0.80095	0.779517	0.721713
1	0.993498	0.989219	0.985594	0.978029	0.969361	0.960333	0.946025	0.938735	0.912463
0.5	0.996318	0.994339	0.992298	0.987972	0.983119	0.97808	0.970074	0.96415	0.949974
0.2	0.998331	0.997412	0.996767	0.995264	0.993256	0.991391	0.987918	0.986575	0.980303

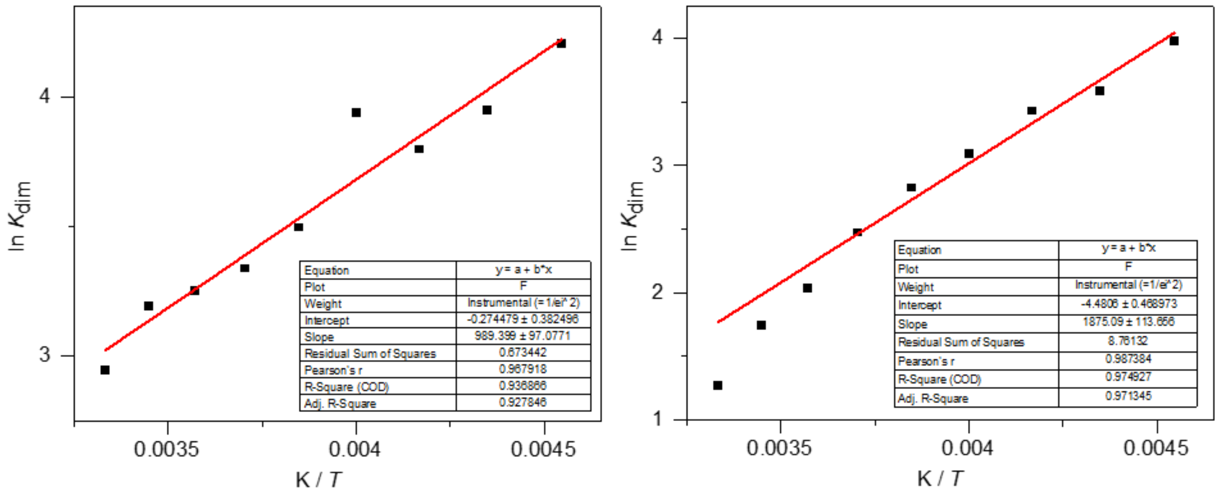

Figure S175: *van't Hoff* plot for complex [**PtLHCl**] (left) and [**PtLHCN**] (right).

V. Cytotoxicity test

The cytotoxicity of the synthesized complexes towards the HepG2 liver carcinoma cell line was assessed using the Resazurin assay according to the method of Johnson et al.[8] Cells were grown in Dulbecco's Modified Eagle Medium (DMEM) supplemented with 10 % (*v/v*) of fetal calf serum (FCS), 100 units/mL penicillin and 0.1 mg/mL streptomycin at 37 °C and 5 % CO_2 in a humidified atmosphere. For the assay, the cells were seeded in 96-well-plates at a density of 15,000 cells/200 µL medium with FCS/well. After 48 ± 2 h, the medium was replaced by serum-free medium with 1 % DMSO containing the tested substance in five concentrations. During every single experiment, 1 % DMSO served as solvent control and 0.01 % (*w/v*) saponin as positive control. After 24 ± 2 h of incubation, the medium with the substance was removed, the cells were washed once with phosphate buffered saline (PBS) and 0.44 mM resazurin solution in serum-free medium was added. The fluorescence was measured using a plate reader at 544 nm excitation/590 nm emission after 1 h of incubation. None of the complexes exhibited intrinsic fluorescence at this wavelength. Three biological replicates with five technical replicates each (n = 3 × 5) were performed for every substance.

Table S19: Relative cell viabilities of HepG2 cells according to the Resazurin cytotoxicity assay (mean viability and standard deviation, $n = 3 \times 5$). The nine synthesized complexes and oxaliplatin are displayed, saponin (PC, 0.1 % *w/v*) was used as positive control.

**Complex**		**PC**	**0.1 µM**	**1 µM**	**10 µM**	**50 µM**	**100 µM**
Oxaliplatin (reference)	Mean	-2 %	115 %	96 %	75 %	52 %	23 %
	Std. dev.	0 %	5 %	6 %	12 %	14 %	7 %
**[PtLH$_2$Cl$_2$]**	Mean	-2 %	105 %	85 %	73 %	Precipitation	
	Std. dev.	0 %	20 %	14 %	12 %		
**[PtLH$_2$Gly]**	Mean	-3 %	120 %	90 %	11 %	1 %	0 %
	Std. dev.	0 %	15 %	3 %	5 %	1 %	1 %
**[PtLH$_2$cbda]**	Mean	-2 %	123 %	95 %	87 %	89 %	Precipitation
	Std. dev.	1 %	11 %	12 %	12 %	10 %	
**[PtLH$_2$Tsgly]**	Mean	0 %	87 %	85 %	57 %	20 %	12 %
	Std. dev.	1 %	7 %	8 %	10 %	10 %	1 %
**[PtLHCl]**	Mean	0 %	82 %	83 %	82 %	Precipitation	
	Std. dev.	1 %	10 %	15 %	9 %		
**[PtLHCN]**	Mean	0 %	97 %	96 %	Precipitation		
	Std. dev.	1 %	5 %	7 %			
**[ReLH$_2$(CO)$_3$Br]**	Mean	-1 %	112 %	105%	105 %	Precipitation	
	Std. dev.	1 %	14 %	11 %	11 %		
**[ReLH$_2$(CO)$_3$Cl]**	Mean	0 %	81 %	76 %	Precipitation		
	Std. dev.	1 %	10 %	8 %			
**[ReLH$_2$(CO)$_3$CN]**	Mean	-1 %	108 %	97 %	98 %	Precipitation	
	Std. dev.	1 %	17 %	18 %	15 %		

References

[1] D. Coucouvanis, *Inorganic Syntheses*, Vol. 33, John Wiley and Sons, **2002**, p. 276.

[2] R. Romeo, L. M. Scolaro, V. Catalano, S. Achar, *Inorganic Syntheses*, Vol. 32, (Ed.: M. Y. Darensbourg), John Wiley & Sons, **1998**, pp 153–158.

[3] Apex4 (2021.4.0), Data Reduction and Frame Integration Program for the CCD Area-Detector System, Bruker AXS Inc., Madison, Wisconsin (USA), **2021**.

[4] Saint (8.40B), Area Detector Control and Integration Software, Bruker AXS Inc., Madison, Wisconsin (USA), **2021**.

[5] G. M. Sheldrick, Sadabs (2016/2), Bruker AXS Inc., Madison, Wisconsin (USA), **2016**.

[6] G. M. Sheldrick, SHELXTL, Structure determination software programs, Bruker AXS Inc., Madison, Wisconsin (USA), **1997**.

[7] C. F. Macrae, I. Sovago, S. J. Cottrell, P.T. A. Galek, P. McCabe, E. Pidcock, M. Platings, G. P. Shields, J. S. Stevens, M. Towler, P. A. Wood, Mercury 4.0: From Visualization to Analysis, Design and Prediction. *J. Appl. Crystallogr.* **2020**, pp. 226–235.

[8] M. K. McMillian, L. Li, J. B. Parker, L. Patel, Z. Zhong, J. W. Gunnett, W. J. Powers, M. D. Johnson, *Cell Biology and Toxicology*, **2002**, pp. 157–173.
